# 116th Annual Meeting, Medical Library Association, Inc., Toronto, ON, Canada, May 13–18, 2016

**DOI:** 10.5195/jmla.2017.123

**Published:** 2017-01

**Authors:** Nicole Mitchell

## INTRODUCTION

The Medical Library Association (MLA) held its 116th annual meeting in Toronto, Ontario, Canada, May 13–18, 2016, at the Metro Toronto Convention Centre, in conjunction with the Canadian Health Libraries Association/Association des bibliothèques de la santé du Canada (CHLA/ABSC) and the International Clinical Librarian Conference (ICLC). The meeting theme was “Mosaic: Be Part of the Big Picture!” Total attendance for the meeting was 1,335, with 327 participating in continuing education courses. Additional meeting content—including the meeting program and various electronic presentations from the business meetings, plenary sessions, poster sessions, and section programs—can be accessed by all meeting registrants via the MLA ’16 website.

## WELCOME TO MLA ’16

### Sunday, May 15, 2016

MLA President Michelle Kraft, AHIP, welcomed attendees to the 2016 annual meeting, noting that the meeting theme, “Mosaic: Be Part of the Big Picture,” reflected the diversity of the organizations who partnered to plan the joint meeting. She updated the audience on the new features of the annual meeting such as only one Business Meeting so there is more time for programming and the new Presidents’ Awards Dinner. President Kraft also reminded attendees to visit the exhibit hall to show support to exhibitors.

President Kraft then introduced Jeanna Hough, president of the Canadian Health Libraries Association/Association des bibliothèques de la santé du Canada (CHLA/ABSC) who welcomed attendees to Toronto. She then introduced Sarah Sutton and Pip Dival, liaisons, who greeted attendees on behalf of the International Clinical Librarian Conference (ICLC).

President Kraft returned to the podium to introduce the 2016 Joint Planning Committee and the 2016 Local Assistance Committee: Anne K. Seymour, cochair, 2016 Joint Planning Committee, and director, Welch Medical Library, Johns Hopkins University, Baltimore–Maryland; Jeff Mason, cochair, 2016 Joint Planning Committee, and horizon scanning officer, Canadian Agency for Drugs and Technologies in Health, Ottowa, Ontario, Canada; Vanessa Kitchin, cochair, 2016 Local Assistance Committee, and research analyst, Ontario Medical Association–Toronto, Canada; and David Lightfoot, cochair, 2016 Local Assistance Committee and information specialist, Health Sciences Library, Saint Michael’s Hospital, Toronto, Ontario, Canada. President Kraft thanked everyone for their work on the meeting and expressed appreciation to the vendors who contributed to the meeting’s success.

Anne K. Seymour and Jeff Mason: Good morning everyone and welcome to Toronto. We are so excited for you all to join us here at “Mosaic 2016: Be Part of the Big Picture.” More than three years ago, representatives of three organizations, each with their own meeting traditions, came together as the 2016 Joint Planning Committee. It has been a privilege to be entrusted by you to knit together the best of what MLA, CHLA/ABSC, and ICLC have to offer.

We are thrilled to officially kick off a meeting that delivers something for everyone. How appropriate is it that we are bringing together three distinct organizations in a city known for its diversity of peoples, cultures, and lifestyles? We’d like to acknowledge the members of the 2016 Joint Planning Committee. This meeting would not have happened without their hard work and dedication. Would the members of the 2016 Joint Planning Committee please stand to be recognized? Amy Chatfield, liaison, Section Council; Orvie Dingwall, AHIP, representative, CHLA/ABSC; Pip Divall, liaison, ICLC; Michelle Henley; Carrie L. Iwema, AHIP; Vanessa Kitchin, cochair, Local Assistance Committee; Michelle Kraft, AHIP, liaison, MLA Board of Directors; David Lightfoot, cochair, Local Assistance Committee; Elizabeth Morton; Rikke Ogawa, AHIP; Dale Prince; Gurpreet K. Rana; Melissa Ratjeski; Connie Schardt, liaison, Continuing Education Committee; Brenda L. Seago; and Sara Sutton, liaison, ICLC.

We’d also like to give a special thanks to Ray Naegele, director, Financial and Administrative Services, MLA, and staff liaison; all the staff at MLA headquarters; and Tina Vickery and Paul Graller, meeting planners, HEI; for their support in keeping the planning process running smoothly. And I would like to give a personal thanks to our Canadian hosts and my cochair Jeff for schooling me about all things Canadian. Once again, thank you for coming to Toronto. Let’s not wait another fifteen years to do this again.

Next, we would like to invite David Lightfoot and Vanessa Kitchin to bring greetings on behalf of the 2016 Local Assistance Committee.

Vanessa Kitchin: Hi everyone. I’m so excited to welcome you to Toronto. As a native of this wonderful city, I can’t wait for you to get out and explore. Hopefully, you will have some downtime to check out the cultural events, museums, the plethora of restaurants, the shopping, and especially, the people watching. We have some great library tours organized as well as a really comprehensive restaurant guide, so welcome and enjoy. I can’t believe it’s finally here.

David Lightfoot: Hello. Welcome. Just wanted to assure you that the weather is getting warmer. And I’d like to take a few minutes to thank some of our volunteers. It was sort of an embarrassment of riches, we had so many wonderful people. Thank you very much for your help. Welcome.

Anne Seymour then welcomed Sandy Iverson who introduced Nancy Cooper to talk about this year’s community service project, “Promoting Health Professions for First Nation’s Kids,” which was inspired by work done at the University of Montreal. More information about donating books or money to the project was located in attendee bags and the hospitality desk.

President Kraft returned to the podium to recognize and thank meeting planners and all the vendors who generously contributed to the meeting’s success.

Anne Seymour then introduced President Kraft who gave her presidential address.

## MLA PRESIDENTIAL ADDRESS: MICHELLE KRAFT, AHIP (PLENARY SESSION 1)

Michelle Kraft, AHIP: By now, we have heard the great news that the National Library of Medicine (NLM) has a new director, Patricia Flatley Brennan. Dr. Brennan is the first woman and the first nonphysician to lead NLM and is expected to begin her position in August. Her credentials as a registered nurse, informatician, industrial engineer, and renowned researcher make her uniquely qualified to serve as the next director of the world’s largest biomedical library.

Dr. Brennan has successfully bridged her commitment to providing high-quality patient care as a practicing nurse with an entrepreneurial approach that uses technology and information systems to actively engage patients in furthering their own health care and outcomes. MLA congratulates Dr. Brennan on her appointment, and we look forward to continuing our close partnership with NLM for many years to come.

So let’s get started. Last year, I had a *Star Wars* theme. This year, it’s not a *Star Wars* theme. I had to scale it down a little bit. But I know you guys remember last year and the whole thing was “Do, or do not. There is no try.” And, man, we’ve been doing a lot.

It’s a little ambitious. I want to remind people that it’s a three-year rolling strategic plan. We have four current goals and the MLA sections, we have a goal that’s focused with them, defining what is strategic for them. And the sections and the Section Council have already been talking about this. It’s a member centric approach. The website is directed more toward members and what is available to them. We want to build our community and communication, and we want to build up education. So no wonder all of this is not just a one and done kind of thing. It’s a three-year plan. So hopefully three years from now, we can put a little check mark. But I want to tell you all that we’ve made a lot of accomplishments even now.

Remember this little rocket ship and my great drawing. Don’t forget: as things roll off, we’ll be adding new things. This is not one and done. This is not three and done. We will continue to better MLA. As things come along, we will add them to the strategic plan.

### Goal Number 1

So goal number 1: what does MLA do. Well we’ve done a lot. Thanks to the Strategic Priorities Task Force led by Katherine R. Stemmer Frumento, AHIP, they have presented the recommendations from the Futures Task Force. We redesigned the annual meeting, as you all heard, based on member feedback. As we said, we had over 540 submissions. We’ve reduced the MLA Business Meeting time to have more open or program time. We’ve evaluated MLA international activities. We’re defining the strategic objectives. Please read the Full Speed Ahead blog. That’s where you’ll find a lot of our posts about different things we’re working on, including the international activities. We’re empowering information professionals in developing areas. We’re enabling MLA members to play positive roles globally. We’re working to promote the awareness of international issues. Don’t forget, there is an open forum today at 4:30 p.m. to 5:25 p.m. in room 202B, if you’re interested in the international aspect of MLA.

So we have a bylaws thing going on. I don’t know if you got the email. We’ve been simplifying the rigor for sections and what they need to reply, the governance and how they want to set things up. We’re also working on the bylaws and special rules of amendments. So everybody, if you’re not going to the international forum and you’re not going to the other forum, you have go to the bylaws forum that’s open today from 4:30 p.m. to 5:25 p.m. in room 205B. Every MLA member needs to go to the Business Meeting on Tuesday to vote on the bylaws. So that’s just a small reminder.

### Goal 2: New Professionals

Goal 2 has not been dropped; it has just merged with all the other goals. When we were looking at things and going through strategies and everything, we thought getting more professionals, getting more MLA members, that is what we want to do, that’s a strategy. But it turns out engaging our professionals, getting new professionals, getting people involved, that’s essential to every goal that we have. So instead of dropping it as a goal, it’s just been integrated and merged into the other goals.

### Goal 3: Education

The competencies task force led by Paula Raimondo, AHIP, has been very helpful for creating the competencies for lifelong learning for librarianship. We’ve updated the realms or domains of professional competencies. We’re making them measurable. We’re having tiered competencies, and we’ll be building a new curriculum. Teresa L. Knott, AHIP, will talk more about this in her inaugural address. But if you ever thought of education, and what I mean by tiered competencies and measurable and a curriculum, again I’m dating myself here, but remember when you went to college, and you had the college course book, and you could look and see this is psychology and here’s a 100-, and a 200-, and a 300-level class, that’s kind of what we want to do with our education. Have it in a tiered way so you know what kind of options you have within the CE.

### Goal 4: Technology

We’ve done a lot with technology. We kind of blew up the old MLA site. It was like our old printer here. And we got a new MLA site. There are 50 websites, 120 email discussion lists, 15,000 pages, thankfully not printed, incorporated into the MLANET. Traffic has doubled, and we’ve also saved $150,000 in yearly costs. We’re working on a learning management system (LMS) and authoring tools. That’s around September 2016. And we will be looking at meeting-related systems in 2017. So the next meeting.

### Goal 5: Research

The Research Imperative Task Force, which was led by Susan Lessick, AHIP, FMLA, and they’ve created a MLA Award for Research Advancement in Health Sciences Librarianship. They are working with the Task Force to Review MLA’s Competencies for Lifelong Learning and Professional Success to revise the research competencies. They’re planning on the creation of the MLA Research Training Institute so that librarians, health sciences librarians, will have a better understanding of and will be able to help and do more research. They’ve created lists of studies showing the value and impact of health sciences librarians. And they’ve enhanced the research mentoring program. Information on the mentoring program will be publicized this summer, and the lists showing the value and impact of health sciences librarians will also be on MLA’s website this summer.

So I thank you all for the journey. Somebody was driving the bus so to speak, the RV. It wasn’t always me. It was the committee chairs, the section chairs, the MLA Board, the members, everybody had a chance at helping us bring us down this journey this year. I want to thank the MLA members. On behalf of the Board of Directors, I want to take this opportunity to thank all of you, the MLA members, for the dedication and hard work at making MLA everything that it is and can be. Without you serving in all of the various ways, we wouldn’t have this great organization. You guys were also driving the RV. I thank MLA staff. The staff works very hard every day to support the association and our members. Over the years, I have come to know them and rely upon them for their expertise and for their humor in dealing with so many diverse and unique situations. And they’ve had to deal with me and my humor. So thank you. Will staff members present please stand and be recognized?

I thank the Cleveland Clinic library staff for all of their support this year. Without them, I wouldn’t be here. Without their support and mentoring, I would not be here doing what I am doing right now or what I have been doing. And I know there are two librarians here from the Cleveland Clinic. I’m not going to make you stand and embarrass yourselves but thank you.

And I thank the best family ever. Thanks to my mom, my mother-in-law, for helping out. Thanks to my children—Peter, Sean, and Rachel—who now have come to expect Garrett’s popcorn every time I travel. And a thousand thank yous to my husband Mike who will be here tomorrow. He is my partner in everything. His only questions are “What is on the schedule?” and “How can we make it work?” None of this would be possible without him and the rest of my family.

During the past year, we were saddened to learn that several valued members of our association have died. Their counsel and friendship will deeply be missed. We have produced this short video to honor the memory of these MLA members: Jacqueline D. Bastille, AHIP, FMLA, January 21, 2016; Sara Cole Brown, AHIP, FMLA, August 8, 2015; Nina Dougherty, March 5, 2016; Jack D. Key, December 22, 2015; Erich Meyerhoff, AHIP, FMLA, December 26, 2015; Katherine Lynne Schilling, March 17, 2016; Ann Von Segen, June 26, 2015; and Sunny Worel, July 1, 2014.

Now for the final item on today’s agenda. On behalf of the Board of Directors, I want to take this opportunity to express my thanks to all of you, everyone here, in attendance. You work hard every day, in and out, to support the association and our members. Over the years, I have come to know many of you and tried to come to know more of you. You are always willing and able to work with MLA and other board members, and the president, and the staff, and other association members in efforts on behalf of the membership and everybody in the profession. So I want you all, I’m not going to make you all stand, but I want you all to give yourselves a round of applause.

President Kraft then concluded the opening session and encouraged everyone to attend the John P. McGovern Award Lecture delivered by Ben Goldacre, an award-winning writer, broadcaster, and medical doctor who specializes in unpicking scientific claims made by scaremongering journalists, government reports, pharmaceutical corporations, public relations companies, and quacks.

## OTHER PLENARY SESSIONS

All plenary session videos and slides are available online to MLA ’16 registrants from the MLA ’16 website. See www.eventscribe.com/2016/MLA for more information about all speakers and sessions.

### 2. Sunday, May 15: The John P. McGovern Award Lecture

Introduction: Jeff Mason, cochair, 2016 Joint Planning Committee, and horizon scanning officer, Canadian Agency for Drugs and Technologies in Health (CADTH), Ottawa, Ontario, Canada

*Bad Science, Better Data:* **Ben Goldacre,** writer, broadcaster, and medical doctor

### 3. Monday, May 16: The Janet Doe Lecture for 2016

Introduction: Barbara A. Epstein, AHIP, FMLA, director, Health Sciences Library System and Middle Atlantic Region, National Network of Libraries of Medicine, University of Pittsburgh, Pittsburgh, PA

*We Can Be Heroes: MLA’s Leadership Journey(s):* **M.J. Tooey, AHIP, FMLA,** associate vice president, Academic Affairs, and executive director, Health Sciences and Human Services Library, University of Maryland–Baltimore

### 4. Wednesday, May 18: Plenary Session 4

Introduction: Anne K. Seymour, cochair, 2016 Joint Planning Committee, and director, Welch Medical Library, Johns Hopkins University, Baltimore, MD

**Ellen Jorgensen,** cofounder and director of Genspace, a nonprofit community laboratory dedicated to promoting citizen science and access to biotechnology

## PRESIDENTS’ AWARDS CEREMONY AND DINNER

The Presidents’ Awards Dinner was held on Tuesday, May 17, 2016, from 6:30 p.m. to 10:00 p.m. President Michelle Kraft, AHIP, welcomed attendees and award recipients.

President Michelle Kraft: Good evening! Greetings to you all and welcome to the MLA ’16 Presidents’ Awards Dinner. Today, we honor our colleagues who have made outstanding contributions to the profession. The names of this year’s recipients are listed in the awards program for your reference and highlighted on the monitors in the room. At this time, Canadian Health Libraries Association/Association des bibliothèques de la santé du Canada (CHLA/ABSC) President Jeanna Hough presented the CHLA/ABSC awards.

This was an exceptional year for members of the health information profession. We have members from across the country, and world, who have excelled in every facet of librarianship, and we are pleased to present them and recognize their accomplishments.

Tonight, I thank Nandita S. Mani, AHIP, chair of the Awards Committee, and Steven Douglas, AHIP, chair of the Grants and Scholarships Committee, and all jury members for your time and effort. Nandita, Steven, and members of the juries seated in the audience, would you please stand? Thank you.

I would like to take this opportunity to acknowledge the 2016 MLA section and chapter award winners. MLA sections and chapters annually award and honor members for their outstanding work in their sections or chapters. Would all section and chapter award winners in the audience today please stand to be recognized? Thank you.

The Majors/MLA Chapter Project of the Year Award recognizes excellence, innovation, and contributions to the profession of health sciences librarianship. The Midcontinental Chapter was selected to receive this award for its “Virtual Connections: Wherever You Are—You’ll Be There” conference.

The MLA Section Project of the Year Award is awarded to an MLA section that demonstrates creativity, ingenuity, cooperation, and leadership within the framework of the mandate of the section. The 2016 winner is the Veterinary Medical Libraries Section for its project “Promoting and Advocating for the Profession: Creating a Multi-Faceted Illustrated History of the Veterinary Medical Libraries Section (VMLS) and Its Impact.”

The MLA Rising Stars program identifies members who will spend a year in a curriculum designed to help emerging association members develop their potential to provide leadership to MLA at a national level. We are pleased to introduce the 2016–2017 Rising Star cohort: Phill Jo, Robert M. Bird Health Sciences Library, University of Oklahoma Health Sciences Center–Oklahoma City; Rachel Charlotte Lerner, Health Sciences Library, Quinnipiac University, Hamden, CT; Tony Nguyen, AHIP, National Network of Libraries of Medicine Southeastern/Atlantic Region, Health Sciences and Human Services Library, University of Maryland–Baltimore; and Gregg A. Stevens, AHIP, Pharmacy and Health Professions, Monroe F. Swilley Jr., Library, Mercer University, Atlanta, GA.

Sponsored by EBSCO Information Services, the 4 EBSCO/MLA Annual Meeting Grants provide up to $1,000 each to enable medical librarians new to the profession and working in health sciences libraries to attend MLA annual meetings. This year’s winners are: Nicole Capdarest-Arest, Lane Medical Library, Stanford University, Palo Alto, CA; Elizabeth G. Hinton, AHIP, Rowland Medical Library, University of Mississippi Medical Center–Jackson; Karin Saric, Norris Medical Library, University of Southern California–Los Angeles; and Peace Ossom Williamson, Central Library, University of Texas–Arlington.

The Ysabel Bertolucci MLA Annual Meeting Grant recognizes a health sciences librarian who is involved in nursing, allied health, consumer health, or international librarianship. We are pleased to present this year’s grant to Jennifer Leigh Kaari, University Hospital, Newark, NJ.

The MLA Continuing Education Grant provides monetary awards to MLA members to develop knowledge of the theoretical, administrative, or technical aspects of librarianship. We are pleased this year to acknowledge Andrea C. Kepsel, AHIP, MSU Libraries, Michigan State University–East Lansing, and Nathalie Reid, AHIP, National Center for Child Traumatic Stress, University of California–Los Angeles.

Established in 1996, the Hospital Libraries Section/MLA Professional Development Grant provides librarians working in hospital and similar clinical settings with the support needed for educational or research activities. One of the 2016 recipients is Lee Luniewski, Melisa Reasner McGuire Health Sciences Library, Scripps Health–Mercy Hospital, San Diego, CA.

The Medical Informatics Section/MLA Career Development Grant awards one individual a grant to support a career development activity that will contribute to the advancement of the field of medical informatics. This year’s recipient is Carrie L. Iwema, AHIP, Health Sciences Library System, University of Pittsburgh, Pittsburgh, PA.

The MLA Scholarship is granted to a student entering an American Library Association–accredited library school. The 2016 recipient, Daron MacQueen, is a student at the University of Western Ontario–London, Canada.

This year’s presenter of the Janet Doe Lecture, M.J. Tooey, AHIP, FMLA, is associate vice president, Academic Affairs, and executive director, Health Sciences and Human Services Library, at the University of Maryland–Baltimore. She is also the director of the National Network of Libraries of Medicine’s Southeastern/Atlantic Regional Medical Library, under contract with the National Library of Medicine at the National Institutes of Health. Tooey delivered this year’s lecture, “We Can Be Heroes: MLA’s Leadership Journey(s),” on Monday. As expected, it was indeed a great lecture.

The Ida and George Eliot Prize recognizes a work published in the preceding calendar year that has been judged most effective in furthering medical librarianship. Sandra De Groote, AHIP, University Library, University of Illinois–Chicago; Mary Shultz, Savitt Medical Library, University of Nevada School of Medicine–Reno; and Neil R. Smalheiser, Department of Psychiatry, University of Illinois–Chicago, are all the winners this year for their article “Examining the Impact of the National Institutes of Health Public Access Policy on the Citation Rates of Journal Articles.”

The Murray Gottlieb Prize was established to recognize and stimulate health sciences librarians’ interest in the history of medicine. The 2016 recipient is Laura Menard, Irwin Library, Butler University, Indianapolis, IN, for her paper, “History of Disorders of Sex Development (DSD) and Medical Intervention.” The Murray Gottlieb Prize was established in 1956 by a gift from Ralph and Jo Grimes of the Old Hickory Bookshop, Brinklow, MD. Since 2010, this award has been sponsored by the MLA History of the Health Sciences Section. MLA is pleased to announce that the prize has been renamed and will now be known as the Erich Meyerhoff Prize to honor late longtime MLA member Erich Meyerhoff.

The Estelle Brodman Award for the Academic Medical Librarian of the Year is given to a member who has made outstanding contributions to academic medical librarianship. This year’s award is presented to Blair Anton, AHIP, Welch Medical Library, Johns Hopkins University, Baltimore, MD.

The Lois Ann Colaianni Award for Excellence and Achievement in Hospital Librarianship is given to a member who has made significant contributions to the profession through overall distinction in hospital librarianship. This year’s award is bestowed on Donna B. Flake, AHIP, FMLA, SEAHEC Medical Library, South East Area Health Education Center, Wilmington, NC.

Named for one of the profession’s most revered leaders, the Lucretia W. McClure Excellence in Education Award recognizes outstanding practicing librarians or library educators in the field of health sciences librarianship and informatics. Jeffrey T. Huber, School of Library and Information Science, University of Kentucky–Lexington, is this year’s recipient.

The Louise Darling Medal for Distinguished Achievement in Collection Development in the Health Sciences recognizes accomplishment in collection development for the health sciences. The 2016 winner is the Association of Vision Science Librarians. The association is a special interest group of both the Association of Schools and Colleges of Optometry and MLA.

The purpose of the Cunningham Memorial International Fellowship is to assist in the education and training of health sciences librarians from countries outside the United States and Canada. The 2016 recipient is Prabavathy Naido, University of KwaZulu-Natal, Medical School Library, Durban, South Africa.

The Eugene Garfield Research Fellowship was established to promote and support research in the history of information science in the medical or health sciences. This year’s recipient is Elizabeth Connor, AHIP, The Citadel, Military College of South Carolina–Charleston.

The David A. Kronick Traveling Scholarship is awarded annually to cover expenses involved in traveling to medical libraries in the United States or Canada for the purpose of studying a specific aspect of health information management. This year’s recipient is Len Levin, AHIP, Lamar Soutter Library, University of Massachusetts Medical School–Worcester.

The Donald A. B. Lindberg Research Fellowship funds research aimed at expanding the research knowledgebase, linking the information services provided by libraries to improved health care and advances in biomedical research. This year’s fellowship is awarded to Alisa Surkis, NYU Health Sciences Library, New York University School of Medicine–New York.

The T. Mark Hodges International Service Award honors an outstanding individual achievement in promoting, enabling, and/or delivering improvements in the quality of health information internationally. The 2016 award is presented to Elaine Russo Martin, Lamar Soutter Library, University of Massachusetts Medical School–Shrewsbury.

The 2015/16 Board of Directors has named five association members as Fellows of MLA. Fellows are chosen for their outstanding contributions to health sciences librarianship and to the advancement of the purposes of MLA. Barbara A. Epstein, AHIP, FMLA, Health Sciences Library System, University of Pittsburg, Pittsburgh, PA, is an example of natural and progressive leadership evinced over a career spanning more than forty years. Helen-Ann Brown Epstein, AHIP, FMLA, Virtua Center for Learning, Mount Laurel, NJ, has demonstrated durable achievement by a sustained level of commitment to the goals of MLA. Donna B. Flake, AHIP, FMLA, SEAHEC Medical Library, South East Area Health Education Center, Wilmington, NC, has provided exemplary leadership to the members of MLA through many years of participation at various levels of MLA. Janice E. Kelly, FMLA, Outreach and Special Populations, National Library of Medicine, Bethesda, MD, has been a well-respected leader in medical librarianship with great passion and commitment for over thirty-five years. Michael R. Kronenfeld, AHIP, FMLA, A.T. Still Memorial Library, A.T. Still University of the Health Sciences, Mesa, AZ, has been an active and enthusiastic MLA member for almost thirty years.

From time to time, the MLA Board of Directors sees that an exceptional contribution has been made to the profession and the goals of the association. This year’s MLA President’s Award goes to a group of members in recognition of their outstanding work implementing the new way of selecting and providing programming for the 2016 annual meeting: Amy Chatfield, acting associate director, Educational and Research Services, Norris Medical Library, University of Southern California–Los Angeles; Carrie L. Iwema, AHIP, information specialist, Molecular Biology, Health Sciences Library System, University of Pittsburgh, Pittsburgh, PA; Melissa Ratajeski, AHIP, reference librarian, Health Sciences Library System, University of Pittsburgh, Pittsburgh, PA; and Brenda L. Seago, professor, Greenblatt Library, Georgia Regents University–Augusta.

The highest honor that MLA confers on any individual is the Marcia C. Noyes Award. President Kraft asked J. Michael Homan, AHIP, FMLA, recipient of the 2015 Noyes Award, to introduce this year’s recipient, Margaret Bandy, AHIP, FMLA.

President Kraft concluded the awards ceremony with the following remarks: “Each year, the awards ceremony reminds us of the outstanding accomplishments our peers have made to the profession of health sciences librarianship. It simultaneously provides the encouragement to continue striving to new levels of achievements. In recognizing these individuals, we applaud the ‘best and brightest’ in the field.”

## BUSINESS MEETING

The Business Meeting was held on Tuesday, May 17, 2016, 9:00 a.m. to 10:25 a.m. President Michelle Kraft, AHIP, welcomed everyone to the meeting and asked Executive Director Kevin Baliozian to introduce the members of the 2015/16 MLA Board of Directors: President Michelle Kraft, AHIP; President-Elect Teresa L. Knott, AHIP; Immediate Past President Linda Walton, AHIP; Treasurer Chris Shaffer, AHIP; Secretary Sandra G. Franklin, AHIP; Chapter Council Chair Angela Dixon, AHIP; Section Council Chair Jodi L. Philbrick, AHIP; and Directors Kristine M. Alpi, AHIP; Melissa De Santis, AHIP; Heidi Heilemann, AHIP; Melissa L Rethlefsen, AHIP; and Lisa K. Traditi, AHIP. Mr. Baliozian then introduced appointed officers, editors, and coordinators: Parliamentarian Patricia Thibodeau, AHIP, FMLA; Sergeant-at-Arms Linné Girouard, AHIP; *MLA News* Editor Cheryl Rowan; *Journal of the Medical Library Association (JMLA)* Editor I. Diane Cooper, AHIP; MEDLIB-L coordinator Richard James; and Oral History Project Coordinator Carolyn E. Lipscomb, AHIP, FMLA.

Mr. Baliozian then recognized the vital role the 13 MLA chapters play in bringing the benefits and services of the association to members at the regional, state, and local levels. He also recognized the 22 sections and special interest groups of MLA that provide significant networking and professional development opportunities for MLA members in their areas of specialization, allowing specific needs and issues to be addressed. He also acknowledged committees, task forces, and representatives to allied organizations for their crucial role in the success of MLA’s programs and services. He then remarked that more than 400 new members have joined MLA since the 2015 annual meeting.

President Kraft returned to the podium to offer congratulations to Dr. Patricia Flatley Brennan on her recent appointment as director of the National Library of Medicine. She also thanked Betsy L. Humphreys, FMLA, for the outstanding job she did as the interim director of the National Library of Medicine.

President Kraft then called to order the Business Meeting of the 2016 MLA annual meeting and asked if a quorum of 250 voting members required for transaction of business was present. Sergeant-at-Arms Thibodeau confirmed the quorum, and President Kraft called on Secretary Franklin to move adoption of the Rules of Assembly. Ms. Franklin explained that the Rules of Assembly include information on addressing the chair, presenting motions, debating, and voting. At the direction of the Board of Directors, she moved that the Rules of Assembly as they appear on MLANET be adopted. Voting paddles were raised, and there being a majority in the affirmative, the rules were adopted. Ms. Franklin then announced that each meeting registrant had a printed copy of the *Official Program* and that the agenda for the 2016 Business Meetings was on page 35. She moved that the agenda be adopted. The vote was affirmative, and the agenda was adopted.

President Kraft announced the ballots for MLA’s election of 2016/17 officers, members of the Board of Directors, and members of the Nominating Committee were sent electronically or by postal service to all 2,848 eligible voting members of the association. One thousand, two hundred and twenty valid ballots were returned, with a participation rate of 42.8%. The election results were certified by votenow.com, Jacksonville, Florida, MLA’s election contractor, on December 14, 2015. Election results were announced in the December 17, 2015, issue of MLA-FOCUS and in the February 2016 issue of the *MLA News.* Complete election results, including vote totals, are published in the *2015/16 Annual Report,* which is available on MLANET.

Election results: President-Elect: Barbara A. Epstein, AHIP; MLA Board of Directors (three-year term): Amy Blevins, AHIP; Keith W. Cogdill, AHIP; Nominating Committee: Helen Ann Brown Epstein, AHIP, FMLA; Kelly Gonzalez, AHIP; Steven J. Greenberg; Shannon Demona Jones, AHIP; Joey Nicholson; Rikke Ogawa, AHIP; Gerald (Jerry) Perry, AHIP; Gurpreet Rana; and Michele R. Tennant, AHIP. Linda A. Walton, AHIP, MLA’s 2015/16 immediate past president will chair the 2017/18 Nominating Committee.

President Kraft then called on Bylaws Committee Chair Ellen Brassil, AHIP, to give background information about the purpose of proposed changes to the bylaws and special rules of order, which will streamline decision making and clarify processes. Documents were posted in the online meeting program and were available in print. Secretary Franklin confirmed that amendments to the bylaws, as well meeting time and place, were published on MLANET on January 28, 2016, announced in the February issue of the *MLA News,* and announced in a post on the Full Speed Ahead blog. President Kraft then called on Parliamentarian Thibodeau to review the process for addressing motion 1 and the amendment to motion 1. To streamline the process, it was moved to temporarily suspend Robert’s Rules of Order, section twelve, to address all amendments together, thereby eliminating the rule to treat each substitution or change as a separate motion. Voting paddles were raised, and there being a majority in the affirmative, section twelve of Robert’s Rules of Order were temporarily suspended. President Kraft called on Bylaws Committee Member Mary Helms to summarize the changes to motion 1 and then read the amendment to motion 1, which was located on pages 4–10 of the handout. Voting paddles were raised, and there being a two-thirds majority in the affirmative, the motion was approved to amend motion 1 as presented.

Parliamentarian Thibodeau then announced that section twelve of Robert’s Rules of Order was no longer suspended, so debates and amendments could continue while working through the amended main motion for all further motions. President Kraft opened the floor to discussion of motion 1 as amended by the amendment to motion 1, the amended main motion. She went on to review the procedures for making motions from the floor to amend the amended main motion. There was a motion and second from the floor to not strike article 4, section 6, requiring the MLA executive director to be bonded and leave the section as is. Voting paddles were raised, and there being a majority in the negative, the motion failed.

It was moved that the board approve for presentation to the membership amendments to the bylaws that have been noted as areas 1, 2, and 3 under motion 1 and as amended by the amendment to motion 1. Voting paddles were raised, and there being a majority in the affirmative, the motion was approved.

President Kraft then went on to motion 2, elimination of the special rules of order. Bylaws Committee Chair Brassil presented motion 2, as stated on page 25 of the handout. Voting paddles were raised, and there being a majority in the affirmative, motion 2 eliminating special rules of order was approved.

President Kraft thanked everyone who worked on the bylaws changes for all of their hard work.

President Kraft then called on Treasurer Shaffer to present the treasurer’s report.

Chris Shaffer, AHIP: Hi, my name is Chris Shaffer. I’m the treasurer for the Medical Library Association. The Board of Directors approved the 2016 business plan at their meeting in November 2015. The business plan includes significant new investments in MLA education programs and reflects over $100,000 in annual savings, which will be ongoing, from the move of MLANET to the Socious platform, removing the need for MLA to maintain servers and software on a number of different platforms, including servers and software in the MLA offices.

The 2016 budget reflects revenues of $2,811,500 and expected expenses of $2,808,455, for a net revenue of $3,045. The 2015 actual preliminary unaudited results show a net revenue of $5,707. I congratulate the other members of the board and the MLA staff on meeting our budget in a time of significant transition and upheaval, which was no small task. I will note that the adjustment for disposal of fixed asset represents the write down for closing down the MLA servers and is not an actual cash loss but is instead a paper loss reflected in depreciation of those servers that we disposed of.

If you would like more information on MLA’s finances, MLA publishes its annual budget every year in the April issue of the *MLA News.* An annual audited report with detailed year-end financial statements is posted each year after June 1 for members in the MLANET file library under Strategy and Finances at www.mlanet.org/p/do/si/topic=221. And you may contact me, the MLA treasurer, or Ray Naegele, the director of financial and administrative services, with any questions.

Next, President Kraft called on Executive Director Baliozian to give the executive director’s report.

Kevin Baliozian: I remember being here a year ago. Some of you who were here last year probably remember the new guy and the slide and wondering what an executive director does. So it’s been about fifteen to sixteen months now, so one year later we all celebrated a wonderful year. This is kind of how I felt. Actually, I’ve had a wonderful time, and I just wanted to share a few things about why I love my job and some of the things that really struck me here. So, MLA members and staff are:

- Welcoming. And I really appreciate your warm welcome. Not just at this time in the meeting but in committees and chapter meetings. It’s just been wonderful and a blessing. So thank you.
- Highly engaged, knowledgeable, selfless, smart. I mean the adjectives could go on and on. This is something that really strikes me at MLA that is genuinely shared by all of you. It’s great. For an
- executive director and staff to have such engaged members is really a blessing as well.
- Self aware, introspective, no sacred cows. I mean it’s amazing when we’re saying, “Okay, we’re going to look into this or look into that,” everybody says, “Great!” You know, let’s reexamine and having people actually end up coming to me and saying, “Could we look at the way we’re doing this?” “Sure, if you feel it should be looked at, that sounds great.” So there’s this general feeling of everyone that we can actually go and reconsider how we’re doing things and see if we can do them better and it’s trickled down everywhere. And it really feels wonderful.
- Hard working. That’s no doubt; oh, my god, the task forces, the committees, the volunteers, the program committees, the juries, and so on. My goodness, the amount of work done by what is close to 500 people who volunteer directly in various roles. That’s a staggering 20% of the membership that actually is directly involved in committees and task forces. I don’t know if you realize, but this is way above the norm for most membership organizations. So, congratulations.
- Passionate. Caring, argumentative, certainly true, online or not. All welcome and wonderful. Communicative. I think that’s the key. People aren’t shy of giving their opinions and then being heard is important and certainly impacting what happens. So, continue. Bring it on. It’s absolutely wonderful.
- And fun. I think I get a sense that those of you engaged are having fun. We’re certainly having fun at the staff. Not necessarily every minute, but every day for sure. And that’s rewarding. I mean you shouldn’t be spending your volunteer time, precious volunteer time, if you’re not going to be having fun in the process. So, that’s why we come to work every day and have a good time, and I know I speak in the name of my staff who feels absolutely the same way. So thank you very much.

Board transparency is something that we’ve been striving as a board to improve. The board actually voted to publish agendas prior to the meetings so you can actually see them online with the calendars. We’ve always published highlights so that’s not new, but now that the board minutes are available to the membership in the file libraries, so you can consult the board minutes as soon as they’re approved, and ones going back, so if you want to know the details of the decisions beyond the highlights, you can do that now from this room on MLANET. So that’s one of the ways we do transparency. Full Speed Ahead, one of the most read blogs and with increasing readership, every time there’s a major topic on that, the board talks about it. We also have committee chairs, and we really are inviting committees, task forces, chapters, and SIGs to contribute to that as well as part of the engagement and full transparency effort. Board liaisons certainly have a big role in transparency and sharing with committees what the board’s doing and, of course, in listening. That’s probably their main role on committees and bringing that information back, one of the many ways the board hears what’s happening with members. And certainly chapter meetings, the MLA updates. All of you who are at chapter meetings have known about that for years. That’s a fun time also to really engage and talk about what we’re doing. So if you have any suggestions, issues, or concerns, engage, engage your board members, engage your staff, engage your liaisons. You can call us, email, speak to us, you can go on social media, lots of different ways of doing it. If you don’t tell us, we’re not mind readers, so we really appreciate that.

Transparency, so to go into the transparency, this is the pre-board highlights of what was discussed and approved just a couple days ago. We met for a day and a half. We do that twice a year, meeting face to face, and two other times, we meet virtually for ninety minutes. And each of those, I could speak for half an hour so I’m just going list them, and if you have questions engage us, we’ll be glad to talk about it. We approved minutes of prior meetings, that’s pretty obvious. We elevated a Consumer and Patient Health Information Section award to the national level. More about that, all these things will be communicated in detail. We established an Awards and Grants Endowment Review Task Force. You know we have $1.5 million in our endowment for grants, and MLA to our knowledge has never actually looked at that. Probably good every 130 years to do that.

We approved the Strategic Priorities Task Force final report and dismissed the task force with thanks. They worked on section and SIG definitions and lately on audience definitions. So that’s been a big building block to future work. We approved the establishment of an online *Journal of the Medical Library Association (JMLA)* platform. That’s of course in addition to PubMed Central and print opt-in options. There’ll be lots of communication about this. None of this goes into effect until next year, but I think you’ll be very happy about how we’re going make the *JMLA* even more accessible to members and nonmembers and just kind of make it a little more modern. So a lot more about that.

We converted the Shaping Our Future Fund to an “impact investing” model. I can’t go into details, but basically it’s making more money available to spend to support the development of educational programs for MLA as part of our goal three. So you’ll hear a lot about that as well, but we do need funds to support that so that is something that the board voted on. Of course, we reviewed all the annual reports. Believe me, board members read every line of every annual report. That is an important part of visibility that we’re doing as well.

This is the last slide. The board for the first time this year has actually done a self-assessment survey. That’s something they’re going do yearly, and that was a spirited and interesting conversation, particularly on those items where there was divergence, and that will lead to action items and an ongoing yearly exercise.

We spent an entire day reviewing every detail of the strategic goals. So there you see one line, but that’s actually six hours or more of work. Here are a few examples of decisions around those. We agreed on a general organizational framework and business model to support the expanded educational curriculum. That’s going to be the next six months of engaging memberships and committees on what the board is thinking in terms of committee structures around that. There are no decisions around those things; those will follow an extensive engagement period of obviously key committees like the Continuing Education Committee (CEC) but not just sections and SIGs that had their own discussions on where they see themselves being involved in this part. So all of that is going to be a dialogue really leading, most likely, to decisions in November for the board meeting.

We established a new MLA strategic goal on communities (sections and SIGs). Spoiler alert, Teresa is going to address this in a few minutes. Approved the concept of the MLA Research Training Institute. You probably have no idea what we’re talking about here. That’s related to goal 5 on research. That’s something for 2017, so we’ll definitely have time to communicate on that. We established the MLA Award for Research Advancement in Health Sciences Librarianship. This is all around recognizing institutions who promote the idea of research and evidence-based practice. Also part of goal 5, so that’s very exciting. We also for the first time had, at least in a long time, had an editor-in-chief update on the *JMLA* and that’s going to become a regular occurrence. Again, it’s for alignment.

So quite a busy meeting, and we’re meeting again tomorrow. There will be no bylaw changes next year, at least that we can think of. And the intention is to go back to time for Q&A, which obviously today we don’t have. But we did some of that last year, and the idea is to use those thirty minutes for Q&A. So we’ll see you next year on that. Thank you very much.

President Kraft returned to the podium and moved on to the annual report. In the interest of time, annual reports were received in a block. The informational reports of the appointed officials, the councils, committees, task forces, representatives, sections, and chapters are found in the *2015/16 Annual Report of the Medical Library Association.* These reports are available on MLANET and will remain there throughout the year. They are also available in paper copy from the executive director’s office by request. There being no corrections or objections from the members, the reports were filed as presented.

She then recognized and thanked retiring MLA Board Members Alpi, Dixon, and Franklin and presented them with certificates as a token of respect and gratitude for work well done. President Kraft also expressed her gratitude to Linda Walton, AHIP, MLA president during the 2014/15 association year. Highlighting some of Walton’s initiatives, President Kraft presented a plaque to Ms. Walton for a job well done.

President Kraft then introduced the new members of the MLA Board of Directors: Barbara Epstein, president-elect; Amy Blevins, director; Keith Cogdill, AHIP, director; and Melissa Ratejeski, Chapter Council liaison.

Teresa Knott then presented outgoing President Kraft with the Presidential Cup and congratulated her for a year when, under her leadership, MLA broadened its opportunities to build its future.

President Kraft next introduced 2016/17 MLA President Teresa Knott who delivered her inaugural address.

## INAUGURAL ADDRESS

Teresa L. Knott, AHIP: One of the first things that you probably need to know about me is that I love a good metaphor. I like storytelling, so as I worked on this presentation, I went through a couple of things. I thought about change. It’s one of my favorite topics. It’s something that’s become constant in our lives. But then, maybe not. I thought about musical theatre. Anybody who knows me knows that I love American musical theatre. I was amused when Barbara Epstein told me the other day that she felt like she was my understudy. I thought about mosaics, because of the theme of this year’s meeting. And I thought this was a really pretty one.

I also considered weaving. In 1998, when we celebrated the centennial of MLA, I was chair of the Membership Committee, and we presented an award recognizing the longest membership in MLA. I’m happy to report that that longest-serving member is still a member of MLA, Lucretia W. McClure, AHIP, FMLA. And to present this award, I got to put words into the president’s mouth. My words were about MLA being a tapestry, I liked the longitudinal threads of how things function across time and distance and then weaving to create a pattern.

But in the end, I decided that I really wanted to talk about building value. Over the last year, the Board of Directors, the staff, the membership have spent a lot of time talking about building value. When we make decisions on the part of the board, we look at it and say, “How does this build value for the organization, for the members, for the component parts?” So that seemed like a fitting focus for this talk.

One of the first things you have to consider about value is who your audiences are. Because different audiences value different things. I might be really excited about one thing, Heidi might be excited about something else. One of the things that informed us this year is the work of the Strategic Priorities Task Force led by Katherine R. Stemmer Frumento, AHIP. What happened is the Strategic Priorities Task Force went through and created a list of who we consider to be our primary audiences. We conceded that we have other audiences, but these are the ones that are core to MLA. If you have other ideas about it please let me know. I’ll be happy to listen to you. Our core audiences are MLA members or people who are health sciences librarians we’d like to be members. How do we reach out to students? Our donors, our vendors, international librarians, library science educators.

As part of the exercise of what we did, we considered what did we do for each audience. In looking at that, we essentially said:

- How do we provide information to a group?
- How do we advocate to or from them? We might have someone advocating on somebody’s behalf toward us.
- How do we provide career services?
- How do we educate or mentor?
- How do we attract them to annual meetings?
- How do we entice people to contribute to MLA publications?
- How do we get financial support from them?

If you’ll think back on the audiences, clearly some of those components or dimensions apply to one group but not to others. So you’ll hear more about that later.

To me, education is a core value of our organization, and it’s one of the major reasons that the organization exists. For us to educate each other, for us to learn from each other through attending presentations, by reading what’s written in the *JMLA,* by writing for the *JMLA,* by doing research, and by interacting with each other through networks. Michelle mentioned the Task Force to Review MLA’s Competencies for Lifelong Learning and Professional Success. I have a hard time, that’s such a long name. Paula Raimondo and her group did an amazing job with this. As Kevin mentioned, over the next six months, we are really going to continue our focus on this existing goal by building an organizational structure to implement our new educational plans.

The plans that we are looking at are step by step. We are almost done with the revised MLA competencies. The group has gone through and said, “What are things that MLA members need to know?” How are they, how do they fit in a framework, what are the tiers that people need to know? Going forward, provide a foundation of how we develop a framework. We will begin to plan how we will update our educational offerings within this framework. As part of this, we are also near the end of purchasing a new learning management system (LMS). We’re close to the end of doing that. We have worked with our partners, the National Library of Medicine and the National Network of Libraries of Medicine, and many members to look at that and make recommendations about the LMS, which should happen in the next couple of months.

Going forward, part of the goal is to develop new content that fills in blanks, building toward courses that support the professional competencies that are new. Part of this will also be developing and implementing a marketing plan where we decide how we offer courses to MLA members. Much of it will be online through the LMS, some will be in person. We are also looking at how we can offer content beyond the boundaries of MLA to people who might be interested and to grow that as a portion of what we do. But it’s important that we go back to that we’re building value for members by improving and expanding our educational offerings.

So as we move on with this, there are some guiding principles that we have all agreed to. The first is that it has to be a student-centric experience. It can’t be, oh well, the instructor wants to teach this. Which, yes, is wonderful, but is there a need for that? It’s about building an organized learning curriculum where, as I said with professional competencies, you step through things. If you take this class, then you know that the natural progression is to take the next course and the next course, although you can jump around. It is really to develop an organized framework for people to move through the curriculum instead of “ah well, there was a lot of interest in this so we’re going teach this class.” Also by taking courses online, there are more possibilities to step through a curriculum more readily than there are currently. Again, the professional competencies are the foundation and the framework that we will build this new system on.

One of the most important parts is to be responsible with our resources. With that, probably the most important resource is the people involved in the resource, making sure that students have what they need, that instructors have the resources that they need to create and deliver content, that staff resources are used appropriately, that member resources are used appropriately as well. Overall, we definitely want to make sure we deliver a high degree of excellence in our work. Kevin mentioned briefly that one of the decisions the Board of Directors made on Friday was that we would use the Shaping the Future Fund, which is actually a quasi-endowment, not an endowment, to develop impact funding.

With impact funding—this is a simplified version—with impact funding, we might pay an instructor to develop a course within the framework of the curriculum. We would have agreements with them in terms of sharing revenues that were generated. The instructor would go teach the class, and hopefully, revenue would accumulate. That revenue, when it exceeded the basic investment, would continue to go back into the Shaping the Future Fund. So you create a cycle of investing, and building, and improving through the use of the fund.

The other goal that we’ve discussed over the past year is looking at an international strategy. There has been a lot of assessment, discussion, investigation, and conversations. And I’m sharing with you that the goals that were proposed:

- strengthening our presence in worldwide impact;
- developing and delivering educational programs;
- building an international network;
- understanding and exploring whether to collaboratively define the different roles and standards for health information governance around the world;
- advocating for the adoption by industry of country models for access, such as HINARI, kinds of things that empower information professionals;
- enabling MLA members to play a role in their global communities, facilitating learning in the worldwide community;
- promoting awareness of international issues; and
- enhancing the quality of health care, education, and research throughout the world.

And I’d like to point out that, in fact, that last bullet point is actually a part of the MLA mission statement.

As we’ve had these conversations, it has become clear is that with developing and delivering educational programs in underserved countries and developed economies, and building on MLA’s international network of health information practitioners and their professional organizations, particularly on those two points, the International Cooperation Section and Librarians without Borders® are doing a great deal of work. The Librarians without Borders does training in information retrieval and library information assistance to underserved countries across the world. They work in partnership with the World Health Organization, and we recently, through the generosity of Elsevier, created the MLA HINARI/Research4Life Grant that supports training activities that promote the use of scientific research resources in emerging and low income countries. We also have various bilateral agreements with other country library associations.

In the end, the board decided that there is actually work being done actively in these areas and that, at this time, creating an international goal does not rise to the level of a board goal. As we’ve looked at this and discussed building value across the association, one of the things that we’ve really focused on is that a board-level goal is something that should require board attention and be something that needs to change. While the international goal is important, it is something that is actually being addressed through the International Cooperation Section, Librarians without Borders, and the Public Health/Health Administration Section, with their interest in global health. This is not to say that in the future that it won’t rise to a board-level goal, but at this point, we feel like it is being managed at the membership level.

So, I would say that the greatest value of our association is our communities, the people in them, how we form them, how we participate with them. This was the goal that was proposed and approved, which is to strengthen the member communities, particularly sections and SIGs. We had a long discussion about whether chapters would be involved. As chapters are independent entities, they may opt in to doing something like this, but it is not something that MLA can dictate to them.

We’ll be looking at analyzing the communities, the architecture, and the roles, looking at member leadership development, content, education, and marketing. The reasons that we’re doing this is:

- to increase the relevance, effectiveness, and value of MLA sections and SIGs to members;
- to empower MLA members at the grassroots level;
- to better support our programs;
- to maximize the value and visibility of member content;
- to reduce administrative complexity; and
- to attract members.

So, this is the whole goal. It was developed very strongly in concert with Section Council; Jodi L. Philbrick, AHIP, and I had many conversations. Section Council will own this goal, so to speak. In terms of community architecture, this is really looking at the scope of sections and SIGs, the work that they do, defining what their roles are as we evolve forward. We are looking at the sections and SIGs also as a place where member leadership development happens. With content and education strategies, some of you may have read my *MLA News* article in April, where I challenged the sections to think about themselves as subject content experts, subject matter experts (SMEs). We are really asking the sections to consider what they can bring to our educational structure as we move forward. Marketing is to make sure that we’re developing strategies that attract members to our sections. For SIGs, we want to have conversations about whether SIG membership has to require MLA membership, or if we can find ways to open that to the broader public.

As a part of moving forward, I will be appointing a task force that will be responsible for this strategic goal. Kevin has taught us very well that somebody has to own it or parts of it. With the Section Council chair, we’re working on developing a committee who will move forward on this. We expect that work to last two to three years. That’s another important thing that you should know about the MLA Board goals. They’re not something that can be done in a year, but they tend to be two to three years in framework. We adopted this as a board last Friday.

For MLA to continue to succeed, it’s my opinion that we need to make sure that our decisions are based on adding value to the members and to our partners we work with, our communities we serve. We build value in the relationships and the networks that we form. We build value through education and programming. And I would say that our members are our greatest asset. I look forward to working with you to keep building MLA’s future value. Thank you.

At the conclusion of her inaugural address, President Knott asked Deborah Sibley and Donna R. Berryman, AHIP, of the 2017 National Program Committee to come to the podium to give the official thank you for the 2015 annual meeting.

Deborah Sibley: Hi everybody. So Donna, we’ve had a great visit to Toronto and learned a lot about the area from our little friend Mosey Mosaic. He told me a couple of raccoon jokes that I wanted to share with everyone, but in the interest of time, we’ll just pass over them. It’s so sad. If you’d like to hear them at another time, just ask either one of us whenever you see us. Okay so, we’ll get to the resolution.

This year’s meeting has been a celebration of diversity and the mosaic in which we live. *Whereas* the 2016 Joint Planning Committee (JPC) has created the framework of a wonderful mosaic of inspiring speakers and new programming formats that enable members of the Medical Library Association (MLA), the Canadian Health Libraries Association/ Association des bibliothèques de la santé du Canada (CHLA/ABSC), and more recently, the International Clinical Librarians Conference (ICLC) to come together to learn in the relaxing and fun environment of Toronto, a multicultural city of 5.5 million people and 200 spoken languages.

Donna Berryman, AHIP: *And whereas* the Local Assistance Committee has cleared the streets of varmints; welcomed us enthusiastically; provided venues for parties, food, and learning experiences as well as everything we needed to know about Toronto and its 10,000 restaurants, 100 public libraries, and 1,600 parks; and

*Whereas* the MLA headquarters staff has planned and run another wonderful meeting without ever letting us see the hard work, thought, and effort that goes into creating this learning and sharing experience.

Deborah Sibley: *And whereas,* the exhibits and exhibitors have provided many updates, fun events, and educational opportunities, both in the exhibit hall and outside of it.

Therefore, be it resolved that the membership of each organization extends profound appreciation and gratitude to the 2016 JPC, exhibitors, sponsors, MLA staff, and presenters for an excellent meeting in the wonderful city of Toronto.

Next, Catherine M. Burroughs and Tania Bardyn, AHIP, of the 2017 Local Assistance Committee invited members to the 2017 annual meeting in Seattle.

At the conclusion of the invitation, President Knott recognized Sandra Franklin, 2015/16 secretary of the MLA Board of Directors, who moved to adjourn the meeting. The motion carried, and the business meeting of the 116th annual meeting was officially adjourned.

## SECTION PROGRAMMING

Section programs were presented in 5 time slots: Sunday, May 15, 3:00 p.m.–4:25 p.m.; Monday, May 16, 10:30 a.m.–11:55 a.m. and 1:00 p.m.–2:25 p.m.; Tuesday, May 17, 3:00 p.m.–4:25 p.m.; and Wednesday, May 18, 9:00 a.m.–10:25 a.m. Paper abstracts that were scheduled to be presented are available on the MLA ’16 website. The final version of the abstracts reflecting only those presented at the meeting is included as a supplemental file to the January 2017 issue of the *Journal of the Medical Library Association.*

## POSTER SESSIONS

Poster sessions were presented in three time slots: Sunday, May 15, 2:00 p.m.–2:55 p.m.; Monday, May 16, 2:30 p.m.–3:25 p.m.; and Tuesday, May 17, 2:00 p.m.–2:55 p.m. Section and chapter posters were presented on Tuesday, May 17, 2:00 p.m.–2:55 p.m. Poster abstracts that were scheduled to be presented are available on the MLA ’16 meeting website. The final version of the abstracts reflecting only those posters presented at the meeting is included as a supplemental file to the January 2017 issue of the *Journal of the Medical Library Association.* The actual posters are available online in the MLA ’16 meeting website.

## OTHER MEETINGS AND EVENTS

### Pre-meeting activities

On Thursday, May 12, the MLA Board of Directors met. On Friday, May 13, the MLA Board of Directors and Credentialing Committee met and the following informal meetings were held: National Network of Libraries of Medicine (NN/LM) Steering Committee and NN/LM Regional Medical Libraries Staff. On Saturday, May 14, the following groups met: CHLA/ABSC Board; chairs, presidents, and allied representatives orientation (committees, sections, and chapters); 2017 National Program Committee; 2017 program planners; Chapter Council; Joint Section Council/Chapter Council; Section Council; and Nominating Committee.

### Sunday, May 15, 2016

On Sunday, May 15, these groups, sections, and SIGs met: Ad Hoc Committee to Review Core Clinical Journals; African American Medical Librarians Alliance SIG; chapter treasurers orientation; Collection Development Section Executive Board Meeting; Consumer and Patient Health Information Section Executive Board Meeting; Fellows of MLA; Gaming in Adult Learning SIG; Health Association Libraries Section; Hospital Libraries Section Executive Board Meeting; International Cooperation Section; Interprofessional Education SIG; *JMLA* Editorial Board; Latino SIG; Pharmacy and Drug Information Section; Research Award judges; Systematic Reviews SIG PRISMA-Search Consensus Group; and Translational Sciences Collaboration SIG. Exhibitor meetings included: Creating a Partnership Between Your Library and the Tech Transfer Department: A Case Study Using Drug Development Tools; Enhancing the Value of the Medical Librarian in Today’s Changing Clinical Decision Support World; and Increasing the Global Visibility of Research: New Tools for Health Care Libraries.

### Monday, May 16, 2016

On Monday, May 16, the following groups, sections, and SIGs met: 2018 National Program Committee; Awards Committee; Books Panel; Cancer Librarians Section; Chiropractic Libraries SIG Business Meeting; Clinical Librarians and Evidence-Based Health Care SIG; Collection Development Section Business Meeting; Complementary and Alternative Medicine SIG; Consumer and Patient Health Information Section Business Meeting; Dental Section; Federal Libraries Section; Governmental Relations Committee; Hospital Libraries Section Business Meeting and Ice Cream Social; Institutional Animal Care and Use SIG; Lesbian, Gay, Bisexual, and Transgendered Health Science Librarians SIG; Medical Library Education Section; Molecular Biology and Genomics SIG; New York-New Jersey Chapter Board; Nursing and Allied Health Resources Section Business Meeting and Executive Board Meeting; Osteopathic Libraries SIG; Professional Recruitment and Retention Committee; Public Health/Health Administration Section; Resource Sharing SIG; Revitalizing the Research Imperative Task Force; Rising Stars Committee; Scholarly Communications Committee; Southern Chapter Executive Committee; Veterinary Medical Libraries Section Business Meeting and Informal Meeting; and Vision Sciences SIG.

### Tuesday, May 17, 2016

On Tuesday, May 17, the following groups, sections, and SIGs met: Bylaws Committee; Educational Media and Technologies Section; History of the Health Sciences Section; Information Literacy in Medical Education SIG; Joseph Leiter NLM/MLA Lectureship Committee; Leadership and Management Section; Librarians without Borders® Committee; Libraries in Curriculum SIG; Medical Informatics Section; Membership Committee; MLA community managers and webmasters; Outreach and Marketing SIG; Public Services Section; Research Section; section treasurers orientation; Systematic Reviews SIG Business Meeting; Task Force to Review MLA’s Competencies for Lifelong Learning and Professional Success; and Technical Services Section.

### Wednesday, May 18, 2016

On Wednesday, May 18, the following groups, sections, and SIGs met: Continuing Education Committee; Grants and Scholarships Committee; Oral History Committee; and Strategic Priorities Task Force.

## OPEN FORUMS

Three open forums were held concurrently on Sunday, May 15, from 4:30 p.m.–5:25 p.m.:

- Discussion of Proposed Bylaws Amendments
- MLA’s International Initiatives
- Public Access to Government-Funded Research Publications

## NATIONAL LIBRARY OF MEDICINE UPDATE

Betsy L. Humphreys, FMLA, acting director, National Library of Medicine (NLM), began the NLM Update, which took place on Tuesday, May 17, from 11:00 a.m.–11:55 a.m. Joyce E. B. Backus, associate director for library operations, began the session with a look back at the last five years of the Regional Medical Library Program, as well as a look ahead to the 2016–2021 cooperative agreements. She then gave a behind-the-scenes look at MEDLINE indexing.

Stacey J. Arnesen, chief, Disaster Information Management Research Center, then gave a report of how NLM responds to public health emergencies, including providing access to literature and resources, developing response tools, and training librarians and other disaster information specialists.

Ms. Humphreys wrapped up the session with information on the new NLM director, Patricia Flatley Brennan, beginning in August 2016, as well as the new director for the National Institutes of Health (NIH) Precision Medicine Cohort Program, Eric Dishman. The NIH Precision Medicine Cohort Program intends to recruit, consent, and actively engage more than one million volunteers in the establishment of a longitudinal research database of electronic health record data, bio specimens, genomic data, participant reported data, and environmental sensor data. Ms. Humphreys continued with personnel updates, noting that David Lipman, director, National Center for Biotechnology Information, and associate director, Biomedical Information Resources, will be staying at NLM. She then announced NLM personnel departures.

Ms. Humphreys then announced that the new NLM director will initiate a new strategic planning process within the framework of the NLM Working Group Report. She went on to discuss NIH’s data sharing and data management policies and priorities, ClinicalTrials.gov and the rulemaking process for US clinical trials registration and results submission, and progress on public access to publications in PubMed Central. Ms. Humphreys concluded the NLM Update with a Q&A session.

## LEGISLATIVE UPDATE

The Legislative Update was held on Tuesday, May 17, from 1:00 p.m.–1:55 p.m., and was moderated by Linda Hasman, chair, MLA Governmental Relations Committee. Committee members provided an overview of health funding, public access, and other relevant issues.

## OTHER SPECIAL EVENTS AND RECEPTIONS

### Saturday, May 14, 2016

- Welcome Reception and Opening of the Hall of Exhibits, 5:30 p.m.–7:30 p.m.

### Sunday, May 15, 2016

- MLA New Members/First-Time Attendees Program, 7:00 a.m.–8:55 a.m.
- Sunrise Yoga, 7:30 a.m.–8:30 a.m.
- Chapter Council Presents Chapter Sharing Roundtables Luncheon, noon–1:55 p.m.
- International Visitors Reception, 6:00 p.m.–7:00 p.m.
- Library School Reunion, 6:00 p.m.–7:00 p.m.

### Monday, May 16, 2016

- Academy of Health Information Professionals Q&A Session, 2:30 p.m.–3:25 p.m.

### Tuesday, May 17, 2016

- MLA Book Authors and Prospective Authors Gathering, 2:00 p.m.–2:55 p.m.
- Presidents’ Awards Dinner, 6:30 p.m.–10:00 p.m.
- After Party, 10:00 p.m.–2:00 a.m.

## SUNRISE SEMINARS

Exhibitors held Sunrise Seminars to provide information and to introduce new products and services. The following seminars were held.

### Sunday, May 15, 2016

- Transforming the Way You Search, Discover, and Deliver, OvidDiscovery
- The Argument for Innovation: Powering Transformation by Putting Ideas to Work, McGraw-Hill Education.
- DynaMed Plus

### Monday, May 16, 2016

- American Psychological Association (APA)
- EBSCO: Solving the “Findability” Conundrum in Medical Research
- Inside/Outside Look at Cochrane: Update from Carol Lefebvre
- New VisualDx, New Way of Diagnostic Decision Making

### Tuesday, May 17, 2016

- Embase: Innovation in Systematic Review Searching

## TECHNOLOGY SHOWCASES

Twelve Technology Showcases were held throughout Sunday, Monday, and Tuesday:

- Best Practice in Authentication: Atlantic Health and OpenAthens
- The R2 Digital Library: A Health Sciences eBook Database
- PlumX
- Serials Management and e-Packages
- Nursing Reference Center Plus
- Opening Ovid: Helping Librarians Boost Patron Engagement with Clinical Resources
- Overview of Distiller Literature Review Software
- Medical Full-Text Databases
- Revolutionary 3D Human Anatomy Solutions
- Covidence: Streamlining Systematic Review Production
- CABI’s Global Health and CAB Abstracts Database
- The Evolving Role of the Library in Institutional and Faculty Assessment: A Discussion of Research Metrics

## CONTINUING EDUCATION COURSES

The 2015/16 Continuing Education Committee offered the following courses to 327 attendees on May 13 and 14, 2016.

### Friday, May 13, 2016

CE100, Oral History Workshop: Conducting, Collecting, and Archiving, **Instructor:** David J. Caruso, director, Center for Oral History, Chemical Heritage Foundation, Philadelphia, PA

CE201, Leading the Way: Preparing for and Enhancing Your Leadership Role, **Instructors:** Lindsay Alcock, head, Public Services, Health Sciences Library, Memorial University of Newfoundland–St. John’s, Canada; Ken Carriveau, director, Delivery Services, Baylor University Libraries, Waco, TX; Robyn Reed, head, Access Services, Schaffer Library, Union College, Schenectady, NY; Kelly Thormodson, assistant director, Harley E. French Library of the Health Sciences, School of Medicine and Health Sciences, University of North Dakota–Grand Forks; and Martin Wood, AHIP, director, Maguire Medical Library, Florida State University College of Medicine–Tallahassee

CE300, Advanced Searching Techniques and Advanced Strategy Design, **Instructors:** Julie Glanville, MCLIP, project director, York Health Economics Consortium, University of York, York, United Kingdom; and Carol Lefebvre, HonFCLIP, independent information consultant, Lefebvre Associates, Oxford, United Kingdom

CE301, Health Literacy 360, **Instructors:** Andrea Szwajcer, AHIP, clinical librarian, University of Manitoba Libraries, St. Boniface Hospital, Winnipeg, MB, Canada; and Lara Killian, AHIP, librarian educator, Patient Education, Library, Nova Scotia Hospital–Dartmouth, Canada

CE400, Grey Horizons: Grey Literature Searching for New and Emerging Health Technologies, **Instructors:** Monika Mierzwinski-Urban, information specialist, Canadian Agency for Drugs and Technologies in Health, Ottawa, ON, Canada; Kelly Farrah, AHIP, information specialist, Canadian Agency for Drugs and Technologies in Health, Ottawa, ON, Canada; and Sarah Bonato, reference/research librarian, Centre for Addition and Mental Health (CAMH), Toronto, ON, Canada

CE500, Expand Your Library Instruction Toolkit: Introduction to Online and Distance Learning Support, **Instructors:** Virginia Pannabecker, health, life science, and scholarly communication librarian, University Libraries, Virginia Tech–Blacksburg; and Margaret Hoogland, AHIP, distance support librarian, A.T. Still Memorial Library, A.T. Still University of the Health Sciences, Kirksville, MO

CE501, APPsense Makes the Patrons Grow Fonder: Mobile Resources in the Health Sciences, **Instructors:** Maureen (Molly) Knapp, AHIP, research support and education librarian III, Rudolph Matas Library of the Health Sciences, Tulane University, New Orleans, LA; and Elizabeth C. Whipple, AHIP, research librarian, Ruth Lilly Medical Library, Indiana University School of Medicine–Indianapolis

CE701, Research by Design: Proposing, Planning, and Carrying out a Research Project for the Practicing Librarian, **Instructor:** Lorie Kloda, AHIP, assessment librarian, McLennan Library, McGill University, Montreal, QC, Canada

CE702, Data Sharing and Reuse: Roles for Health Sciences Librarians, **Instructor:** Lisa Federer, research data informationist, NIH Library, National Institutes of Health, Bethesda, MD

### Saturday, May 14, 2016

CE302, Becoming an Expert Searcher, **Instructor:** Terry Ann Jankowski, AHIP, FMLA, assistant director, User Experience, Health Sciences Library, University of Washington–Seattle

CE303, Searching ClinicalTrials.gov, the World Health Organization Portal (ICTRP), and Regulatory Agency Websites to Identify Clinical Trials for Systematic Review and Other Clinical and Research Questions, **Instructors:** Julie Glanville, MCLIP, project director, York Health Economics Consortium, University of York, York, United Kingdom; and Carol Lefebvre, HonFCLIP, independent information consultant, Lefebvre Associates, Oxford, United Kingdom

CE304, Genes in the Library: Concepts and Resources in Genetics and Bioinformatics, **Instructor:** Helene R. McMurray, head, Bioinformatics Consulting and Education Service, Edward G. Miner Library, and assistant professor, Biomedical Genetics, University of Rochester Medical Center, Rochester, NY

CE305, Librarians without Borders®: HINARI and Internet-Based Information Resources for Health Professionals in Low and Emerging Income Countries, **Instructors:** Lenny Rhine, FMLA, coordinator, E-Library Training Initiative, Librarians without Borders, MLA; and Karin Saric, librarian, Norris Medical Library, University of Southern California–Los Angeles

CE401, Perspectives in Research Data Management, **Instructors:** Alisa Surkis, translational science librarian; and Kevin Read, National Library of Medicine associate fellow, Ehrman Medical Library, New York University School of Medicine–New York

CE402, Fundamentals of Data Visualization, **Instructor:** Jacqueline Wirz, research data specialist, Library, and director, Professional Development and Graduate Student Affairs, School of Medicine, Oregon Health & Science University–Portland

CE403, Beyond Readability Formulas: Writing Clear and Effective Patient Education and Communication Materials, **Instructor:** Ruti Volk, AHIP, patient education librarian, University of Michigan Health System–Ann Arbor

CE600, Librarian and Active Learning Models: Team-Based Learning (TBL), Problem-Based Learning (PBL), and Case-Based Learning, **Instructors:** Susan Cavanaugh, evidence-based medicine librarian; and Sharon Whitfield, emerging technologies librarian, Cooper Medical School, Rowan University, Camden, NJ

CE601, Library Instructional Design, **Instructors:** Mary Edwards, AHIP, reference and liaison librarian and assistant university librarian, Health Science Center Libraries; and Judith Murphy Roberts, instruction consultant and training program coordinator, George A. Smathers Libraries, University of Florida–Gainesville

CE703, Evidence-Based Medicine (EBM)/Evidence-Based Practice (EBP): The Basics: Study Design and Randomized Controlled Trials, **Instructor:** Connie Schardt, AHIP, FMLA, adjunct faculty, School of Library and Information Science, University of North Carolina–Chapel Hill

CE704, ABCs of Research Impact: Altmetrics, Bibliometrics, and Citations, **Instructor:** Robin Featherstone, information specialist, Alberta Research Centre for Health Evidence (ARCHE), Department of Pediatrics, Edmonton Clinic Health Academy, University of Alberta–Edmonton

CE705, Supporting Systematic Reviews: The Basics, **Instructors:** Janene Batten, nursing librarian, Harvey Cushing/John Hay Whitney Medical Library, Yale University, New Haven, CT; and Angela Myatt, curriculum liaison librarian, Briscoe Library, University of Texas Health Science Center–San Antonio

### Saturday, May 14, 2017, 2-hour course options

CE306, Serving Mental Health Clients in Your Library, **Instructor:** Brooke Ballantyne Scott, librarian, Library Services, Royal Columbian Hospital, New Westminster, BC, Canada

CE404, Literature Surveillance Apps: Tools for the Next Generation of Table of Contents Notification Services, **Instructor:** Maureen (Molly) Knapp, AHIP, research support and education librarian III, Rudolph Matas Library of the Health Sciences, Tulane University, New Orleans, LA

CE706, Improve Your Data! How to Use Surveys Effectively in Health Care Library and Information Science (LIS) Research, Evaluation, and Audit, **Instructor:** Hannah Spring, senior lecturer, Research and Evidence Based Practice, Faculty of Health and Life Sciences, York St. John University, York, United Kingdom

## RESOURCES AND SERVICES

Sponsored by PEPID Medical Information Resources, the Hospitality Center was staffed by the Local Assistance Committee and provided maps and information about local Toronto attractions. Volunteer bloggers, the Local Assistance Committee, and the 2016 Joint Planning Committee contributed to the official meeting blog with tips, announcements, and more. Live streaming conversations were available on Twitter using hashtag #mlanet16, and Elsevier ClinicalKey sponsored a MOSAIC ’16 mobile app. Complimentary WiFi was available throughout the convention center courtesy of The JAMA Network. For those seeking new jobs and prospective employers, the Job Placement Center was open from Saturday through Tuesday. Sponsored by McGraw-Hill Medical, the Relaxation Station offered attendees a seated chair massage to ease their meeting stress. The MLA Connections Booth offered attendees the opportunity to donate to the MLA grants and scholarships program. The Hall of Exhibits was open Saturday through Tuesday and included a booth for MLA ’17 in Seattle.

## SUPPLEMENTAL FILES

Appendix MLA ’16 Poster Abstracts

Appendix MLA ’16 Program Session Abstracts

