# Correction

**DOI:** 10.5195/jmla.2017.202

**Published:** 2017-04

## VOLUME 105

**105(1) January, page E1**

Mitchell N. 116th Annual Meeting, Medical Library Association, Inc., Toronto, ON, Canada, May 15-20, 2015. J Med Libr Assoc. 2017 Jan;105(1):E1-E20. DOI: http://dx.doi.org/10.5195/jmla.2017.123.

An incorrect date is given in the title. The title should be: 116th Annual Meeting, Medical Library Association, Inc., Toronto, ON, Canada, May 13-18, 2016

