# Supplementary material for: 116th Annual Meeting, Medical Library Association, Inc., Toronto, ON, Canada, May 13–18, 2016
Source: J Med Libr Assoc. 2017 Jan;105(1):E1–E20. doi: 10.5195/jmla.2017.123 (PMC5234456; doi:10.5195/jmla.2017.123)
Supplement: Appendix [file mosaic16_poster_presentations.pdf]

Medical Library Association
Mosaic ’16 Poster Abstracts

Abstracts for the poster sessions are reviewed by members of the Medical Library Association Joint Planning Committee (JPC), and designated JPC members make the final selection of posters to be presented at the annual meeting.

Poster Number: 1

Time: Sunday, May 15, 2016, 2:00 PM – 2:55 PM

Painting the Bigger Picture: A Health Sciences Library's Participation in the University Library's Strategic Planning Process

Adele Dobry, Life Sciences Librarian, University of California, Davis, Davis, CA; **Vessela Ensberg**, Data Curation Analyst, Louise M. Darling Biomedical Libary, Louise M. Darling Biomedical Library, Los Angeles, CA; **Bethany Myers, AHIP**, Research Informationist, Louise M. Darling Biomedical Library, Louise M. Darling Biomedical Library, Los Angeles, CA; **Rikke S. Ogawa, AHIP**, Team Leader for Research, Instruction, and Collection Services, Louise M. Darling Biomedical Libary, Louise M. Darling Biomedical Library, Los Angeles, CA; **Bredny Rodriguez**, Health & Life Sciences Informationist, Louise M. Darling Biomedical Library, Louise M. Darling Biomedical Library, Los Angeles, CA

Objectives: To facilitate health sciences participation in developing a strategic plan for the university library that aligns with the university's core mission and directs the library's focus over the next five years.

Methods: The accelerated strategic planning process was planned for summer 2015, to be completed by fall 2015. The process was facilitated by bright spot, a consulting group. Seven initial areas of focus for the library were determined: Library Value and Visibility, Teaching and Learning, Research Process, Information and Resource Access, Relationships Within the Library, and Space Effectiveness. Each area of focus was assigned to a working group of 6-8 library staff members. The working groups were responsible for obtaining staff input in order to draft each section of the strategic plan. Five members of UCLA Library's Research, Instruction, and Collection Services department participated in five different working groups in order to provide a local unit perspective to the planning process.

Results: The resulting strategic plan will be described at the MLA annual meeting.

Conclusions: This approach to strategic planning enabled a completed plan to be delivered on time, incorporating input from the whole library.

Poster Number: 4

Time: Sunday, May 15, 2016, 2:00 PM – 2:55 PM

Less Shelves, More What? How Are Academic Health Sciences Libraries Choosing to Replace Their Shelves?

Alanna Campbell, Health Sciences Librarian, Health Sciences Library/Public Services, Sudbury, ON, Canada

Objectives
In the past decade academic health science library collections have become predominantly digital in response to advancing technology and user demand. This transition in how libraries collect materials has had a significant impact on library spaces. Many libraries have begun sweeping discontinuation of their bookshelves in response. What are they replacing their shelves with? The objective of this study will be to research what academic health sciences libraries are doing with their previously shelf consumed spaces including what impact this has had on services, utilization of space by users and post-renovation user and staff feedback. While there are many case studies on library space renovations and project management there is a gap in information on academic health sciences libraries in general and existing trends.

Methods
The structured questionnaire was open to all libraries belonging to the Association of Academic Health Sciences Libraries (AAHSL) who have removed at least one bookshelf in the last five years. Resulting qualitative data was assessed using a framework analysis while quantitative data was examined using SPSS.

Results
Of the 162 AAHSL members who were sent the survey there were 14 qualifying response. While responding libraries had removed between five to "thousands" of bookshelves all had kept some shelving. Collections stored on the removed shelves were primarily discarded or transferred to storage. The majority of libraries gained group study and individual study space. The amount of additional seating ranged from 23-340 seats. A minority of libraries lost some space or all space. Only a small fraction of libraries introduced new services such as 3-D printing and free scanning of journal articles. Most libraries experienced an increase in visitor statistics since their shelf removal and received positive feedback from library users and library staff.

Conclusion
In conclusion while the response rate was low some interesting trends in how libraries approach removing bookshelves and the outcomes of these projects were identified. One in particular is that the majority of libraries are prioritizing study space and collaborative spaces in their space modifications.

Poster Number: 7

Time: Sunday, May 15, 2016, 2:00 PM – 2:55 PM

PubMed "Early Alerts": A Pilot Study to Support Prospective Detection of Emerging Adverse Drug Events

Alfred Sorbello, Medical Officer, CDER/Food and Drug Administration/DHHS, Office of Translational Sciences/CDER/Food and Drug Administration/DHHS, Silver Spring, MD; **Anna M. Ripple**, Information Research Specialist, U.S. National Library of Medicine/NIH/DHHS, Cognitive Sciences Branch/Lister Hill National Center for Biomedical Communication/National Library of Medicine/NIH/DHHS, Bethesda, MD; **Olivier Bodenreider**, Senior Scientist, U.S. National Library of Medicine/NIH/DHHS, Cognitive Sciences Branch/Lister Hill National Center for Biomedical Communication/National Library of Medicine/NIH/DHHS, Bethesda, MD

Objectives: The FDA conducts post-market drug safety surveillance by analyzing reports submitted to the FDA Adverse Event Reporting System (FAERS). To enhance prospective detection of emerging adverse drug events (ADE), we leveraged existing PubMed “MyNCBI” functionalities to survey the biomedical literature for the latest published safety information in the use case of the new oral hepatitis C drugs.

Methods: Using PubMed “MyNCBI” cubby functionality, we established a search strategy to retrieve citations most recently added to PubMed and provide automated weekly emails (PubMed ‘Early Alerts’) with abstracts and links to available full text citations. The search strategy includes three criteria: (1) Drugs of interest; (2) Citation scope: MEDLINE journal citations, as soon as they are entered in PubMed (prior to indexing); (3) Time Period: week of interest.

Results: A typical weekly PubMed 'Early Alerts' email delivery includes abstracts with links for 12-15 recently published citations from MEDLINE journals spanning 5-8 oral hepatitis C drugs. Our search strategy is designed for recall. Its precision can be increased significantly by requiring specific words in the titles or abstracts (“safety”, “toxicity”, “adverse”, and “tolerability”).

Conclusions: By leveraging existing PubMed “MyNCBI” functionalities, we can canvass a broad range of MEDLINE journals for the latest drug safety data. This approach lessens reliance on time-consuming, inefficient ad hoc searches and complements traditional approaches to finding relevant safety information. FDA Medical Officers and Safety Evaluators are evaluating our strategy by manually reviewing the ‘Early Alerts’ for new information that may portend an emerging ADE.

Poster Number: 10

Time: Sunday, May 15, 2016, 2:00 PM – 2:55 PM

Supporting Curriculum Development in Undergraduate Medical Education: Linking the Association of College and Research Libraries Framework for Information Literacy to Local Practice

Amy C. Studer, Health & Life Science Librarian, Blaisdell Medical Library, UC Davis, Sacramento, CA; **Beth Callahan**, Head, Research Services, UC Davis, University Library / Research Services, Davis, CA

Objectives

To build a foundational model to support curriculum development in undergraduate medical education at the University of California, Davis, by mapping the new ACRL Framework for Information Literacy for Higher Education (hereafter referred to as "ACRL Framework") to graduation competencies, learning objectives, and milestones. This map will provide a platform for communicating the value of librarians as partners in instruction with medical school faculty, curriculum committees, and other stakeholders.

Methods

The authors reviewed local medical school graduation competencies, learning objectives, and milestones; the ACRL
Framework
; and, relevant published literature. Key stakeholders were identified and interviewed, including medical students and faculty. The ACRL
Framework
was mapped to the medical school curriculum milestones to provide a guide for when and how information literacy could be incorporated into the curriculum. Once completed, the resulting map, along with information gleaned from the interviews, will be utilized to develop relevant education sessions, in partnership with local stakeholders.

Results

Through the mapping process, the authors found that many of the information literacy frames, knowledge practices, and dispositions in the ACRL
Framework
mapped readily to the local competencies. In particular, they were highly relevant to the evidence-based medicine (EBM) competencies in the current medical school curriculum. However, the ACRL

Framework
provided additional depth to the competencies, potentially offering an enriched perspective to learning objectives and milestones. In some cases, the ACRL Framework
identified higher level learning objectives, based on Bloom's taxonomy, than the local milestones specify. The ACRL
Framework
also enabled gaps to be identified in the local learning objectives and milestones.

Interviews with stakeholders identified new opportunities for librarian involvement in information literacy instruction related to EBM and inclusion of some of the ACRL Framework's
information literacy frames, knowledge practices, and dispositions.

Conclusions

The authors found the ACRL
Framework
to be relevant to undergraduate medical education, especially when mapped to local EBM-related graduation competencies, learning objectives, and milestones. The resulting map could serve to guide conversations with stakeholders and support revisions of the current curriculum. Opportunities were identified for increased library engagement in instruction.

Poster Number: 13

Time: Sunday, May 15, 2016, 2:00 PM – 2:55 PM

Developing an Instruction Series for Summer Research Students in Veterinary Medicine

Andrea C. Kepsel, AHIP, Health Sciences Educational Technology Librarian, MSU Libraries, East Lansing, MI

Objectives:

To increase information literacy offerings within the Michigan State University (MSU) College of Veterinary Medicine, the veterinary medicine librarian took what was traditionally a one-shot orientation session and expanded it into a three-part instructional series tailored to the needs of visiting summer research students. The active learning activities and assessment strategies used will be discussed.

Methods:

In the summer of 2015 the MSU College of Veterinary Medicine hosted 30 undergraduate and DVM students from institutions across the country as part of their grant-funded summer research program. The veterinary medicine librarian developed an instruction series designed to enforce information literacy skills early in the participants' research careers that included an orientation session, a hands-on searching workshop, and a citation management session. A variety of active-learning activities were employed to reinforce the concepts being taught and provide the students with hands-on experience using library resources. Different assessment strategies were tailored to each of the instruction sessions, offering valuable feedback to both the librarian and program coordinator. Anticipated outcomes of this instruction series include increased librarian integration into the veterinary medicine programs, and development of information literacy skills in students early in their research careers.

Results:

Expanding the instructional offerings to the summer research students increased the veterinary medical librarian's interactions with both the students and the program coordinator, helping to strengthen her liaison role within the college. By seeing the students at multiple points throughout the program the librarian was able to introduce concepts when the students needed them most and build upon knowledge gained in the previous sessions.

Assessments of each session identified points where the students struggled and provided the librarian an opportunity to address their questions at subsequent sessions. The instructional series was determined to be a success and, with some

minor changes based on feedback from the participants, will hopefully continue for future summer research programs at the MSU College of Veterinary Medicine.

Conclusions:

Multiple instruction sessions throughout the summer provided an opportunity to reinforce ideas and concepts over time and relate them to where the participants were in their research process. The veterinary medicine librarian was able to interact more with the participants than previous one-shot sessions allowed and built valuable relationships within the MSU College of Veterinary Medicine.

Poster Number: 16

Time: Sunday, May 15, 2016, 2:00 PM – 2:55 PM

Staff Wellness Counts Too: The Care and Feeding of the "Feel Good 15" Blog

Ann Glusker, AHIP, Reference and Consumer Health Librarian, Business/Science/Technology Dept., The Seattle Public Library, Seattle, WA

Objectives: To find avenues for all staff to connect with a wellness activity without feeling shamed or daunted; to assess staff interest in a message that isn't explicitly related to work activity; specifically to create a blog which provides interesting and fun ideas for spending a 15 minute break in ways that lead to wellness.

Methods: The Workplace Environment Committee approved a trial run of the "Feel Good 15" blog. The blog is written by the consumer health librarian, with input on topics from staff. Each article gives suggestions for activities during a 15 minute period, such as stretching, dream interpretation, eating carrots, etc. The tone is warm, light, slightly ironic and humorous, and each post features further reading suggestions and a dramatic picture. The blog features six categories of posts: food, exercise, body health, mental health, habit formation, and miscellaneous. It is posted at the beginning of each pay period. The blog was initially promoted through the intranet and by encouraging managers to mention it to staff. Staff were encouraged to set up alerts to the blog, and also there is an announcement on the intranet message board each time a post is added.

Results: So far, the readership has been good; out of a staff of about 675 persons, the Feel Good 15 blog has gotten between 144 and 539 hits per month, and to date has 48 staff members who have set up an alert. Most posts have at least one comment, and some quite a few more. Many staff members have expressed appreciation for having something fun to read as part of their work life, whether or not they engage in the suggested activity.

Conclusions: Creating a welcoming and humorous blog to encourage staff wellness is simple and straightforward to implement, and in this case was very well-received. More importantly, staff report that the blog's suggestions have led to their pursuing health-related activities in the workplace.

Poster Number: 19

Time: Sunday, May 15, 2016, 2:00 PM – 2:55 PM

Customizing the iPad to Support a Clinical Fellowship in Radiology: A Qualitative Study

Antonio P. DeRosa, AHIP, Research Informationist II, Memorial Sloan Kettering Cancer Center Library, New York, NY; **Delia M. Keating**, Associate Attending, Memorial Sloan Kettering Cancer Center, Radiology Department, New York, NY

Objectives: To determine the value of supplying Fellows with iPad mini tablets pre-loaded with productivity apps, pertinent eBooks, diagnostic tools, health information resource pathways, and current awareness resources; and to test whether or not a tablet with selected resources chosen by the informationist will enhance Fellows experience during a one-year clinical rotation in a Radiology service.

Methods: Pre-, mid-, and post-clinical rotation survey questionnaires will be administered via SurveyMonkey online and will be qualitatively analyzed in this pilot program. Fellows have an orientation where they are introduced to the iPad as well as the various clinical, productivity, and research tools provided, where the initial survey will take place. Another survey will be administered during the exit interview after the fellows' rotation. Results will then be analyzed by both the Embedded Librarian and the Attending Radiologist who oversees the education program for rotating fellows and residents in the service.

Results: Comparing the before and after survey results will help to determine future perspectives on iPad lending and resources provided, as well as shed light on the value of information/technology services provided by the informationist in the clinical setting. The results will also help to inform the addition or removal of certain resources to the iPad tablets as well as help to better understand the productivity and workflow needs of the fellows.

Conclusions: Changes and improvements to what is made available on the iPad tablet will take place as a result of the study. Also, the test group of Fellows for this pilot study will have a say in the information provided on the iPad tablets in the future. Soliciting feedback before and after the clinical rotation should help to forge new partnerships between the informationist and the future Fellows.

Poster Number: 22

Time: Sunday, May 15, 2016, 2:00 PM – 2:55 PM

Creation of Complementary and Alternative Medicine (CAM) Search Filters for the Cochrane Complementary Medicine Specialized Register of Trials

Becky Skidmore, Information Specialist, Skidmore Research & Information Consulting, Gloucester, ON, Canada; **Tamara Rader**, Patient Engagement Officer, Canadian Agency for Drugs and Technologies in Health, CADTH, Ottawa, ON, Canada; **Raymond S. Daniel**, Information Technician, Ottawa Hospital Research Institute, Knowledge Synthesis Group, Smiths Falls, ON, Canada; **Alain Mayhew**, Senior Research Associate, Ottawa Hospital Research Institute, Knowledge Synthesis Group, Ottawa, ON, Canada; **L. Susan Wieland**, Assistant Professor, University of Maryland School of Medicine, Center for Integrative Medicine, Providence, RI

Objectives: To identify all complementary and alternative medicine (CAM)-related trials in Cochrane CENTRAL and other relevant databases considered to be high-yield sources of CAM trials (e.g., Allied and Complementary Medicine (AMED), Index to Chiropractic Literature (ICL), PsycINFO) for inclusion in the Cochrane Complementary Medicine register of trials. Eligible trials will receive the specialized register code SR-COMPMED for incorporation into CENTRAL.

Methods: After checking the range of filters available through the ISSG search filter resource, https://sites.google.com/a/york.ac.uk/issg-search-filters-resource/home, PubMed's CAM subset http://www.nlm.nih.gov/bsd/pubmed_subsets/comp_med_strategy.html was selected as the most comprehensive filter available. Information regarding its development and maintenance was sought via Cochrane's Information Retrieval Methods Group (IRMG) and MLA's Expert Searching listservs. Two information specialists (IS) analyzed the filter for accuracy, appropriateness, comprehensiveness, and precision. The strategy was refined and vocabulary for new CAM therapies was developed and added. To overcome problems of precision when searching PubMed (e.g., lack of proximity operators), a line-by-line version of the strategy was translated to OVID MEDLINE. The OVID version was used as a basis for translating to other databases and platforms. The Cochrane Highly Sensitive Search Strategy for randomized controlled trials (RCTs) was adapted and applied to each database.

Results: Our review of the current PubMed CAM filter revealed spelling errors, omissions, redundancies, inconsistencies, and difficult or questionable logic. We reviewed the underlying concepts, updated the MESH, verified journal names and developed the MEDLINE OVID strategy. Adjustment was required to correct spelling errors, word forms, redundancies, and field labels. Proximity searching was adjusted

to increase specificity. We incorporated new vocabulary for emerging CAM concepts (e.g., environmental interventions such as therapeutic gardens, exposure to nature, etc.). In particular we analyzed zero-retrieval lines for errors. Once finalized, the new MEDLINE strategy was systematically translated to other databases and quality checked for accuracy.

Conclusions: We have systematically created base CAM filters for use in multiple databases. In doing so, we have reliably identified additional trials for the Cochrane Complementary Medicine register of trials. Testing the sensitivity and specificity of these filters is the next logical step.

Poster Number: 25

Time: Sunday, May 15, 2016, 2:00 PM – 2:55 PM

Social Media and Lesbian, Gay, Bisexual, Transgender (LGBT) Health: A Primer for Health Librarians

Brooke Ballantyne Scott, Librarian, Royal Columbian Hospital, Fraser Health, Royal Columbian Hospital Library, New Westminster, BC, Canada; **Blake Hawkins**, Graduate Student, University of British Columbia, Master's of Library and Information Studies, Vancouver, BC, Canada

Objectives: Since the early 2000s, there has been a limited amount of scholarship by librarians concerning the LGBTQ population, their health, and their engagement or practices with health information and services. The purpose of this paper is to synthesize findings that demonstrate the complexities concerning LGBTQ health and information interactions in the context of advancements in social media.

Methods: The research question guiding our project is "how does social media connect members of the LGBTQ community to healthcare?". We completed a scoping review using MEDLINE , Embase, CINAHL, and PsycINFO. We limited to publications from 1990 – 2015, and to English language. We found 93 articles across these databases, which was reduced to 77 after de-duplication, and further reduced to 28 after culling for relevance to our research question. In 2000 and 2001, two articles were written regarding why health librarians should care that LGBTQ health could differ from that of other populations. Since the early 2000s, there has been limited scholarship concerning LGBTQ people and health librarianship. It is our aim to fill this 15-year gap and argue that health librarians, who have a significant role in social media and healthcare, should also be aware of these interactions.

Results: The literature demonstrated that there have been advances over time concerning LGBTQ people and how social media is used to connect them to healthcare information and services. Prior to the early 2000s, the methods used were more on the microscale of information engagement. From the mid-2000s onward, however, the usage of social media has allowed for interactions on the macroscale between the LGBTQ community and healthcare. Currently, the usage of social media towards LGBTQ users is more prevalent in the USA. Other findings include the recruitment and training of peer educators (using established models and guidelines) for health outreach on social media platforms, strong partnerships between the LGBTQ community and universities and public health organizations, and the importance of understanding and respecting both online and LGBTQ culture in health interactions. Populations studied are mostly MSM (men who have sex with men), and therefore research hasn't reached other important subsets of the LGBTQ community in regards to social media connecting them to healthcare.

Conclusions:
There are a variety of ways that social media is being used for LGBTQ health, and themes from the literature that should become more known amongst health librarians.

Poster Number: 27

Time: Sunday, May 15, 2016, 2:00 PM – 2:55 PM

Chocolate Ambassador: Outreach to Internal Medicine Residency Program in a Community Hospital

Bonita Archer, Liaison Librarian, Werner Medical Library / Rochester General Hospital / Rochester Regional Health, Rochester, NY

Objectives: Werner Medical Library identified the opportunity to grow librarian outreach to the RGH Internal Medicine Residency Program. At outset, IM Residents spent time using library computers but requests for librarian help or literature searches were low. With a designated Internal Medicine Residency librarian, the goal was to increase our visibility to residents, and lay the foundation for future partnerships and collaborative work with their department. The first step was to find our part in the whole, and for this we went outside of the library.

Methods: The newly appointed librarian and library director met with program directors and administrators to establish relationships and to identify contacts and areas for the librarian to participate. As a result, the librarian was granted access to the program’s online forum and added to the distribution list for weekly teaching schedule and rosters for incoming residents. Residency coordinators suggested office hours for residents, and the librarian started afternoon IM Lounge hours 1-3 times a week in the IM Residents' Lounge. Essentials brought were business cards, literature search forms and chocolates. With administrative approval, the librarian attended residency noon conferences to gain knowledge of program content. Hospital-wide Grand Rounds offered another arena to learn and meet people. Presentations or rounds where librarian provided literature search support were given priority.

Poster Number: 35

Time: Sunday, May 15, 2016, 2:00 PM – 2:55 PM

Coming to a Library Near You: How Librarians Brought a National Library of Medicine Traveling Exhibit to Their Institutions

Carolyn Martin, AHIP, Consumer Health Outreach Coordinator, University of Washington, NN/LM Pacific Northwest Region, Seattle, WA; **Christopher Bishop**, Health Sciences Librarian, Marian University, Mother Theresa Hackelmeier Memorial Library, Indianapolis, IN; **Caryl Wyatt**, Reference/Instructional Librarian, Ivy Tech Community College, Lawrence Campus Library, Indianapolis, IN

Objectives: Three libraries in Indianapolis provide a unique example of collaboration by hosting a National Library of Medicine traveling exhibit at three locations within the city. Hosting the exhibit, “Harry Potter’s World,” will provide greater awareness of the libraries and their host institutions through a fun educational atmosphere and bring attention to National Library of Medicine resources through Harry Potter stories.

Methods:
The National Library of Medicine traveling exhibits are an overlooked resource that can benefit clinicians, researchers, students, educators and the general public. The poster showcases a unique collaboration between a consortium of libraries in central Indiana as a way to encourage all kinds of libraries to consider hosting an exhibit to engage their communities in health and medicine. A community college librarian requested funding from the Central Indiana Health Sciences Library Consortium (CIHSLC) for the National Library of Medicine traveling exhibit, “Harry Potter’s World: Renaissance Science, Magic, and Medicine” to come to her library and other libraries of CIHSLC. The community college librarian contacted NLM and reported back that NLM was agreeable to this unique request where three various organizations in one city collaborated to host an exhibit over three booking periods.

Results:

Each of the three libraries hosted and promoted the exhibit through various marketing tools, such as events, flyers, and handouts. Students and faculty expressed their appreciation for the exhibit, and faculty members reached out to librarians on ways to incorporate the exhibit into assignments for health science students.

Conclusions:

The collaboration of three libraries to bring a NLM travelling exhibit to Indianapolis was

a success. Utilizing the resources of both the Consortium and the National Library of Medicine benefitted not only the libraries, but also the students and faculty who were impacted by experiencing the exhibit and made aware of the resources available to them through their library and the NLM.

Poster Number: 36

Time: Sunday, May 15, 2016, 2:00 PM – 2:55 PM

InfoBundles: Using Free, Simple Web Tools to Dramatically Enhance Online Information Retrieval

Carrie L. Iwema, AHIP, Information Specialist in Molecular Biology, Health Sciences Library System, University of Pittsburgh, Pittsburgh, PA; **Angela Zack**, Web & Application Programmer, University of Pittsburgh, Health Sciences Library System, Pittsburgh, PA; **Ansuman Chattopadhyay**, Head of Molecular Biology Information Service, Health Sciences Library System, Health Sciences Library System, Pittsburgh, PA

Objectives: To present library-developed web-based tools connecting digital text to online databases, allowing readers to immediately gather relevant information while reading scientific articles. Collections of these “InfoBoosters” are grouped into bundles accessing complementary information, including “LiteratureSearch”, “MolecularDBs”, “ConsumerDBs”, and “ClinicalDBs”. These tools assist readers with revealing information not directly described in the text and potentially foster the creation of new hypotheses.

Methods: As life sciences research becomes more interdisciplinary, the papers we read increasingly include biological and clinical concepts outside of our expertise. Information is readily available in various databases and websites, but they are scattered through the internet and challenging to locate. Our academic health sciences library developed “InfoBoosters”- an easy to install web browser widget connecting digital text to databases and retrieving information on-demand. For example, upon selecting the title of an article and clicking the “LiteratureSearch” bundle in your browser bar, a pop-up window appears displaying links to AltMetric, GoogleSchlrByTitle, AuthorProfile-ORCID, PubMed, and more. Selection of AltMetric brings up a small window with article-level metrics. Selection of GoogleSchlrByTitle goes to Google Scholar and provides the number of times the paper has been cited. InfoBooster Bundles are convenient tools to help with knowledge discovery, streamlined reading, and even manuscript preparation.

Poster Number: 39

Time: Sunday, May 15, 2016, 2:00 PM – 2:55 PM

Partnering with Young Adults to Design Youth-Friendly Web Pages on Mental Health Topics

Cathryn Chiesa, Resource Librarian, National Child Traumatic Stress Network, National Child Traumatic Stress Network, Durham, NC; Nathalie Reid, AHIP, Manager, Information and Resources & Manager, CE Program, National Child Traumatic Stress Network, National Child Traumatic Stress Network, Los Angeles, CA

Objectives: This paper/poster examines the process and importance of working side by side with young adults to include their unique perspective when developing youth friendly webpages on a website where none existed. Youth need to be informed partners with professionals in the treatment and recovery from traumatic experiences so their voice needs to be included in webpage development.

Methods: The XXXX Network's website is an information hub for professionals who provide care to children, teens, and young adults experiencing traumatic stress. The website, originally created for practitioners, provided little information for young people. This project grew out of the need to add youth friendly resources on mental health topics to the existing XXXX.org website. Authors will describe how with input from the XXXX Youth Task Force they created youth friendly webpages. Partnering with the Task Force fostered the development of pages with layout, design, activities, and language appealing to youth. Challenges of creating youth friendly pages on a website designed for professionals and the benefits of collaborating with young adults will be described. Findings will encourage others to develop partnership with youth so their perspective is addressed when developing resources designed for adolescents, teens, and young adults.

Conclusions

Development of the youth webpages is proceeding more slowly than anticipated. Factors affecting the process include youth taskforce only meets once a month via phone, NCCTS staff time restraints and other projects, staff needing time to become familiar with using Storyboard to write scripts and with using Articulate. Although the project is taking longer than anticipated staff and the taskforce are excited about the new pages. We hope to have 3 or 4 topics available by the end of 2016 and to complete the project by the end of 2017. We have learned much about the development process and look forward to sharing our knowledge in the poster.

Poster Number: 40

Time: Sunday, May 15, 2016, 2:00 PM – 2:55 PM

Meeting a Mosaic of Research Needs: Constructing a Resource Guide for Qualitative Researchers

Chana Kraus-Friedberg, Graduate Research Assistant, Health Sciences Library, UNC-Chapel Hill, Durham, NC; Barbara Rochen Renner, Library Services Evaluation Specialist and Liaison, Allied Health Sciences,Adjunct Professor, Department of Allied Health Sciences, School of Medicine, Health Sciences Library, Health Sciences Library, Chapel HIll, NC

Objectives: In response to requests, staff at an academic health sciences library assessed the need for a new online resource for users and librarians. Qualitative research resource needs identified include compilation of local resources and services; resources to understand, evaluate, retrieve, write, and publish qualitative research; resources about integrating qualitative research into systematic reviews; and resources for emerging areas such as implementation science.

Methods: Following increasing requests from users and liaison librarians, the authors located and surveyed existing online resources and guides for qualitative research to determine the need for a new resource and potential gaps that needed to be filled. The authors combed existing resources for useful links, spoke to local and national experts, searched websites of relevant campus centers and programs, and identified appropriate literature via database and library catalog searches. Assessment of the guide will include review of drafts by external experts in qualitative research from multiple disciplines and follow-up with librarians and researchers who have recently requested information on qualitative methods. Additional information can be compiled on user and librarian requests for assistance and guide usage statistics. Coverage is expected to grow based on the evolution of qualitative research methodology and its application to new subject areas.

Results: The guide was made live in October 2015, and initial responses from users and librarians have been positive. The authors have received feedback from both on campus and off campus users, including librarians and educators who are working to support student researchers in the health sciences. External experts in qualitative inquiry have also reviewed the guide and suggested additional sources.

Conclusions: The authors continue to receive feedback from users and to refine and expand the guide in response to it. In particular, they are investigating the prospect of adding resources on the use of qualitative research in implementation studies and Community Based Participatory Research (CBPR).

Poster Number: 42

Time: Sunday, May 15, 2016, 2:00 PM – 2:55 PM

Noble Learning Resource Center Reboot

Christine A. Willis, Director of Knowledge Management & Learning Resources, Noble Learning Resource Center, Shepherd Center, Atlanta, GA

Objectives: The NLRC received an Express Hospital Library Promotion Award in 2015 from the NN/LM SE/A to increase awareness of the resources for patients, families, and staff regarding research and information related to the catastrophic injuries treated at Shepherd Center. Through promotion of the NLRC with staff the goal is to generate more recommendations to use the resources for medical research.

Methods: In order to measure the impact of outreach interventions, door count and usage statistics that have been recorded for several years will be compared to statistics after June 1, 2015. Outreach efforts include coffee breaks, Trustworthy Resource classes, and presentations in staff meetings. In addition to daily statistics, a survey was sent to the spinal cord injury peer supporters, nurse educators, case managers, and recreation therapy staff to assess knowledge of current resources prior to any outreach efforts. After six months a follow-up survey will be sent to the same group to assess the effect of the outreach efforts on their use and referrals to the NLRC. A third measure is in place at the reference desk using a tablet computer with a Google Drive survey asking patients and families about their visit to the resource center.

Results

Unfortunately there is incomplete data from 2013 – 2014, therefore statistics from 2011-2012 were used as a benchmark. Door count statistics dropped significantly due in part to a change in fax machine and popular fiction book availability, 2011 = 4153, 2012 = 3628, and 2015 = 2234. However, despite the change in these non-research services there was a 55.6% increase in the number of therapy visits in 2012 and 2015,and ready reference questions increased by 50 questions from 201 2 and 2015. Survey results from the staff did show a statistically significant difference in use of the library resources during the six month timeframe. The reference desk survey did not have a large response (n=5) but the responses were positive in that patrons found the information they sought.

Conclusions: Based on observation of those who are visiting the NLRC it has become much more focused on research and as a learning space. More patrons have been coming in to read, check out books, and meet colleagues. The coffee breaks are the most successful draw for staff, but the Trustworthy Resources

classes during six month had only one person attend. There are areas for improvement in letting patients and families know about the available resources.

Poster Number: 44

Time: Sunday, May 15, 2016, 2:00 PM – 2:55 PM

Helping to Fix Mistakes: Librarian Involvement in a Nursing Mistakes and Misjudgments (M&M) Conference

Christopher P. Duffy, Director, Library Services and Continuing Education, Medical Library, Robert Wood Johnson University Hospital, Somerville, NJ; Kathleen E. Zavotsky, Assistant Vice President, Robert Wood Johnson University Hospital, The Center for Professional Development, Innovation and Research, New Brunswick, NJ

Objectives: To describe the successful collaboration between a Medical Librarian and the The Center for Professional Development, Innovation and Research Nursing Department at an Academic Teaching Hospital to bring Librarian services to a monthly Nursing Mistakes & Misjudgments (M&M) conference.
To identify the benefits that having a librarian at the monthly Nursing M&M conference has had in supplying nurses with evidence-based information when discussing mistakes or misjudgments that occurred when treating for patients. This poster will also address the impact that having a librarian at this conference has had on overall nurse satisfaction, library promotion, and overall quality of the M&M conference.

Methods: In 2014, The Medical Librarian at Robert Wood Johnson University Hospital partnered with the Assistant Vice President of The Center for Professional Development, Innovation and Research to collaborate at a monthly nursing conference where nurses discuss mistakes or misjudgments that occurred when caring for their patients. At this conference, called "Nursing M&M", four cases that ended up having poor outcomes resulting from nursing care are presented each month by staff nurses who treated for those patients.

Every month, the medical librarian attends the M&M meeting at two campuses within the Robert Wood Johnson Health System. The Medical Librarian brings his laptop with him to each conference. As the cases are discussed, the librarian is able to conduct searches to help facilitate the spread of evidence-based best practice literature to the conference attendees.

Results: Library support at a Nursing M&M conference has resulted in the dissemination of evidence-based literature to support improvements in nursing practice at an Academic Teaching Hospital. Staff nurses have been able to use literature that the library has provided in the Nursing M&M conference to create protocols to improve practice in a variety of areas, including perioperative

pressure ulcer prevention.

Conclusions: Having a librarian present to provide real-time literature searching during a Nursing M&M conference has resulted in quick an timely dissemination of evidence-based literature to help fix mistakes in misjudgments in nursing care.

Poster Number: 46

Time: Sunday, May 15, 2016, 2:00 PM – 2:55 PM

Dogs on Call in the Library

Dana Ladd, Community Health Education Center Librarian, VCU Libraries Community Health Education Center, VCU Libraries Community Health Education Center, Lanexa, VA

Objectives: In addition to the attendant complications of dealing with illness, medical tests, and procedures, many patients experience uncertainty, stress, and anxiety. Studies show positive medical effects of therapy dog interaction with patients including reducing fear, anxiety, and depression. The Community Health Education Center, a library for patients and their family members located in an academic medical center wanted to provide a stress reduction program for patient and family library visitors using therapy dogs.

Methods: The library collaborated with Dogs on Call, a hospital based therapy dog program, to provide a stress reduction program for library visitors. The therapy dog program consists of dedicated, trained, and certified therapy dogs and their handlers who provide in-hospital visitation. The director of the Center for Human Animal Interaction provided presentations on stress reduction techniques and on the positive benefits of dog interaction for stress reduction during the holidays. The dog teams interacted with attendees following the program giving the attendees a chance to visit and interact with the dogs. Additionally, three therapy dog teams provide regular weekly visits to the library throughout the year to interact with library visitors and staff. The librarian provides the dogs with their favorite dog treats as a thank you incentive for visiting.

Results: The library held three stress reduction programs with the therapy dog teams with a total of 68 attendees. Of those filling out evaluation forms, nine people rated the program a four and thirty-one rated the program a five on a scale of 1 (worst) to 5 (best). One attendee wrote that the therapy dog program was a “very good stress reducer. Excellent!” Another attendee wrote, "[The speaker] is awesome. This is a great program." Three dog teams have continued to visit the library regularly over the past four years interacting with patients and staff.

Conclusions: Bringing the dogs into the library is beneficial to not only patients and their family but library staff as well. The program presentations and visitations have been overwhelmingly successful based upon written evaluation and verbal feedback. The library will continue to collaborate with Dogs on Call to

provide this unique stress reduction program for patients and staff in the library.

Poster Number: 48

Time: Sunday, May 15, 2016, 2:00 PM – 2:55 PM

Librarian Embedded as Faculty in a Year-Long Nursing Research Internship

Daniel Trout, Nursing Liaison Librarian, University of Rochester Medical Center, Edward G. Miner Library, University of Rochester Medical Center, Rochester, NY; ; ;

Objectives: We wanted to investigate if having an increased focus on library resources as well as having a librarian as registered faculty member in the internship helped nurses in their quest to enhance their careers. We want to show that having a librarian was an asset to the interns and other faculty members from a qualitative and quantitative standpoint.

Methods:
The last 3 cohorts of Research Interns were electronically surveyed (years, 2013,2014,2015). Most questions were closed ended (n=23) while the last 3 questions were open ended.

Results:

Most interns replied with an 87% response rate (14/17). Consistent with Magnet-designated medical centers, most nurses had a bachelor's degree (79%) Since graduation from the internship, 9/14 (64%) of the nurses conducted their own literature search and 12/14 (86) utilized library resources. These resources include utilizing books, journal articles, visiting the library, using PubMed for clinical information and using point of care tools to help with patient care. All alumni research interns found the library valuable in helping nurses. When asked what they liked about the Research Internship responses included: "Librarian involvement and education on lit searches".

Conclusions:
Having a librarian as a faculty member in the Nursing Research Internship provides valuable support to the Research Interns as well as educating nurses about the resources that the library offers in support of medical research in a large academic medical center.

Poster Number: 52

Time: Sunday, May 15, 2016, 2:00 PM – 2:55 PM

Creating a Dedicated Consultation Suite for Collaboration

Debra Werner, Librarian for Science Instruction and Outreach, John Crerar Science Library, John Crerar Library, Chicago, IL

Objectives: Consultations are an essential service in health sciences libraries. As medical research moves toward team-based and collaborative work, consultations often include more than one researcher and a librarian's office is often inadequate to accommodate groups. This presentation describes the creation of a dedicated Consultation Suite in a health sciences library at an urban academic health center.

Methods: A vacant office in a health sciences library presented the opportunity to refit it as a dedicated Consultation Suite, with integrated technology. Private funding was secured to begin work on the infrastructure of the Consultation Suite and an NN/LM Technology Improvement Award was later attained to purchase the technology for the room, including: dual HD screens, an HDMI switch box, a Macbook Air laptop, and assorted converter and adapter cables.

Results: Creating a Consultation Suite for collaborative work was, itself, a collaboration. Several library departments were involved and coordination was required to keep the project moving forward and meet award deadlines. Outside consultants were needed to provide expertise on equipment and technology beyond the institution's standard set-ups. Installation charges for the new technology and delivery costs for the furniture were larger than expected. To realize cost savings, our institution's own services were used when possible. In the end, a Consultation Suite was created with wall-mounted dual displays and technology-integrated furniture to easily project researchers' laptops and mobile devices on the displays.

Conclusions: Creating a dedicated Consultation Suite can enhance librarian-researcher collaboration. The ease with which individuals can display their laptop or mobile device screens promotes seamless information sharing. Individuals have easy access to the keyboards, and the close proximity to the screens facilitates communication. It allows the librarian to interact with physicians, nurses, medical students, and biomedical researchers in ways such as pointing to the screen, enabling hands-on learning, etc. The right-sized space furnished with equipment that enhances interactive teaching is an asset to a library's consultation services. Receiving NN/LM technology funding increases an institution's capacity to provide technology-enabled services.

Poster Number: 54

Time: Sunday, May 15, 2016, 2:00 PM – 2:55 PM

Diabetes Education: A Strategic, System-Wide Initiative

Diane G. Schwartz, FMLA, Director of Libraries, Kaleida Health Libraries, Kaleida Health, Buffalo, NY; Keith C. Mages, Clinical Informationist, Kaleida Health, Kaleida Health Libraries, Buffalo, NY

Objectives: To demonstrate that planning and collaboration among an interdisciplinary team of healthcare professionals, including librarians, can motivate the design, creation and implementation of a system-wide educational portfolio for diabetic patients. Using the Plan-Do-Check-Act model, the project will build on the success of three separate patient education programs, to achieve best educational practices and improve clinical outcomes, e.g., HbA1c below 7.

Methods: The venue is in an inner-city teaching hospital whose patients are primarily African-Americans and Latinos. Three separate undertakings exist; a librarian embedded in the heart failure clinic using tablet computers to teach diabetics with heart disease how to access, and evaluate quality internet information; a primary care physician and certified diabetes educator (CDE) using Learning About Diabetes (http://www.learningaboutdiabetes.org) to provide patients with simply written diabetes-care information, and; a nurse and a CDE providing monthly group instruction on meal planning, preparation & exercise. Patients cannot easily take advantage of each undertaking. Creating and distributing a system-wide educational portfolio (low reading-level, in English and Spanish) to encapsulate the messages of each separate educational practice is this project's major focus. Statistician developed measures will promote understanding of the new educational portfolio, each separate educational program's effectiveness, and overall patient outcomes.

Poster Number: 56

Time: Sunday, May 15, 2016, 2:00 PM – 2:55 PM

The Shifting Kaleidoscopic View Resulting from a Mandated Change in Hospital Affiliations

Dixie A. Jones, AHIP, Library Director, Health Sciences Library, LSU Health Shreveport, Shreveport, LA; Barbara Reilly, Interlibrary Loan Librarian, LSU Health Shreveport, Health Sciences Library, Shreveport, LA

Objectives: The state mandate for the university to stop operating its own hospital set off a cascade of events, beginning with finding a private partner to manage the hospital. The Library's objective was to meet the ensuing challenges which were like moving pieces forming a shifting kaleidoscopic view rather than a still mosaic where one could see the big picture.

Methods: Affiliation changes are common these days. In this case, the separation of hospital and school was complex because all functions were intertwined. The new partnership formed after separation was originally considered a win-win situation. However, the relationship soon became contentious. While trying to keep an eye on the big picture and hold pieces in place, the Library performed disparate tasks to normalize operations: notifying vendors of changes; maintaining contacts with hospital employees who had new email addresses in a directory now hidden behind their firewall; reporting hospital usage of Library computer labs to charge for what was formerly free; coping with reduced operating and personnel budgets because of loss of financial support from the hospital; communicating with two different IT departments who were at loggerheads; and forming relationships with new hospital IT personnel to communicate about electronic health record resources.

Results

Like a kaleidoscope, with each "turn" of events, the picture has changed. During the transition, morale was low and a number of long-time employees, uncertain about their futures, either retired or fled elsewhere. Possible new legal battles loom between the hospital's operating partner and the school. However, the Library operating budget has been restored for the time being, The school and hospital IT departments are settling in to their new, separate roles and are cooperating with each other and with the Library. A couple of resources have been contextually integrated into the electronic health record by hospital IT. The school's revenues have declined but the school continues to support the Library through this lean period.

Conclusions

The state legislature has come to the financial rescue of the school a few times

to make up for loss of income from the hospital, but will not continue to do so. New revenue streams must be found. While some internal pieces are continuing to shift a bit, the big picture is beginning to come back together. Secure funding for the school would supply a missing piece to fill a gaping hole in the big picture. Meanwhile, the Library is settling into the new normal.

Poster Number: 59

Time: Sunday, May 15, 2016, 2:00 PM – 2:55 PM

Continuing Altmetric Analysis

Edith Starbuck, Associate Information Services Librarian, Donald C. Harrison Health Sciences Library, Health Sciences Library, Cincinnati, OH; Charles Kishman, Associate information Services Librarian, Donald C. Harrison Health Sciences Library, Health Sciences Library, Cincinnati, OH; Sharon Purtee, Senior Librarian, University of Cincinnati, Health Sciences Library, Cincinnati, OH; Don P. Jason, III., Clinical Informationist, Health Sciences Library , Donald C. Harrison Health Sciences Library / University of Cincinnati Libraries, Cincinnati, OH; Kristen Burgess, Assistant Director for Research and Informatics , Health Sciences Library, University of Cincinnati, Cincinnati, OH; Tiffany Grant, Research Informationist, Health Sciences Library, University of Cincinnati, Health Sciences Library, Cincinnati, OH

Objective:
The Health Sciences Library is interested in the continued assessment of a 2013-2014 analysis of the extent of altmetric scores for articles published by selected departments in the College of Medicine (COM) compared with traditional metrics. The intent of this continuing analysis is to determine whether altmetric scores are an indicator of initial publication importance as well as future impact.
Methods:
The analysis was conducted on the highest altmetric-scored articles (n=40) from the initial sample of 4,210 articles and 3,678 unique publications of 228 tenure-track COM faculty from 2009-2013. Publications were analyzed according to Scopus Times Cited and Altmetrics.com. Additional factors analyzed were field(s) of study and MEDLINE's core clinical journal subset.
Results:
The highest scored altmetric articles (n=40) from the initial 2009-2013 sample were analyzed. Thirty percent of the altmetric scores changed 12% or less between 2013 and 2015. In all three years, 40% of the highest altmetric scored articles focused on stroke, obesity, and diabetes; in 2015, 23% of the most cited articles (n=40) focused on stroke and heart disease. Eight percent of the most cited articles in 2015 had high altmetric scores in 2013.
Conclusions:
Analysis of the highest altmetric-scored articles (n=40) from the initial 2009-2013 sample shows there is no direct correlation between initial high altmetric scores and the number of times the article was cited in 2015. What the data does show is 40% of the articles with the highest altmetric scores all three years were about stroke, obesity, and diabetes. Over half of all evaluated publications were from the 7 journals included in Medline's core clinical subset. This indicates articles in core clinical journals, on common chronic diseases are likely to receive the

highest altmetric scores. In contrast, just 8% of the articles cited most often in 2015 had high altmetric scores in 2013. In 2015, the top 25% of most cited articles were about heart disease, stroke, hepatitis c, thyroid, epilepsy, fungal genome, and delayed effects of prenatal exposures. The majority were from 3 journals included in Medline's core clinical subset. These findings seem to indicate that articles with high initial interest in social media cannot predict a long term impact in the scientific community.

Poster Number: 62

Time: Sunday, May 15, 2016, 2:00 PM – 2:55 PM

Designing a Library Resource Module for an Interprofessional Curriculum

Elizabeth G. Hinton, AHIP, Reference Librarian, Informational Services; Susan B. Clark, Library Director; Rowland Medical Library, University of Mississippi Medical Center, Jackson, MS

Objectives: To integrate a library module into an emerging campus interprofessional education (IPE) initiative by collaborating with faculty from the Office of Academic Affairs, the School of Pharmacy, and the School of Health Related Professions. Success of the module will be measured by student engagement and responses to an assessment given at the end of the course.

Methods: The library is participating in a new campus wide IPE course in order to promote the importance of using evidence-based resources in an interprofessional healthcare setting. Students from the schools of Nursing, Medicine, Dentistry, Health Related Professions, Pharmacy, and Graduate Studies will meet in assigned small groups for tabletop exercises and complete individual assignments in the learning management system (Canvas). Librarians will select a relevant case study scenario to be added to the online curriculum. Based on provided descriptions of the resources (UpToDate, DynaMed, VisualDX, and TOXNET), students will be asked select a resource for use in the clinical scenario and post reasons for their selection on the course's discussion board. Additionally, students will be directed to post a reflection to their interprofessional peers' responses. Results will be used to determine further library IPE endeavors.

Results: Out of the 51 students enrolled in the course, 30 participated in the library module discussion board. All schools of study were represented in the discussion: 5 students from the School of Medicine, 4 from the School of Dentistry, 4 from the School of Pharmacy, 4 from the School of Graduate Studies, 8 from the School of Nursing, and 5 from the School of Health Related Professions (dental hygiene, occupational therapy, health information management, and physical therapy were represented). Responses to the case studies were thoughtful and indicated a strong appreciation for the differing roles among healthcare professionals. Assessment of the library module effectiveness will be included in a forthcoming assessment of the entire IPE course.

Conclusions: Since the course was voluntary, student participation in the library module exceeded expectations and the pilot program was considered a

success. Once assessment data is analyzed, the format of future IPE library modules can be considered.

Poster Number: 65

Time: Sunday, May 15, 2016, 2:00 PM – 2:55 PM

Letters from the Big House: Providing Consumer Health Reference for Texas Prisons

Emily Couvillon, Liaison Librarian, TMC Library, Houston, TX; Adela Justice, AHIP, Senior Librarian, The Learning Center, UT M.D. Anderson Cancer Center, Houston, TX

Objectives: Librarians at a large academic medical library provide consumer health reference to prisoners across the state of Texas. Inmates correspond with librarians via US mail regarding their consumer health questions. The prisoner letters are a valuable record of the information needs of those confined to these facilities. This poster identifies key health concerns of those utilizing the prisoner reference service.

Methods: The authors of this study reviewed the content of the letters for recurring questions and themes. 192 letters from 45 prisons over the past seven years were analyzed using conventional content analysis. An initial coding scheme of key concepts was created by the two investigators, both of whom have experience answering prisoner letters. Each investigator independently applied these codes to an initial set of ten letters in order to test and refine the coding scheme. Discrepancies were discussed between the investigators and resolved before coding the full set of letters. Using a general inductive approach, investigators coded with a focus on identifying themes and understanding relationships among themes.

Results: The majority of requests were for consumer health overviews of a disease or drug, suggesting prisoners are most concerned with being more informed about their personal health issues. Analysis also revealed distrust of physician diagnoses, concern over prison environmental conditions, and desire for legal assistance.

Conclusions: The prisoner reference program sheds light on health information needs of Texas prisoners. Many are facing serious chronic conditions and their questions suggest limited access to on-site health information. Understanding prisoner health concerns can inform better information dissemination practices in the future.

Poster Number: 69

Time: Sunday, May 15, 2016, 2:00 PM – 2:55 PM

Health Literacy Promotion Achieved Through a Mosaic of Approaches and Teams: Case Studies Shared

Erica Lake, Associate Librarian; Jean P. Shipman, AHIP, FMLA, Director; Alice Weber, Emeritus Faculty; Spencer S. Eccles Health Sciences Library, Spencer S. Eccles Health Sciences Library, Salt Lake City, UT

Objectives: The responsibility to improve health literacy rests not just on patients, but on all health care providers and administrators. Health sciences librarians can be invaluable in supporting and promoting health literacy in their organizations. Collaborations that include teams of clinicians, administrators, librarians, students, and the community can have an impact on improving health outcomes, reducing costs, and empowering patients.

Methods: Through strong partnerships with inter-discipline health care professionals, librarians at a university health sciences library are encouraging patients and the general community to improve their health literacy through a series of projects. A variety of case studies will be presented for peers to emulate: the creation of a consumer health library; an innovative clinic health information kiosk; a tutorial for health sciences students to improve their awareness of the importance and impact of health literacy; joint research into health behaviors and outcomes affected by health literacy; a patient-centered digital medical home; and the inventorying of relevant and appropriate in-house patient education materials.

Results: Results from the various projects will be grouped to provide themes for other librarians to consider when implementing similar programs. Overall, librarians were coordinators of the projects and were able to work with other health care team providers to disseminate the results of the projects more broadly across the institution.

Conclusions: Results from the various projects will be grouped to provide themes for other librarians to consider when implementing similar programs. Overall, librarians were coordinators of the projects and were able to work with other health care team providers to disseminate the results of the projects more broadly across the institution.

Poster Number: 72

Time: Sunday, May 15, 2016, 2:00 PM – 2:55 PM

Supporting Terminology Standards and Interoperability in Nursing Practice

Erin D. Foster, NLM Associate Fellow, Oregon Health & Science University, Portland, OR

Objectives

To develop a National Library of Medicine (NLM)

web page that provides information on nursing terminologies and standards, demonstrates how to find synonymous terms between nursing terminologies, and links to relevant resources that support nursing care documentation. The creation of this resource

responds to NLM stakeholder

needs

and is intended to address legislative requirements (i.e., Meaningful Use)

for documenting health information within the context of nursing practice.

Methods

The creation of the web page included: determination of scope and audience, development and identification of web page content and resources, and production of a video tutorial.

Results

In July 2015, the NLM released the web page:

Nursing Resources for Standards and Interoperability
.
The scope and audience of the webpage were determined through consultation with nursing subject experts. The intended users are nurses, nursing students, nursing informaticists, and those exploring nursing terminologies for systems development and/or integration purposes.
The
page's content focuses on clinical and nursing terminologies (e.g., SNOMED CT, LOINC)
used in documenting health information and includes resources that support nursing practice. Finally, a video tutorial was produced to demonstrate how to extract synonymy between nursing terminologie
s
,
using
the Unified Medical Language System (UMLS)

Metathesauru
s
,
for the purposes of facilitating data exchange
.

Conclusion
s

The ability to share and encode patient data is a key step toward the development and evolution of nursing practice. This web page not only highlights valuable
NLM
resources, but contextualizes the need for interoperability

between nursing terminologies and those terminologies that are legislatively mandated for use in electronic health records (EHRs). As such, the

[Nursing Resources for Standards and Interoperability](#) web page is a valuable resource for the nursing community.

Poster Number: 75

Time: Sunday, May 15, 2016, 2:00 PM – 2:55 PM

Refining an Automated Process for Tracking Institutional Publications

Evan Sprague, Library Assistant, Medical Library, Medical Library, Rochester, MI

Objectives: Tracking institutional publications is a time consuming process that many libraries undertake in order to meet Liaison Committee on Medical Education requirements for accreditation. A medical library at a recently fully accredited medical school has been developing a computer script using Perl to automate parts of the task in hopes of expediting this process.

Methods: The library has established a step-by-step process for locating institutional publications beginning with searching various resources, such as Scopus, Web of Science, and PubMed. The located citations are then imported and stored in the citation management tool, EndNote. To identify authors affiliated with our institution, XML data is exported from EndNote and sent through the Perl script. This data can be imported back into EndNote for further manipulation as needed. This poster depicts our continued refinement of the search algorithm in the script used to locate authors associated with the institution, via a plan-do-check-act (PDCA) cycle. It is anticipated that this program will be made open source to aid other libraries in the endeavor of identifying and managing institutional scholarly publications.

Results: In a sample set of 329 citations, the script located 75.3% of the authors correctly. 18.8% of the authors were false positives and 5.9% of the institutional authors were missed. Some false positives are expected to be unavoidable. The missed authors were mostly due to not accounting for non-alphanumeric symbols in names, such a hyphens. Further refinement of the script should enable it to locate most of the authors it missed.

Conclusions: This script shows promise in reducing the time needed to locate an institution's authors. It will still be necessary to confirm authors after the script has been run. However, once the script can take into account the exceptions that prevented it from locating the 5.9% of authors it missed, it will allow the user to only confirm likely institutional authors. This will greatly reduce the time and effort needed in identifying and managing institutional scholarly publications

Poster Number: 78

Time: Sunday, May 15, 2016, 2:00 PM – 2:55 PM

Participation of an Academic Health Sciences Library in a University First-Year Summer Read Program

Frances A. Delwiche, Library Associate Professor, Dana Medical Library, University of Vermont–Burlington

Objectives: The First-Year Summer Read Program is a common feature of the first-year experience for students at university campuses nationwide. When the book selected has a strong medical theme, it presents an outstanding opportunity for academic health sciences librarians to reach out to faculty and students, to play an active role in campus life, and to engage with the larger community.

Methods: In the First-Year Summer Read Program, all incoming students are asked to read a book that has been selected for its interdisciplinary relevancy and thought provoking content, thus providing students with their first exposure to academic life. In 2013 and 2015, an academic health sciences library actively participated in its university's First-Year program by creating an in-house educational exhibit on the topics of cholera and epilepsy, respectively. Building on the conviction that exhibits can serve as powerful tools for education and outreach, this poster describes the process of developing the exhibits' content; creating a viable design, given limited resources; and publicizing the end products. The poster describes the outcomes of the projects, focusing on the rewards and challenges associated with this form of information dissemination, as well as the strengthening of key relationships between the library and multiple groups across campus.

Results: The exhibits consisted of four large, full-color posters mounted in a glass case located in the library's main entryway. Rich in images, the posters depicted the historic, scientific, and clinical aspects of the diseases featured in the books. Content was drawn from a wide range of scholarly resources, as well as conversations with scholars, healthcare professionals, and members of the community. Faculty and graduate students currently conducting research on the diseases were invited to contribute content to the exhibits, highlighting their research. A bibliography, selected readings, and books from the circulating collection augmented the visual experience. Both exhibits were launched in early September of their respective year, and remained on display throughout the fall semester. Publicity was disseminated across campus via the usual broadcast channels and through library liaison communiques, culminating in a campus-wide reception held late in the semester.

Conclusions:

The First Year Summer Read program has provided the Library with a valuable opportunity for outreach and collaboration with faculty and students, researchers, healthcare professionals, and members of the community. Through its involvement, the Library provided the campus community with a unifying experience, and solidified the perception of "Library as Place" in the minds of a new generation of students. The Library looks forward to expanding its role in the program by participating in the selection of books for future classes.

Poster Number: 81

Time: Sunday, May 15, 2016, 2:00 PM – 2:55 PM

Analyzing Website Usage by Intended Audiences

Gale Dutcher, AHIP, Deputy Associate Director, Specialized Information Services, National Library of Medicine, Bethesda, MD; Andrew Plumer, Outreach Librarian, Division of Specialized Information Services , NLM, Bethesda, MD; Janice E. Kelly, Chief, Outreach and Special Populations Branch, Specialized Information Services, Specialized Information Services, Bethesda, MD

Objectives: A large federal library sought to examine ways to increase the reach of two specialized online health information resources by health professionals and the public by analyzing website referrals and social media platforms. The library hoped to understand whether the specialized content was effectively reaching its intended audiences and determine ways to increase its reach.

Methods: A study was undertaken to analyze how website referrals, email, and social media drive users to two specialized health information online resources. Different strategies were employed to analyze key performance measures for the websites and each social media outlet. We analyzed website referral traffic and estimates of the intended audience reached. In addition, we analyzed newsletter subscriber engagement and registration paths for sign up, current Twitter followers and recent conversations, Facebook posts and communication factors that drive the most engagement and cross referenced email subscribers and social media supporters to determine the most active users. Study findings suggest you can increase audience potential by optimizing search engine results, increasing the sharing potential of social media posts, positioning newsletters on the website for easy registration and using each outlet to recruit users for other outlets.

Poster Number: 84

Time: Sunday, May 15, 2016, 2:00 PM – 2:55 PM

Improving Research Skills of Graduate Students: A Collaboration Across Disciplines

George Shaw Jr., Student, University of South Carolina, School of Library and Information Science, Sumter, SC

Objectives: Effective writing and information-seeking skills are necessary for students who plan to enter a post-master's program, such as a medical or doctoral program. However, many graduate programs do not offer opportunities for students in clinical and allied health related disciplines to collaborate on research projects while improving their research and writing skills.

Methods: The study uses a self-perceived questionnaire on a 5-point Likert scale that ranges from "1" (very incompetent) to "5" (very competent). The study identifies the level of perceived professional skills of students in health related graduate programs. SACS accredited Colleges and Universities in the South Carolina midlands geographic area that have a Nursing, Medicine, Pharmacy, or Allied Health related discipline graduate department were selected to participate in the questionnaire. Using a grounded theory methodology, written comments from students will be analyzed to identify common themes and provide additional insight into students' writing skills and confidence to conduct academic research projects. The results from the study will show that students will have a high perception of their medical knowledge, problem-solving abilities, communication skills, and basic medical and science knowledge. Students' research skills and the ability to write scientific reports and conduct research projects will be low.

Results:
Results are still being finalized and will be available during Poster Session I on-site in May

Conclusions: Preliminary results show that students enter into graduate level programs with a varying degree of writing skills. Typical writing concerns when writing include style, organization, and academic voice. Students expressed the lack of communication and research collaboration between faculty members of the area colleges.

Poster Number: 87

Time: Sunday, May 15, 2016, 2:00 PM – 2:55 PM

Librarians Plan a Virtual Annual Meeting: You'll Never Guess What Happened Next!

Heather L. Brown, AHIP, Head of Collection Services, University of Nebraska Medical Center, McGoogan Library of Medicine, Omaha, NE; Rebecca Graves, AHIP, Education Services Librarian, J. Otto Lottes Health Sciences Library, Columbia, MO

Objectives: A virtual annual meeting was held by the Midcontinental Chapter of the Medical Library Association in 2015. The Chapter's goal was to simplify planning, so that the process required less time and personnel than in-person meetings. This poster details the execution and results of a virtual conference. Additionally, lessons learned by the planning committee will be offered.

Methods: A small group of planners coordinated various aspects of the meeting without the traditional system of committees. An in-person meeting was used as the template for the planning process. Customary elements of the meeting were translated in to the virtual environment and scaled down considerably. Some traditional elements of in-person meetings, such as hospitality and local arrangements, were eliminated. Other were replaced, such as venue selection which became online platform choice and coordination. A new template for meeting planning emerged, with input from registrants who posed questions and scenarios not previously considered by the planners. In addition to an analysis of registration, revenue, and expenditures, a survey was conducted after the meeting to assess the quality of the content and opinions on the virtual format.

Results: Registration and attendance exceeded expectations, with 25 individual registrants attending and 136 individuals attending as part of 10 group registrations. One group was from another Chapter. Social media interaction took place on Twitter and Facebook, with attendees posting photos and comments throughout the meeting. Additionally, expenditures for the online platform were lower than anticipated, allowing for a profit for the Chapter. Of the respondents to the meeting evaluation (n=79), 82% (n=65) indicated that they would attend a virtual MCMLA annual meeting again. The keynote was rated Excellent by 82% of attendees and overall, the sessions were rated as useful to the needs of attendees or their organization. Though comments were overall positive, there was mention of the lack of networking.

Conclusions:
Over two four-hour online sessions, the attendees viewed sessions that mirrored a face-to-face annual Chapter meeting. Though a virtual meeting

cannot meet all the needs of the in-person meeting, the quality of the content, the positive experience had by the majority, and the scaled-down planning process proved to the Chapter that a virtual meeting is an effective and practical method to conduct Chapter business. The MCMLA Chapter will be adding virtual meetings to the meeting rotation.

Poster Number: 90

Time: Sunday, May 15, 2016, 2:00 PM – 2:55 PM

Beyond Searching: Nontraditional Roles of a New Health Sciences Librarian

Iris Kovar-Gough, AHIP, Health Sciences Librarian, Michigan State University Libraries, East Lansing, MI

Objectives: How can librarians become integrated into medical school curricula? Are there options beyond traditional search skills tutorials? This poster will explore the ways one health sciences librarian advocated for increased curriculum integration and the non-traditional skills, competencies, and roles employed.

Methods: A medical school curriculum redesign created opportunities for a new liaison librarian to embed herself. By advocating for integration in a proposal to college deans, making connections with faculty, identifying gaps in student competencies, and promoting her expertise, the librarian now participates in college curriculum projects. These include consulting on a curriculum-mapping database, creating an online intervention to improve student performance on a literature search exam, developing digital media standards for a medical illustration image bank, and becoming the information literacy expert in an evidence-based medicine (EBM) curriculum workgroup and a team member on a general curriculum workgroup. The liaison's efforts were evaluated using faculty feedback and student performance data. Anticipated outcomes are integration of information literacy skills into the curriculum, improved information architecture for the development and assessment of the new curriculum, and students with increased competence in EBM.

Results:
The librarian has become integrated into the medical school curriculum beyond the traditional role of "expert searcher." This competency still plays a role in her professional responsibilities but she has been able to extend her College's view of her expertise to include information architecture, curriculum design, and health information literacy broadly. Feedback from faculty has been generally positive and the College has welcomed her participation in curriculum projects that may have been previously seen to be outside the scope of her area of expertise.

Conclusions: By cultivating positive relationships with faculty and being open to new and unusual avenues for curriculum integration outside of one-shot searching instruction the health sciences librarian has started to be integrated into the medical school curriculum. Integration can take many forms and in this example her expertise and willingness to learn new skills and collaborate inside

and outside of the library has led to librarian curriculum involvement outside of the norm.

Poster Number: 93

Time: Sunday, May 15, 2016, 2:00 PM – 2:55 PM

How Coloring Outside the Lines Led to Working Outside the Box: A Continuation of a Library Tech Group's Challenges and Keys to Successfully Implementing New Tech Services in the Library

Jennifer Herron, Emerging Technologies Librarian, Ruth Lilly Medical Library, Ruth Lilly Medical Library, Indianapolis, IN; Kellie Kaneshiro, AHIP, Library Technology Team Leader, Ruth Lilly Medical Library, Ruth Lilly Medical Library, Indianapolis, IN; Jason Lilly, Library Systems Analyst, Ruth Lilly Medical Library, Indiana University School of Medicine, Indianapolis, IN; Gabriel Rios, Library Director, Ruth Lilly Medical Library, Ruth Lilly Medical Library, Indianapolis, IN

Objectives: Our library technology group previously identified the unmet technology needs of our library and is using a guided approach to offer these new services and opportunities to our users. Our group began utilizing connections from departments and people outside the library in our institution to help us develop, enhance, and promote our new services.

Methods: In our previous technology needs assessment our group identified three key areas to focus our efforts on. These areas include: increasing our social media presence to reach users outside the library (and located at different campuses across the state), designing a makerspace, and setting up a 3D printing service in the library. Our group's initial "if you build it" approach with our new 3D printing service did not yield as much user engagement or activity as desired and led to our collaboration with people and departments outside of the library. Some of the departments our group has worked with include: Informatics, Communications, Learning Technologies, University Information Technology Services, Advanced Visualization Lab, and Occupational Therapy. Working with this mixture of backgrounds has helped our group troubleshoot issues, identify knowledge gaps, and find collaborators for future events.

Results: The library technology group reviewed the areas we wanted to focus our efforts and then identified what resources within the university and outside of the library might help us with these areas. Our group met with a variety of departments which led to new partnerships and support for our 3D printing initiative.

Meetings with all departments and faculty members highlighted a shared interest in developing a 3D conference for all interested people to attend and discuss their experiences in 3D printing, modeling and provide a chance to

share knowledge between groups on campus. This is currently in the works between members of the library technology group and individuals met during these outside meetings.

Conclusions: Going outside the library has led to numerous partnerships and collaborations which will enhance the services that library's new makerspace will be able to offer. Furthermore, the library now has support from multiple departments and faculty members for assistance with 3D printing and modeling. These meetings have helped to form a collaborative network of those involved with 3D printing and modeling on campus. This network will serve as support for troubleshooting in 3D printing and provide access to the group's collective knowledge for advice and support in 3D printing.

Poster Number: 96

Time: Sunday, May 15, 2016, 2:00 PM – 2:55 PM

The Role of the Embedded Clinical Librarian in a Chronic Pain Management Telemedicine Learning Program

Jessica Babineau, Information Specialist, Library and Information Services, Toronto Rehab - University Health Network, Toronto, ON, Canada; Jane Zhao, Research Coordinator, ECHO Ontario Chronic Pain and Opioid Stewardship, Toronto Rehab - University Health Network, Toronto, ON, Canada; Ruth Dubin, MD PhD, Co-chair of ECHO Ontario, ECHO Ontario Chronic Pain & Opioid Stewardship, Associate Adjunct Professor, Department of Family Medicine, Queen's University, Kingston, ON, Canada; Andrea D. Furlan, MD PhD, Co-chair of ECHO Ontario, ECHO Ontario Chronic Pain & Opioid Stewardship, Associate Professior, Department of Medicine, University of Toronto, Senior Scientist, University Health Network, Toronto, ON, Canada

Objectives

To describe the role and value of an embedded clinical librarian within an innovative interprofessional team that delivers chronic pain education to Primary Care Providers (PCPs) in underserved urban, rural, and remote areas.

Methods

ECHO Ontario Chronic Pain and Opioid Stewardship (ECHO Ontario) is the first replication of the ECHO model in Canada and
began with a goal to help PCPs to provide better chronic pain management. Using a hub-and-spoke model, an interprofessional hub team delivers education to multiple spoke PCPs through telemedicine. The embedded clinical librarian is an important part of the interprofessional hub team, attending each weekly session. Further discussions also occur outside sessions through an online message board and via email. Questions arise from both hub and spoke members regarding didactics, patient case presentations and online message board discussions. The librarian provides evidence-based resources to the community based on these questions that arise. To determine the value of the librarian's role, questionnaires were distributed to the community and metrics were collected. The scope of the librarian's role was also assessed.

Results

From June 2014 to November 2015, the librarian attended 61 out of the 69 ECHO Ontario sessions, conducted 29 literature searches, and spent an average 36 minutes per week on ECHO related tasks in addition to attending weekly 2 hour sessions. Based on questionnaire results, the embedded clinical librarian role was received well by both interprofessional hub members (n=13) and spoke PCPs (n=27).
Ten (76.9%) hub members and 22 (81.5%) spoke members answered *yes* to the question "Should we continue to offer clinical librarian services during ECHO sessions?"
Feedback was positive and encouraged continued use of librarian services. Questionnaire feedback, however, suggested a lack of awareness from some spoke members regarding the specific role and services provided by the librarian. When reassessing the clinical librarian role, the scope of responsibility grew from
providing evidence-based resources to also include current awareness resources and addressing copyright and information dissemination issues.

Conclusions

The embedded clinical librarian role has been a valuable part of ECHO Ontario and has been well received. This new role demonstrates the value of a librarian within an interprofessional team focused on primary care education using telemedicine, with the ongoing, iterative goal in mind of how to best meet the needs of the ECHO Ontario community.

Poster Number: 98

Time: Sunday, May 15, 2016, 2:00 PM – 2:55 PM

Narratives of Access

JJ Pionke, Applied Health Sciences Librarian and Assistant Professor, Social Science, Health, and Education Library at the University of Illinois Urbana Champaign, University of Illinois at Urbana-Champaign, Champaign, IL

Objectives: This project delves into the narratives and experiences that people with different kinds of disabilities, who are at various levels of academic engagement, experience in the library. These narratives include obstacles and potential solutions so that the participants have a sense of ownership in how these issues will be addressed.

Methods: This study will utilize an interview approach at a large university. There will be interviews spread out over four disabilities (sight, mobility impairment, Autism, and Post Traumatic Stress Disorder) and three classes (undergraduate students, graduate students, and faculty/staff).

Results: A total of 8 interviews were conducted that encompassed four disabilities (Post Traumatic Stress Disorder, vision impairment, Autism, and mobility impairment). Participants included all class groups at the university (undergraduate students, graduate students, and faculty/staff).

Conclusions: Participants had a lot to say in terms of not only what was working in the library in terms of physical, digital, and human interventions as well as what was not working. Their suggestions were well thought out and helped illustrate the need for major changes not only in actual interventions but in how libraries in general think about functionally diverse peoples.

Poster Number: 101

Time: Sunday, May 15, 2016, 2:00 PM – 2:55 PM

Looking Backward to Go Forward: Five Years of Health Sciences Library Practicums: What Have We Learned?

Jonna Peterson, Senior Clinical Informationist, Northwestern University, Galter Health Sciences Library - Northwestern University, Chicago, IL; Rebecca Raszewski, AHIP, Associate Professor & Information Services Librarian, University of Illinois at Chicago, Library of the Health Sciences, Chicago, IL

Objectives: This abstract reflects on a public services health sciences library practicum over the last five years. The authors will discuss what the students and librarians have learned from the practicum and future directions.

Methods: The practicum is a collaboration between a private and a public health sciences library. Eight students participated in the practicum between June 2011 and April 2015. This practicum requires the student to work 120 hours; 60 at each institution. The student completes projects at each institution and is given first hand opportunities to observe and network with librarians. The student is responsible for a weekly report and a culminating presentation outlining the experience. This project seeks to determine if the practicum influenced the students to obtain a position in a health sciences library. Students were interviewed at the completion of their practicum and again one year after the practicum was completed or the student had graduated. The interviews were conducted by an employee of one institution who had limited contact with the student during their practicum experience.

Results: During the practicum, the student utilized core health sciences databases, medical reference books and journals. By observing librarians working in both onsite and virtual settings, the student experienced a variety of library instruction and library skills workshops. Six students completed the initial interview. Two students could not be contacted for the study. Four students completed the second interview. One student was working full time in a special library. One student was working part time in a public library. Another student had a full time position in an academic library. One student had not obtained a library position at the time of her second interview, but was hired shortly after to work at one of the participating institutions.

Conclusions: In the last five years of planning library practicums, the supervising librarians have made adjustments to both the process and content. The recruitment ad has been modified and the practicum has been offered during two semesters. Originally, the practicum was advertised at Midwest

library schools only. With the proliferation of distance graduate library education programs, the list of institutions has been expanded to include schools nationwide. Our recruitment ad is also posted to a variety of library listservs. The practicum fills a need in library education as many library schools do not consistently offer health sciences courses.

Poster Number: 109

Time: Sunday, May 15, 2016, 2:00 PM – 2:55 PM

Implementing a Demand-Driven Acquisitions Pilot

Karen McElfresh, AHIP, Resource Management Librarian, Health Sciences Library and Informatics Center, The University of New Mexico Health Sciences Library and Informatics Center, Albuquerque, NM

Objectives: The purpose of this poster is to describe the implementation of a Demand-Driven Acquisition (DDA) plan at an academic health sciences library. The poster will also describe how the library's new Integrated Library System (ILS) supports DDA plans by providing an automated workflow to manage pools of available and purchased titles.

Methods: DDA plans allow libraries to vastly increase the number of titles available in their collection and typically result in overall cost-savings because titles are not purchased unless they are accessed by users. The University of New Mexico Health Sciences Library & Informatics Center piloted a DDA plan in 2015-16 as a way to provide e-books on narrow topics that typically would not be of interest to a wide number of users. Librarians reviewed various DDA programs with different providers and chose one that works seamlessly with the library's ILS, OCLC's WorldShare Management Services. The success of the pilot will be determined by looking at the number of titles loaned and/or purchased, total expenditures, and cost per use. Additionally, the vendor allows the library to create survey questions users must answer before accessing a title, and data from these questions will also be examined.

Results: Titles in the DDA plan received much more use than we anticipated. Within 3 months, there were nearly 200 short-term loans and 8 titles were auto-purchased. The majority of the use of titles in the plan came from students and residents, followed by faculty and staff.

Conclusions: Overall, the DDA pilot was very successful and we plan to continue to use this acquisitions model in the future.

Poster Number: 111

Time: Sunday, May 15, 2016, 2:00 PM – 2:55 PM

They Like Us! They Really Like Us! Using Appreciative Inquiry to Evaluate Outreach to Unaffiliated Health Professionals

Kate Flewelling, Outreach Coordinator, National Network of Libraries of Medicine, Middle Atlantic Region, National Network of Libraries of Medicine, Middle Atlantic Region, Pittsburgh, PA

Objectives: To determine what unaffiliated health professionals valued about their experiences with the medical library. To identify ways to publicize library services to unaffiliated health professionals.

Methods: Eight phone interviews were conducted with unaffiliated health professionals who had received services from the library. Participants who were chosen were either members of an advisory group or who had been funded to conduct health information outreach. Representatives from mental health, public health and clinical health were included.
Using an Appreciative Inquiry evaluation model, staff asked participants:
1. Tell me about the best experience you have had with the library?
• What specifically did you like about that experience?
• What did you value about that experience?
• What do you wish the library could offer you?
2. What would you tell your colleagues would be a reason(s) to become involved with the library?
3. What are the best ways to publicize our services to professionals like you and to your constituency?

Poster Number: 113

Time: Sunday, May 15, 2016, 2:00 PM – 2:55 PM

Assessing Undergraduate Nursing Students' Information Needs and Perceptions of the Library: A Longitudinal Evaluation: Year 1

Kate Saylor, Health Sciences Informationist; Emily C. Ginier, Health Sciences Informationist; Nandita S. Mani, AHIP, Assistant Director, Academic & Clinical Engagement; Taubman Health Sciences Library, Ann Arbor, MI

Objectives: The project will follow incoming undergraduate students in the traditional BSN program at University of Michigan School of Nursing (UMSN) over four years. Using data from yearly assessment surveys and focus group interviews, this project will allow us to identify gaps in information seeking skills, inform our instruction, improve selection and development of learning tools and resources, and identify the best time for library interventions and communication.

Methods: Data will be collected for our longitudinal evaluation over 4 ½ years as we follow a single cohort of students. Our yearly assessment surveys will be distributed to the entire cohort. Based on recent enrollment reports, we estimate that the survey will be distributed to 150-160 students. Our 5-7 focus group participants will be recruited from the study cohort. The focus group will meet once per year for 4 years. Individual follow-up interviews will be conducted with the focus group participants 6 months post-graduation. Since the participants may relocate post-graduation, these interviews will be conducted through conference or video calls. We will analyze our qualitative data using a coding frame that we will develop inductively throughout the coding process.

Results and Conclusion: We are currently analyzing data. The results and conclusion will be presented on the poster.

Poster Number: 116

Time: Sunday, May 15, 2016, 2:00 PM – 2:55 PM

One Size Doesn't Fit All: Meeting the Needs of Hospital Staff Through Interprofessional Collaboration and a Responsive Website Update

Katie D. McLean, AHIP, Librarian Educator BA, LIT, MLIS, Library Services, Nova Scotia Health Authority , Halifax, NS, Canada; Katie Quinn, Library Technician, Nova Scotia Health Authority, Hospital Library Services, Halifax, NS, Canada; Vivien Gorham, Library Technician, Nova Scotia Health Authority, Hospital Library Services, Halifax, NS, Canada; Chelsey Millen, Library Technician/Program Assistant, Nova Scotia Health Authority, Leadership Development, Halifax, NS, Canada; Jackie Zoppa, Library Technician, Nova Scotia Health Authority, Hospital Library Services, Dartmouth, KS, Canada

Objectives: In order to be truly accessible, library websites must adapt to both screen size and user needs. Updating to LibGuides 2 CMS and working with hospital staff (e.g. knowledge translator, occupational therapist) on four collaborative projects allowed our library to address both factors.

Methods: A content management strategy and basic style manual were developed to support use of the updated website. Four new projects (Elder Care in Hospital, Wound Care, Primary Health Care and the Wheelchair Skills Training Program) were developed in collaboration with hospital staff. For two of the projects, select hospital staff were trained and given access to add and update content. Prior to the release of each project, an interprofessional editorial team of hospital and library staff carried out a final review of layout, language and functionality. Website statistics from Google Analytics were monitored both before and after the update. Statistics for the new projects were also compared to long-standing library web page statistics.

Results: Of the four projects, three resulted in the library hosting content entirely. One project involved the library developing web content to be hosted in a separate content management system. All projects involved working with hospital staff to edit, lay out and enhance content for the web.

Conclusions: Hospital libraries can play a vital role in web content design and management. Usage statistics for library-supported web resources show consistent use and rank highly compared to other organizational guides and websites. This role allows the library to become embedded in the work that hospital staff do, and leads to future collaborations.

Poster Number: 117

Time: Sunday, May 15, 2016, 2:00 PM – 2:55 PM

Medical Librarians' Contributions to Instruction on Organizing Scholarly Research

Keith Engwall, AHIP, Assistant Professor, Web & Emerging Technologies Librarian, Medical Library, Oakland University William Beaumont School of Medicine, Rochester, MI; Stephanie M. Swanberg, AHIP, Assistant Professor, Information Literacy & eLearning Librarian , Medical Library, Oakland University William Beaumont School of Medicine, Rochester, MI; Stephen Loftus, Associate Professor, Oakland University William Beaumont School of Medicine, Biomedical Sciences, Rochester, MI

Objectives: Beyond teaching search strategies and other information mastery skills, librarians can also provide meaningful instruction on the organization of scholarly research as a preparatory step towards publication or presentation. From development of structured abstracts to design of posters and presentations, librarians can draw upon their experience in managing and organizing information to provide value to the medical school curriculum.

Methods: Librarians at a newly accredited medical school have identified opportunities to extend library instruction beyond locating information and provide students with best practices for organizing their scholarly research. In addition to information mastery instruction given in the M1 year, librarians also provide one session on developing a structured abstract and another on the design of posters and presentations in the fall of the M2 year. These sessions are provided within the medical school's longitudinal Capstone course, in which medical students participate in a research project spanning their medical school career, including presentation of their progress in the M2 and M4 years. This poster will highlight the objectives and content of these sessions, how they fit into the broader course structure, student evaluations, and how the sessions have evolved. Benefits, challenges and lessons learned will also be covered.

Results: The Medical Library was responsible for three mandatory M2 sessions in the 2014-2015 and 2015-2016 academic years:
2014-2015

- Structured Abstracts, 90 minutes

- Best Practices for Scholarly Presentations, 90 minutes

- Creating and Critiquing Scholarly Posters, 30 minutes

2015-2016

- Structured Abstracts, 90 minutes

- Best Practices for Scholarly Presentations and Posters, 90 minutes

- Strategies for Publishing, 30 minutes

Student evaluations were mostly positive for all sessions in both years. Faculty continue to be very welcoming to librarian involvement in the curriculum. Content has been expanded and rearranged, and these sessions or their equivalent are expected to be taught by librarians in the future.

Conclusions: Opportunities for instruction beyond information mastery exist for librarians in a medical school curriculum. Through faculty engagement in course development, sessions drawing upon the expertise of librarians can provide value to medical students.

Poster Number: 119

Time: Sunday, May 15, 2016, 2:00 PM – 2:55 PM

Reflections on Turning Forty: The Ottawa Valley Health Libraries Association/Association des bibliotheque de la sante de la Valle de l'Outaouais History Project

Kelly Farrah, AHIP, Research Information Specialist, Information Services, CADTH, Ottawa, ON, Canada; Jessie Cunningham, Librarian and Records Management Assistant, Canadian Medical Protective Association, Library, Ottawa, ON, Canada; Caitlyn Ford, Research Information Specialist, Information Services, CADTH, Ottawa, ON, Canada; Jennifer Skuce, Information Technician, CADTH, Information Services, Ottawa, ON, Canada; Michelle Purcell, Medical Librarian, Arnprior and District Memorial Hospital, Library, Arnprior, ON, Canada

Objectives:
The Ottawa Valley Health Libraries Association (OVHLA)/l'Association des bibliothèques de la santé de la vallée de l'Outaouais, originally the Ottawa-Hull Health Libraries Group, dates back to 1974. The Association was one of the first chapters of the Canadian Health Libraries Association/Association des bibliothèques de la santé du Canada. In 2015 the bulk of its history was contained in boxed paper records and the memories of various members throughout the years. The objective of this project was to collect, organize, and share our historical documents, and to create a timeline of the Association's important moments.

Methods:

A working group of chapter members was formed to gather, organize, and archive information related to the development and activities of the Association. Past and present OVHLA members were solicited to contribute historical records. Historical documents and artifacts, including meeting minutes, chapter reports, membership lists, treasurer records, and photographs were reviewed and prepared for archiving.
Issues of Journal of the Canadian Health Libraries Association (JCHLA) and Bibliotheca medica Canadiana were also scanned to identify relevant information. Additionally, questionnaire-based interviews were conducted via email with six past-presidents and one long-standing OVHLA member to gather their reflections.

Both the historical records and the interview responses were analyzed to develop a narrative review and timeline of the Association's significant moments.

Results:
Outcomes from the OVHLA history project include: a narrative review of the Association's history published in JCHLA, a timeline of OVHLA's key achievements, submission of OVHLA's records for deposit in the Archives and Special Collections of the University of Ottawa, and a new history section on the Association's website featuring important historical documents, a list of past executive members, and the timeline of achievements.

Conclusions

This project highlights OVHLA's rich history and has made the Association's records accessible to members as well as anyone interested in the heritage of health librarianship in the Ottawa Valley region.

Poster Number: 122

Time: Sunday, May 15, 2016, 2:00 PM – 2:55 PM

Is There More Research on Plant-Based Foods in Some Countries than in Others?

Xiaomei Gu, Clinical Education Librarian, Hardin Library for the Health Sciences, Hardin Library for the Health Sciences, Iowa City, IA; Eric Rumsey, Librarian, Hardin Library for the Health Sciences, Hardin Library for the Health Sciences, Iowa City, IA; Janna Lawrence, AHIP, Deputy Director, Hardin Library for the Health Sciences, University of Iowa–Iowa City

Objectives: With increased interest in the importance of diet in preventing and treating chronic diseases, plant-based foods (PBFs) have gotten much attention in recent years. Realizing that PBFs are a more important part of the diet in many non-US countries, we will analyze the country of authors for articles in PubMed on PBFs.

Methods: To determine whether some countries have more emphasis on PBF research than others, we will use a hedge that we have developed to search in PubMed for PBFs. We will combine this with a search for the country of the authors. In addition to searching for PBFs in general, we will also search for specific types of PBFs and combine this with the country of the authors.

Poster Number: 127

Time: Sunday, May 15, 2016, 2:00 PM – 2:55 PM

Introduction to Free and Low-Cost Information Resources for Optometrists at the 2015 American Academy of Optometry Annual Meeting

Kristin Motte, Director of Library Services, Library, New England College of Optometry, Boston, MA; Suzanne Ferimer, AHIP, Librarian & Director of Learning Resources, Weston A. Pettey Library/University of Houston Libraries, Weston A. Pettey Library, Hosuton, TX

Objectives: To provide attendees of the American Academy of Optometry, through a workshop at their annual meeting, evidence based references and resources, on-line journals, and other materials which they could access freely.

Methods: Drawing on resources and experiences providing instruction at our home institutions, the authors developed a resource list of free materials, databases, journal titles, websites, etc. that optometrists could access. Those attending the workshop were asked to bring a mobile device which was WiFi compatible. During the workshop the material was presented in sections and after each presentation the attendees accessed the resources presented as the instructors moved through the room answering questions.

Results: 19 AAO attendees pre-registered and 10 attended. While a small group, all present noted how useful the session was. The authors, along with a third collaborator, have proposed a follow-up workshop for the Academy 2016 in Anaheim, CA.

Conclusions: We feel that the Academy members now see the benefit of having librarians as partners in presenting information resources which help in bettering patient care. Our hope is that librarians will be included in future Academy programing.

Poster Number: 130

Time: Sunday, May 15, 2016, 2:00 PM – 2:55 PM

"Is Anyone out There?": Connecting Health Researchers Across the Arctic

Laura Bartlett, Technical Information Specialist, National Library of Medicine, Specialized Information Services, Outreach and Special Populations, Bethesda, MD; Sigrid Brudie, Medical Reference Librarian, Consortium Library, Alaska Medical Library, Anchorage, AK; Blake Hawkins, Graduate Student, University of British Columbia, Master's of Library and Information Studies, Vancouver, BC, Canada; Kathleen Murray, Alaska Medical Library Manager, University of Alaska Anchorage, Alaska Medical Library, Anchorage, AK

Objectives
To highlight the breadth, depth, and diversity of research, researchers, and data in the Arctic we are collecting and narrating a directory that will be available through the Arctic Health portal. This directory will connect researchers using their interdisciplinary research areas. This is a steps towards creating an open, sharing environment that will lead to data sharing among reseachers.

Methods
A mixed methods approach is being used which includes an environmental scan, development of a survey interview instrument, and development and assessment of a researcher profile template, An environmental scan was conducted to explore what researcher and data sets are currently available that can be incorporated in the directory. Using commonly asked questions by researchers to University of Alaska Anchorage staff, a researcher profile was developed. 30 researchers from mutliple disciplines including medical, scientific and humanities, have been recruited and profiled. These researchers are from Alaska, Canda, and other Arctic regions, including those working with indigenous populations. The researchers are testing the profile of others to determine if the profiles provide sufficient information to assess the relevance of research and data for the inquiring researcher. Refinement of the researcher profile template will be based on feedback. The Alaska Native Tribal Health Consortium has also leant their research directory to aid in development in the template.

Results
Through environmental scans and working with partner agencies, we have developed a researcher profile template. Our next steps are to populate the database with records from the University of Alaska, U.S. National Institutes of Health, and the Alaska Native Tribal Health Consortium. The Arctic Health team will be reaching out to other U.S. funding agencies, and international research and government organizations to contribute their information.

Conclusions
As Arctic research adopts "One Health," a research database is much needed to create the connections between research, researchers, and data.

Poster Number: 135

Time: Sunday, May 15, 2016, 2:00 PM – 2:55 PM

Facilitating Access to Lesbian, Gay, Bisexual, Transgender (LGBT)-Specific Health Information for Patients and Providers

Lindsay Blake, AHIP, Clinical Information Librarian, Robert B. Greenblatt, MD Library, Augusta University, Augusta, GA; David Kriegel, Director, Student Education, Georgia Regents University, Dept. of Family Medicine, Augusta, GA; Lara Stepleman, Co-Director Educational Innovation Institute, Georgia Regents University, Dept. of Psychiatry and Health Behavior, Augusta, GA

Objective: Develop an online health information portal that allows LGBT patients to access reliable online resources that specifically address LGBT health concerns and to facilitate learning, teaching, and patient care at a student run, free Equality Clinic which serves the under and un-insured in the LGBT community.

Methods: This project seeks to address underserved LGBT patients as well as facilitate service based learning through utilization of a web portal for medical professionals and patients. During the first phase of the project medical, psychological, and training resources were collected. Special attention was placed on resources for transgender health since 70% of Equality Clinic patients seeking health services, such as cross-sex hormone therapies, identify with this population. A web design librarian built the portal with this base of information. Health care providers started using the new portal with clinic patients in January 2016. An ongoing survey of clinic providers and patients helped identify information gaps and augment the existing pages to assist in the continuing development of the portal.

Results: The portal was debuted to LGBT Equality Clinic patients and the Equality Clinic Board in January 2016. The site was also featured in a panel session during Come Out for Health Week in March 2016. Cards were created to advertise the portal in the Equality Clinic waiting room and for clinicians to hand out to patients. Equality Clinic patients, volunteers, and community supporters contributed feedback, comments, and suggestions for improved navigation and additional resources. Especially helpful were the addition of many local resources known mostly by word of mouth within the local LGBT community.

Conclusions: The LGBT Health portal has been a useful resource for the clinic volunteers and patients. The suggested addition of local LGBT friendly doctors and facilities as well as many local resources known mostly by the established LGBT community have helped to make the site more useful to the local LGBT

community, especially to newly arrived members. Equality Clinic volunteers have reported that resources found within the portal have helped them to feel more confident in approaching, treating, and referring LGBT patients.

Poster Number: 136

Time: Sunday, May 15, 2016, 2:00 PM – 2:55 PM

Drop in Fridays: Literature Searching for All

Lisa A. Marks, AHIP, Director of Libraries, Mayo Clinic, Staff Library, Scottsdale, AZ; Diana Almader-Douglas, Librarian, Mayo Clinic, Hospital Library, Phoenix, AZ; Diana F. Rogers, Library Assistant, Mayo Clinic Arizona, Clinic Library, Scottsdale, AZ

Objectives: Library staff noticed a trend in requests for literature searching for school, work and non-work related projects. Not knowing what, if anything is being taught in the curriculum, library staff thought it would be worthwhile to have “office hours” or “drop in” time to assist with literature searching to improve self-sufficiency in researching and help improve literature searching skills.

Methods: Library staff established “Drop in Fridays” where staff could literally drop into the libraries to seek assistance with their project. Hours were set every Friday from 11:00am to 2:00pm to cover lunch breaks. A pre and post evaluation completed by each participant will measure knowledge before and after each session. Sessions will be approximately 15 minutes each with the option to make an appointment with the librarian for more in-depth, complicated searches. Librarians will also make the option of “coming to you” available for those who can’t get away from their work space. In addition, the project will include the development of internal marketing efforts to advertise and promote the project to staff, to expose library services and resources, to increase library usage, to educate library patrons about services and resources, and to assess the needs of library users.

Results: Our program began October 2, 2015. To this point, we have had 16 patrons "drop in" for assistance with their literature searching skills. Majority of our participants are from the department of nursing followed by allied health patrons. We learned that participants improved their searching skills and became more confident in searching independently. We also learned that 100% of participants plan to make use of our library resources and services in the future.

Conclusions: Based on the number of particpants and their comments to this point, we feel our "Drop in Fridays" program has been successful and will continue to offer the program. We will also look at comparing participation in our hospital versus our ambulatory care setting.

Poster Number: 138

Time: Sunday, May 15, 2016, 2:00 PM – 2:55 PM

Focus on the Forest, Not the Trees: A Checklist for Planning Chapter Meetings

Lisa K. Traditi, AHIP, Head of Education and Reference, Health Sciences Library, University of Colorado Health Sciences Library/Education & Reference, Aurora, CO; Jon Crossno, AHIP, Cataloging & Metadata Librarian, Health Sciences Digital Library & Learning Center, University of Texas Southwestern Medical Center, Dallas, TX

Objectives:
After successfully planning Quint*Essential: Convergence and Collaboration, a meeting of five MLA chapters, the authors share the lessons they learned from their experience

Methods:
Held Oct.12-16, 2014, Quint*Essental was a joint meeting of the

Midcontinental Chapter of MLA(MCMLA),
Medical Library Group of Southern California & Arizona(MLGSCA),
Northern California & Nevada Medical Library Group(NCNMLG),
Pacific Northwest Chapter of MLA(PNCMLA), and
South Central Chapter of MLA(SCCMLA). After reviewing successes and failures of their multi-chapter meeting, the authors used SurveyMonkey to gather the opinions and advice of other multiple chapter meeting planners. The authors then identified key activities and time-sensitive tasks necessary to planning such a meeting.

Results: From this amalgam of information, they created a checklist designed to help future planners, whether for individual or multiple chapter meetings. This checklist also includes a recommended timeline for when essential milestones should be reached.

Conclusions: Holding a multi-chapter meeting, while a daunting task, can be beneficial to chapters and attendees. A vetted planning checklist, along with strong communication, skills with shared decision-making, and effective record-keeping are key components for success.

Poster Number: 141

Time: Sunday, May 15, 2016, 2:00 PM – 2:55 PM

Osteopathic Research: Digging for Morsels in Mainstream Medical Resources

Lori Fitterling, Digital Services/Reference Librarian, D'Angelo Library, D'Angelo Library, Kansas City, MO; Jean Sidwell, Library Branch Director & KCOM Librarian Liaison, A.T. Still Memorial Library , A. T. Still Memorial Library, Kirksville, MO

Objectives: A unified accreditation system has aligned osteopathic and allopathic medical education but librarians continue to recognize the absence of osteopathic subjects within mainstream medical resources. By gathering information about osteopathic libraries and their search experiences, this poster presents strategies and rubrics for osteopathic research to highlight the differences between osteopathic and allopathic literature searching.

Methods: Information was gathered in three ways: 1) A ten question survey was drafted and sent to thirty osteopathic university libraries; 2) PubMed was searched for osteopathic topics using key words and MeSH headings; 3) two osteopathic reference librarians from different universities compiled statistics on osteopathic reference literature searches, utilizing varied search strategies. Survey questions addressed concerns about the small amount of osteopathic information included in PubMed, how to locate international osteopathic literature sources, the use of OSTMED.DR database, and how libraries present osteopathic information resources to users on library websites. Statistics were also obtained from the Still National Osteopathic Museum citing the number of foreign and United States researchers who request osteopathic information. All data was compiled and analyzed.

Results

Results from the survey show that in 2015, 24% of reference questions at osteopathic libraries have addressed osteopathic topics and 77% of librarians surveyed found it hard to locate osteopathic information (83% found it hard to locate *international* osteopathic information). This indicates a general problem in finding osteopathic information. And while 50% of libraries subscribe to OSTMED.DR (an osteopathic medicine digital database created from a partnership between Edward Via College of Osteopathic Medicine (VCOM) and VTLS, Inc. and endorsed by the AOA and AACOM), only 33% would use it as a primary resource. Fifty percent of respondents would consult a chiropractic database to find osteopathic information indicating the use of alternative resources. Only two respondents indicated that they have worked on a systematic review of an osteopathic topic, indicating a lack of osteopathic systematic reviews. Only 61% of libraries surveyed have finding aids for osteopathic information on their website and several databases were cited as

alternative resources for finding osteopathic information.

Conclusions

Librarians face challenges when researching osteopathic topics. Results of this study show that there is a need for primary osteopathic resources, finding aids, and for osteopathic systematic research reviews to be included in the medical literature. The study also offers the search experiences, search strategies, and database recommendations for osteopathic reference searching.

Poster Number: 144

Time: Sunday, May 15, 2016, 2:00 PM – 2:55 PM

Community Collaborations to Establish Consumer Health Information Services for Underserved Populations

Margaret Ansell, Nursing and Consumer Health Liaison Librarian, University of Florida, Health Science Center Libraries, Gainesville, FL; Linda B. Cottler, Dean's Professor and Chair, Department of Epidemiology, University of Florida, College of Public Health and Health Professions and College of Medicine, Gainesville, FL; Vicki Piazza, HealthStreet Manager, University of Florida, College of Medicine, Gainesville, FL; Michele R. Tennant, AHIP, UNIVERSITY LIBRARIAN, Health Science Center Library, Biomedical and Health Information Services, Health Science Center Libraries, Gainesville, FL

Objectives: The consumer health librarian at an academic health science library collaborated with a community outreach center to launch a coordinated program of consumer health services to community members with limited access to healthcare, with the goal of increasing their knowledge of health information resources. The evaluation built into this pilot project provides valuable feedback about the project's feasibility and effectiveness.

Methods: The community center, established by the university's department of epidemiology, is designed to connect underserved populations to health providers, social services, and research studies conducted by the university, with the goals of improving the health of these populations, increasing their involvement with and knowledge of the research process, and reducing the population bias of research at the university. With this particular setting in mind, the pilot project, using funding from an internal university-wide library grant, performed a needs assessment, hosted training workshops for center staff on online consumer health resources, and purchased a collection of print consumer health monographs to be housed at the center. Referrals to print materials or online resources were implemented by the staff as part of their workflow. The needs assessment was used to customize services, and follow up evaluation will determine the project's effectiveness.

Poster Number: 147

Time: Sunday, May 15, 2016, 2:00 PM – 2:55 PM

Managing the Virtual Hospital Library in the Middle East Based on a US Hospital Library Model

Marian T. Simonson, AHIP, Systems Medical Librarian, Floyd D. Loop Alumni Library, Cleveland Clinic, Cleveland, OH; Manish Kohli, Chief, Medical Informatics, Cleveland Clinic Abu Dhabi, Cleveland Clinic Abu Dhabi, Abu Dhabi, United Arab Emirates; Michelle Kraft, AHIP, Senior Librarian, Cleveland Clinic Alumni Library, Cleveland Clinic Library, Cleveland, OH; Naeema Rabia, Project Assistant, Cleveland Clinic Abu Dhabi, Cleveland Clinic Abu Dhabi, Abu Dhabi, United Arab Emirates; Gretchen Hallerberg, AHIP, Library Director, Cleveland Clinic Alumni Library, Cleveland, OH

Objective:

The Cleveland Clinic partnered with Abu Dhabi's government to build a new hospital in the United Arab Emirates. Library services for Cleveland Clinic Abu Dhabi (CCAD) are managed remotely by Cleveland Clinic's Floyd D. Loop Alumni Library in Cleveland, Ohio. This poster discusses the planning, implementation, and experiences in setting up the library and its first year of operation.

Methods:

Discussion of library services for the new hospital began in 2008. The original plan was to build a library with space for two staff, meeting and training rooms, and study areas. Library resources were planned to be virtual from the outset with no space for collections. CCAD received initial bids for library resources from Middle East vendors in 2012-13. During January-March 2014, plans changed from on-site to remote management of the library by Alumni Library staff in the US. An initial list of resources was sent to vendors for pricing. Final selection and licensing of resources were completed by July 2014 along with the library's website. Target date for library implementation was August 2014, the start of physician and nurse onboarding.

Results:

Key challenges during the first year of operation included vendor negotiations, especially separating CCAD from the Alumni Library; educating CCAD caregivers regarding library resources and policies; managing workflows across different time zones and work weeks; and designing an intuitive website for a multilingual workforce. The hospital opened for patients in March 2015 and celebrated it first year of operation in March 2016. Usage statistics were generated periodically and resource selection modified accordingly. Key milestones that enhanced the usability of resources included the customization of PubMed for Linkout and Outside Tool; website redesign; and the implementation of EZProxy for off campus access.

Conclusion:

Although the first year of operation was successful, opportunities for growth are many, such as: training the Project Assistant in the Middle East on the use of library resources; developing customized training videos of how to use library resources for caregivers; streamlining workflows between CCAD and the Alumni Library; and developing strategies to increase usage statistics for existing resources.

Poster Number: 152

Time: Sunday, May 15, 2016, 2:00 PM – 2:55 PM

Resource Format Preferences Across the Medical Curriculum: Insights from a Cross-Sectional Survey

Maureen Knapp, AHIP, Research Support & Education Librarian III, Rudolph Matas Library of the Health Sciences, Tulane University - Matas Library, New Orleans, LA; Keith Pickett, Research Support, Web Resources and Education Librarian, Rudolph Matas Library of the Health Sciences, Rudolph Matas Library of the Health Sciences, New Orleans, LA

Objectives: It is no new development that academic health sciences libraries are moving towards more and more electronic resources, but what do formats to current medical students and residents really prefer? Does preference change over the course of the medical curriculum?

Methods: A five question multiple choice survey with optional comments was developed in June 2014. Survey received IRB approval July 2014. Each question had three possible answers: Print, Electronic, or No Preference. Questions included: preference for general books, required medical textbooks, works over 10 pages, works under 10 pages, sharing an item critical to patient care with a colleague. The same survey was distributed to first year medical students (T1s, n=184), third-year medical students entering clinical clerkships (T3s, n=212), and incoming residents (R, n=140). Data was collected from September 2014 – July 2015, tallied manually, and transferred to Excel. Qualitative responses were transcribed into Word, hand-sorted into categories, and transferred to Excel. Data was compared across the groups to determine preferences and trends from the first year of medical school through residency. This abstract reports on the qualitative responses.

Results: Overall response rate was 89% (n=478/536). A majority of respondents preferred to read general books and textbooks in print regardless of where they are in the curriculum (T1=80.33%, T3=68.42%, R=56.19%). Qualitative responses cited for print preferences included eyestrain/comfort, annotation, personal habit, and tactile experience. However, a majority of respondents also preferred electronic for works less than 10 pages (T1=54.64%, T3=63.68%, R=55.24%). Preference for electronic increased when sharing with a colleague (T1=38.8%, T3=42.11%, R=58.10%). Qualitative reasons for electronic preference included ease of sharing or sending, accessibility, searchability, portability, speed, and the perception that electronic materials are less likely to be lost.

Conclusions: The data collected suggests two conclusions. First, demand for print is high and remains high throughout the medical curriculum. Second, preferences for electronic works increases over time. Electronic articles and

book chapters seem to be most preferred in a clinical setting as opposed to a classroom setting. For collection development purposes, collections should provide print copies of required textbooks to cater to students and incoming residents desire for print and electronic.

Poster Number: 155

Time: Sunday, May 15, 2016, 2:00 PM – 2:55 PM

Bibliometric Analysis and Funding Success to Evaluate an Organization's Research Grant Decisions

Me-Linh Le, Health Sciences Centre Librarian, Neil John Maclean Health Sciences Library, Neil John Maclean Health Sciences Library, Winnipeg, MB, Canada; Gregory W. Hammond, Executive Director, Manitoba Medical Services Foundation, Manitoba Medical Services Foundation, Winnipeg, MB, Canada; Tannis Novotny, Executive Administration, Manitoba Medical Service Foundation, Manitoba Medical Service Foundation, Winnipeg, MB, Canada; Grant Pierce, Executive Director of Research, St. Boniface Hospital, Professor of Physiology and Pharmacy, Winnipeg, MB, Canada; John Wade, FRCPC, Professor Emeritus and Dean Emeritus, Faculty of Health Sciences, University of Manitoba, College of Medicine, Winnipeg, MB, Canada

Objectives: The Manitoba Medical Services Foundation (MMSF), a non-profit medical foundation that has provided nearly $20 million to support and fund research since 1974, sought to evaluate the subsequent output of both its successful and unsuccessful operating grant applicants. The foundation, which focuses on supporting new researchers, worked with the Library to determine whether its grant review process was successful in selecting the best candidates from 2008 to the 2012 competitions.

Methods: Using information up to 2014 for the five years of grants, which totaled $1,912,300 in funding, an analysis was first completed for all successful and unsuccessful grant applications. The analysis focused on two areas: publication history and funding history. Scopus – one of the largest databases in the world and a resource committed to eliminating author identification issues – was employed to determine the number of published articles and the h-index for each researcher. The funding databases of the three largest federal granting agencies in the country were searched to determine whether a researcher had subsequently obtained other grants. The bibliometric and funding data were statistically analyzed to assess the impact of a researcher's initial grant result on their future publication output and funding success, as well as the local multiplier effect for the granting organization.

Results: Statistical analyses clearly demonstrated that those researchers who received funding from the MMSF went on to have greater academic productivity than unsuccessful candidates. Specifically, successful candidates had a greater number of publications, a higher h-index, larger amount of funding from the major Canadian research granting organizations, and greater odds of receiving funds as either co-investigators or lead principal investigators. Analyses also showed that successful applicants were ultimately very successful in bringing

future external funding back to the province, with a local multiplier effect of 10:1 (i.e., for every $1 spent on Manitoba-based researchers, $10 returns to the community).

Conclusions: This research demonstrated that the current process used by MMSF is successful at selecting individuals who subsequently go on to become high-performing researchers. These researchers are ultimately more productive and obtain more funding than those individuals that are not selected. Furthermore, this project demonstrates a new way for Libraries to use metrics to assist organizations or institutions as they are called upon to demonstrate their value and impact on the community.

Poster Number: 158

Time: Sunday, May 15, 2016, 2:00 PM – 2:55 PM

Multistate Librarian Journal Club: Building Community, Research Capacity, and Inquiry

Melissa L. Rethlefsen, AHIP, Deputy Director / Associate Librarian, Spencer S. Eccles Health Sciences Library, Spencer S Eccles Health Sciences Library, Salt Lake City, UT; Tara Brigham, Medical Librarian, Mayo Clinic Libraries, Winn-Dixie Foundation Medical Library, Jacksonville, FL; Matt Hoy, Supervisor, MCHS – Eau Claire, MCHS – Eau Claire Medical Library, Eau Claire, WI; Ann Farrell, Librarian V, Mayo Clinic Libraries, Plummer Library, Rochester, MN; Leah Osterhaus Trzasko, Librarian, Health Science Library, Plummer Library / Public Services, Rochester, MN

Objectives: To describe the development and maturation of a multi-institutional, cross-country journal club for librarians and information specialists

Methods: Beginning in May 2013, a group of interested librarians at a single institution with branches in four states formed a lunchtime journal club. The club, which was open to all library staff within the institution, was facilitated by a single librarian and met monthly using Google Hangouts. This club expanded to another institution in 2014 and opened up to others in 2015. Each month, 1-2 participants present an article selected by the group or the facilitator; the entire club joins in the critical appraisal process after hearing a brief discussion of a research design or statistical concept related to the article. Articles were chosen for their relevance to medical libraries, unique and applicable methodologies, and the strength of the research design. In addition, the group invited several authors of appraised articles to discuss their research methodology in more depth.

Results: The journal club has met most months since May 2013. Appraised articles ranged in topic and discipline, covering articles published by medical librarians, academic librarians, physicians, and informaticians. Basic research concepts covered included differences between quantitative and qualitative research, p values, cross-sectional study design, and other topics of interest to group members. 18 individuals have participated; 13 are current active participants. 3 external authors and 1 guest speaker have contributed to journal club sessions. Video quality varies, but attendees can always hear each other clearly, and meetings can easily be created and updated.

Conclusions: The ease of use of the Google Hangouts format has allowed the club members to connect across multiple time zones with minimal technical problems. Since beginning journal club, several members have published and presented their own research more frequently. Librarian and information

specialist journal clubs can highlight advances in the field, increase capacity for evidence-based library practice, and build community.

Poster Number: 160

Time: Sunday, May 15, 2016, 2:00 PM – 2:55 PM

How Systematic Are They? Outing High-Quality Lesbian, Gay, Bisexual, Transgender (LGBT) Health Systematic Reviews and Meta-Analyses

Mellanye Lackey, Associate Director of Education & Research, University of Utah, Eccles Health Sciences Library, Salt Lake City, UT; Joseph G. L. Lee, Assistant Professor, East Carolina University, Department of Health Education & Promotion, Greenville, NC; Thomas Ylioja, PhD Student, University of Pittsburgh, School of Social Work, Pittsburgh, PA

Objectives: To determine if systematic reviews and meta-analyses on LGBT Health Issues meet reporting requirements for reviews as established by Cochrane Reviews and the Prisma Statement.
To bring attention to optimal practices for systematic reviews, focusing on search term inclusion and independent coding by at least two reviewers.

Methods: Researchers conducted a systematic review of systematic reviews and meta-analyses of LGBT health issues in three databases, PubMed, PsycInfo and Embase. Inclusion criteria and exclusion criteria guided the reviewers as they selected full text articles for review, and independently coded and extracted data for analysis. Articles were included if they had at least two, independent reviewers coding, discussed at least one domain of sexual orientation, and documented the search terms used in their reviews. Exclusion criteria included AIDS/HIV articles, grey literature, articles about the impact of gay parents on children, the treatment of homosexuality or transgender-related issues as a disease, or the origins of homosexuality or transgender-related issues. No geographic, date or language limits were applied.

Poster Number: 163

Time: Sunday, May 15, 2016, 2:00 PM – 2:55 PM

Building Medicine at the University of Michigan: Past and Present

Merle Rosenzweig, Informationist; Anne Perorazio, Media Developer; Elise Wescom, Art and Design Student; Chase Masters, Enabling Technologies Informationist; Scott Hanley, Alumni; Taubman Health Sciences Library, Ann Arbor, MI

Objectives: The University of Michigan Medical School (UMMS) opened its doors in 1850. It had many firsts among medical schools in the U.S: first to own and operate its own hospital; to admit women; to teach science-based medicine; to introduce the modern medical curriculum and clinical clerkships. The objective is to tell storied history of medicine at U-M through its architecture.

Methods: Throughout time, people have used visual devices to capture stories. Architecture forms a visual, spatial link between the past, present, and future, becoming a point in the timeline of a place. In this poster we have chosen to tell the story of medicine at the University of Michigan through the many buildings that connect to it’s past, present and future. To obtain images of the older buildings that tell the story of the past we searched the Bentley Historical Library that serves as the official archives of the University. After selecting the pictures to include in the poster we found the locations on the campus on which those buildings stood and photographed the buildings that are now there.

Results: The history of medicine at the University of Michigan has been told in books and pictures. We have captured the “Building of Medicine" at the University of Michigan in a unique and visual way.

Conclusions: This poster aims tell the story of medicine at the University of Michigan as an architectural history. By juxtaposing the images of buildings from the past with those of the present we are, we believe, telling the story of medicine at U-M in a way that has never been done previously.

Poster Number: 165

Time: Sunday, May 15, 2016, 2:00 PM – 2:55 PM

Incorporating User Data into the Design and Creation of a Mobile-Compliant Page to Deliver Content to Clinicians and Trainees at an Academic Health Sciences Center

Mia S. White, AHIP, Medical Education and Technologies Informationist; Amy Allison, AHIP, Associate Director; Woodruff Health Sciences Center Library, Woodruff Health Sciences Center Library, Atlanta, GA

Objectives: From 2012 to 2015, various technological changes were implemented university wide at an academic health sciences center which impacted access by clinical faculty to library resources, specifically to ejournals and point of care tools. To streamline and to accommodate mobile platforms, the presentation of content most useful to clinical faculty and trainees was redesigned for easier access.

Methods: With the implementation of a new authentication system to access online resources, clinical faculty and trainees lost the ability to use third-party applications, particularly for table of contents alerts. Based on observations of how the users accessed and used journal content on their mobile devices and some of the problems they encountered, informationists explored options for improving ease of access to library-licensed content. Requirements for a webpage to deliver this content were defined. Informationists then collected usage data on journals and databases relevant to clinical faculty trainees and interviewed them to determine the most frequently used resources. Using the institution's mobile-compliant content management system, an informationist created a prototype page to address identified needs. The page was piloted with a group of faculty, residents, and students who were invited to use the page and to provide feedback via SurveyMonkey.

Results: Eight individuals in the target audience provided feedback on their experience with accessing e-resources with their mobile device. Seven (87.5%) stated that they would access the pilot site again. Suggestions from respondents on how to improve the site included maintaining a small selection of resources, preferences for links to mobile sites for the library's e-resources rather than links to apps that have to be installed, and including links to the teaching hospitals' EMR applications.

Conclusions:
The pilot study indicated that faculty, residents, and students are interested in a page designed for mobile access to key clinical journals and other information

resources. The suggestions for improving the pilot page will be investigated for implementation and the authors will explore expanding the program to other disciplines. The low response rate indicates that an online survey may not be the most effective means of gathering feedback on the library website from this institution's faculty and trainees in the clinical setting. Further investigation into feedback methods with higher response rates may be useful.

Poster Number: 168

Time: Sunday, May 15, 2016, 2:00 PM – 2:55 PM

Reflective Practice for Professional Development Through a Collaborative Systematic Review Project

Misa Mi, AHIP, Associate Professor and Medical Librarian, Medical Library, Medical Library, Rochester, MI; Jie Li, Asst. Director for Collection Management, Charles M. Baugh Biomedical Library, Biomedical Library, North Mobile, AL; Lin Wu, AHIP, Instructional Associate Professor and Pharmacy & South Health Science Center Campus Librarian, Texas A&M University, Medical Sciences Library, Kingsville, TX; Wendy Wu, Information Services Librarian, Shiffman Medical Library, Shiffman Medical Library, Detroit, MI; Yingting Zhang, AHIP, Information & Education Librarian, Robert Wood Johnson Library of the Health Sciences, Rutgers University, New Brunswick, NJ

Objectives: Learning is most effective when it takes place in a context as a collaborative rather than an isolated activity. Experience serves as the basis for learning and cannot take place without reflection. We formed a team of 6 health sciences librarians to conduct a collaborative systematic review (SR) project. Upon completion of the project, we reflected on our involvement in the project for reflective practice as a process of professional development. Grounded in Donald Schön's reflective practice as a conceptual framework, the research study is to investigate the nature, impact of our participation in the project, how we came to understand our behavior, and how we developed an awareness of our own actions and the effects of our participation on our professional development. The goal is to understand what was involved in conducting a SR so that we can better understand how researchers would undertake a SR and hence serve them better. The knowledge of the variations in how librarian participants understood and perceived the process of conducting a SR will help them and other health sciences librarians improve the process and SR-related initiatives and services. The knowledge will also help establish a close link between professional development, applied research, and reflective practice of health sciences librarianship.

Methods: A phenomenographic approach, which provides a lens through which to explore different understandings of a phenomenon, was used to explore the variations in which we experienced with the SR project, the multiple ways in which we came to understand SR, and the different roles we took in the process. Five health sciences librarians across different library settings participated in the study. Interviews were conducted with a structured questionnaire to prompt participants to reflect on various experiences in the SR project. Participants' responses will be analyzed with a qualitative approach to explore and capture a range of possible ways of conceptualizing and understanding what it took to

undertake a SR. Responses will be classified into conceptual categories. To increase the trustworthiness of the research, the participants will be involved in the phenomenological analysis for the purpose of investigator triangulation.

Poster Number: 171

Time: Sunday, May 15, 2016, 2:00 PM – 2:55 PM

The Librarian's Role on a Patient Family Advisory Council

Nancy Goodwin, AHIP, Director, Middlesex Hospital, Library & Knowledge Services, Middletown, CT

Objectives: This presentation describes how the librarian at a community hospital helped establish the Patient Family Advisory Council (PFAC) and facilitated projects by its members.

Methods: A PFAC is composed of former patients or their family members who experienced services and care in a health system. It provides valuable input to hospitals focused on patient- and family-centered care. This poster highlights how the librarian helped to plan and establish a PFAC at a community hospital. The creation of the PFAC, its mission and goals, as well as the projects the librarian facilitated are outlined. These projects include creation of three checklists for patient use, complete revision of the hospital's admission booklet, recommendations on signage at the outpatient center and an intranet site explaining the PFAC to hospital staff. The librarian educated the PFAC members on the concepts of health literacy and provided expertise when the council's projects had a health literacy component.

Results: The librarian's participation in the creation and ongoing work of her hospital's PFAC helped the group to reach its annual goals.

Conclusions: The librarian can play a valuable role on the hospital's Patient Family Advisory Council.

Poster Number: 175

Time: Sunday, May 15, 2016, 2:00 PM – 2:55 PM

Optimizing Patient Care Curriculum: Bridging Three Domains of Knowledge for a Fully Integrated, Longitudinal Curricular Thread

Nandita S. Mani, AHIP, Assistant Director, Academic & Clinical Engagement, Taubman Health Sciences Library, Taubman Health Sciences Library, Ann Arbor, MI

Objectives: As U-M Medical School embarks on a major, comprehensive curricular revision, we have significantly modified and expanded curriculum components related to medical decision making. Previously, instruction in this area was provided over the first three year years of medical school in different courses and clerkships via a series of curricular experiences that combined lectures, small group problem-solving sessions and computer informatics workshops. While the former approach was successful by some key measures – e.g., students' scores on biostatistics and clinical epidemiology components of national licensure exams were well above the national average – the previous curriculum architecture limited the integrated longitudinal approach that is needed in order to create habits of inquiry and reflection that are required for lifelong learning. Using a conceptual model adapted from Hoffman et al. (2014), we have set the goal of achieving a fully-integrated curricular thread that encompasses a comprehensive set of inter-related skills: (1) Evidence-Based Medicine (including clinical informatics), (2) Patient-Centered Communication Skills, and (3) Shared Decision-Making, which we have called the Optimizing Patient Care Curriculum (OPCC).

Methods: An interdisciplinary team with combined expertise in the three OPCC foci developed an inaugural curriculum for first-year learners. Learning objectives have been developed, and experiences encompassing each of the three OPCC domains, with integration points in the following curricular areas: M1 Launch (expanded first-year orientation during the first weeks of medical school), Foundations in Molecular Medicine (first sequence of the M1 year, with a focus on biochemistry and genetics), Chief Concern Course (a longitudinal course with a focus on clinical reasoning), and Initial Clinical Experience (a longitudinal first-year clinical experience with a focus on patients, healthcare teams, and healthcare systems). Through intentional planning and collaboration, faculty from OPCC and the aforementioned curricular areas have been building a truly integrated curricular thread. OPCC elements which are being integrated into curricular components are being designed by incorporating experiential learning and critical reflection.

Results: Student feedback on initial OPCC sessions has been favorable. Sessions during M1 Launch yielded the following evaluation outcomes: 91% of students Agree or Strongly Agree that "Overall, the OPCC activities increased my understanding of patient-centered communication"; 81% of students Agree or Strongly Agree that "Overall, the OPCC activities increased my understanding of clinical informatics"; 89% of students Agree or Strongly Agree that "Overall, the OPCC activities increased my understanding of shared medical decision-making."

Conclusions: Compared to developing a stand-alone curriculum, a primary challenge when integrating a curricular thread such as OPCC lies in the additional effort required to fully integrate material from multiple domains in a manner that builds sustainable habits, opportunities for inquiry, and also touches all relevant elements of the new curriculum in ways that are synergistic. By assembling an interdisciplinary team to develop the curriculum, and by intentionally and proactively seeking and achieving points of collaborative integration with other curricular components, we were able to initiate a fully-integrated, longitudinal curricular thread. Next steps include summative and program evaluation surveys and reviews.

Poster Number: 178

Time: Sunday, May 15, 2016, 2:00 PM – 2:55 PM

Creating a Community of Preceptors

Nicole Mitchell, Information Technology Librarian and Associate Professor of Library & Information Sciences, Alabama College of Osteopathic Medicine, Library/Learning Resource Center, Dothan, AL; Lisa Ennis, Information Technology Librarian and Professor of Library & Information Sciences, Alabama College of Osteopathic Medicine, Library/Learning Resource Center, Dothan, AL; Amanda Gant, Clinical Sciences Coordinator, Alabama College of Osteopathic Medicine, Clinical Sciences, Dothan, AL; Stephen Miller, Associate Dean of Clinical Sciences/Associate Professor of Family Medicine and Osteopathic Principles and Practice, Alabama College of Osteopathic Medicine, Clinical Sciences, Dothan, AL

Objectives: To describe and report on the resources, outreach, and training opportunities developed for community preceptors and third and fourth year osteopathic medical students.

Methods: In support of their service, preceptors, over 800 physicians, receive access to all library resources. Librarians created an online "portal" for preceptors, participated in on-site visits and presentations, and will travel this fall to conduct faculty development sessions for preceptors on using EBM resources at the point of care.

Results: The Preceptors & Students guide was launched during the first "road show" orientation sessions for preceptors in Spring 2015. This guide is essentially a one-stop shop for preceptors looking for information on accessing library resources and evidence-based medicine as well as resources for preceptor support and development such as effective teaching strategies, tips for orienting the student to the clerkship, teaching in a clinical setting, assessing student ability, and more. In addition to resources geared toward preceptors, the guide also includes a section of resources for students and core site coordinators.

Conclusions: This fall librarians will survey preceptors on a variety of topics and will use the data to tailor our upcoming fall training sessions, specifically in teaching EBM and the use of EBM resources. The guide will continue to grow and develop as we receive feedback from preceptors and students and as we conduct more preceptor training sessions throughout the fall of 2015. We will present data from the guide as well as surveys and feedback from the in-person faculty development sessions.

Poster Number: 180

Time: Sunday, May 15, 2016, 2:00 PM – 2:55 PM

Librarian Participation in "Hands-On" Evidence-Based Medicine Rounds with Pediatric Residents

Pamela Hargwood, AHIP, Information and Education Librarian, Rutgers, The State University of New Jersey, Robert Wood Johnson Library of the Health Sciences, New Brunswick, NJ; Elizabeth Goodman, M.D., Assistant Professor of Pediatrics, Rutgers, The State University of New Jersey, Robert Wood Johnson Medical School, New Brunswick, NJ; George Kaliyadan, M.D., Clinical Instructor of Pediatrics, Rutgers, The State University of New Jersey, Robert Wood Johnson Medical School, New Brunswick, NJ

Objectives: To describe the successful implementation of a project to increase the ability of pediatric residents at a medical school to practice evidence based medicine (EBM) in real time; examine if patient care is supported or changed based on the resident's findings; and discuss the role of the medical school librarian in this process.

Methods: In July 2014, the Department of Pediatrics implemented "hands-on " inpatient EBM rounds to help pediatric residents learn how to better formulate clinical questions, locate relevant literature, and apply their EBM findings to patient care in "real time". These hour long rounds take place bi-weekly and are held on inpatient wards. All pediatric residents rotating on the inpatient team are invited to attend the EBM rounds. The medial librarian and two pediatric faculty members assist the residents with formulating clinical questions based on the inpatients they are currently following and searching for an answer to their question. The residents summarize their search and findings using a PICO worksheet. The following day the residents are evaluated on whether or not they presented their EBM findings on attending rounds and if patient care was supported or changed based on these findings.

Poster Number: 183

Time: Sunday, May 15, 2016, 2:00 PM – 2:55 PM

Engaging Diverse Communities in Cancer Conversations Through Creation of Structure and Metadata within Twitter

Patricia F. Anderson, Emerging Technologies Informationist, Taubman Health Sciences Library, Taubman Library, University of Michigan, Ann Arbor, MI; Matthew S. Katz, Doctor, Lowell General Hospital, Lowell General Hospital, Lowell, MA

Objectives: Intending to develop an online space engaging to both clinicians and patients, we created a cancer tag ontology for Twitter. The goal was to foster boundary-spanning between diverse communities through partnership, to build off the successes and best practices of existing Twitter cancer hashtag communities, and to encourage self-managed communities for information quality, in the context of appropriate metadata practices.

Methods: Based upon two de novo hashtags, #bcsm and #btsm, an organized system of hashtags, the cancer tag ontology (CTO) was designed in July 2013 for online use. Metadata criteria applied included factors such as length, standard formatting, adherence to alphabetical sorting for related subtopics, and similar principles. All tweets were archived in the Symplur Healthcare Hashtag Project for later analysis. We conducted a retrospective study of 25 hashtags used on Twitter April 2011 – September 2014 using data from Symplur, LLC. We classified up to 100 most active users of each hashtag as follows: patient; doctor; non-doctor health care professional (HCP); individual NOS (I); healthcare organization (HCO), other organization (OO); or spam. Tweet activity was analyzed quarterly for all tags.

Results: The study included 531,765 tweets from 77,454 users. The original hashtags (#bcsm, #btsm) had the most use with 249,312 and 110,465 tweets. Other tags began use in Q3 2013, with the most active new tags being those with Twitter chats: #ayacsm; #gyncsm; #lcsm; #mmsm; #pancsm. These accounted for 93% of measured Twitter activity. User participation breakdown showed 11% patients, 20% doctors, 3% HCP, 32% I, 30% HCO, 1% OO, and 3% spam, with patients most active with ~46 tweets each. All NIH Comprehensive Cancer Centers use the tag ontology. Wide adoption has spawned three new tag ontologies: oncology, radiology, urology.

Conclusions: Typical hashtag adoption patterns have shown a reluctance to adopt prescribed hashtags outside of formal events. The success of the tag ontology shows the desire for engagement and partnership among the target

communities. We have demonstrated the feasibility and growth of organized, cancer-specific hashtags on Twitter used by a variety of stakeholders in cancer care. Use of the CTO indicates potential value of online interaction. Further study is needed to determine whether the CTO has any impact on access, outcomes, information quality, as a model for other areas of medicine, or as a resource for future research.

Poster Number: 186

Time: Sunday, May 15, 2016, 2:00 PM – 2:55 PM

Piecing Together Public Access Compliance Issues: Analysis of One Institution's Challenges

Patricia L. Thibodeau, AHIP, FMLA, Associate Dean for Library Services & Archives, Medical Center Library & Archives, Medical Center Library & Archives, Durham, NC; Emily Mazure, AHIP, Biomedical Research Liaison Librarian, Medical Center Library & Archives, Duke University, Durham, NC; Emma Heet, Associate Director Collection Services, Duke University, Medical Center Library & Archives, Durham, NC; Virginia M. Carden, AHIP, Administrative Research Librarian, Duke University, Medical Center Library, Durham, NC; Beverly Murphy, AHIP, FMLA, Assistant Director, Communications and Web Content Management; Hosptial Nursing Liaison, Duke University, Medical Center Library & Archives, Durham, NC; Megan G. Van Noord, Research & Education Librarian, Duke University Medical Center Library, Duke Medical Center Library & Archives, Durham, NC

Objectives: Evaluate the articles that remain non-compliant with the NIH Public Access Policy to determine any trends or issues that could be addressed systematically.

Methods: Even though the institution has achieved a high level of compliance, additional strategies need to be identified to resolve outstanding publications. Based on a non-compliance report from the Public Access Compliance Monitor, the Library was able to sort the data and identify trends. Data was collected on the publication year of publications, the status of publications within the manuscript submission system, the total number of principal investigators, the degree of non-compliance among those investigators, and units that may have higher levels of non-compliance. Journal and therefore publisher information was also evaluated for non-compliant publications. The data will be shared with research offices also concerned with compliance issues for the university and used to develop strategies for achieving better compliance.

Results:

It is clear from the data and discussions that timely reminders of non-compliant publications are needed and appreciated. In addition, monitoring progress report due dates and generating additional reminders works well too. However, based on requests from researchers and their staff, the completion of biosketches and progress reports still remain a major trigger for addressing compliance issues. Researchers still ignore requests for initial and final

approvals. A critical point in the research process is the acceptance of the manuscript and immediate handling of the submission. Researchers want someone to handle this for them. The Library is now exploring a manuscript repository approach that would allow better central

ized
tracking and submission. Compliance for older publications (2008 to 2011) still remains a challenge.

Poster Number: 188

Time: Sunday, May 15, 2016, 2:00 PM – 2:55 PM

Field Study of the Evidence-Based Dentistry Activity of Predoctoral Students and Preceptors at Off-Campus Dental Offices

Peter Shipman, Dental Medicine and Cancer Librarian, Research and Education, Augusta University Robert B. Greenblatt, MD Library, Augusta, GA; Julie Zadinsky, Assistant Dean for Research, Augusta University, College of Nursing, Augusta, GA; Tasha Wyatt, Educational Researcher, Educational Innovation Institute, Augusta University, Medical College of Georgia, Augusta, GA

Objectives: To measure evidence-based dentistry (EBD) activity between predoctoral dental students and their dental preceptors to improve dental school curriculum and training.

Methods: Predoctoral dental students (PDS) learn and apply evidence-based dentistry (EBD) principles and practices during training in an accredited dental school clinical environment. PDS are also required to practice briefly at off-campus dental practices to learn how to treat patients and improve chair skills under a dental preceptor's supervision. PDS and preceptors will be interviewed independently of each other by a medical librarian to recall EBD activity during patient care occurring in the off-campus setting. Both will be asked to recall EBD behaviors that address patient-centered needs and preferences, influence clinical decision making about patient diagnoses and treatment plans, and frequency of the application of scientific research to clinical problem solving. Interview transcripts will be transcribed and analyzed by an educational researcher for themes or trends in EBD activity in the off-campus settings.

Results: My results are not complete yet.

Conclusions: My results are not complete yet.

Poster Number: 191

Time: Sunday, May 15, 2016, 2:00 PM – 2:55 PM

Getting Your Foot in the Door: Implementing Embedded Office Hours

Rachel R. Helbing, AHIP, Liaison Librarian, Texas Medical Center LIbrary, Client Relationship Management Department, Houston, TX

Objectives: Securing library engagement from a school that is physically located across campus is challenging. In response, the nursing liaison devised and implemented a program to provide office hours in the nursing school building. The main objective of the program is to increase the frequency of contact with administration, faculty, and students, resulting in more awareness and use of library services.

Methods: The librarian leveraged social capital built in the first six months of her nursing liaison assignment by emailing a variety of connections to seek assistance with securing a space in the nursing school building. Enthusiastic about the idea, the Dean and Associate Dean of Research provided a cubicle, and later an office, for the librarian's use. Office hours and consultations are now held there regularly. Promotion of the new service has included fliers, social media, verbal announcements during orientations and classes, and notices sent to several nursing school email lists by a faculty champion. Interactions that take place during the office hours are specifically coded in the reference analytics program to allow accurate evaluation of the service. Patrons who receive a library consultation are also given the opportunity to complete an online consultation evaluation.

Results: During the time period of August 1, 2015 through January 31, 2016, office hours were held for a total of 88 hours. During those hours, there were 20 patron interactions. Outside of those hours, the librarian's office was used for 21 consultations.

Conclusions: Awareness and utilization of library services have increased with the implementation of on-site office hours. Proximity to a librarian drives patron willingness to seek help.

Poster Number: 194

Time: Sunday, May 15, 2016, 2:00 PM – 2:55 PM

Becoming Part of the Bigger Picture: Improving Partnerships Across Campus to Provide Improved Interdisciplinary Access to Institutionally Licensed Resources

Rebecca Harrington, AHIP, Electronic Resources Librarian, Maguire Medical Library, Florida State University College of Medicine, Tallahassee, FL

Objectives: Like many medical school libraries, ours operates under the administration of the College of Medicine, not the University Libraries. With this decentralized system, the medical library had maintained separate electronic resources lists, remote user authentication, and discovery tools. To improve access for our users, we have moved to a unified plan for electronic resource delivery, building stronger campus library collaborations.

Methods: Maintaining parallel systems is costly to libraries due to the cost of software and its management, and costly to users who must learn to navigate two or more systems to arrive at the information they need. A thorough review of the duplicate systems was conducted to identify functions and roles as they relate to the medical library and develop evaluation criteria for implementing changes. Key stakeholders at both the medical library and the university libraries worked to coordinate, establish team-management protocols, and when efficient, merge e-resource management systems across the university library system. After evaluation, there were some tools and procedures that were deemed too specialized, and must be maintained separately. Even in those cases, we learned more about the other campus libraries' processes and services, which led us to new content management ideas and streamlined electronic resources management.

Results: A number of collaborations with the University Libraries resulted in both time and cost savings to our library staff, as well easier navigation and increased resource access for our patrons: • Consolidated to a single remote-user authentication system (EZProxy) • Eliminated manually-generated A to Z lists of e-books and e-journals in favor of the university libraries' electronic resource management (ERM) system • Implemented a link resolver in PubMed, rather than maintaining our own LinkOut Holdings • Added the University Libraries' discovery tool (Summon) to our medical library website.

Conclusions: The College of Medicine's programs continue to grow more interdisciplinary and its educational goals reach far beyond its original departmental boundaries. Our former decentralized system kept us isolated. Our

users had to search multiple tools to check their access to electronic resources. By unifying information technology functions, and some acquisition and cataloging services, we have reduced duplication of resources while providing consistent, reliable access to licensed resources regardless of the source of funding within the University.

Poster Number: 196

Time: Sunday, May 15, 2016, 2:00 PM – 2:55 PM

Using Checklists to Train Users to Use the Mosaic of Library Resources and Services

Rick Wallace, AHIP, Assistant Director, Quillen College of Medicine Library, Quillen College of Medicine Library, Bristol, TN; Nakia Woodward, Sr. Clinical Librarian, Quillen College of Medicine Library, Quillen College of Medicine Library, Johnson City, TN

Objectives: The purpose of this study was to create a checklist that could be used in the training of a college of pharmacy faculty so that there were no gaps in their information literacy; to implement the checklist; and to measure the users response to the intervention.

Methods: A checklist was developed that covered the information resources of the library and the information skills needed by pharmacy faculty. All faculty were required to meet with a librarian during the academic year and cover everything on the checklist in one or multiple sessions. Faculty were surveyed as to their responses to the training.

Results: Librarians learned the value of using a checklist. Better relationships were developed with the college of pharmacy faculty. Weaknesses in the libraries training of pharmacy faculty were discovered and remedied.

Conclusions: Checklists are used in medicine to obtain quality. Checklists can be used in medical librarianship for the same purpose.

Poster Number: 197

Time: Sunday, May 15, 2016, 2:00 PM – 2:55 PM

Quality Improvement of an Online Bioinformatics Portal

Rob Wright, Basic Science Informationist; Peggy Gross, Public Health Informationist; Welch Medical Library, Baltimore, MD

Objectives:
Through user feedback, measure the value of and improve a library website that connects users to core resources in genomics, transcriptomics, proteomics, and other omics disciplines.

Methods:
The library's Bioinformatics Portal was launched in 2013 with advice from key faculty members and input from research groups and other stakeholders. It is a robust, curated clearinghouse of bioinformatics databases, tools, and tutorials. Using SurveyMonkey, a survey was developed and administered to faculty, staff, postdoctoral fellows, and graduate students across multiple schools and departments on a large academic medical campus. The survey gathered information about bioinformatics research domains and demographics. It also measured user perceptions of the portal's organization, design, features, and relevance to research. Based upon research interests, survey respondents were directed to specific pages on the portal and asked to evaluate the quality of resources on those pages. Responses to questions with preset choices were analyzed using SurveyMonkey. Comments sections were analyzed using NVivo. Changes to the portal's design and content were made based on these analyses.

Results
The 17-question survey was completed by 96 respondents. They reported the following breakdown of data use in their research: 75% genomics, 47% transcriptomics, 47% proteomics, 31% epigenomics, 24% metabolomics, and 15% pharmacogenomics. Seventy-one percent used more than one of these types of data.
Eighty-one percent found that the portal covered all important aspects of bioinformatics. Most also found the individual omics pages highly relevant. Respondents suggested the addition of resources to existing categories on the portal and recommended a number of new resource categories.
Only 51% found the portal attractive and engaging. Some free-text comments suggested alternative ways of organizing resources. While some respondents praised the portal's design, a larger number saw deficiencies, including poor readability, difficulty scanning for information, and a complicated structure.

Conclusions:
The survey gave the library valuable information about the demographics and bioinformatics interests of portal users. Genomics data was the most used type of data, but most used more than one type of bioinformatics data in their research. The relevance of resources on the portal as a whole and for individual omics pages was regarded as high by respondents. This being the case, they also identified a number of missing resources and categories of resources. Only about half of the respondents found the portal attractive and engaging. They made extensive comments regarding the portal's design and organization. These suggestions were incorporated into the portal's redesign.

Poster Number: 200

Time: Sunday, May 15, 2016, 2:00 PM – 2:55 PM

Introducing Evidence-Based Medicine Concepts to Academic Librarians: A New Model for Instruction

Roberta Bronson Fitzpatrick, Associate Director, George F. Smith Library of the Health Sciences, Newark, NJ;

Objectives: A poster describing a CAT-writing project was presented at the Rutgers University “State of the Libraries” meeting in 2014. It gained the attention of the Chancellor. At the conclusion of the discussion, he urged that transferrable concepts be taught to other RU librarians and incorporated within their instructional programs. A workshop was designed and presented in June and November 2015.

Methods: Specific elements of the CATs sessions were deemed to be transferrable to those providing instruction to students in the sciences and social sciences, as well as education and other disciplines. These include: foreground/background questions; writing a focused question; breaking a search question into the PICO format to assist with term generation, and the evidence pyramid. The workshop covered the CATs project as an example of a type of complete assessment, in that students must use the component skills to write their own CAT (Critically-Appraised Topic). Each of the noted transferrable concepts was covered. Attendees had time to come up with an example which would fit their subject audience and there a general discussion portion where ideas were exchanged on how to best incorporate these new skills into instructional programs.

Results: The workshops resulted in offers to teach collaboratively outside of our home campus. All attendees identified concepts which could be used directly so slightly modified to fit various disciplines. For example, article types listed in the evidence pyramid did not fit every discipline but librarians considered other types of resources, such as dissertations/theses and web sites/pages were listed in this format.

Conclusions: Rutgers University had few health sciences programs prior to 2013. Teaching the workshops was a good vehicle for informing our new colleagues about how instructional sessions are approached in the health sciences. It also allowed us to become part of the cadre of instructors and to be invited to attend meetings which focus on curriculum development and instructional techniques.

Poster Number: 203

Time: Sunday, May 15, 2016, 2:00 PM – 2:55 PM

Engaging Library Staff in Meaningful Work

Ryan Harris, AHIP, Reference Services Manager, Health Sciences and Human Services Library, Health Sciences and Human Services Librarian, Baltimore, MD; Everly Brown, Head, Information Services, Health Sciences and Human Services Library, Health Sciences and Human Services Library, Baltimore, MD

Objectives: Academic Health Sciences Library has an Information Services Department consisting of two librarians and six library services specialists. Specialists spend approximately sixteen hours a week working at the Information Services Desk providing reference, circulation, and computing assistance. To foster professional development and engage specialists in significant work, librarians developed new projects and roles for staff when not at the desk.

Methods: To understand staff ambitions and interests, Information Services librarians met with staff one on one to discuss the skills and expertise that they would like to develop. During these meetings, staff identified skills they were not learning in school, but thought were important (several staff members are working on MLS or other degrees). Areas of interest included: building instruction and leadership skills, and to support emerging technologies. The Information Services Librarians also collaborated with other departments, discovering projects and initiatives in need of skilled assistance. These efforts allowed the Information Services librarians to build a wide scope of new work to engage staff, with training provided as needed. After this process, staff were assigned new work projects that both help support the library and build their skills.

Results: Information Services Staff are now actively engaged in a variety of projects. They collaborate on research impact reports for campus Appointment, Promotion, and Tenure committees, develop graphic designs for library signage and handouts, oversee the library's Twitter and Facebook accounts and push out news stories to the campus' electronic newsletter, edit and create content for the library's electronic newsletter, teach workshops on library resources, and provide orientations and assistance to the library's 3D printing service. Staff members are more engaged in library-wide committees and chair the Staff Education and Training, and Exhibits and Displays committees.

Conclusions: Developing meaningful work for staff took time, careful planning, training and collaboration. The entire process was made easier because staff already possessed strong oral and written communication skills and had a willingness to develop new expertise. Information Services Staff are now

actively engaged in a variety of new projects and roles that enhance their skill sets as professionals and provide greater job satisfaction as they engage in meaningful and stimulating work that supports library initiatives. We continue to actively consider new ways to challenge and engage the staff.

Poster Number: 207

Time: Sunday, May 15, 2016, 2:00 PM – 2:55 PM

Metric Mosaic: Sixty-Five Pieces to the Puzzle to Demonstrate the Value of the Library to Stakeholders Using a Three-Pronged Approach to Benchmarking

Sandra Oelschlegel, AHIP, Library Director / Associate Professor; Martha F. Earl, AHIP, Assistant Director / Associate Professor; Preston Medical Library, Preston Medical Library / Health Information Center, Knoxville, TN

Objectives: To describe the process and impact of the Health Information Center/Preston Medical Library (HIC/PML) development of a comprehensive benchmarking tool, a Scorecard, and continuous quality assessment survey. These tools demonstrate the library's value to both academic medical center leadership and community stakeholders.

Methods: Relocation of the HIC/PML to increase patient centered services and resources resulted in direct reporting to both the Dean and the CEO. To influence decisions related to the library's funding and roles, the library used benchmarking, as a structured process improvement tool, to measure specific metrics over time. The project involved three branches of development and implementation: a benchmarking tool, a Scorecard using the hospital's OnFocus software, and a continuous quality assessment survey. By establishing what quantitative and qualitative data were of interest to both the Dean and the CEO, librarians developed a tool measuring 65 library metrics and a survey using questions similar to Press-Ganey. The Dean and CEO then chose a subset of both the metrics and survey data, to be reported monthly by the library director during meetings with each leader to present results and share feedback.

Results: This process allowed librarians to show value in terms relevant to hospital and academic leadership. Monthly reporting utilizing the scorecard and meetings with leadership provided the library director the opportunity to influence the decision makers. Benchmarking illustrated a 200% rise in facility use and 538% increase in consumer health services. Survey results revealed high user satisfaction. The scorecard displayed Sparkline trends of increasing utilization of resources and services. Both the Dean and the CEO expressed positive impressions based on the trends shown in the data. They shared those impressions with the key stakeholders, Board of Directors and Board of Visitors.

Conclusion: Proven success in the library's new location was shaped in part by the metrics selected and reported. The three pronged approach has successfully resulted in administrative support for continuation and growth of the HIC/PML in its new location.

Poster Number: 210

Time: Sunday, May 15, 2016, 2:00 PM – 2:55 PM

Has the Distribution and Role of Clinical Librarians in the United Kingdom Changed in the Last Ten Years?

Sarah Sutton, Clinical Librarian, University Hospitals of Leicester, Market Harborough, N/A, United Kingdom

Objectives: To establish if the number of Clinical Librarians (CLs) has changed over the eleven years since the first Clinical Librarian survey was conducted in 2004.. To investigate whether newer roles such as Embedded Librarians or Informationists have been established. To see if there is geographical variation in such roles and to gain data to enable further networking of these post holders.

Methods: A questionnaire was sent to the UK email discussion lists, also blogged and tweeted. The questionnaire was matched to the one used in 2004, so that trends could be identified.

Results: The results of the questionnaire were analysed. In 2004 26 librarians responded to the original survey and identified themselves as working as CLs (although the actual job title may have been different) in 2015 47 respondents identified as CLs, with an additional 32 identifying as Outreach Librarians, 9 as Embedded Librarians and 2 as Informationists. The 2004 CLs were largely full time, with 92% in this category, in 2015 the split was 58% full time and 42% part time.

Conclusions: Clinical Librarians have increased in numbers in the last ten years but so have other roles that mimic or build on the Clinical Librarian role. In 2004 Clinical Librarians were largely full time, in 2015 the part time roles have increased dramatically. A map will be included in the actual poster showing geographical distribution of the different roles.

Poster Number: 212

Time: Sunday, May 15, 2016, 2:00 PM – 2:55 PM

Interlibrary Loan and Document Delivery: A One-Year Pilot Program to Test the Impact of a No-Charge Service Model

Scott Thomson, AHIP, Library Director, Boxer Library, Rosalind Franklin University of Medicine and Science, North Chicago, IL

Objectives: To study the impact that ceasing to charge patrons for interlibrary loan and print scanning services would have on staff workload, patron satisfaction, and the library's ability to maintain copyright compliance.

Methods: Patrons frequently reported concerns over the fees associated with interlibrary loan (ILL) and print scanning services, even though many requests were filled at no cost. Moving to a completely no-charge model appeared to offer an easy way to increase satisfaction and simplify services. Funds generated by the services were trivial, so concern over lost revenue was minimal. However, there was concern that request volume could increase significantly, impacting staff time, and making compliance with CONTU "Rule of 5" copyright guidelines difficult. Therefore, we elected to conduct a one-year pilot program with a specific user population to measure the impact that moving to a no-charge model would have on services. In the fall of 2012, full-time faculty were notified that up to 8 fee-generating requests would be filled at no cost for the duration of the pilot program.

Results: Service usage did not increase significantly during the pilot program. The number of print scanning requests did not increase, and ILL article requests increased only 11%. At the conclusion of the pilot program, the library ceased charging for ILL and scanning services. Despite our efforts to gauge the impact that this change would have, service usage increased well beyond expectations and has continued to climb. Scanning requests increased 263% in 2013 and 75% in 2014. ILL requests increased 53% in 2013 and 153% in 2014. No other significant changes were made to the library collection/services to explain increased service use.

Conclusions: A pilot program may not accurately indicate the impact that moving to a no-charge model will have on service usage. Despite a significant increase in request volume, staff has been able to comfortably meet demand, although more workload sharing has become necessary, and stronger interlibrary loan policies and procedures have become important to help insure that patrons do not abuse the service. Compliance with CONTU copyright guidelines has not been difficult. Patron satisfaction, while not formally studied,

seems to have increased as well, as compliments are plentiful and complaints are now rare.

Poster Number: 213

Time: Sunday, May 15, 2016, 2:00 PM – 2:55 PM

Space, Resources, and Technology: Surveying Patron Needs at a Dentistry Library

Sean Stone, Dentistry Librarian, Indiana University School of Dentistry Library, Indianapolis, IN

Objectives: The IU Dentistry Library began in 1881 and is one of the only libraries of its kind. Library patrons were surveyed to determine their current perceptions and needs for space, resources and technology. Most existing surveys are inapplicable given the library's unusual nature. Patron data is crucial given recent changes, new curricula and space planning.

Methods: The library conducted an online survey of all Indiana University School of Dentistry students, faculty and staff which ran in Spring 2015 using the web-based survey management platform, Qualtrics. The anonymous survey consisted of one demographic question and 19 content questions. The total number of email recipients was n=1340 and there were n=256 responses and n=202 completed surveys yielding a response rate of 19% and a completion rate of 79%. By overall groups, respondents were 67% students, 16% staff, 14% faculty and 3% other. Questions measured patron library space and facility use, knowledge of and desire for library services and resources, technology usage and perceptions as well as overall negative and positive impressions and suggestions for improvement.

Results: Staff, resources, location and specific spaces were cited by respondents as positives while space issues such as allocation and the footprint of print collections were seen as the biggest negatives. Patrons are conservative in their adoption of new technology and have limited interest in social media but are well outfitted with current devices. Their desires for library services and technology focused on power access with high demand for additional outlets and charging options as well as a desire for printing but not computer access.

Conclusions: The results of the survey are driving current measures and short term plans for changes to library spaces, resources and services. It will also inform long term plans including a space plan that calls for reallocating and even expanding or moving the library to create a space with less emphasis on collections and more group and quiet/individual study areas. The IUSD library is completely changing its approach to print collection maintenance and access to better balance stewardship of historical and special collections with providing space desperately needed by patrons.

Poster Number: 214

Time: Sunday, May 15, 2016, 2:00 PM – 2:55 PM

Medical Librarians' Role in Clinician Recruiting in the Information Age

Shalu Gillum, AHIP, Head of Public Services, Harriet F. Ginsburg Health Sciences Library, Harriet F. Ginsburg Health Sciences Library, Orlando, FL; Natasha Williams, User Services Librarian, University of Central Florida College of Medicine, Harriet F. Ginsburg Health Sciences Library, Orlando, FL; ;

Objectives: To investigate online methods (1) by which clinicians might be looking for jobs in a broad range of specialties; and (2) how the college should advertise clinical job openings that would yield the greatest return on investment.

Methods: We assembled a team of faculty medical librarians, a human resources professional and a physician. To determine where clinicians might be looking for jobs, searches were performed in Google, and terms developed based words an individual might use to perform the query. Searches were conducted for twelve clinical specialties. We identified eight terms, with the name of the specialty inserted before the search term. Searches were conducted and the results recorded from the first two pages of Google screens. We excluded paid advertisements for our analyses.

Results: In total, 176 searches were conducted and 3520 results recorded. From these results it was evident that the same websites or types of websites appeared in the first page of search results in Google, i.e. the top ten search results, as Google defaults to ten search results per page. Our findings revealed that the following five websites or types of websites appeared in the top ten search results across all specialties searched: Indeed, Practice Link, JAMA Career Center, NEJM Career Center, and a society website (i.e. relevant to the specialty searched), for example, American Association of Clinical Endocrinologists.

Conclusions: Based on the results of these searches, the medical librarians were instrumental in providing guidance for all future search committees at the College of Medicine charged with hiring clinicians. We were able to develop best practices for employers hiring clinicians in an academic medical center, and advice for clinicians searching for employment online.

Poster Number: 216

Time: Sunday, May 15, 2016, 2:00 PM – 2:55 PM

The Hospital Librarian: A Mosaic of Talents and Skills Meeting the Information Needs of Health Care Professionals

Sheila Hayes, Semi-Retired, Semi-Retired, Semi-Retired, Broad Brook, CT

Objectives: Hospital Librarians once offered a unique service with very strict parameters as to what a medical library was, what it looked like and what it contained. The existence of the library was supported by the Joint Commission. The Joint Commission mandated that a hospital would have a library. The physical space was everything and the librarian maintained this space. When the Joint Commission changed this mandate from an actual library to offering Knowledge Based Information (KBI) medical libraries came under siege to morph into something else from the traditional space. In reviewing the literature from 1997 to the present the change from space centered to librarian centered will be illustrated in cartoon form. This will illustrate the extraordinary talents and skills that have been added to this profession to stay alive and become part of hospital services not as a standalone but as an integrated part of the institution.

Methods: The review of literature will cover 1997-present. This review will look at all the talents and skills that were illustrated in the JAMIA publication 1997 4(1): 87 that sited the categories that have expanded library skills into education, finances, fundraising, general librarianship, health sciences, institutional and professional skills, marketing, medical informatics, growth in character and skills, use of previous experience as building blocks, research and development and technical skills. The literature will also be reviewed for the addition of electronic medical record involvement, learning management systems and continuing medical education as library services. Personal stories will be collected from librarians who have transitioned from library as place to librarian centered using the HLS ListServ as well as individual interviews with noted librarians who have experienced it all. The author is an artist and the cartoons will be original works.

Results: The results should be a positive understanding of the hospital librarian. And, the illustrations can be used by any librarian who needs to see the whole picture of this remarkable profession. The reference list that will be offered is the results of a three part journal club (12 MLA credits over a year and a half) by the author.

Conclusions: This presentation will be a light illustration of the profound changes that hospital librarians have experienced and a look at the possibilities for the future.

Poster Number: 219

Time: Sunday, May 15, 2016, 2:00 PM – 2:55 PM

Presenting Institutional Research Performances from the Perspective of Researchers: A Case Study of Constructing the "Pure Experts" System in Taipei Medical University (TMU)

Shu-Yuan Siao, Head, Knowledge Services Sec, Main Library, Taipei Medical University Library, Taipei, N/A, Taiwan; Hsiao-Fen Yu, Librarian, Taipei Medical University Library, Knowledge Services Sec., Taipei, N/A, Taiwan; Tzu-Heng Chiu, Professor/Director, Center of General Education/International Cooperation Division, National Central Library, Center of General Education/ Library, Taipei, Taiwan

Objectives: To integrate academic achievements, highlight research specialties, cumulate CV of faculty, display research performances, and enhance international visibility and global collaboration, TMU assigned the university library (TMUL) to build up the "Pure Experts" system. This poster shares experiences of this project, including how TMUL conduct the project, procedures in choosing system, and what kind of data to be imported… etc..

Methods: This project was conducted in the following steps: (1) survey existing systems - invite related vendors to the TMUL to introduce their systems; (2) hold project meeting - serve as the chairperson, the Vice President called for a meeting to evaluate each system and discuss elements of entries to be imported. The project team includes members from the University Library, Office of Finance, Office of Human Resources, Office of Information Technology, and Office of research and development; (3) procurement of the selected system (the "Pure Experts"); (4) cumulate and import data; (5) launch the system; and (6) system application and service promotion.

Results: In Taiwan, Taipei Medical University (TMU), National Taiwan University (NTU) and National Taiwan Normal University (NTNU) have constructed similar systems. In order to search potential researchers, enhance international visibility and global collaboration, TMU chose to utilize the Elsevier Pure Experts Portal. The project started on Oct.1 2015 and the"pure experts" system was launched on Feb.2 2016 with the data of 91 academic unites and 555 faculty (http://tmu.pure.elsevier.com/). TMU library allocated 2 full time staff to collect data, and personnel files, Scopus publication, research projects, patents, equipment, projects, books of each expert were uploaded to the system. NTNU used the same system (http://ntnu.pure.elsevier.com/en/) in Sept. 2014 and it included data of 200

faculty and 92 academic units. NTU used an ORCID-based system (http://ah.ntu.edu.tw/web/Index.action) developed by a local vendor and it included 2,530 faculty and 193 research units.

Conclusions: There are only three universities constructing expert systems in Taiwan. Two of them utilized "the Elsevier" solution and one of them chose to develop customized system with a local vendor. Both approach has its pros and cons. The difficulties of this project were the willingness of academic units to provide information, data collecting and proofing, and foreign vendor can't react our requests immediately. Finally, whether this system can enhance the international visibility and research opportunities of TMU is still need to be examined.

Poster Number: 225

Time: Sunday, May 15, 2016, 2:00 PM – 2:55 PM

Health Literacy with Comics: Using a Comic Book Format to Help Families Prevent Childhood Obesity

Talicia Tarver, Research and Education Librarian, VCU Libraries: Tompkins McCaw Library, Virginia Commonwealth University, Richmond, VA; Deidra Woodson, Metadata and Digitization Librarian, Health Sciences Library, LSU Health Shreveport Health Sciences Librarian, Shreveport, LA; William Olmstadt, AHIP, Associate Library Director, LSU Health Shreveport, LSU Health Shreveport Health Sciences Library, Shreveport, LA; Charlie Tudor, Assistant Systems Librarian, LSU Health Shreveport, LSU Health Shreveport Health Sciences Library, Shreveport, LA; Nicholas Fechter, Graphic Artist, LSU Shreveport, LSU Health Shreveport Health Sciences Library, Shreveport, LA; John Vanchiere, Assistant Professor, Department of Pediatrics Chief, Section of Pediatric Infectious Diseases, LSU Health Shreveport Department of Pediatrics, Section of Pediatric Infectious Diseases, LSU Health Shreveport Health Sciences Library, Shreveport, LA

Objectives: This poster will illustrate a National Library of Medicine (NLM)-funded health literacy project to create a comic book, offered in both print and online formats, to address the obesity epidemic among at young readers (4-8 years of age).

Methods: The librarians applied for an NLM Health Literacy Award to fund the project. The award was used to cover the artist's commission and all printing and promotion costs. The librarians then hired a graphic arts student from a local university to illustrate the comic. After consulting the Pediatrics Department faculty for expert advice on content and the local population, the librarians began developing the story. They sent the text to the artist, who sketched rough drafts. After the sketches were approved, he illustrated them on story boards, digitized them, and added color using a graphics computer.

Results: Upon completion, 500 print copies were printed and distributed throughout the community with the cooperation of the Pediatrics Department and the local public library systems. To reach as many readers as possible, an online version was also launched at http://www.healthelinks.org/kidshealth/. This project was publicized throughout many venues, including the first annual Louisiana Comic Con, the local newspaper and radio station, and countless newsletters and websites. The online version has received over 1233 (1101 via the web and 132 via mobile) hits to date.

Conclusions: Pediatricians from the Pediatrics Department were surveyed to

determine if the subject matter had been effectively depicted. Anecdotal information was also collected from teachers, public librarians, local community leaders, and children. All results were overwhelmingly positive, and the librarians continue to receive feedback that the project was successful in its attempts to communicate health information to children.

Poster Number: 228

Time: Sunday, May 15, 2016, 2:00 PM – 2:55 PM

Pattern of Co-Authorship of Medical Researchers in University of Ibadan

Toluwase Victor. Asubiaro, Librarian II, University of Ibadan/University College Hospital, E. Latunde Odeku Medical Library, College of Medicine, Ibadan, N/A, Nigeria

Objectives: The paper aims at identifying the degree of collaboration and types of research collaboration among medical researchers at University of Ibadan through the analysis of the pattern of co-authorship of published articles of medical researchers of the University that are indexed on PubMed.

Methods: Data for this research work consisted of 1,100 articles/publications that were retrieved based on two criteria on PubMed database. First, the articles are those whose authors have affiliation with University of Ibadan and indexed on PubMed database. Secondly, the time frame for publication dates is a five-year period of 2010 to 2014 available. Variables such as number of authors, departments of authors, institutions of authors and countries of authors were collected and analyzed. The degree of research collaboration will analyzed through the analysis of co-authorship. The types of research collaboration are categorized as domestic intra-institution and domestic inter-institution and international collaboration. This will be analyzed by considering the number of departments, institutions and countries of co-authors.

Poster Number: 231

Time: Sunday, May 15, 2016, 2:00 PM – 2:55 PM

Cost Recovery at a Multiregional Health Sciences Library

Travis Schulz, AHIP, Enterprise Director of Medical Library, Sanford Health, Bismarck, ND

Objectives: In the fall of 2014 two independently administered health sciences libraries within a single rural health system were administratively consolidated. A directive from hospital administration required an analysis of the library collections and services to demonstrate a return on investment. After a retroactive analysis of internal markets, ejournal and ebook statistics, and interlibrary loan borrows, a cost recovery plan was created.

Methods: A business plan was created to map out the objectives, mission, and goals of the multiregional library. As part of the business plan's financial plan, a cost recovery strategy was developed and applied across four metrics: hours library personnel spent conducting literature searches; full-text journal access; interlibrary loan borrows; and ebook access.

The hourly rate for literature searches was determined by analyzing business literature and selecting a consulting fee model that fit the library's method of service delivery. The value of ejournal and interlibrary loan borrows was calculated by using an industry standard fixed value. Ebook value was calculated by establishing a fixed value using internally established metrics.

Results: Applying the cost recovery strategy across the four metrics for Fiscal Year 2015 revealed a cost recovery of 72.13%. The cost-benefit ratio was calculated at 2.08 for ejournals; 9.23 for interlibrary loan; and 1.57 for ebooks.

Conclusions: The proposed methods demonstrate a basic cost recovery strategy using tangible metrics. This cost recovery method can be used to communicate to hospital administration a return on investment for library expenditures. Further improvement in the cost recovery strategy can be made by quantifying and monetizing the impact library collections and services have on intangible metrics such as process improvement; innovation, research, and translational medicine initiatives; and clinical outcome and patient experience goals.

Poster Number: 234

Time: Sunday, May 15, 2016, 2:00 PM – 2:55 PM

Two Years in the Life of a Nursing Embedded Librarian

Victoria Burchfield, Nursing Information Librarian, Robert B. Greenblatt, MD Library, Augusta University, Augusta, GA

Objectives: To demonstrate usage patterns and activities of librarians embedded in a college of nursing.

Methods: Two years' worth of usage statistics collected by embedded librarians were examined for patterns and trends in utilization of embedded librarian services. Interviews were conducted with librarians who had acted in the role of embedded librarian for the college of nursing since the inception of the university's embedded librarian program to gain clues into the ways in which the librarians were active in the college.

Results: From June 13, 2013 through June 12, 2015, 521 reference transactions were recorded from the college of nursing. 276 (53%) of these transactions were initiated by students and 185 (36%) by faculty, with the remainder initiated by staff, senior administrators, librarians, and employees of the university's health system. Student-initiated transactions were primarily basic reference or one-on-one instruction sessions, while faculty-initiated transactions were mostly basic reference, literature search requests, or for scholarly support. Within the college of nursing, the librarian is part of the center for nursing research and regularly attends academic affairs meetings and information technology committee meetings.

Conclusions: Insight into the ways in which the embedded librarian integrated into the college of nursing can help other embedded nursing librarians identify ways to become involved in the college, and knowledge about services utilized can help librarians target advertising of their services.

Poster Number: 237

Time: Sunday, May 15, 2016, 2:00 PM – 2:55 PM

Comparing the Effectiveness of Conceptual Search Methods: Is a Fast Approach Sufficient for the Production of Sound Systematic Reviews: A Prospective, Double-Blinded, Controlled Study

Wichor M. Bramer, Biomedical Information Specialist, Medical Library, Erasmus MC - Medical Library, Gouda, N/A, Netherlands

Objectives:
When performing searches for the systematic review, it is imperative to ensure that no (important) relevant articles are missed. Several guides and methods have been published that provide guidance in searching for the systematic review. Despite these rigorous and detailed methods, searching for a systematic review requires experience and a lot of time. Recently, a new, faster method created by the first author, was used in hundreds of SR projects. The goal of this article is to compare the effectiveness of different searching methods and experiences.

Methods:
Search strategies for several new systematic reviews will be designed independently by three information specialists, one coordinating searcher and two test searchers (roles can vary among persons). After the coordinating searcher has performed a librarian-mediated search strategy for a systematic review he or she invites two test searchers to try to replicate or improve the search results. On a set date he or she sends information on the review question to the test searchers, who then start searching simultaneously. Every 30 minutes they send their preliminary results to the coordinating searcher. After a maximum of 4 hours the searches must be finalized.
The results will then be combined into one single dataset and deduplicated to present to researchers. We determine overlap between the search results, and characteristics of the search strategies (e.g. number of search terms, databases and search results). After the included references of the review are determined, an independent information specialist checks the search results of each searcher for presence of the included reference. What was the recall of the final searches, and how did the recall evolve during the search time.

Results: The results are not yet available at the time of submitting the abstract.

Poster Number: 240

Time: Sunday, May 15, 2016, 2:00 PM – 2:55 PM

SHARE the Info: Spreading Health Awareness with Resources and Education: A National Network of Libraries of Medicine-Funded Program

Yini Zhu, Managing Librarian, Head of Access Services, George F. Smith Library of the Health Sciences, Rutgers, the State University of New Jersey, Newark, NJ; Mina Ghajar, Information & Education Librarian, Rutgers, The State University of New Jersey, George F. Smith Library of the Health Sciences, Newark, NJ; Ermira Mitre, Library Technician, George F. Smith Library, Rutgers , The State University of New Jersey, Access Services Department, Newark, NJ

Objectives: Despite the availability of various online patient education resources, the majority of physicians have not incorporated them into practice. The SHARE Program's objective is to reach out to physicians, faculty, students, nurses, patients and their families to raise the awareness of consumer health information resources available through the National Library of Medicine and the Health Sciences Libraries at Rutgers University.

Methods: In evaluating our library services, we determined that a gap existed in promoting patient education resources. To bridge this, we decided to create the SHARE Program (Spreading Heath Awareness with Resources and Education). First, we determined that this program should be jointly implemented by the Access Services and Reference departments due to their respective strengths in knowledge of library services and research instruction. Next, we agreed that our efforts should focus on promoting NLM's MedlinePlus health and Drug Information, and HealthyNJ due to their comprehensive and authoritative information. Finally we determined the format for SHARE would be weekly tables/tents at the Medical School and the University Hospital where we would conduct demonstrations and trainings and distribute specially designed SHARE Card, instructional and health literacy materials. Evaluation form and log sheet would be collected for program assessment and analysis.

Results: SHARE launched on August 26, 2015 and is slated to end on August 30, 2016. Evaluation forms and log sheets are being collected at each session to assess participant feedback and the resulting data are being analyzed and evaluated as they are collected. A detailed program report will be submitted to the NN/LM Grant Review Committee before May 14, 2016.

Conclusions: As this is an ongoing project, a conclusion is not available at this time. All collected data will be analyzed prior to MLA 2016. Conclusion will be included in the poster.

Poster Number: 3

Time: Monday, May 16, 2016, 2:30 PM – 3:25 PM

Seeing the Big Picture Through Smaller Screens: Characterizing the Library Users' Experience on Mobile Devices

Aileen M. McCrillis, AHIP, Head, Information Services and User Experience, NYU Langone Medical Center, NYU Health Sciences Library, New York, NY; Catherine Larson, Head, Systems & Technology / Systems Librarian, NYU Health Sciences Library, NYU School of Medicine, New York, NY; Allison E. Piazza, AHIP, Health Sciences Librarian Fellow, Pratt Institute, NYU Health Sciences Library, New York, NY

Objectives: Mobile devices are becoming profoundly important tools for finding and accessing information. This is especially true in medicine, where clinicians routinely use mobile devices to inform clinical decision-making. Accessing institution-licensed content on mobile devices; however, can be problematic resulting in a negative user experience. We will perform an assessment of selected library systems and institution-licensed information resources for mobile compatibility.

Methods: We will perform an assessment of selected library systems, publisher platforms, and licensed information resources for compatibility with several types of smartphones and tablets. We will characterize compatibility based on the availability of standalone mobile websites, mobile-responsive websites, apps, and the ability to access institution-licensed content through existing channels (e.g. a library's website). We will also explore the barriers and facilitators to accessing institution-licensed content on mobile devices. The results of this study will reveal the possible pitfalls of providing access to licensed information on mobile devices and help to inform an improved experience for mobile device users.

Poster Number: 6

Time: Monday, May 16, 2016, 2:30 PM – 3:25 PM

Better Together: Combining Research and Writing Support for Student Scholars

Alexander J. Carroll, Research Librarian for Engineering and Biotechnology, North Carolina State University Libraries, Raleigh, NC; Kelsey Corlett-Rivera, Head of Research Commons, University of Maryland, McKeldin Library, College Park, MD; Linda Macri, Director of Writing and TA Training Initiatives, University of Maryland, The Graduate School, College Park, MD

Objectives: Libraries offer workshops on finding evidence; writing centers offer assistance with synthesizing evidence into effective literature reviews. The University of Maryland (UMD) Libraries and the UMD Graduate School Writing Center investigated the effectiveness of combining these traditionally separate efforts into a suite of instructional programming, which presented research and writing as linked and iterative components of a process rather than distinct stages of scholarship.

Methods: The UMD Libraries sought to increase its impact on student scholarship by creating information literacy workshops that better met the needs of students. To determine student needs, library staff queried liaison librarians, who noted that graduate and upper-level undergraduate students struggle with entering into their discipline's academic discourse. To demonstrate to students the iterative process of finding and using evidence, library and writing center staff developed programming on how to write a literature review for a thesis or dissertation. Staff from both departments designed instructional content on how to find, access, store, and use evidence effectively when writing. These sessions also introduced students to the concept of scholarship as a conversation, how to apply the stasis theory of composition, and explained the rhetorical purpose of conducting a literature review. Attendance and session evaluations were used to measure effectiveness.

Results: A traditional graduate student workshop series held in the library, which presented research and writing as separate processes, was attended by 22 students in 2013 and 35 students in 2014. The literature review workshops, which offered integrated research and writing instruction, were attended by 119 students in 2014 and 96 students in 2015.

Conclusions: This successful collaboration highlights the benefits of forming strategic partnerships with groups on campus who have shared values with the library, as these sessions developed in concert with the Graduate School Writing Center substantially improved student attendance at library workshops. Subsequently, discipline specific breakout sessions have been developed,

which utilize the expertise of liaison librarians to offer focused training on specific resources and writing techniques within subject disciplines. This collaboration also has led to the development of additional experimental programming with the Graduate School Writing Center. Some of these initiatives include librarian office hours in the writing center, and workshops on transforming a traditional research paper into a compelling oral presentation.

Poster Number: 9

Time: Monday, May 16, 2016, 2:30 PM – 3:25 PM

Tais-Toi and Do It: A Free Webinar Case Study

Amy Blevins, Associate Director for Public Services , Ruth Lilly Medical Library , Indianapolis, IN; Margaret A. Hoogland, AHIP, Distance Support Librarian, A.T. Still University of Health Sciences, A.T. Still Memorial Library, Kirksville, MO; Ryan Harris, AHIP, Reference Services Manager, Health Sciences and Human Services Library, Health Sciences and Human Services Librarian, Baltimore, MD; Maureen Knapp, AHIP, Research Support & Education Librarian III, Rudolph Matas Library of the Health Sciences, Tulane University - Matas Library, New Orleans, LA

Objectives: An MLA section decided to provide free educational webinars to members of MLA on issues related to distance education, information literacy, etc. Current section members were eligible for free MLA CE credits for one of the webinars.

Methods: A section member belongs to an institution that provides all library staff members with access to webinar systems. This person partnered up with members of the section to host the first ever section organized educational webinar in September 2014. Section members and other professional colleagues, volunteered their time as presenters. In July 2015, the section hosted its second webinar via the ZOOM conferencing system. In September 2015, the section hosted a webinar from which section members were able to obtain MLA CE credits. The sections' CE coordinator worked with MLA to obtain CE credit and the section provided the financial support. All of the webinars were advertised through the section's blog, list-serv, Facebook account, and Twitter hashtag. Surveys were sent out to the participants of the first and third webinars to measure satisfaction, demographics, and future webinar topics.

Poster Number: 12

Time: Monday, May 16, 2016, 2:30 PM – 3:25 PM

Driving the Last Nails into the Print Coffin: Improved Course Support Through Reserve Promotion and Evaluation

Andre Joseph. Nault, Head Librarian, Veterinary Medical Library, Veterinary, St. Paul, MN; Nicole Theis-Mahon, Librarian Liaison to School of Dentistry & HSL Collections Coordinator, Health Sciences Libraries, Health Sciences Libraries, Minneapolis, MN

Objectives: How can a veterinary library take a lead role in educating students on materials which support their course learning and simplifying access to these items while minimizing the cost to students? A use assessment of materials used in courses was also needed to provide evidence of impact and insure that appropriate formats were chosen to maximize student satisfaction and learning.

Methods: A list of all the required, recommended, or reserve materials listed in syllabi for all the courses in the Doctor of Veterinary Medicine (DVM) program were gathered by the Office of Student Affairs and shared with Library staff. During the fall 2014 semester, 21 difference courses listed information resources in their syllabi; in the spring 2015, 26 did. Web pages containing Google analytic code were constructed for each semester by program year, and all these materials were listed for quick reference for students. Hyperlinks were inserted into all html citations to allow quick retrieval from the print collection, or quick access to the e-version. Several book chapters or sections were scanned and placed into e-reserves as needed when the publisher was not offering an electronic version for sale to libraries.

Results: A total of 47 classes referenced informational resources, while additional 29 courses did not. These materials totaled 246 titles, although multiple formats and editions were listed on the course readings web pages. Circulation and use data revealed 435 uses of print materials, while ebook views were 15,963; over 36 times higher. E-reserves accounted for 320 uses. Percentage wise, this indicated a very strong preference for e-access, as 97.4% of materials accessed by students were electronic . [Web page views of course pages are still being tabulated due to a recent platform migration]

Poster Number: 15

Time: Monday, May 16, 2016, 2:30 PM – 3:25 PM

Building the Big Picture: Creating a Systematic Review Process Across a Multi-Site, Multi-Library System

Ann Farrell, Librarian V, Mayo Clinic Libraries, Plummer Library, Rochester, MN; Tara Brigham, Medical Librarian, Mayo Clinic Libraries, Winn-Dixie Foundation Medical Library, Jacksonville, FL; Lisa A. Marks, AHIP, Director of Libraries, Mayo Clinic, Staff Library, Scottsdale, AZ; Amanda K. Golden, Librarian III, Mayo Clinic, Mayo Clinic/Plummer Library/Center for Regenerative Medicine, Rochester, MN; Leah Osterhaus Trzasko, Librarian, Health Science Library, Plummer Library / Public Services, Rochester, MN; Jennifer Schram, Librarian, Medical Library, Mayo Clinic Libraries, Eau Claire, WI; Diana Almader-Douglas, Librarian, Mayo Clinic, Hospital Library, Phoenix, AZ; Connie M. Bongiorno, Librarian, Mayo Clinic, Mayo Clinic - Saint Marys Campus, Rochester, MN; Matt Hoy, Supervisor, MCHS – Eau Claire, MCHS – Eau Claire Medical Library, Eau Claire, WI

Objectives: Our objective was two-fold: 1) To ensure that our experienced reference librarians were aware of and were consistently using best practices for systematic reviews and 2) to develop a formal process for completing SR searches, from authors requesting librarian assistance to handing off final search results.

Methods: With the push to publish systematic reviews (SR) authors are encouraged to use library services for the search process. Several options exist to learn the SR process, such as self-study, MLA CE courses and multi-day workshops. At our institution, with campuses across the Midwest, Florida and Arizona, librarians formed a special interest group (SIG) to work together to become adept at the SR process.

The learning process was an iterative one. We created a repository to share documents, search strategies, and EndNote libraries. We developed a survey to gauge librarian SR experience before and after the implementation of the SIG and a spreadsheet to track details about search requests. We met monthly to review our progress, discuss search methodologies and best practices for searching databases. We also created a LibGuide about the SR process for both librarians and library users.

Poster Number: 18

Time: Monday, May 16, 2016, 2:30 PM – 3:25 PM

Emerging Library Leadership Across the Spectrum

Annie M. Thompson, Director, University of Southern California, Wilson Dental Library, Los Angeles, CA; Stephen Kiyoi, AHIP, Library Director, Barnett-Briggs Medical Library, UCSF at ZSFG, San Francisco, CA; Nancy Olmos, Head, Metadata and Content Management, Collection Resources Division, Norris Medical Library, Norris Medical Library, Los Angeles, CA

Objectives: This poster will discuss healthcare library leadership from the perspective of three new leaders in the field, discuss the challenges new leaders face in light of shifting trends in health sciences librarianship, and present information regarding the availability of educational leadership growth opportunities.

Methods: Three librarians will discuss their experiences related to taking on new leadership roles at early career stages. An environmental scan of educational programs and resources available for emerging and new leaders will also be conducted and presenters will also reflect on their personal experience related to leadership. The programs include the NLM Associate Fellowship, the Harvard Leadership Institute for Academic Librarians, the UCSF Masters of Science in Healthcare Administration and Interprofessional Leadership, as well as a Graduate Academic Certificate in Advanced Management in Libraries and Information Agencies from the University of North Texas.

Results: This poster will provide a look at trainings available to those interested in leadership roles and it will also depict each librarian's path so far.

Conclusions: Each librarian will be available to discuss lessons learned along with how leadership training has impacted their careers.

Poster Number: 21

Time: Monday, May 16, 2016, 2:30 PM – 3:25 PM

Collaborative Quality Assurance in Literature Searching

Beata Pach, Manager, Library Services, Knowledge Services, Public Health Ontario, Toronto, ON, Canada; Allison McArthur, Library Information Specialist, Public Health Ontario, Library Services, Toronto, ON, Canada; Susan Massarella, Library Information Specialist, Public Health Ontario, Library Services, Toronto, ON, Canada; Domna Kapetanos, Library Operations Technician, Public Health Ontario, Library Services, Toronto, ON, Canada; Sarah Morgan, Library Operations Technician, Public Health Ontario, Library Services, Toronto, ON, Canada

Objectives: Public Health Ontario provides expert scientific and technical advice and support to government, local public health units and health practitioners. Library Services support is embedded in project plans across the organization to ensure a consistent approach to PHO research products. In turn the quality of PHO research relies on the expertise of the library team in conducting comprehensive literature searches.

Methods: To ensure that Library Services delivers literature searches which are of a consistently high quality, a unique rapid peer review and quality assurance process for search strategies has been implemented. The team has made this process an integral part of their workflow. Team members provide feedback on all aspects of search strategies, including database selection, relevancy/sensitivity of subject headings and keywords, combination of search concepts, application of limiters, and proofreading of search syntax. This collaborative approach to search strategy development leverages the collective expertise of the team, promotes knowledge translation and capacity building among Information Specialists and Library Technicians, and improves the quality and consistency of search strategies. This process has also facilitated the development of a shared repository of search terms for frequently searched public health concepts which has been utilized by the team to expedite search strategy development.

Results: N/A - embedded in the methods section

Conclusions: The rapid peer review process of search strategies demonstrates how this approach is successful in improving the overall quality of the host organization's knowledge products and evidence-based advice.

Poster Number: 24

Time: Monday, May 16, 2016, 2:30 PM – 3:25 PM

Evaluating Online Health Information Sources: A How-to on Mixed-Method Heuristic Design

Blake Hawkins, Graduate Student, University of British Columbia, Master's of Library and Information Studies, Vancouver, BC, Canada; Heather L. O'Brien, Assistant Professor, University of British Columbia, School of Library, Archival, and Information Studies, Vancouver, BC, Canada

Objectives: Consumer health websites play a vital role in the mobilization and translation of population-based health information. In order to decide whether to engage with these websites or recommend them to others, health consumers and librarians need a quick and effective means of evaluating them. The poster describes the development and application of a heuristic measure for men's health websites.

Methods: We developed a heuristic evaluation tool that built upon Jacob Neilsen's 10 Usability Heuristics For Usability Design. We augmented these established heuristics with knowledge gleaned from the men's health, health promotion and information seeking literature focused on the inclusion for different racial and sexual minority groups. The elements incorporated from the heuristic measure included the website system, visual design, and content; as they were the most pertinent for the purpose of this project. Furthermore, we incorporated theoretical aspects from health promotion, men's and queer health and health information seeking for sub-questions regarding content and our analysis. The resulting tool was applied to two British Columbia men's health websites to assess their functionality, visual design, and information content.

Results: This mixed-method approach to creating a measure was successful at highlighting beneficial and problematic areas of the sample websites. The five measures that were taken from Jenkins (1992) model were able to evaluate the structural and functionality aspects of the websites. We were able to evaluate if the information was free of dead links and other error glitches that might cause frustration for potential men's health information seekers. Furthermore, the measure could evaluate design and if the presentation of information was overwhelming or not for the seeker – this can have an effect on the level of engagement by the users. By incorporating qualitative and secondary questions, we were able to retrieve even richer data about the sample websites regarding men's health. It became apparent that currently, men's health is sometimes forgetting ethnic and sexual minorities. Neither of our websites had a visible Aboriginal or Gay, Bisexual, Transgender, or Queer content, which is problematic since these two groups have poorer health outcomes.

Conclusions:
By creating this mixed-method heuristic measure it provides the opportunity for further research with a larger sample regarding men's health promotion online materials. Potentially, the success with these two websites was an anomaly and there are more elements that need to be considered when creating this type of evaluation for online materials.

Poster Number: 30

Time: Monday, May 16, 2016, 2:30 PM – 3:25 PM

Evaluative Bibliometrics Meets the Clinical and Translational Science Institute

Caitlin Bakker, Biomedical/Research Services Liaison, University of Minnesota, Bio-Med Library, Minneapolis, MN; Katherine Chew, Research/Outreach Services Librarian, Health Sciences Libraries, Health Sciences Libraries / University of Minnesota, Minneapolis, MN

Objectives: Bibliometrics, the application of quantitative analysis to publications, is of growing importance for institutions, departments, and research centers. For Clinical and Translational Science Award (CTSA) centers, these metrics are both a tool for assessment and for self-advocacy. This poster describes one library's collaboration with a CTSA center to employ evaluative bibliometrics.

Methods: The Libraries were approached by a CTSA center to engage in a process of identifying and implementing the most appropriate bibliometrics for evaluation purposes. This initiative leverages the library's understanding of NIH's Public Access Compliance Monitor, Scopus, Web of Science, and research networking systems such as SciVal Experts and Pure. Using grant information, a strategy was developed to identify all CTSA-funded publications and to calculate and represent effective measures of impact.

Poster Number: 33

Time: Monday, May 16, 2016, 2:30 PM – 3:25 PM

Open Outreach: Launching an Open Access Policy in the School of Nursing

Caitlin Pike, AHIP, Nursing and Medical Humanities Liaison Librarian, Indiana University-Purdue University Indianapolis (IUPUI), IUPUI University Library, Indianapolis, IN

Objectives: On October 7th, 2014, a Midwestern university's Faculty Council approved a campus-wide Open Access Policy. The opt-out policy leverages the institutional repository to provide open access to faculty scholarly articles. Some participation of the faculty members is required to make it work. We hypothesized that personalized liaison intervention, rather than a generic library-based email, would increase participation from the faculty.

Methods: Following several University-wide announcements of the Open Access Policy's adoption, the School of Nursing (SON) liaison librarian sent individually tailored emails requesting the final revised manuscripts to faculty members with publications in 2015. These emails were sent in April and August, and reminder emails were sent two weeks after the initial contact. Response rates were tallied after each round of emails to determine the effect of liaison involvement in the manuscript collection process.

Poster Number: 38

Time: Monday, May 16, 2016, 2:30 PM – 3:25 PM

Patient Education on an Inpatient Rehabilitation Unit

Catherine Young, Librarian Supervisor, Medical Library, Saskatoon Health Region, Saskatoon, SA, Canada

Objectives: To provide patients and families with access to reliable health information and improve their health literacy, while also increasing the Medical Library's profile within the organization, by taking ownership of a patient education program on an inpatient rehabilitation unit.

Methods: The Medical Library runs a patient education program on an inpatient rehabilitation unit that admits approximately 300 inpatients per year for a variety of conditions, with the most common diagnoses being Stroke, Acquired Brain Injury, and Spinal Cord Injury. A librarian attends weekly scheduling meetings on the unit to make patient education appointments for new patients and attaches a letter explaining the purpose of the appointment to the patient's schedule. During the scheduling meeting, the librarian consults with unit staff regarding patients' abilities to participate independently in appointments. The following week, a librarian meets with patients and their families in their rooms or in the unit lounge. During the appointment, the librarian provides health literacy instruction, takes information requests, and demonstrates an online patient education guide on an iPad for patients and families who identify as internet-users.

Poster Number: 41

Time: Monday, May 16, 2016, 2:30 PM – 3:25 PM

Heart-Healthy Conversations: A Librarian-Physician Partnership to Promote Disease Management and Wellness Information to Cardiology Patients

Christine Caufield-Noll, AHIP, Manager, Library Services, Johns Hopkins Bayview Medical Center, Harrison Medical Library, Baltimore, MD; Linda Gorman, Director, Library Services, Johns Hopkins Bayview Medical Center, Harrison Medical Library, Baltimore, MD;

Objectives: To partner with outpatient cardiology physicians to provide their patients with relevant and appropriate health information from MedlinePlus.gov and other reliable sources. This information will reinforce healthy habits and promote successful disease management. This partnership will also grow the visibility of library staff on campus and demonstrate librarians' ability to support patient-centered care.

Methods: Setting: An outpatient cardiology clinic on the campus of an academic medical center staffed with two faculty, three fellows, and one library staff person.

Population: Adults with heart conditions who are visiting the clinic for routine care.

Intervention: Patients will be brought to the librarian by their physician for an information consultation. The librarian will query the patients about which topic(s) they would like more information, or the physician will ask the librarian to research the patient's specific condition or provide healthy lifestyle materials. Patients leave with a packet of information and their physicians are apprised of the topics discussed so they can add this feedback to the patient's electronic health record. The librarian will also follow up with patients by phone two weeks after their appointments to assess the relevance and usefulness of the information provided.

Results:
In Phase I (7/2014-9/2015), the author (LG) saw 94 patients in clinic. In Phase II (9/2015-present), the authors (LG & BV) saw 69 additional patients, bringing the total number of patient interactions for the whole project to 163. In Phase II, patient satisfaction surveys were initiated.
Forty-five percent of those patients (n=31) completed this survey.
Seventy-one percent were "very satisfied" with a librarian as part of their healthcare team. Seventy-one percent "will use" the resources with which they

were provided. Fifty-two percent "will use" the online resources shown to them. Ninety percent responded that the information given to them at the visit was "very helpful" or "helpful."

Conclusions:

This project has encouraged a valuable partnership between the cardiology clinic and library. Physicians utilize the librarian for literature searches and patients are able to receive authoritative health information. Patients are equipped with valuable information as they leave their clinic visit, allowing them to be more engaged in their health care. This project demonstrated that an embedded librarian partnering with physicians can be a value-added service for patients. It is expected that this partnership between the library and cardiology clinic will become a regular part of library services.

Poster Number: 45

Time: Monday, May 16, 2016, 2:30 PM – 3:25 PM

Be Part of the Big Picture Through Community Engagement: Collaboration and Partnership Provide Summer Library Programs for Middle School Students

Dana Ladd, Community Health Education Center Librarian, VCU Libraries Community Health Education Center, VCU Libraries Community Health Education Center, Lanexa, VA; Irene Machowa Lubker, Research and Education Librarian, Virginia Commonwealth Univeristy, Tompkins- McCaw Library for the Health Sciences, Richmond, VA; Thelma A. Mack, Research and Education Coordinator, Virginia Commonwealth University, VCU Libraries, Tompkins McCaw Library for the Health Sciences, Richmond, VA

Objectives: As part of a university wide program committed to community engagement, The Community Health Education Center (CHEC), a library for patients and their family members, and the Tompkins McCaw Library (TML) for the Health Sciences participated in a program designed to offer middle school students an opportunity to explore science, technology, the arts, math, and health sciences. The libraries provided the students with an overview of consumer health and health sciences libraries.

Methods: A diverse group of students from the Summer Discovery Camp, sponsored by the Mary Francis Youth Center visited the libraries. Activities were planned to align with the overall vision and goals of the youth center's program. The session in the CHEC library included four activities through which the students rotated. The activities included: 1. Library reference treasure hunt; 2. Exploring health careers table; 3. Children's area health games activities; and 4. Interactive online health and wellness games and activities. For the second half of the program, the students visited TML where Special Collections staff explained various medical tools used in early medical and dental practices. Students were then given an opportunity to explore health science careers including academic requirements and salaries online. Finally, the students participated in an interactive activity aimed at learning about health conditions using the medical conditions of celebrities as examples.

Results: Three sessions were given over the past three years with a total of 45 students attending. Verbal feedback from the students and the Summer Discovery Coordinator was positive. While the students did not specifically evaluate their library sessions, their overall experience with their sessions was overwhelmingly positive with 92% reporting, "I liked my morning classroom experience" of which the library program was one of the morning sessions.

Conclusions: This partnership and collaborative program allows the libraries to provide outreach services through an interactive program aligned with the vision and mission of the Summer Discovery Camp. These programs not only provide middle school students with an overview of consumer and health sciences libraries and finding health information but also allow them to explore other health sciences careers in a fun, interactive environment.

Poster Number: 47

Time: Monday, May 16, 2016, 2:30 PM – 3:25 PM

The Evolution of Health Sciences Librarian to Knowledge Manager: Reflections on Building a Knowledge Management Framework

Dana Thimons, AHIP, Associate Director of Knowledge Management, American Association of Colleges of Pharmacy, Alexandria, VA

Objectives
One-year fellowship at a professional association building a knowledge management framework to identify, capture, and share the knowledge assets of pharmacy faculty and association staff. The goal of the framework is to increase effectiveness and reduce redundancy by ensuring that the appropriate knowledge is easily accessible and available, as well as creating an environment that encourages innovation and collaboration.

Methods
An academic pharmacy liaison librarian was selected for a year-long fellowship at a professional association. To increase competency in knowledge management (KM), the fellow conducted an in-depth literature review and visited organizations with established KM strategies. The fellow attended the association's meetings; met with staff; and examined the network drive, website, and intranet to gain an extensive understanding of the association's knowledge assets and needs. The fellow conducted focus groups with pharmacy faculty and visited schools of pharmacy to identify members' knowledge assets and needs. The association intends to improve pharmacy education by implementing KM practices to meet members' knowledge needs. A KM framework was drafted. The framework detailed the people, processes, cultural changes, and technology necessary to implement KM. Pilot projects were identified, launched, and assessed to gain member buy-in and determine if the framework should be fully activated.

Results
The association's Board of Directors approved the KM framework. The association created a full time knowledge manager position for a library and information science professional. The fellow was hired for this KM role to continue working on the framework and implementing KM throughout the association.

Conclusions
There is no one size fits all approach or solution to implementing KM within a library or organization. Knowledge management increases effectiveness and

reduces redundancy by ensuring that the appropriate knowledge is easily accessible and available. KM is not a magic solution to every problem that a library or organization faces. Many elements of knowledge management are similar to those already utilized by librarians. The skills needed to complete many librarians' regular job duties—including conducting reference interviews, literature searches, organizing information, committee work, marketing services, change management, and assessment—are transferrable and help with successful KM implementation. As libraries and librarians' roles evolve, librarians may want to consider roles as knowledge managers or implementing knowledge management in their libraries.

Poster Number: 50

Time: Monday, May 16, 2016, 2:30 PM – 3:25 PM

Politics: Dirty Word or Fascinating Opportunity to Leverage Your Skills and Abilities?

Darell Schmick, Research Librarian, University of Utah, Spencer S. Eccles Health Sciences Library, Salt Lake City, UT; Barb Jones, Library Advocacy/Missouri Coordinator, University of Missouri, J Otto Lottes Health Sciences Library, Columbia, MO

Objectives: To establish a peer based book discussion group specifically on the topic of political savvy in the health sciences/hospital work environment.

Methods: Members across the Region were surveyed to identify areas of needed training. A topic suggested by many was advocacy, specifically being "politically savvy". Acting on this, two librarians across institutions within the same region identified books that would lead to engaged, action oriented discussion groups. Discussions took place over Adobe Connect, utilizing the software provided by the National Network of Libraries of Medicine (NN/LM). Discussion group attendees were surveyed to determine the effectiveness of the book discussion group as a training tool, and whether or not they increased their political awareness.

Results: Preliminary survey responses demonstrate positive results for participants. Answers to survey questions indicate that participants have a greater awareness of politics in their respective workplaces. All participants elected to participate in a future book club. Open ended responses reflected this sentiment, as one specific commenter stated "more, more, more" regarding future book club opportunities. At time of writing, there are two more book clubs scheduled.

Conclusions: The peer based book discussion group specifically focusing on political awareness and skills proved to be a success. While initially targeted at Health Sciences Librarians within the Midcontinental Region, the book club enjoyed participants from different library environments across the nation.

Poster Number: 58

Time: Monday, May 16, 2016, 2:30 PM – 3:25 PM

Assessing the Impact of Literature Search Services: Supporting Our User Community's Work Productivity

Donna S. Gibson, Director of Library Services, Library, Memorial Sloan Kettering Cancer Center, New York, NY; Antonio P. DeRosa, AHIP, Research Informationist II, Memorial Sloan Kettering Cancer Center Library, New York, NY; Marisol Hernandez, Senior Reference Librarian, Library, Library, New York, NY; Konstantina Matsoukas, Senior Reference Librarian, Memorial Sloan Kettering Cancer Center, Library, New York, NY

Objectives: To evaluate the impact and value of literature searching by determining how scientists and health care professionals actually used the information in the search results in their research and medical activities.

Methods: A nine question, online survey was developed to solicit feedback and identify the reason(s) for the search request, how the user planned to utilize the information received, and its potential impact on the user's work. Each time a literature search was requested, the reference team (four librarians), sent the survey link with the search results. Data collection started in February 2015 and ended December 2015.

Results: During this 11 month period, a total of 805 searches were completed by the reference team. From this pool of potential survey responses, we received a total of 220 completed surveys, for a survey response rate of 27.3%. The results of our survey provided a define list of reasons that show the value and impact that mediated-search results have for our user community.

Conclusions

This online survey helped to identify the impact and value of mediated-literature search services and showcase connections between the search results and how the user utilized the information received and the impact the content (search results) had on their work and decision-making.

Poster Number: 61

Time: Monday, May 16, 2016, 2:30 PM – 3:25 PM

Student Perceptions of Librarian-Facilitated Interprofessional Education Sessions

Elizabeth G. Hinton, AHIP, Reference Librarian, Informational Services; Mitzi R. Norris, Executive Director for Academic Effectiveness; Susan B. Clark, Library Director; University of Mississippi Medical Center, Jackson, MS

Objectives: A librarian-led assessment initiative was developed to answer the following questions: What impact do interprofessional (IP) sessions have on a) student beliefs and attitudes about interprofessional learning, and b) student learning about basic life support (BLS) in an interprofessional environment? The study incorporated currently enrolled students representing different programs of study who had undergone BLS training.

Methods: The library worked with multiple campus groups to host an interprofessional student event in the library's Collaborative Learning Center with a clinician/library faculty-led discussion on BLS and interprofessionalism. Outcomes were measured by pre/post-tests and facilitated discussion for four separate focus groups. A clinician administered a BLS pre-test and library faculty administered an IP core competency survey. Following a short video clip depicting an interprofessional BLS event, students were asked to react to the video and share their opinions and feelings regarding IP. Finally, post-tests were administered in order to measure subjective interprofessional growth and the retention of BLS review. Notes were taken during the discussions so keywords could be analyzed as qualitative data upon conclusion.

Results: The initial sample indicates growth in both objective and subjective measures. Answers to the IP competencies clearly show a stronger appreciation for interprofessionalism after the sessions. Scores from the six-point rubric increased from 6-24 points for each IP competency, with one competency regarding the avoidance of discipline-specific jargon showing a 120% increase. In addition, BLS test results show the potential for reviewing basic clinical concepts in an interprofessional education environment. 88% of the test scores either improved or remained the same. Common themes emerged throughout the sessions that demonstrate student preference for transparency of school curricula and more chances for interprofessional interaction.

Conclusions: Health sciences students recognize the importance of interprofessional education. In an effort to help their institutions meaningfully connect IP to core curricular competencies, libraries can assert themselves as stakeholders in the evolving curricular standards. By collaborating with

colleagues from different campus departments, librarians can successfully aid in the facilitation of interprofessional events.

Poster Number: 64

Time: Monday, May 16, 2016, 2:30 PM – 3:25 PM

From the Inside Out: Writing as Healing Program Uses Research-Based Reflective Writing Techniques to Reduce Stress and Improve Quality of Life

Ellen Justice, AHIP, Community Health Librarian & Manager, Junior Board Cancer Resource Library, Helen F. Graham Cancer Center, Christiana Care Medical Libraries, Newark, DE; Joan DelFattore, Professor Emerita, University of Delaware, Junior Board Cancer Resource Library, Newark, DE; Leanne Holveck, Senior Library Assistant, Junior Board Cancer Resource Library, Junior Board Cancer Resource Library, Newark, DE

Objectives: To describe the *Writing as Healing* program and its outcomes.
Methods: The library staff hosts a volunteer writing instructor who facilitates free 2-hour workshops, open to all, twice per month. She also offers 1-hour sampler workshops to healthcare employees, who learn about the program and develop writing skills for their own wellness. Marketing methods will be described. Anecdotes from participants and the instructor will be presented. Survey results will be highlighted.
Results: In November 2014, the Junior Board Cancer Resource Library staff offered two 2-hour writing sampler workshops which attracted thirty-six participants. A program evaluation and brief survey indicated that overall, participants were "very much satisfied" with the sampler. All indicated that they learned something, and thirty agreed it would benefit their wellness. Most wanted a regularly scheduled program, and most wanted to learn expressive and/or reflective writing and poetry. The instructor and the occasional guest instructors have successfully provided that content. The program is based on medical and psychological studies demonstrating the effectiveness of expressive/reflective writing in improving quality of life and reducing stress. Research-based writing exercises and discussion prompts are used and explore various themes. In addition, our main instructor creates a safe, relaxed, and trusting environment. Since March 2015, the library staff hosted twenty workshops with ninety-five participants, some of whom have attended multiple sessions. Six abbreviated staff workshops attracted thirty-three. The program has been listed in the local newspaper's health section; we post and distribute a flyer; it is regularly listed on Christiana Care's Events & Classes website; it has been promoted via the External Affairs publication FOCUS; it is listed in the libraries' newsletters; and we email notices to library customers.
Conclusions
Most people who attend *Writing as Healing* express satisfaction with what they gain from the writing and discussions. There are new attendees each month, and many participate monthly. Costs, including staff time, are very

manageable. Continued interest, satisfaction, and growth motivate us to keep offering the program, and the librarian has broadened our collection with recommended writing resources including a magazine, DVD, and books. Offering programming to the public improves their knowledge of our library services as well as creating a writing community that explores interesting and difficult topics in a private, confidential setting.

Poster Number: 67

Time: Monday, May 16, 2016, 2:30 PM – 3:25 PM

Searching for Evidence: An Analysis of Medical Student Examination Data

Emily Mazure, AHIP, Biomedical Research Liaison Librarian, Medical Center Library & Archives, Duke University, Durham, NC; Megan G. Van Noord, Research & Education Librarian, Duke University Medical Center Library, Duke Medical Center Library & Archives, Durham, NC; Megan von Isenburg, AHIP, Associate Director, Research and Education, Medical Center Library & Archives, Duke Medical Center Library, Durham, NC; Brandi Tuttle, AHIP, Research & Education Librarian, Medical Center Library & Archives, Medical Center Library & Archives, Durham, NC

Objectives: In second year medical students completing an Evidence Based Medicine (EBM) assignment during their internal medicine rotation, which aspects of the PICOTT (Patient/Problem, Intervention, Comparison, Outcome, Type of Question, and Type of Study) and PubMed search strategy predict relevant search results and selection of articles?

Methods: In this IRB approved study, we analyzed student examination data collected over four years from the Objective Structured Clinical Examination (OSCE) EBM station. Using a case, students were responsible for developing a PICOTT and clinical question, conducting a PubMed search, selecting relevant articles, and critically appraising an article using standard FRISBE validity criteria. All data from the assessment grading rubrics were anonymized and coded. Using correlation and regression analyses, we will look at the following relationships: Does a high score on the developed PICOTT lead to a higher score for article selection? Does a high score on the search strategy lead to a higher score for article selection? Are there individual factors of the PICOTT or search that lead to selecting appropriate articles? Additionally, we will examine how time impacted performance.

Poster Number: 70

Time: Monday, May 16, 2016, 2:30 PM – 3:25 PM

MS Buddy: A Personalized Tablet Lending Program for Multiple Sclerosis Patients

Erica Lake, Associate Librarian, Spencer S. Eccles Health Sciences Library, Spencer S. Eccles Health Sciences Library, Salt Lake City, UT

Objectives: ILEAD leadership institute brings together academic, public, and special librarians to create participatory technology projects addressing identified community needs. A 2015 ILEAD Utah team developed an innovative service connecting individuals physically and socially isolated by multiple sclerosis (MS) to information, resources, and support.

Methods: An abundance of MS information and support is available online, however many people living with the disease in rural and urban areas of Utah do not have access to a computer or internet service, or do not have sufficient technology skills. Others lack confidence in their ability to identify trustworthy information. MS patients often have long stretches of time between doctor visits and do not always feel well enough to leave the house. During that interim, many information needs occur and the likelihood for isolation and withdrawal increases. The MS Buddy service loans iPads to participants to help them learn more about their disease through vetted apps, websites, videos, and support resources. The librarians provide individual training on using the tablet, social media and email, and preload personalized content for each participant based on their geographical location. This includes links for their doctor, health care facility, local public library, regional health library, and MS chapter.

Results: Participants' evaluations from the beta phase have been very positive. Participants gained the skills and confidence needed to use a variety of technologies and to access meaningful information, and increased their online and community support connections. The ILEAD team created a step-by-step toolkit to enable other librarians and health care providers to adapt and implement a similar project in their communities.

Conclusions: Many patients today want "high tech" health care services as well as "high touch" interactions, and librarians are in a unique position to bridge these wants through services that are personalized to individual patient preferences and needs.

Poster Number: 74

Time: Monday, May 16, 2016, 2:30 PM – 3:25 PM

Analysis of a Textbook Program at a Multi-Campus Library System

Estelle Hu, AHIP, Health Sciences Bibliographer/Assistant Professor, Library of the Health Sciences, University of Illinois at Chicago, Library of the Health Sciences, University of Illinois at Chicago, Chicago, IL; Rebecca Raszewski, AHIP, Associate Professor & Information Services Librarian, University of Illinois at Chicago, Library of the Health Sciences, Chicago, IL

Objectives: This research project analyzes textbooks' usage that were purchased with funding from a library fee on multiple campuses.
Methods: Print textbooks were purchased for students from colleges across three library locations, Chicago, Peoria and Urbana. Chicago started purchasing textbooks with the library fee in fiscal year 2014 while Peoria and Urbana started fiscal year 2015. Chicago's textbooks were held in the reserve collection behind the circulation desk, while textbooks were either behind the circulation desk or in a browseable, open reserves section at Peoria and Urbana. Circulation reports were collected from January 2014 to October 2015. The reports included circulation data by library location, and patron types.
Results: Out of 346 titles purchased at Chicago with the library fee, 145 (42%) of them circulated 1092 times. These 145 textbooks were almost half of the 298 reserve books that circulated overall during January 2014-October 2015. They also represented 1,092 (59%) out of 1,838 of the total number of circulated reserve books.
Out of the 26 titles purchased at Urbana with the library fee, only 6 titles circulated 124 times. This was only 6% of the 107 reserve books that circulated overall. The library fee funded textbooks' circulation was 124 (7%) of 1776 total checkouts. Even though Urbana serves fewer colleges, the total amount of reserve titles that circulated was 1,776, compared to 1,838 at Chicago.
For Peoria, 56 titles were purchased with the library fee. Twenty-five titles circulated 82 times which was 92% of the 27 circulated titles. The 82 times of uses represented 92% out of the total use 89.
Medical students, residents and fellows were the patron types that used textbooks the most frequently: 476 (26%) out of 1838 for Chicago, 1206 (68%) out of 1776 for Peoria and 98 (87%) out of 115 for Urbana.
September, October and January were the months with the highest circulation rates among all locations.
Conclusion: While the textbook program had been having an impact on Chicago's and Peoria's reserve collections' circulation, library fee funded textbooks made up a small portion of Urbana's reserve collections' circulation. Textbooks circulated the most frequently during the beginning of fall and spring semesters. E-book versions of the most heavily circulated print textbooks will

be considered for future purchases. The textbook's e-book version usage data and circulation data through the end of December 2015 will be monitored for further analysis.

Poster Number: 80

Time: Monday, May 16, 2016, 2:30 PM – 3:25 PM

Putting the Pieces Together: Designing Online Instruction for a Family Medicine Clerkship

Gail Y. Hendler, Associate Provost and Director, Health Sciences Library, Loyola University Chicago Health Sciences Library, Maywood, IL; Elizabeth Q. Huggins, Information Services and Instruction Librarian, Loyola University Chicago, Health Sciences Library, Maywood, IL

Objectives: Feedback from the 2013 Family Medicine clerkship information skills training revealed challenges to onsite learning experienced by students assigned to remote rotations. Library faculty collaborated with the clerkship director in 2014 to design and deliver online instruction to remove learning barriers. Instructors created an interactive SoftChalk module which included a mandatory assignment that was returned to students with formative assessment.

Methods: Instructors reviewed existing curriculum with the clerkship director and adapted content for online learning using a constructivist approach. SoftChalk was selected to create and deliver the module to 160 third-year medical students staggered over the academic year. Based on a clinical case students would likely encounter during the rotation the module taught information strategy and skills clinicians would need to find answers to patient care questions and included: focusing the question with PICO, using PICO terms in search strategy, navigating Clinical Queries to locate an answer. The module contained a PICO quiz and a video tutorial on searching Clinical Queries. Students applied skills taught in the assignment: summarize the case; focus with PICO, navigate Clinical Queries with PICO, locate best evidence from the search. Students received formative assessment from librarians on information skills and from preceptors on critical appraisal skills.

Results: Students gained hands-on practice in applying information skills taught in the module to a case from their Family Medicine rotation. Submissions revealed varying levels of expertise in meeting the four learning objectives. Formative assessment allowed librarians to reinforce learning and provided the opportunity to teach additional skills when appropriate.

Conclusions:
Softchalk is an easy-to-use and affordable instructional technology that enabled the curriculum designers to meet learners' preferences and needs for online delivery of library skills training. Librarians plan to meet with the course director

and the instructional design team to review course evaluations and plan next steps.

Poster Number: 83

Time: Monday, May 16, 2016, 2:30 PM – 3:25 PM

How to Prevent Your Flip from Flopping: Five Key Mistakes to Avoid When Switching to the Flipped Classroom Model

Gary S. Atwood, Health Sciences Education Librarian, Dana Medical Library / University of Vermont, Burlington, VT

Objectives: Contrary to popular perception, successfully adopting the flipped (or inverted) classroom model requires more than just recording videos of lectures for students to watch outside of class. This poster will highlight five key mistakes that teachers sometimes make when adopting the flipped classroom model and outline effective strategies to avoid them.

Methods: A broad review of the literature provided the basis for my research findings. My research began by reading "Flip Your Classroom: Reach Every Student in Every Class Every Day" by Jonathan Bergmann and Aaron Sams, the two teachers credited with launching the flipped classroom movement. In addition, I read the studies and reviews referenced on the Flipped Learning Network's "Research on Flipped Learning" website. My last activity involved a keyword search for "flipped classroom" OR "inverted classroom" in PubMed. Three recently published research articles that describe efforts to convert a medical course to the flipped classroom model were selected for review. I identified the common factors that contributed to the success or failure of the flipped classroom initiatives discussed in the literature and synthesized the recommended practices to address the five most pressing problems that arose.

Results: Based on my research, there are five key mistakes that teachers make when adopting the flipped classroom model:

1. Failure to adapt course content to the new model
2. Failure to select and implement technology that is appropriate for the information being taught
3. Failure to anticipate the costs associated with the flipped classroom model
4. Failure to incorporate effective assessment methods into the course
5. Failure to convince students to embrace the new teaching model

One of these mistakes alone will not cause a course to fail, but committing more than one makes it increasingly difficult to be successful.

Conclusions: Adopting the flipped classroom model requires careful planning and lots of hard work in order to be successful. By understanding and avoiding the five key mistakes outlined in this poster, teachers can increase their chances of success and create a class where students are engaged in the learning process.

Poster Number: 86

Time: Monday, May 16, 2016, 2:30 PM – 3:25 PM

A Mosaic of Interdisciplinary Teammates Developing and Implementing Clinical Training with Virtual Humans

Hannah F. Norton, AHIP, Reference & Liaison Librarian, Health Science Center Library, Biomedical and Health Information Services, Health Science Center Libraries, Gainesville, FL

Objectives: In order to train clinicians on team skills without all team members being present, an interdisciplinary group of collaborators at a major academic health center and affiliated teaching hospital developed a series of team trainings using virtual humans. These trainings are targeted towards improving communication across the clinical team for the purposes of patient safety.

Methods: Virtual humans, computerized displays that look and speak like people, provide much of the realism of an actor or standardized patient while increasing consistency in response to trainees' diverse actions. Using this experiential environment, nurses were trained in the following scenarios: discrepancy in closing count during surgery and taking and labeling a blood sample in the midst of distractions. During implementation of the first training scenario, library staff joined an interdisciplinary team of simulation experts, computer engineers, IT professionals, and nurses to serve as training proctors, running virtual human software and selecting recorded responses based on trainees' actions. Library staff assisted in the development of the second training scenario, then taught nurse educators to serve as training proctors. This project serves as a model for the libraries in teaching the use of tools and technologies that our patrons have designed.

Poster Number: 89

Time: Monday, May 16, 2016, 2:30 PM – 3:25 PM

Medical Librarian as Fulbright Scholar: You Could Be the Next Winner

Hongjie Wang, AHIP, Head of Research & Instructional Services, Lyman Maynard Stowe Health Science Library, Farmington, CT

Objectives: How could medical librarians enhance their chance to become winners of the highly competitive, merit-based grant of the Fulbright Program?

Methods: Drawing upon his personal experience as Fulbright Scholar in China 2013-14 and as Fulbright Review Committee member 2014-2016, this presenter will highlight the nature, scope and timeline of the program and share useful tips on how a medical librarian could be the next winner for the prestigious Fulbright award.

Results: Medical librarians will become familiar with the Fulbright Program and motivated to compete for this life-changing learning experience for their professional advancement and personal growth.

Conclusions: The Fulbright Program is the largest U.S. exchange program offering opportunities for medical librarian all over the world to participate. It aims to increase mutual understanding between the people of the United States and other countries through the exchange of persons, knowledge, and skills. Medical librarians and their libraries will benefit tremendously from this experience.

Poster Number: 94

Time: Monday, May 16, 2016, 2:30 PM – 3:25 PM

Expanding Marketplace Application Opportunities: The Public Library as a Certified Application Counselor Organization

Jennifer L. Kaari, Community Services & Family Literacy Librarian, Montclair Public Library, Montclair, NJ

Objectives: For the 2015-2016 Open Enrollment period, the Information Services Department of a public library sought to expand our capacity to provide Marketplace enrollment assistance to our community. In previous years, the library hosted navigators from a local community organization to provide patrons with enrollment assistance. However, patron demand outnumbered the available appointments the navigators were able to provide.

Methods: The library was designated a Certified Application Counselor organization by the Centers for Medicare and Medicaid Services in 2015. This allows the library's employees and volunteers to become Certified Application Counselors (CACs) after completing the appropriate CMS training. CACs help consumers understand, apply, and enroll for health coverage through the Marketplace. Throughout the 2015-2016 Open Enrollment period, the Community Services Librarian and library volunteers are available during scheduled drop-in periods and by appointment to assist patrons one-on-one with completing and understanding the Marketplace application and enrollment process.

<>

Poster Number: 95

Time: Monday, May 16, 2016, 2:30 PM – 3:25 PM

All of These Things Are Not Like the Others: A Comparison of Six Clinical Librarian Programs at the University Health Network

Jessica Babineau, Information Specialist, Library and Information Services, Toronto Rehab - University Health Network, Toronto, ON, Canada; Ani Orchanian-Cheff, Archivist & Information Specialist, Library and Information Services, University Health Network, Toronto, ON, Canada; Melanie Anderson, Information Specialist, Library and Information Services, University Health Network, Toronto, ON, Canada; Bogusia Trojan, Director, Library and Information Services, University Health Network, Toronto, ON, Canada; Marina Englesakis, Information Specialist, Library and Information Services, Health Sciences Library / Library and Information Services, Toronto, ON, Canada

Objectives: T
o compare and contrast the formats and outcomes of six clinical librarian initiatives established over the last 10 years within different clinical programs at the University Health Network (UHN), an academic teaching hospital in Toronto, Canada.

Methods

A comparative case presentation demonstrates the range and variety of services provided through six clinical librarian programs for family medicine, general surgery, psychiatry, bariatrics, genitourinary oncology, and chronic pain management, of which five continue to this day. Elements considered include program mandates, meeting structures and librarian's roles and responsibilities. Indirect impact on library service usage will be explored.

Results:
Over the course of 10 years, six clinical librarian programs were created at UHN. Of the six, only one was discontinued after a few years:
librarian attendance at weekly genitourinary tumor boards.
The remaining five continue to provide meaningful service to clinicians, despite a high level of heterogeneity in the programs' scope of practice. Benefits and value of clinical librarian programs were demonstrated through varying means including survey data, the duration of our existing programs, interest expressed by other clinical areas, acknowledgements from program education leads, positive feedback from participants, as well as apparent correlations with increased usage of library services. Program descriptions and evaluations will be discussed in greater depth.

Conclusions:
Clinical librarian initiatives need to be flexible and fluid in their roles in order to successfully embed themselves within clinical programs. Our comparison demonstrates that even within one institution, there is no single formula for a clinical librarian's role. The needs and mandates of individual programs vary. Despite their variance, each clinical librarian program supports the institution to be a learning organization, and contributes to evidence-based patient care and safety.

Poster Number: 100

Time: Monday, May 16, 2016, 2:30 PM – 3:25 PM

Delays in Availability of Faculty Journal Articles: Local Effects of Open Access Policies

John H. Wiswell, Health Sciences Librarian, Applachian State University, Belk Library, Boone, NC

Objectives: This study will explore descriptive statistics about Open Access availability of locally produced journal articles. Of articles published by faculty at three regional universities, what proportion is available immediately on the open web? For how long are the remainder delayed? What part of the delay is due to publisher restrictions? How is this changing?

Methods: Monitor several article databases (and Google Scholar) for peer-reviewed publications by university faculty from several departments at three regional universities. Collect dates they are published online. Check each article each month for non-subscription, Open Access availability. Collect information on publisher embargoes and other restrictions. Collect publisher, disciplinary, author, and article information. At least one figure will show each journal article on the vertical axis, with horizontal bars representing delay in OA availability, publisher embargo, and the difference. At least one table will show descriptive statistics.

Results: My results are not complete yet.

Conclusions: My results are not complete yet.

Poster Number: 103

Time: Monday, May 16, 2016, 2:30 PM – 3:25 PM

Reading for Recovery: Exploring Partnerships Between Professionals in Addiction and Information Science

Judit H. Ward, Director of Information Services, Center of Alcohol Studies, Center of Alcohol Studies, Piscataway, NJ; William A. Bejarano, Senior Information Specialist, Center of Alcohol Studies, Rutgers University, Piscataway, NJ

Objectives:
To highlight the collaboration of academic and public librarians with addiction professionals, counselors, and researchers To provide an example of how a library can bridge the gap between research and practice by exhibiting existing resources, benefiting from information services To demonstrate an ALA Carnegie–Whitney Grant-funded project to create a tool to serve broad audiences related to books promoting recovery

Methods:
Based on recent advancements in research and practice in the emerging field of bibliotherapy, that is, using reading as a supplementary treatment option, the authors have been collaborating to develop an online tool that will connect those in need with resources that may meet their needs. Our team of information and addiction professionals has been working on a project called "Reading for Recovery" (R4R). Methods include establishing inclusion criteria for a database that will serve as reader's advisory for librarians, addiction counselors, and therapists. Titles include educational literature, self-help books, memoirs, fiction, and other classics. Consolidating metadata and exploring potential platform options are crucial components of the project. The interface will be created using the most current digital library technologies to develop an English-language database of titles already available in public libraries in the United States.

Results
The pilot resulted in a successful collaboration across disciplines. The public librarian was essential to establish selection criteria and adopt a "book club" model, enhancing the reading experience with talking points and questions created by our team. Collaboration with addiction professionals on the general potential of art therapy confirmed the need for a tool which encourages the readers' active participation. Experimenting with four different applications confirmed our hypothesis that there is no single platform to meet the needs of the diverse audience. One solution is to develop two separate platforms in

tandem; one for browsing, and another for searching.

Conclusions

This project needs the multiple angles of all participants to ensure the input of all diverse perspectives. Subject specialist librarians are in a unique position to coordinate a transdisciplinary project. Working as partners with addiction professionals, librarians can add value to treatment options, given their expertise on title selection and technology. Still underutilized as a treatment modality in the United States, bibliotherapy offers great potential to connect people in recovery with their inner selves through reading. This project could serve as the catalyst to adopt the European model of training librarians and graduates of relevant degrees to become bibliotherapists.

Poster Number: 104

Time: Monday, May 16, 2016, 2:30 PM – 3:25 PM

Be a Part of the Big Picture--Literally! Library-Sponsored Crowdsourced Art, Puzzles, Pet Therapy, and Other Stress-Busting Activities for Medical Students

Kacy Allgood, AHIP, Research and Community Engagement Librarian, Indiana University School of Medicine, Ruth LIlly Medical Library, Indianapolis, IN; Jennifer Herron, Emerging Technologies Librarian, Ruth Lilly Medical Library, Ruth Lilly Medical Library, Indianapolis, IN; Sherry Kieper, Technical Assistant, Indiana University, Ruth Lilly Medical Library, Indianapolis, IN

Objectives: Encourage medical students to pursue a balanced work/life by providing study break activities in the library. Participants expand creative and collaborative skills & develop coping skills. Utilize library space in unique ways, in the service of our students.

Methods: Purchase, organize, schedule and publicize crowdsourced creative projects, including: puzzles, paint projects, coloring books for adults, pet therapy visits and more. New creative projects are available every week. Students assemble puzzles, and use colored pencils in coloring books and use acrylic markers to complete canvases of varying sizes. The paintings are similar to a large, community, paint-by-number. The library art committee selects royalty free images to trace onto varying sizes of stretched canvases. Every two weeks, students are provided a fresh canvas to fill in or design. Students complete the artwork. Completed artwork is hung on library walls or donated to appropriate departments or university-related officials. Students were provided with a small survey to determine use and needs.

Results: Pilot project has been tested. Puzzles are completed within 1-4 weeks, depending on complexity. Paintings were usually complete within one week. Most participants create during late-night library hours. Students complete large paintings quickly, sometimes following the paint-by-number plan, and often adding their own creative details. Make-and-take artwork is completed in only one night.

Conclusions: The presence of the creative projects and other stress-busting activities encourages students to take a study break & utilizes library space for the service of our students. Survey results indicate students are engaged in the creative activities, want them to continue. Students often suggest ideas for crowdsourced art.

Poster Number: 106

Time: Monday, May 16, 2016, 2:30 PM – 3:25 PM

Mind-Mapping the Brainstorm: A Visual Technique for Search Query Planning

Kaeli Vandertulip, AHIP, Health Science Liaison Librarian, University of Texas at Arlington Libraries, Euless, TX

Objectives: Complex topics with multiple facets, related terms, and levels of complexity benefit from a flexible search technique that still provides some structure. The mechanism I use and promote is based on graphic organizers used to help students organize their writing.

Methods: I would like to demonstrate and explain the mind-mapping technique I use primarily with nursing students creating complex literature review searches. This has helped me in reference interviews and in one-shot instructions by allowing me to show students the intricacies of their topics as well as the level of flexibility required to find the highest quality research that relates most to their topics of interest.

Results: As a program description, there are no formal results aside from examples of successful queries.

Conclusions: Students want to search for PICO questions in full, using the first terms that come to mind. By using mindmaps, I am able to show novice researchers how to break down a search, to consider broader and narrower terms, and how to keep track of their search histories. This also makes explaining nested searches, MeSH terminology and subheadings, and keyword hierarchies easier.

Poster Number: 108

Time: Monday, May 16, 2016, 2:30 PM – 3:25 PM

The Big Picture: An Interprofessional Collaboration to Increase Awareness of Veteran Care Among Health Care Students

Karen S. Lamson, Reference and Instruction Librarian; Assistant Professor, Library and Learning Resources, MCPHS University, Worcester, MA

Objectives: The purpose of this study was to develop an annual Veteran Centered Care Conference targeting contemporary healthcare needs of Veterans, outside of the VA setting, with the goal of enhancing the knowledge of the health professional students across the MCPHS University campuses.

Methods: An interprofessional collaboration of library, nursing, physical therapy, physician assistant, and pharmacy faculty was formed to establish a Veteran Centered Care Conference. The conference involved panelists consisting of veterans or those who care for veterans and provided discussion on a variety of topics: access to care, appropriate assessments, mental health issues, traumatic brain injury treatment and care of women veterans. A case study was facilitated by the faculty and used to engage an interprofessional team of students after the panel. Attendees completed a survey tool and reflection following the conference. This study received IRB approval.

Results: To be added at a later date.

Conclusions: To be added at a later date.

Poster Number: 118

Time: Monday, May 16, 2016, 2:30 PM – 3:25 PM

How Is Gray Literature Used in Horizon Scanning Reports on Medical Devices?

Kelly Farrah, AHIP, Research Information Specialist, Information Services, CADTH, Ottawa, ON, Canada; Monika Mierzwinski-Urban, Information Specialist, Information Services, Information Services, Ottawa, ON, Canada

Objectives

Horizon scanning is a process used to identify and monitor new and emerging health technologies. Often, scant literature on these cutting-edge technologies exists in bibliographic databases. This project investigated the use of grey literature in horizon scanning reports on non-drug medical technologies, including: how often it is cited and which sources are most frequently cited.

Methods

A retrospective review of horizon scanning reports on non-drug medical technologies, including medical devices, laboratory tests, and procedures was conducted. A random sample of 22 reports was selected from a compilation of 130 reports published in 2014 by major international horizon scanning services and health organizations. For all reports, the percentage of grey literature references cited compared to bibliographic references was calculated. For each grey literature reference cited, the source of the reference was recorded. Additionally, each grey literature reference was classified by type using pre-determined categories. The total number of times a source was cited in the bibliographies of all the reports was computed. The most frequently cited sources in each category type will be used to recommend key websites for grey literature searching on new and emerging non-drug medical technologies.

Results

On average, 48% (299/617) of the references listed in the bibliographies of the horizon scanning reports reviewed were grey literature. The three most frequently cited types of grey literature were information from manufacturers (29% of all grey literature references, including manufacturers' press releases), regulatory agencies (9%), and clinical trial registries (9%). The U.S. Food and Drug Administration (FDA) and Clincaltrials.gov were the most frequently cited specific sources, comprising 7% and 9% of grey literature references respectively. There was great diversity amongst all other sources cited in the horizon scanning reports, with each appearing with a frequency of 2% or less in the bibliographies of all reports.

Conclusions

Grey literature represents a large proportion of references cited in horizon scanning reports on non-drug medical technologies. Approximately a third of

grey literature references originated from the manufacturers of these technologies. Almost half of the grey literature cited came from three sources: the manufacturers, Clinicaltrials.gov, and the FDA. Due to heterogeneity in the other grey literature sources cited, it would be difficult to create one standard checklist of key websites for identifying grey literature across all types of non-drug technologies. Further research is needed to examine the context in which grey literature is used within horizon scanning reports.

Poster Number: 124

Time: Monday, May 16, 2016, 2:30 PM – 3:25 PM

Piloting an Online Evidence-Based Practice Course for Nurses

Kim Mears, AHIP, Scholarly Communications Librarian; Lindsay Blake, AHIP, Clinical Information Librarian; Robert B. Greenblatt, MD Library, Augusta University, Augusta, GA

Objectives: The health sciences library collaborated with the Nursing Shared Governance Evidence-Based Council of a hospital system to create an online Evidence-Based Practice (EBP) continuing education course designed to foster interest in research and encourage the design and execution of EBP projects that could be completed in nursing units.

Methods: The Evidence-Based Council administered a validated assessment survey (Melnyk, 2012) to measure interest and knowledge of EBP among nurses in the hospital. A majority of respondents indicated interest in participating in online training in EBP principles and learning the practical applications for applying these principles to patient care. Librarians and nursing faculty adapted previously established curriculum and hosted the course in the institution's online learning management system. The course includes seven modules covering such topics as asking clinical questions, acquiring evidence, appraising evidence, and evaluating outcomes. Participants are allowed four weeks to complete all course activities. A pre and posttest design measures change in nurses' knowledge of EBP. The course instructors also completed paperwork to qualify the course for Georgia Nurses Association (GNA) continuing education credit.

Results: The course was piloted in August 2015. Twenty-nine nurses from the hospital registered for the pilot course to earn GNA credit. Feedback from the nurses that completed the course included the need for more time to complete the course, computer access difficulties, and challenges with the delivery format. Changes to the course were made to improve content and participant experience. The free online course is now offered to the nursing staff on a recurring basis.

Conclusions: It is expected that administering the course in an online format will allow more nurses to complete the course and learn EBP principles. The course instructors intend to investigate if an increase in EBP research projects occurs within nursing units as a result of the course.

Poster Number: 126

Time: Monday, May 16, 2016, 2:30 PM – 3:25 PM

Leveraging Development to Build a Foundation for the Future

Kristi L. Holmes, Library Director, and Associate Professor of Preventive Medicine, Health and Biomedical Informatics Division; Margarita Chung, Associate Department Administrator; Galter Health Sciences Library, Chicago, IL

Objectives: The X Library at Y University successfully established and fostered a relationship with our Office of Development to better advance a community of support for the library. This effort was originally initiated to support a large-scale renovation of library facilities and was expanded to better connect with key stakeholder groups and grow support for library initiatives.

Methods: The X Library is fortunate to have existing endowment funds which enable us to see first-hand their significant impact on library resources and services. The funds had not been actively cultivated for over 20 years, providing an excellent opportunity for new growth. Initial contact with the Office of Development was made by the library through email and presentations at key campus leadership meetings to highlight the upcoming library renovation and several new key library initiatives. A development officer was assigned to the library and together, the library and Office of Development have established a good working relationship and a series of activities to grow financial support of library space and initiatives. Better communication and productive collaboration with the Office of Development has been a multi-phase effort and a critical component of this successful initiative.

Poster Number: 129

Time: Monday, May 16, 2016, 2:30 PM – 3:25 PM

Connecting the Libraries and Athletics Through Information Literacy Instruction to Strength and Conditioning Interns

Lara Sapp, Health Sciences & Kinesiology Librarian; KT Vaughan, AHIP, Director, Rose Library; James Madison University, Harrisonburg, VA

Objectives: Explore a new relationship between the libraries and athletics by connecting the Health Sciences Librarian and the Strength & Conditioning Program at a mid-sized, public university.

Methods: Development and inclusion of a problem-based, information literacy instruction session into the didactic training of interns. The session focused on locating and searching the scholarly literature and understanding the foundational concepts of evidence-based practice. To support the learning and research needs of interns, a collection of core journals and books was identified and developed. Access to these resources is facilitated through a guide tailored to the interdisciplinary nature of this population. The methods used closely parallel those used for traditional outreach to a new academic program, but were adapted to the unique setting in University Athletics.

Results: This project is an example of extending library liaison services to a non-academic unit on the university campus. Outcomes include additional opportunities to connect with students outside of courses, including requests for instruction, collection purchases, and facilities use from other University Athletics departments and teams.

Conclusions: Extending library outreach and support to non-academic units is an opportunity for broader collaboration, thus increasing the library's presence across campus. This blending of research and practice brings together the two halves of undergraduate life – academics and athletics – in a novel way for this university.

Poster Number: 140

Time: Monday, May 16, 2016, 2:30 PM – 3:25 PM

Looking Back with Pride--Looking Forward with Purpose: Researching the History of an Osteopathic University

Lori Fitterling, Digital Services/Reference Librarian; Marilyn J. DeGeus, Director of the Library; Robyn Oro, Cataloger/Serials Assistant; D'Angelo Library, Kansas City, MO

Objectives: Osteopathic medical education has played a vital role in medical practice. This project illustrates the historical impact of one university as it approaches its centennial year. Today it is recognized as the 5th oldest osteopathic university in the country, the 10th largest medical school in the US, and proudly claims 60% of practicing DOs in its state, as alumni.

Methods: This project highlights events that demonstrate the impact one university has had on the history of the Osteopathic profession. By examining artifacts, historical documents, Osteopathic communications, and various archival materials, items that best represent the campus community for the past 100 years have been selected for use in this initiative. The library worked to restore important photographs and documents, digitize early osteopathic literature and research early medical instruments to create a historical timeline of events which depicts a visual representation of the medical impact this university's 10,000+ graduates have had on medical education.

Results:
The historical timeline clearly demonstrates the impact Kansas City University had, and continues to have, on the osteopathic profession and the Kansas City community. The visual display of people, places, documents, and events of a university with a remarkable heritage and a longstanding commitment to health, wellness, medical scholarship, and community service reflects a century of diversity. Historical highlights reflecting this diversity include photos of the founding board in 1916 consisting of twelve Doctor of Osteopathic Medicine physicians with four of them being women; Mamie Johnston, D.O., a woman, and the first graduate of our institution; the role Children's Mercy Hospital played in the location of the university as it is today; Harry J. Walter, D.O., the first Doctor of Osteopathic Medicine to be commissioned as a medical officer in the United States military, as well as the four name changes which took place over the last century.

Conclusions:

Kansas City University of Medicine and Biosciences has had a vital impact on medical education, the osteopathic profession, and the Kansas City community as visually represented in this project.

Poster Number: 143

Time: Monday, May 16, 2016, 2:30 PM – 3:25 PM

Piecing Together an Online Nursing Journal Club

Madeleine Taylor, Madeleine Taylor, MLS, MA; Medical Librarian, St. Joseph's Healthcare System, St. Joseph's Healthcare System, Paterson, NJ; Patricia May, Director of Library Services, St. Joseph's Healthcare System, Health Sciences Library, Paterson, NJ; Kathy Faber, Clinical Nurse Leader, St. Joseph's Healthcare System, Nursing Administration, Paterson, NJ

Objectives: The Health Sciences Library and the Evidence-Based Practice and Research Nursing Council's chair, the Clinical Nurse Leader, collaborated with the Information Technology Department to create an online nursing journal club to provide nurses at the bedside an opportunity to regularly participate in literature review in order to develop skills in evidence-based practice and primary nursing research.

Methods: Each nursing division has an "Online Navigator" position. These navigators, with the assistance of the librarians, identify articles to be utilized in the journal club. The library provides articles to the navigators, who write questions based on the articles. An answer sheet is created by library staff for each article. The library staff then posts links to the article and the answer sheet to the hospital's intranet. The Clinical Nurse Leader works with the Education Department to apply for continuing education credits to encourage participation in the online journal club. Future plans include an online discussion board. The Clinical Nurse Leader, along with the Navigators, provides guidance in using the Iowa model of evidence-based practice. Based upon the knowledge acquired in participation, the nursing staff determines if there is sufficient evidence to create a pilot project for changing nursing practice.

Results: In the first two years, approximately 30 contact hours of CE was available to nurses, and over 1,000 hours were earned. 24 IRB-approved nursing research projects were initiated, and four nursing practice changes were implemented.

Conclusions: The program is successful. The online discussion board has become a reality. Nurses continue to participate in the journal club and discussions, and continue to design projects to change practice.

Poster Number: 149

Time: Monday, May 16, 2016, 2:30 PM – 3:25 PM

Human Profile of Academic and Medical Librarians: What We Do in the Shadows?

Mariana Lapidus, Associate Professor, Reference Coordinator, Henrietta DeBenedictis Library, Henrietta DeBenedictis Library, Boston, MA; Samuel King, Library Manager, Assistant Professor of Library & Learning Resources, Manchester Campus Library, Manchester Campus Library, Manchester, NH

Objectives: - To identify top non-work activities and interests of academic and medical librarians in the U.S. - To compare the findings to such benchmark indicators as U.S. Census Bureau and Bureau of Labor Statistics data on leisure activities of adult population in the country to bridge the gap in knowledge about librarians' social profile and improve the public perception of this profession.

Methods: Unlike some other professions, physicians being just one example, professional librarians have been frequently surveyed on their expertise, services and work activities rather than on their personal interests. This study utilizes the survey instrument, which will be designed to collect statistical data on leisure time, hobbies and interests of academic and medical librarians, as well as their pet ownership. The survey will be administered electronically via MLA, NAHSL and other library listservs inside the U.S. The data will be then summarized and compared to the latest information found in the Statistical Abstracts of the U.S. (U.S. Census Bureau) and the American Time Use Survey (Bureau of Labor Statistics) in order to create the up-to-date portrait of the information professional supported by real life numbers instead of anecdotal evidence.

Results: The online survey was completed by 533 librarians, the vast majority of them being from New England and South Atlantic regions. References services librarians represent 55% of the respondents. Such overwhelming responses allowed collecting a significant amount of important statistical data related to off-work activities and leisure interests of librarians and present it in graph and chart formats.

Conclusions:
The data collected by this survey demonstrated that librarians are information professionals with extremely wide variety of interests and talents. Most people working in the library field are regularly involved into arts, music and sports activities. Other conclusions will be provided within the poster.

Poster Number: 151

Time: Monday, May 16, 2016, 2:30 PM – 3:25 PM

Supporting the Search for Alternatives to Painful and/or Distressful Procedures

Mary W. Wood, Librarian, Carlson Health Sciences Library, Carlson Health Sciences Library, Davis, CA

Objectives: USDA inspections identify lapses in Animal Welfare Act compliance, including insufficient proof of consideration of alternatives. The inspections and citations for non-compliance are at the level of the IACUC and DVM inspector, who are following USDA policies. Among the numerous regulations required by the Principal Investigator is USDA Animal Care Policy 12: Consideration of Alternatives to Painful/Distressful Procedures.

Methods: Research and publication remain the foundation of biomedical advancement. Compliance with regulations, guidelines, and standards of practice is necessary in order to continue receiving research funds and to publish. One small piece of the biomedical regulatory puzzle is the search for alternatives to the proposed use of animal models, with an emphasis on recognizing and addressing potential pain or distress.

Biomedical, veterinary, and health sciences librarians have been increasingly involved with their institutions' IACUCs and in offering support to their research faculty with their alternatives searches. While any librarian working with biomedical research is capable of helping their researchers, the specificity of the policies and the expectations of the reviewing bodies require background and instruction. Historical perspective, an overview of the regulatory landscape, database suggestions, and search strategies would be valuable to the librarian working in this area.

Results: The UC Davis Center for Animal Alternatives Information has been supporting UC Davis researchers with their alternatives search compliance since 1997. Longtime membership on the campus IACUC and participation on campus laboratory and animal facility inspections allows for a comprehensive understanding of the related issues and concerns. A resource created in a common format to readily share insights, guidance, and suggestions would be invaluable for librarians considering establishing a service to support this cohort of scientists.

Conclusions: This poster will outline the design and content of the new Search for Alternatives libguide, as well as provide suggestions on outreach and further development.

Poster Number: 157

Time: Monday, May 16, 2016, 2:30 PM – 3:25 PM

DMPTool Customization for Researchers Writing Data Management Plans (DMPs)

Melissa A. Ratajeski, AHIP, Coordinator of Data Management Services, Health Sciences Library System, Health Sciences Library System, Pittsburgh, PA; Carrie L. Iwema, AHIP, Information Specialist in Molecular Biology, Health Sciences Library System, University of Pittsburgh, Pittsburgh, PA

Objectives: The Data Management Planning Tool (DMPTool) is a free resource allowing researchers to create, review, and share DMPs to meet funder requirements.

This poster will detail a librarian-led initiative for University of Pittsburgh to become a DMPTool partner institution and the efforts taken by librarians to brand the tool, customize a preexisting funder template, and promote DMPTool usage across campus.

Methods: In January of 2015 librarians from two administratively separate library systems on campus partnered to register University of Pittsburgh as a DMPTool partner institution.

Upon registration, librarians were able to customize the tool with institutional branding and contact information for librarians who could review DMPs. The preexisting template for the NIH Genomic Data Sharing (GDS) Policy was the first to be customized by the librarians with the inclusion of links to institutional specific resources and boilerplate suggested responses. Librarians consulted with a number of data management stakeholders across campus including those from the Office of Research, Computing Services, Clinical and Translational Science Institute, and Institutional Review Board during the customization process. To promote the usefulness of the customized tool librarians have held targeted training sessions on using the DMPTool, written library newsletter articles, and advertised at the university's annual science symposium.

Poster Number: 162

Time: Monday, May 16, 2016, 2:30 PM – 3:25 PM

"Safe, Effective, and Potent": Conquering Polio at the University of Michigan School of Public Health

Merle Rosenzweig, Informationist; Kate Saylor, Health Sciences Informationist; Taubman Health Sciences Library, Taubman Health Sciences Library, Ann Arbor, MI

Objectives: The story of the eradication of polio in the United Stats is well known. However, the role played by the University of Michigan School of Public Health (UMSPH) is not as familiar. The poster highlights the achievements of the UMSPH in carrying out one of the most successful vaccination campaigns in history, which eventually led to the conquest of polio.

Methods: We gathered information for this poster from various resources including: the March of Dimes Foundation's Archives; the News Archives of the University of Michigan School of Public Health; the University of Michigan Bentley Historical Library, the Archives of the University; the papers of Jonas Salk held at the University of California, San Diego, Mandeville Department of Special Collections; and the "Evaluation of the1954 Field Trial of Poliomyelitis Vaccine: Final Report".

Results: In the United States, a polio epidemic in 1952 involved almost 60,000 cases. Thousands of children were paralyzed, many were confined to crutches or wheelchairs for life, and others, whose breathing muscles were paralyzed, were placed inside a sealed ventilator chamber known as an iron lung. In 1954, the U.S.Vaccine Advisory Committee approved a field test of Dr.Jonas Salk's polio vaccine. Under the direction of Dr.Thomas Francis, Salk's mentor during a fellowship at UMSPH, a massive controlled field trial was launched. Almost two million children in the United States, between ages of six and nine, participated.

Conclusions: On April 12, 1955, in the University of Michigan's Rackham Auditorium, Dr. Thomas Francis Jr., director of the Poliomyelitis Vaccine Evaluation Center at the University of Michigan School of Public Health, announced to the world that the Salk polio vaccine was up to 90% effective in preventing paralytic polio. The Salk polio vaccine transformed a disease that once horrified America into a memory.

Poster Number: 166

Time: Monday, May 16, 2016, 2:30 PM – 3:25 PM

Diverse Voices: Enlisting Stakeholders in User Needs/Usability Testing to Inform Redesign of HealthReach, a Multilingual Health Information Website from the National Library of Medicine

Michael Honch, Analyst, ICF International, Rockville, MD, ICF International, Rockville, MD, Bethesda, MD; Laura Bartlett, Technical Information Specialist, National Library of Medicine, Specialized Information Services, Outreach and Special Populations, Bethesda, MD; Carrie Gould-Kabler, Director of Community Outreach, Center for Public Service Communications, Center for Public Service Communications, Claiborne, MD; Gale Dutcher, AHIP, Deputy Associate Director, Specialized Information Services, National Library of Medicine, Bethesda, MD; Janice E. Kelly, Chief, Outreach and Special Populations Branch, Specialized Information Services, Specialized Information Services, Bethesda, MD; John C. Scott, President, Center for Public Service Communications, Center for Public Service Communications, Claiborne, MD

Objective:

HealthReach is the National Library of Medicine portal to quality multilingual and multicultural health resources. Once for refugee service providers, the portal now serves anyone assisting limited English-proficiency individuals. A needs assessment and test of the portal's usability was performed, enlisting refugee health coordinators, healthcare professionals, and information professionals. The objective was to determine the requirements of our expanded stakeholders, informing the redesign.

Methods:

To determine needs of new stakeholders and test usability, the HealthReach team developed a study enlisting diverse professional participants. Participants were 5 refugee health coordinators who were long term project partners, 5 health care professionals, and 4 public librarians that are new stakeholders. The study used mixed methods of usability tasks and interviews about user needs during a one-on-one, one-hour session held online. Participants performed a series of heuristic tasks based on study questions about portal content, features, and search functionality, unique to each user group, and portal use in professional settings. Results were collected, analyzed, and prioritized for their insights about enhancing the layout, navigation, terminology, and search interface for all stakeholders.

Results:

The HealthReach team collected qualitative data about participants' experience and their ability to use the portal effectively. This data informed development of the portal redesign requirements to streamline and simplify search features,

improve display of search results and document properties pages, and enhance awareness of consumer health information and provider tools on HealthReach.
Conclusion:
The redesigned and enhanced HealthReach portal at https://healthreach.nlm.nih.gov/, with new responsive versions for tablet devices and mobile phones, will be available April 2016.

Poster Number: 167

Time: Monday, May 16, 2016, 2:30 PM – 3:25 PM

Library Services and Cultural Competency in Health Professions Education and Patient Care

Misa Mi, AHIP, Associate Professor and Medical Librarian, Medical Library, Medical Library, Rochester, MI; Yingting Zhang, AHIP, Information & Education Librarian, Robert Wood Johnson Library of the Health Sciences, Rutgers University, New Brunswick, NJ

Objectives: This study will be conducted to investigate how health sciences libraries provide services to lead, promote, and support initiatives in cultural competence in health professions education or patient care and to examine health sciences librarians' opinions on cultural competence in relation to library services and professional development. The study will serve as needs assessment for our ultimate goal: using the study results to make informed decisions on developing and offering future continuing education opportunities on cultural competency for health sciences librarians. The research findings will contribute to our understanding what types of library services are provided to develop or support cultural competency initiatives and how health sciences librarians perceive cultural competence for librarians. The results will be useful for developing future continuing education courses tailored to health sciences librarians in developing or enhancing their cultural competency and expanding library services to support any cultural competency initiatives in health professions education or patient care in their own institutions.

Methods: This will be a quantitative research design. Data will be collected with a survey questionnaire. The questionnaire is developed to include 16 items eliciting demographic information, addressing the status of health sciences libraries in provision of culturally competent services and librarians' perceptions of the importance of cultural competence. Participants will be health sciences librarians. The questionnaire will be pilot tested with a convenience sample of health sciences librarians to enhance the content validity of the questionnaire. After the questionnaire is revised, its online version via SurveyMonkey will be administered to subscribers of the MEDLIB-L listserv of the Medical Library Association. Descriptive statistics (e.g., mean, standard deviation, correlations) will be used to analyze data collected from the survey.

Poster Number: 170

Time: Monday, May 16, 2016, 2:30 PM – 3:25 PM

Determining the Mosaic of Information Usage Habits of Faculty and Staff

Nakia Woodward, Sr. Clinical Librarian, Quillen College of Medicine Library, Quillen College of Medicine Library, Johnson City, TN; Rick Wallace, AHIP, Assistant Director, Quillen College of Medicine Library, Quillen College of Medicine Library, Bristol, TN; Rachel R. Walden, Associate Dean, Department of Learning Resources, Quillen College of Medicine, Quillen College of Medicine Library, Johnson City, TN

Objectives: The purpose of this study is to determine the actual information usage habits of faculty and staff at ** versus librarians perceptions and opinions.**

Methods: A prevalidated pilot tested survey will be conducted of the faculty and staff of the *** College of Medicine and ****College of Pharmacy. The survey will examine what are the most useful resources, specifically databases and journals, for their research and work. The results of the survey will be compared to the library's current collection and the research results will help serve as a basis for future collection development decisions. Library staff will also be surveyed to allow for a comparison between library staff perceptions and actual utility of the collection.**

Poster Number: 173

Time: Monday, May 16, 2016, 2:30 PM – 3:25 PM

NOVEL's Next Chapter: Piecing Together a Sustainable Product

Nancy T. Lombardo, AHIP, Associate Director for Information Technology; Christy Jarvis, AHIP, Head of Information Resources and Digital Initiatives; Spencer S. Eccles Health Sciences Library, Spencer S. Eccles Health Sciences Library, Salt Lake City, UT

Objectives: Explore sustainability options for a professional society's digital repository
Curate and organize repository content into an added-value product for licensing
Refine the collaboration and coordination of expertise of librarians and society members

Methods: This project involves librarians working with a society to find a strategy to provide financial sustainability for an open access digital repository. In order to create a revenue generating product, librarians work with society members to select, peer-review and organize learning objects into the discipline's structured professional training objectives. Following a curriculum outline developed by the society, librarians identify areas for collection development and target contributions from society members. The completed outline will provide added-value to the open access collection leading users to the highest quality objects and guiding them through the content in a logical sequence to enhance learning. The final product will have the potential to be licensed to libraries, residency programs and health sciences institutions providing a sustained revenue stream to support the project for years to come.

Poster Number: 182

Time: Monday, May 16, 2016, 2:30 PM – 3:25 PM

Developing a Replicable Methodology for Automated Identification of Emerging Technologies in Health Care

Patricia F. Anderson, Emerging Technologies Informationist, Taubman Health Sciences Library, Taubman Library, University of Michigan, Ann Arbor, MI; Skye Bickett, AHIP, Reference and Education Librarian, Library, Library, Suwanee, GA; Joanne Doucette, Assoc. Dir. Univ. Libraries; Assoc. Prof., Library and Learning Resources, Library and Learning Resources, Boston, MA; Pamela Herring, AHIP, Electronic Resources Librarian, Harriet F. Ginsburg Health Sciences Library, Harriet F. Ginsburg Health Sciences Library, Orlando, FL; Andrea C. Kepsel, AHIP, Health Sciences Educational Technology Librarian, MSU Libraries, East Lansing, MI; Tierney Lyons, Librarian, ., ., Honesdale, PA; Scott McLachlan, Librarian, Oxford Institute for Energy Studies, Library, Oxford, United Kingdom; Lin Wu, AHIP, Instructional Associate Professor and Pharmacy & South Health Science Center Campus Librarian, Texas A&M University, Medical Sciences Library, Kingsville, TX

Objectives: For the MLA systematic review projects, Team 6 on Emerging Technologies was seeking a replicable and authoritative methodology to mine Pubmed for emerging technologies relevant to health librarians in North America. Organizations working in this space, such as the Gartner Group and the New Media Consortium, either have no formal methodology or do not share their strategy publicly. Thus, it was necessary to create a methodology for this purpose that can be used by MLA now and in the future.

Methods: Creation of a search strategy involved identifying appropriate free text and MeSH terms for three concept groups (emerging technologies, emerging, and technology), then testing these individually and in combination for sensitivity and specificity. Each concept group was enriched with specific journal titles with high specificity for the topic. Inclusion/exclusion criteria were identified for the level of specificity required for terms and titles. Concept groups were combined as: ["Emerging Technologies" OR (Emerging AND Technologies)]. The search was limited to the most recent 3 years and English language. The data set for analysis was composed of titles and abstracts from the search results. Analysis was performed with text mining software to identify "hot spots" of particular interest.

Results: [in process]

Conclusions: [in process]

Poster Number: 190

Time: Monday, May 16, 2016, 2:30 PM – 3:25 PM

Mosaic Marketing: Reaching Rural Circuit Hospitals

Rachel W. Becker, Circuit Librarian, Rochester Regional Health, Rochester, NY

Objectives: The purpose of the program was to create a library services marketing campaign at multiple, geographically distant health institutions with varying levels of connectivity.

The program took place at three rural hospitals in the greater Rochester NY area, served by a newly hired Circuit Librarian.

Methods: The Circuit Librarian met with physician, nursing and allied health leadership groups at each hospital to determine preferred communication outlets and resources, as well as remote access capabilities. Based on feedback from these meetings, the Librarian developed a unique, mixed-methods marketing methodology to advertise library services. Marketing techniques included a combination of digital, print and in-person approaches unique to each department and institution. Digital marketing consisted of email blasts, online newsletters and links to maintenance of certification materials. Paper promotion included introductory "meet your librarian" flyers, handouts on available resources, and business cards provided at classes, meetings and hospital events. Finally, in-person promotion involved brief introductory presentations at hospital leadership meetings, membership in hospital committees, and classes on using Library resources.

Results: In the eight months since hiring the Circuit Librarian, there were 100 search requests from the 3 Circuit hospitals, compared with 73 requests for the same eight-month period the prior year. The Librarian presented at nursing and physician leadership meetings at all three locations, participated in nursing grand rounds, joined one hospital's Magnet Steering Committee, and was invited to be a guest lecturer for the 'New Visions' program, a health careers exploration class for high school students. At the one year mark, the Circuit Librarian will distribute a survey to collect feedback on the strengths and limitations of the program.

Conclusions: By responding to the needs of individual user groups, the Circuit Librarian Program successfully implemented a mixed-methods marketing campaign at multiple rural hospitals in the upstate New York area. Future goals for the Circuit Program include conducting a needs assessment for a digital core reference collection at one hospital, increasing physician awareness and

utilization of the program at all locations, and expansion of off-site access to electronic Library resources.

Poster Number: 193

Time: Monday, May 16, 2016, 2:30 PM – 3:25 PM

Andragogy and Information Literacy Instruction: A Survey of Medical Librarians

Rebecca McCall, AHIP, Director, Mercy College of Nursing and Health Sciences LIbrary, Southwest Baptist University, Springfield, MO

Objectives: Andragogy is the theory of adult education. Its focus is the learning process and learners' internalized needs. Andragogy provides best practices for adult information literacy instruction, but there is limited research on its use in medical librarianship. The purpose of this study is to better understand medical librarians' knowledge of, attitudes toward, and applications of andragogy in information literacy instruction.

Methods: After obtaining Research Review Board approval, a cross-sectional web-based questionnaire will be distributed to medical librarians via professional discussion lists. The survey will include questions on librarians' knowledge of and views on andragogy; their training in its use; any challenges to its application; rates of use; and demographics. After the survey closes the data will be analyzed using descriptive statistics to identify patterns in the use of andragogy in information literacy instruction by medical librarians.

Poster Number: 199

Time: Monday, May 16, 2016, 2:30 PM – 3:25 PM

Counting the Colorful: The Events Assessment Task Force

Roberta Bronson Fitzpatrick, Associate Director, George F. Smith Library of the Health Sciences, Newark, NJ; ; ; Zara Wilkinson, Reference and Instruction Librarian, Rutgers University, Camden, NJ; ;

Objectives: With the advent of RCM, Responsibility Center Management, as a new financial/budgeting model, fees assessed to schools or departments are more transparent. To demonstrate the value of the libraries beyond collections, an Events Assessment Task Force was formed to gather information about the non-instructional, more social, events hosted by the university libraries.

Methods: The Task Force began by establishing definitions for what constituted an "event". Members solicited suggestions for event types, such as art shows, Lego building contents, book clubs, exhibits, guest lectures, health information fairs, stress buster activities, and more. There was discussion about the information that was useful to capture for each event, such as number attending, co-sponsorship with another group, time/date/location. An online form was developed. It has drop-down menus which list event types, duration, and other common elements, which make recording easier. The data will be imported into a database capable of report generation. The database is modeled after one currently in use for collecting bibliographic instruction data and reflects both AAHSL and ARL needs At each step of the process, Task Force members solicited comments from library faculty and staff who were involved with various events.

Results: As of this writing, the Events Assessment Task Force presented both the data collection form and the events database to USC, the User Support Council. Both were approved. The next step is to conduct local training sessions to familiarize library faculty and staff with the form and the database in order to acquaint them with all involved procedures and to gain their participation in the data collection.

Conclusions: In the process of developing the online data collection form and the database, it was interesting to note the many types of events hosted by Rutgers University Libraries. By recording the added value that the libraries bring to the university, it may spark more collaborative ventures among members of the libraries staff and with other schools/departments within the university.

Poster Number: 201

Time: Monday, May 16, 2016, 2:30 PM – 3:25 PM

Mentoring When You Least Expect It: Working Across Institutions to Foster Professional Excellence

Robin M. N. Parker, Evidence Synthesis and Information Services Librarian, Dalhousie University, WK Kellogg Health Sciences Library, Halifax, NS, Canada; Katie D. McLean, AHIP, Librarian Educator BA, LIT, MLIS, Library Services, Nova Scotia Health Authority , Halifax, NS, Canada; Sarah M. Visintini, Evidence Synthesis Coordinator, Maritime SPOR SUPPORT Unit (MSSU) & Nova Scotia Cochrane Resource Centre Center for Clinical Research, Maritime SPOR SUPPORT Unit (MSSU) & Nova Scotia Cochrane Resource Centre Center for Clinical Research, Halifax, NS, Canada; Melissa Helwig, Information Services Librarian, W.K. Kellogg Health Sciences Library, W.K. Kellogg Health Sciences Library, Halifax, NS, Canada

Objectives:
Frequently, mentoring is framed as a relationship between senior and junior librarians. In our experience, becoming a mentor can happen when you least expect it, including when you are a relatively new professional. We will present our experiences of mentoring in early career and highlight some lessons learned for anyone on either side of a mentoring relationship.

Methods:
Mentoring has taken on many forms amongst our local professional colleagues - from providing advice to current students to taking the time to assist colleagues with professional growth. By highlighting collaborative professional opportunities including journal clubs, special interest groups, and peer review of research, a group of health librarians working in different contexts will showcase how we have engaged in mentorship both vertically and horizontally. With the support of our respective research, hospital, and academic workplaces, we have overcome institutional barriers to cooperate. Though we don't conduct formal evaluation, we track participation in journal club sessions and share mutually beneficial resources using technology such as Google Drive.

Results:
Using testimonials from participants, we present how our vertical and horizontal mentoring relationships have benefited our professional work in the following areas: research collaboration, continuing education opportunities, peer support, and in serving our patrons. We share activities and lessons learned that have allowed us to turn the small size of our professional community into a strength, and our experiences on both sides of the mentoring relationship.

Conclusions:

We challenge the belief that mentors must by definition be more experienced or well into their careers. It has been our experience that peers can often be untapped resources when developing skills or seeking expertise on projects. In the health sciences library and information environment in the Maritimes, collaboration and cooperation have optimized the productivity of our work, as well as provided us with professional development opportunities that might otherwise be unavailable or inaccessible. In addition to establishing a robust local network of professional support, this style of multi-directional mentoring amongst early career librarians creates efficiencies in teaching initiatives and support for cross-affiliated researchers.

Poster Number: 206

Time: Monday, May 16, 2016, 2:30 PM – 3:25 PM

Special Considerations in Podiatry Collection Development

Samantha N. Mosby, Library Assistant, Rosalind Franklin University of Medicine and Science, Chicago, IL; Scott Thomson, AHIP, Library Director, Boxer Library, Rosalind Franklin University of Medicine and Science, North Chicago, IL

Objectives: To outline special considerations in podiatry collection development due to the small size of the profession and unique qualities, such as long-term relevancy and rarity, of podiatry publications.

Methods: The library received several large donations of podiatry materials from alumni. These materials included books, journals, society bulletins, program booklets, and unbound monographs. Library personnel and current podiatry professors evaluated these materials to determine if they should be added to the collection. The decision to add or discard items was made based upon current library holdings and relevance to the field. Through the process of receiving and evaluating the donated materials, the library determined that collection development and weeding of podiatric materials needs to be handled differently than other health science fields.

Results: Due to the rarity and continued relevance of most of the donated materials, only books and journals which were already held by the library were not added to the collection in most cases. Older journals (1920s-1950s), society bulletins, and monographs were found to have very few, if any, holdings in the WorldCat OCLC catalog. Books on topics such as biomechanics of the foot, gait, and posture contain information that is still useful to students, despite their age. The majority of these publications are out of print and/or without electronic copies, and should thus be kept for the historical record.

Conclusions: Podiatry is a unique field within the health sciences, and podiatric materials require strict attention. Only nine schools in the U.S. grant D.P.M. degrees, and podiatric materials are therefore often printed in smaller quantities and can be rare. Older materials are especially likely to be rare and should be retained. Additionally, podiatric materials tend to age well and information in them often remains relevant for longer periods of time. Materials should not be weeded from collections without consulting podiatry professionals. Podiatry collection development necessitates special research, consultation, and evaluation to ensure the health of the collection.

Poster Number: 209

Time: Monday, May 16, 2016, 2:30 PM – 3:25 PM

"But I Already Know All of This!" Utilizing a Pre-Class Assessment to Customize Library Instruction

Sarah E. Katz, Senior Assistant Librarian/ Health Sciences Librarian, University of Delaware Library, University of Delaware Library / Reference and Instructional Services, Newark, DE

Objectives: To determine if utilizing a pre-class questionnaire to design a customized library instruction session best addresses gaps in interest, knowledge, and skill among nutrition students in a one-time, two-hour library instruction session.

Methods: In a two-hour, one-time library instruction session, senior undergraduate nutrition students arrive with varying interests, levels of knowledge, and skill. By implementing a pre-class questionnaire, the hypothesis is that the instructor can better gauge student opinions regarding these competencies. As a result of the pre-class questionnaire, a customized instruction session will be designed in response to both the learning objectives set for the library instruction session as well as the needs and questions expressed by students in the questionnaire. Following the customized instruction sessions, students will be asked to respond to post-class evaluation questions to determine whether the customized instruction session best addressed gaps in the competencies identified above. In this poster, student pre and post-class evaluations, learning outcomes, and feedback will be discussed and compared with those sessions where pre-class questionnaires were not distributed.

Results: Detailed results will be available when the poster is uploaded and presented at the 2016 MLA Conference in Toronto in May 2016.

Conclusions: Implementing a pre-class assessment resulted in more engaged students. While the material presented did not ultimately change much from what was presented in sessions prior to the pre-class assessment, students were more involved in the session because the topics and material they requested was covered. In addition, the pre-class evaluation prevented students from commenting that "they already learned this" during the session as they asked for the material to be covered in the pre-class assessment and the session was designed to fit their needs. A more in-depth conclusion will be presented at the 2016 MLA Conference in Toronto in May 2016.

Poster Number: 215

Time: Monday, May 16, 2016, 2:30 PM – 3:25 PM

Designing an Interactive Web-based Tutorial for Health Sciences Students: A Collaborative Library Project

Sharon Leslie, AHIP, Public Health and Health Sciences Librarian, University Library, Research and Engagement, Georgia State University Library, Atlanta, GA; Eric Willoughby, Web Programmer, Georgia State University, University Library, Digital Library Services, Atlanta, GA; Jennifer Jones, Assessment & User Experience Librarian, Georgia State University, University Library, Library Administration, Atlanta, GA; Tazar Gissentanner, Library Instructional Support Coordinator, Georgia State University, University Library, Research and Engagement, Atlanta, GA

Objectives: To create compelling, interactive Web-based tutorials for academic students in the health sciences to strengthen their research skills, as well as offering library faculty instructors a user-friendly tutorial builder template.

Methods: “Guide on the Side” is a free, open-access software tutorial tool developed by the University of Arizona libraries that uses active “live” content via a Web browser. A subject librarian worked with the library’s web programmer to design a more visually interesting template than the original ‘out of the box’ application with (redacted)-branding. The librarian created content, interactive exercises and quiz questions for a ‘Basic Searching in PubMed’ tutorial. The library’s instructional support coordinator suggested design, graphics and language revisions. Five students were then recruited by the library’s assessment librarian to evaluate the tutorial. The students completed a pre-usability study with scripted questions and verbal follow-up to gauge users’ perceptions of the overall ease-of-use of the format, navigation, content, and stumbling blocks particular to the PubMed tutorial. The tutorial was revised based on the input received and implemented.

Results: The open-access Guide on the Side application was found to be a good choice for self-producing tutorials and templates. It is more user-friendly than Camtasia or Captivate software, potentially reducing institutional costs for licenses; tutorials and templates can be easily updated, eliminating laborious revisions of recorded efforts. Additionally, using an interactive system may increase student’s learning comprehension as immediate feedback is offered, based on the answers to the exercises. (Redacted) library faculty now have a tested template they may use to create tutorials for their own subject disciplines.

Conclusions: The collaboration among library faculty and staff was essential to creating a unique platform for subject librarians to deliver active-learning instruction activities. Student assessment and promotion of the final tutorial is underway; outcomes are forthcoming.

Poster Number: 218

Time: Monday, May 16, 2016, 2:30 PM – 3:25 PM

Developing a Database of Critically Appraised Systematic Reviews in the Field of Pain Management

Shelley A. M. de Kock, Information Specialist, Kleijnen Systematic Reviews Ltd, Information Team, York, N/A, United Kingdom; Marie Westwood, Reviews Manager, Kleijnen Systematic Reviews Ltd, Reviews, York, United Kingdom; Kate Misso, Information Specialist Manager, Kleijnen Systematic Reviews Ltd, Information Team, York, United Kingdom; Lisa Stirk, Information Specialist, Kleijnen Systematic Reviews Ltd, Information Team, York, United Kingdom; Sohan Deshpande, Reviewer, Kleijnen Systematic Reviews Ltd, Reviews Team, York, United Kingdom; Jeanette Kleijnen, Office Manager, Kleijnen Systematic Reviews Ltd, KSR Ltd, York, United Kingdom; Antony McLellan, IT Manager, Kleijnen Systematic Reviews Ltd, KSR Ltd, York, United Kingdom; Jos Kleijnen, Director, Kleijnen Systematic Reviews Ltd, KSR Ltd, York, United Kingdom

Objectives: To identify systematic reviews on pain management from a comprehensive range of resources, in order to build a database of critically appraised systematic reviews (SRs) with clinical bottom-lines to support clinical practice and patient care.

Methods: Information Specialists developed a sensitive strategy and SR filter to maximise recall of candidate references. Embase, MEDLINE, MEDLINE In-Process Citations, MEDLINE Daily Update, Cochrane Databases of Systematic Reviews (CDSR), PsycINFO, Allied and Complementary Medicine Database (AMED), Database of Abstracts of Reviews of Effects (DARE) and CINAHL were searched to retrieve systematic reviews on pain and pain relief management. Searches were limited from 2010 onwards. Results were downloaded, and duplicates removed using EndNote X6. Experienced Information Specialists sifted results to remove non-SR records, reviews of reviews, reviews of guidelines and non-pain records. Reviewers then critically appraised each review, using an adaption of the risk of bias ROBIS tool. For each review, an overall risk of bias, summary and clinical bottom- line statement was written.

Results: 87% of SRs submitted for critical appraisal came from MEDLINE, Embase and CDSR. 30% of results came from databases other than MEDLINE and Embase. Of the SRs which have been critically appraised so far by reviewers, 62% are of high risk of bias while 7% are unclear.

Conclusions: Searches of multiple sources are essential to ensure comprehensive retrieval of SRs on pain as relying only on MEDLINE and Embase could mean a substantial 30% of SRs are missed. The large numbers of

SRs deemed to be of high risk of bias or unclear, according to the ROBIS tool, is of concern for clinical decision makers.

Poster Number: 221

Time: Monday, May 16, 2016, 2:30 PM – 3:25 PM

Scholarly Projects: The Librarian's Role in Undergraduate Medical Education Curriculum Design

Stephanie Kerns, Associate University Librarian; Tracy Dana, Clinical and Outreach Librarian; Oregon Health & Science University, OHSU Library, Portland, OR

Objectives: To describe the collaboration between the subject faculty and the liaison librarians in creating the informatics curriculum in Scholarly Projects, a capstone component of the curriculum, and to compare the Scholarly Projects Proposal course, taught to the first cohort in Fall 2015, to the second cohort, taught in Winter 2016.

Methods: The undergraduate medical education (UME) curriculum at a large urban research university recently underwent a curriculum transformation. The new curriculum was implemented with the first cohort of students (MED18) in August 2014. One component of the curriculum is a longitudinal, multi-year capstone project called the Scholarly Project. As designed, it will begin in the first year and be completed in the final year. Librarians are assigned by subject specialty to groups of students depending on their area of interest. The first piece of the Scholarly Projects curriculum is a proposal course designed to allow the students to create their proposal in a structured fashion. As this course is taught to the second cohort of students (MED19), librarians will make improvements based on feedback gathered from the MED18 students and faculty in the first course.

Poster Number: 223

Time: Monday, May 16, 2016, 2:30 PM – 3:25 PM

Opportunities for Librarians in Enhancing Integration in the Medical School Curriculum

Stephanie M. Swanberg, AHIP, Assistant Professor, Information Literacy & eLearning Librarian , Medical Library, Oakland University William Beaumont School of Medicine, Rochester, MI; Keith Engwall, AHIP, Assistant Professor, Web & Emerging Technologies Librarian, Medical Library, Oakland University William Beaumont School of Medicine, Rochester, MI; Robin Rivest, Director of Curriculum Data Management, Oakland University William Beaumont School of Medicine, Office of Medical Education, Rochester, MI

Objective:
The integrated curriculum model in medical education strives to promote medical students' retention of knowledge and skills by integrating basic science and clinical content across all years of the medical school curriculum. Librarians' skills in information searching, organization, and data analysis can enhance the evaluation of integration in the curriculum. The challenge lies in demonstrating our value to curriculum stakeholders.

Methods: Librarians at a newly fully accredited medical school hold faculty positions in the school of medicine, serve on standing committees, and participate in curriculum planning, review, and reform. At least one librarian serves on all of the school's curriculum committees, including as chair of the curriculum integration subcommittee (CIS). The charge of the CIS is to continually analyze and provide recommendations on how to enhance the integration of the school's curriculum. This poster will describe the specific contributions of librarians to curriculum integration, such as developing relationships with the Office of Medical Education; conducting literature searches to discover curricular topic standards; investigating graph databases for curricular analysis; and creating an automated process for sorting and linking course learning objectives to institutional competencies. Recommendations for other librarians on how to increase their participation in curriculum integration will also be covered.

Results: Through work on the CIS, librarians have established their place in contributing to the successful integration of the curriculum. Major outcomes have included: 1) the successful mapping of all course and clerkship objectives to the medical school competencies using an automated process developed by a librarian; 2) piloting the Integration Topic Reports project to develop reports outlining how a particular topic is currently taught in the school curriculum, how this compares to other schools, and an accompanying literature review; 3) testing the use of graph databases to map intricate relationships between content in the curriculum and those teaching it.

Conclusions: Librarians are already recognized for their contributions to data management for research purposes, so it is natural that we also have a role in data management and analysis for curricular purposes including integration.

Poster Number: 224

Time: Monday, May 16, 2016, 2:30 PM – 3:25 PM

A Journal Club Tech Tool for Clinicians

Susan Baer, Director of Libraries & Archives, Regina Qu'Appelle Health Region, Regina General Hospital, Health Sciences Library, Regina, SA, Canada; Amy Weisgarber, Library Technician, Health Sciences Library, Regina Qu'Appelle Health Region, Regina, SA, Canada; Michelle Dalidowicz, Client Services Librarian, Regina Qu'Appelle Health Region, Health Sciences Library & Archives, Regina, SA, Canada; Robyn Hocking, Client Services Librarian, Regina Qu'Appelle Health Region, Health Sciences Library, Regina, SA, Canada

Objectives: Participating in a journal club is valuable for engaging in spirited discussions, refining critical appraisal techniques and assessing the applicability of the article to clinical practice. No web-based tool exists to facilitate journal club documentation. Two clinical departments within Regina Qu'Appelle Health Region expressed a need for a tool to capture the rich discussions and relevance of the article in practice.

Methods: An extensive literature search uncovered only one relevant article on developing a web-based tool to organize journal club materials. Library staff identified the functionality and features needed for an ideal website and developed a checklist to evaluate potential platforms to test. A prototype was built using WordPress for a librarian journal club. Multiple tests and revisions were conducted. A test Pediatricians' journal club site was implemented and feedback was sought from the physicians. An online survey was conducted to gauge the number of journal clubs in the Regina Qu'Appelle Health Region.

Results: The Pediatricians' journal club members were very responsive to the website created. Their executive assistant was integral in maintaining and populating the site for the physicians. The Pediatricians agreed to use the site for one year, when a further evaluation of the effectiveness of the site and any maintenance issues will be reviewed. The results of the region-wide survey indicated that there are more journal clubs in existence than originally anticipated. The majority of the respondents felt their critical appraisal skills could be improved.

Conclusions: The feedback from the Pediatricians regarding their use of the journal club website will help to determine how extensively the template can be shared with other journal clubs in the Health Region. The Pediatricians felt that having a site where the articles reviewed by the journal club with their assessments to practice in the Pediatrics and Neonatal Intensive Care Units will be useful for new staff, visiting physicians, interns and residents. The results of the survey indicate that regular critical appraisal instruction may be a valuable

support for a variety of clinicians, including physicians, pharmacists, dietitians, and nurses.

Poster Number: 227

Time: Monday, May 16, 2016, 2:30 PM – 3:25 PM

Retention and Reward of Volunteer Clinical Faculty: Collaborative Librarian Outreach

Tiffany Moxham, Coordinator of Medical Library Programs, Medical Library Services Unit, Orbach Science Library, Medical Library Services Unit, Orbach Science Library, Riverside, CA

Objectives: Volunteer clinical faculty retention is one of the long-term challenges of community-based medical schools. Library services and resources access is one of the positively associated extrinsic benefits of retaining faculty status. The aim of the program was to develop collaborative methods to implement a sustainable, outreach program across multiple sites with no additional personnel.

Methods: Programmatic needs were determined by assessment of current volunteer resource access and the results of a faculty survey. Forty percent of all faculty requested library-related faculty development as a top priority. Staged implementation of initial outreach was developed based on curriculum areas, current preceptors, departmental priorities, and individuals/groups with minimal outside institutional resources. Partnerships were formed with the Faculty Development, longitudinal clinical experience, and clerkship teams. In-person joint introductory sessions rotating through multiple hospital and group sites were paired with dissemination of brochures by collaborators. Clinical departments and groups were then targeted for longer, focused, training sessions. To ensure maximum reach, first year students were invited to participate in providing brochures and information at their first preceptor visit. Outreach was increased exponentially across different departments, locations, and specialties. Technology support was incorporated to include videos, guides and tutorials.

Results: IRB approved data collection of number, types, method of contact plus location and specialty.

Conclusions: My results are not complete yet.

Poster Number: 230

Time: Monday, May 16, 2016, 2:30 PM – 3:25 PM

If a Picture Is Worth a Thousand Words, What Is the Value of 290 Historical Pictures? The Vital Role of Volunteers in Doing the Math

Tracy C. Shields, AHIP, Reference Medical Librarian; Jane A. Pellegrino, AHIP, Department Head; Lisa R. Eblen, AHIP, Associate Department Head; Naval Medical Center, Portsmouth, Library Services, Portsmouth, VA

Objectives: To inventory the original hospital's pictures and provide captions of historical context.

Methods: When the first naval hospital building, originally built in 1830, was renovated in 2002, the building was decorated with historical photographs. Part of the renovation was an inventory of hung pictures. These framed, reprinted photographs document much of the history, both medical and military, of the building and the institution. During the intervening years, the command historian – a volunteer position at this facility – researched and documented information on the pictures, and included local historical context. In early 2015, a project to update the original inventory was undertaken by the command historian and a second volunteer from the library.

Results: The two volunteers physically took inventory of 290 hung pictures in the building, wrote and edited captions for each, and formatted the captions for printing. With the assistance of and input from an area naval museum, the two volunteers have revised the captions and prepared them for printing for installation at each picture. The project was started, developed, and implemented by the two volunteers, with the support of the library's director and Command leadership who secured the funding to complete the project.

Conclusions: My results are not complete yet.

Poster Number: 233

Time: Monday, May 16, 2016, 2:30 PM – 3:25 PM

What Are the Outcomes Associated with the Use of Online Consumer Health Information in Primary Health Care? A Systematic Review with Framework Synthesis of Quantitative and Qualitative Evidence

Vera Granikov, Research-embedded health information specialist, McGill University, Family Medicine, Montreal, PQ, Canada; Reem El Sherif, Graduate Student, McGill University, Family Medicine, Montreal, PQ, Canada; David Li Tang, Affiliate, McGill University, Family Medicine, Montreal, PQ, Canada; Mathieu Bujold, Post-doc, McGill, Family Medecine, Montréal, PQ, Canada; Quan Nha Hong, PhD Candidate, McGill University, Family Medicine, Montréal, PQ, Canada; Cristiane Galvao, Professor, University of Sao Paulo, Ribeirao Preto Medical School, Ribeirao Preto, SP, Brazil; Carol A. Repchinsky, Special Projects Pharmacist, Canadian Pharmacists Association, n/a, Ottawa, ON, Canada; Jo-Anne Hutsul, Managing Clinical Editor, Canadian Pharmacists Association, n/a, Ottawa, ON, Canada; Lynn Dunikowski, Director of Library Services, College of Family Physicians of Canada, Library, London, ON, Canada; Francesca Frati, Librarian, Health Sciences Library, Health Sciences Library, Montreal, PQ, Canada; Linda Shohet, Executive Director, The Centre for Literacy, n/a, Montreal, PQ, Canada; Lillian Vineberg, Patient Representative, n/a, n/a, Montreal, PQ, Canada; Gillian Bartlett, Associate Professor, McGill University, Family Medicine, Montreal, PQ, Canada; Roland Grad, Associate Professor, Department of Family Medicine, Family Medicine, Montreal, PQ, Canada; Michael Shulha, PhD Candidate, McGill University, School of Information Studies, Montreal, PQ, Canada; Lorie Kloda, AHIP, Librarian, Concordia University Library, Montreal, PQ, Canada; Bernard Burnand, Physician in Chief, Professor, Lausanne University Hospital, Institute of social and preventive medicine, Lausanne, Switzerland; Sophie Desroches, Associate Professor, Université Laval, School of Nutrition, Faculty of Agriculture and Food Sciences, Quebec, PQ, Canada; France Legare, Full Professor, Université Laval, Family Medicine, Quebec, PQ, Canada; Florence Millerand, Professor, Université du Québec à Montréal, Département de communication sociale et publique, Montreal, PQ, Canada; Benoit Rihoux, Professor, Université catholique de Louvain, Centre de Science Politique et de Politique Comparée, Louvain-la-Neuve, Belgium; Isabelle Vedel, Assistant Professor, McGill University, Family Medicine, Montreal, PQ, Canada; Pierre Pluye, Full Professor, FRQS Senior Research Scholar, McGill University, Family Medicine, Montreal, PQ, Canada

Objectives

Systematic reviews in public health and oncology suggest the use of Online

Consumer Health Information (OCHI) improves knowledge, participation in health care, and health outcomes. The objectives of this systematic review are to revise a conceptual framework and list the types of patient health outcomes associated with the use of OCHI in primary health care.

Methods

We followed the four stages typically used in systematic reviews: identification of potentially relevant studies using a comprehensive search strategy; selection of relevant studies; quality appraisal of selected studies; and synthesis of retained studies. Eligibility criteria included: qualitative, quantitative, or mixed methods study; English or French; 1990-2014; primary health care; online consumer health information use. Four health librarians developed and peer-reviewed the search strategy. Searches were conducted in Medline, Embase, PsycINFO, CINAHL, LISA, and the grey literature. Two independent researchers selected (DistillerSR) and critically appraised (Mixed Methods Appraisal Tool) all included studies. Data extraction and synthesis consisted of a deductive-inductive qualitative thematic analysis (NVivo), followed by a disambiguation/ harmonization of themes. A framework synthesis was used, which consisted of coding evidence against an a priori framework to produce a revised framework.

Results

Out of 4322 unique records identified in our search, 66 studies fulfilled the eligibility criteria (48 quantitative studies, 11 qualitative, 3 mixed methods and 4 using diverse designs). Study participants were either OCHI consumers (patients, caregivers, general public) or clinicians (family physicians, dentists). Nine of the included studies examined a specific OCHI intervention (e.g. information prescriptions). The majority of the studies examined general online health information seeking behaviour and use. Included studies demonstrated that using OCHI is associated with both positive and negative outcomes. Main negative outcomes were: 'deterioration of the patient-physician relationship', 'increased worrying', and 'overuse or misuse of health services'. We identified new system-related outcomes. For example, using OCHI may increase physician's responsibilities or lead to excessive ER visits and longer wait times. Moreover, we established factors influencing OCHI acquisition and outcomes (e.g. health literacy). All identified themes underwent a harmonization process, resulting in a terminology of 45 key terms and concepts.

Conclusion

Based on these results, we propose a revised conceptual framework of the outcomes associated with the use of OCHI in primary health care, as well as potential factors (facilitators and barriers) influencing the acquisition, cognition, and use of information.

Poster Number: 236

Time: Monday, May 16, 2016, 2:30 PM – 3:25 PM

The "5S" Integrated Levels of Evidence Pyramid: The Evolution of a Model

Whitney A. Townsend, Informationist, Taubman Health Sciences Library, UM Library, Ann Arbor, MI; Emily C. Ginier, Health Sciences Informationist, University of Michigan, Taubman Health Sciences Library, Ann Arbor, MI; Nandita S. Mani, AHIP, Assistant Director, Academic & Clinical Engagement, Taubman Health Sciences Library, Taubman Health Sciences Library, Ann Arbor, MI; Mark MacEachern, Informationist, Taubman Health Sciences Library, Taubman Health Sciences Library, Ann Arbor, MI; Anne Perorazio, Media Developer, Taubman Health Sciences Library, Ann Arbor, MI

Objectives: Develop a model based on the "5S" levels of organisation of evidence pyramid that integrates study types and additional information sources in order to facilitate appropriate resource targeting and recognition of evidence-based versus non-evidence-based resources.

Methods: The "5S" levels of organisation of evidence pyramid developed by RB Haynes is a widely used model that was designed to assist healthcare practitioners in targeting high level of evidence information to aid in clinical decision making. While the 5S pyramid is undoubtedly useful, incorporating a broad range of information types within the context of the pyramid was necessary; therefore, new components were integrated. We present the "5S" integrated levels of evidence pyramid model that approaches clinical decision making in a holistic manner by incorporating non-evidence-based resources, study types within the existing pyramid levels, a delineation between primary and secondary sources of information, and helps to map available resources to corresponding level.

Results: To be reported

Conclusions:
To be reported

Poster Number: 242

Time: Monday, May 16, 2016, 2:30 PM – 3:25 PM

Collaborating Across Campus to Support Faculty Research and Publishing: Library-as-Partner Model

Young-Joo Lee, Senior Clinical Librarian, Louis Stokes Health Sciences Library, Howard University , Washington, DC

Objectives: This study is designed to explore how the library can build strong, long-term partnerships with other prominent departments on campus whose missions also support research and publishing. The goal is to create a program that will expand the educational role of the librarian and to measure the impact of these collaborations using both qualitative and quantitative methods, including faculty evaluations.

Methods: To explore a library-as-partner model, the author will describe collaborations she developed with three prominent on-campus resources providing research support services: the Office of the Associate Provost of Research and the Office of Sponsored Programs, both of which support research and funding, and a center supporting clinical research and data management practice. Examples of cooperative efforts with each included the creation of monthly seminars and other presentations sponsored by the partnering offices and co-coordination of popular and high profile events on campus. To further strengthen the partnerships, the author also invited the staff of each office to take part in her own lectures. Evaluation of the model will be done through interviews and commentary from each partner as well as through survey results from faculty who attended or took part in the aforementioned services and events.

Poster Number: 2

Time: Tuesday, May 17, 2016, 2:00 PM – 2:55 PM

Collecting Pre-Class Information from First-Year Pharmacy Students in Order to Increase Student Engagement with Library Instruction

Adelia B. Grabowsky, Health Sciences Librarian, Ralph Brown Draughon Library, Auburn University, Auburn University, AL

Objectives: To increase student engagement, both in a one-shot library instruction session and with the Pharmacy subject guide which provides supplemental instruction information.

Methods: Setting/Participants: 151 first year pharmacy students enrolled in an introductory drug literature class. Methodology: One week prior to library instruction, students were sent a link to the Pharmacy subject guide, asked to spend 10 to 15 minutes examining information on seven of the tabs, and then to answer three open-ended questions. The questions asked students to list at least one thing learned from the subject guide, one thing that confused them or that they would like more information about, and last what they believed to be most important for the librarian to cover in class. Information obtained was used to structure the library instruction session. Increased engagement was evaluated by comparing both student interactions in class (questions asked and answered by students) and use of the subject guide for six months after class to the previous year's cohort.

Results: Seventy-six percent of students completed the pre-class questionnaire. Searching PubMed and Knowing which databases/resources to use ranked number 1 and 2 for the "Confused" question and number 3 and 2 for the "What should be covered" question. Number 2 for the "Confused" question was Finding full text; How to search more effectively ranked number 1 in "What should be covered". Class interactions were greatly increased compared to the previous year's cohort and use of the Pharmacy subject guide for 6 months after the 2015 class was more than double use of the guide for 6 months after the 2014 class.

Conclusions: Current learning theories suggest that students are more engaged when they feel they have input into instruction. Asking students what confused them and what they would like to see covered seemed to increase engagement among first year Pharmacy students, both in class and after class with supplemental instruction material in the form of a subject guide.

Poster Number: 5

Time: Tuesday, May 17, 2016, 2:00 PM – 2:55 PM

Opportunities and Efficiencies in Building a New Service Desk Model

Alexa Mayo, AHIP, Associate Director for Services, Health Sciences and Human Services Library, University of Maryland–Baltimore; Everly Brown, Head, Information Services, Health Sciences and Human Services Library, Health Sciences and Human Services Library, Baltimore, MD; Ryan Harris, AHIP, Reference Services Manager, Health Sciences and Human Services Library, Health Sciences and Human Services Librarian, Baltimore, MD

Objectives: After years of declining reference, shelving and circulation activities, we understood the library's model of separate Circulation and Reference Desks with distinct staffs (15 FTE total) and expertise no longer made sense. In summer 2015, we merged the two desks and reexamined our service model from the ground up to ensure that the library remains a vibrant and active space.

Methods: The Services management team planned the new Information Services Desk. We conducted a literature review and examined service models at peer institutions. We met with staff individually and in groups to hear about their ideas for improving our user's experience, and to gain trust and buy-in as we moved toward the transition. Teams collaborated to envision and design the improved service-oriented desk as well as how to use the space left vacant after the merge. Staff cross-trained so that they shared a set of core competencies. Position descriptions were rewritten and reclassified to reflect the new work environment; there were also layoffs. To improve service, staff moved away from task-oriented work, thereby reducing interruptions and guiding people seamlessly through transactions. After a year of department-wide thoughtful planning, the new Information Services Desk rolled out in summer of 2015.
Results: The new Information Services desk is located in the former Circulation Desk area. The former Reference Desk was repurposed as an Innovation Space with 3D printing and scanning. We do not staff this space, but are trained to use the equipment and assist patrons as needed.
Conclusions: The creation of the new Information Services Desk was successful. Faculty, staff, and students continue to have a high level of reference and circulation service. Part of the success of the transition is attributed to staff involvement throughout the process.

Poster Number: 8

Time: Tuesday, May 17, 2016, 2:00 PM – 2:55 PM

Taking on New Roles: Teaching Evidence-Based Medicine as Part of the Curricular Team

Amanda Chiplock, Senior Medical Librarian/Department Head, Florida Atlantic University, Wimberly Library / Medical and Health Sciences Collections & User Services Department, Boca Raton, FL

Objectives: At a new medical school, librarians took on the role of educator and became part of the curricular team. The goal was to integrate medical librarians into the curriculum with the anticipated outcome of effectively teaching evidence-based medicine (EBM) to first-year medical students and filling a gap in the curriculum. To increase student engagement, the medical librarians incorporated interactive techniques.

Methods: Three steps in EBM directly relate to information literacy: ask, acquire, and appraise. Thus, medical librarians were invited to develop and implement the EBM program. They joined the college's curriculum meetings and chaired the learning resources committee.

The librarians researched evidence-based practice and collaborated with physicians to develop the EBM program, which was designed as part of the Foundations of Medicine course. Five EBM lectures were scheduled per academic year between 2011 and 2016 including large group activities combined with audience response tools (Mentimeter) and Jeopardy-style games (FlipQuiz).

Each academic year, students received an exam at the end of the first semester and individually completed a search assignment at the end of the second semester. The assignment included presenting recommendations for treatment to preceptors, who then evaluated the presentations. The exam and overall assignment were graded by the physician/librarian team.

Results: Based on observation, student responses from surveys completed after every EBM session, and feedback from course directors and faculty facilitators at Florida Atlantic University, Charles E. Schmidt College of Medicine, the EBM program designed for first-year and second-year lectures is successful in teaching lifelong learning skills of research, appraisal, and application of clinical research literature. Students are more engaged in EBM with the introduction of new interactive activities such as gaming, online tutorials, and small group activities. However, what seems to be lacking is the students' understanding of how to implement EBM in clinical practice.

An unexpected result of this project was the development of the EBM thread through all four years of the undergraduate medical curriculum due to the success of the EBM lectures, and an opportunity for the medical librarians to become leaders in curriculum planning as we are now a resource for other faculty who wish to create interactive lectures and activities.

Conclusion

Medical librarians will continue to work closely with the curriculum committees, course directors, and EBM thread director to create and provide innovative, interactive EBM lectures. The lectures are becoming more effective with each new interactive activity. We will work to improve the area of application.

Poster Number: 11

Time: Tuesday, May 17, 2016, 2:00 PM – 2:55 PM

More than Expert Searchers? A Case Study for Librarians Exploring Roles Beyond Databases

Ana Patricia Ayala, Instruction & Faculty Liaison Librarian; Erica Lenton, Faculty Liaison & Instruction Librarian; Gerstein Science Information Centre, Toronto, ON, Canada

Objectives: Background: At the University of Toronto (UofT), librarian involvement in a variety of reviews and grant proposals happens largely and primarily on an ad-hoc basis. The role of health sciences and medical librarians as expert searchers is well established and recognized, yet librarians can, and have, also been called upon to navigate other components of the research process (Dudden 2011). Involvement includes but is not limited to: project manager, research coordinator, and process/protocol expert. Librarian involvement is not only recommended, but has become a requirement for grants from key funding agencies, such as the Canadian Institutes of Health Research (CIHR), for a proposal to be considered complete and admissible (CIHR 2010). This is wonderful news, but are we prepared? Objectives: The objective of this study is to develop a sustainable set of service standards in order to clarify the roles and tasks to be performed by librarians in research initiatives such as systematic reviews, but perhaps more importantly, scoping and realist reviews, as support requests for these newer, less familiar, methodologies have become increasingly common.

Methods: Methods: We will gather and organize pre-established protocols for each type of research study and disseminate them widely. An environmental scan will be performed to learn more about how librarians at UofT are currently supporting research teams' reviews. This knowledge can then be shared among our colleagues and incorporated into the content of our instruction portfolio.

Results: Results: The results of this initiative and anecdotal gathering are ongoing and will be presented at the MLA/CHLA meeting.

Conclusions: Conclusions: Librarians offer significant contribution and insight beyond their searching skills; they can successfully act as project managers, process experts, and disseminators of protocols (communicators). Their role continues to evolve. Above all our goal remains to have an open and honest conversation on what librarians and information specialists' experiences have been so far in these type of initiatives, what our roles are and what they should be.

Poster Number: 17

Time: Tuesday, May 17, 2016, 2:00 PM – 2:55 PM

Out of the Ether: Leveraging iTunes U for Grand Rounds Lectures

Annie R. Oldenburg, MLIS Student, University of Wisconsin Milwaukee School of Information Studies, Rochester, MN

Objectives: Graduate medical education depends upon reliable, easily accessible, specialty-specific information sources. A department's collection of grand rounds lectures is one such valuable source. However, lectures given in the otorhinolaryngology department at the Mayo Clinic were essentially inaccessible following the live presentations. The objective of this program was to identify and implement a mobile, secure, and searchable grand rounds library for residents and staff.

Methods: Program development was divided into two phases: platform selection and content migration. During phase one, the author interviewed members of the residency cohort to determine inclusion criteria for platform evaluation. The iTunes U platform was ultimately chosen for meeting the criteria of universality, security, mobility, search functions, and ease of use for both the audience and the administrator. The author collaborated with hospital staff from the compliance and marketing departments to identify and resolve issues such as copyrights and branding. During phase two, the department's collection of grand rounds lectures was digitally reformatted, cataloged, and uploaded to iTunes U. Audio and video editing presented unique challenges. The author presented a pilot version of the library to the residency cohort to familiarize the group with iTunes U and to generate feedback. Alterations were then made, including the addition of multimedia content.

Results: Residents showed a mixed utilization of the iTunes U course. Several users accessed the course frequently (multiple times per week) while others only accessed the course a single time. Though several staff physicians registered for the course, none accessed the course more than once. One staff physician recommended opening access to outside audiences. Frequent users enjoy the ease of use iTunes U provides and requested catalog expansion.

Conclusions: This pilot program proved the iTunes U platform 1) is compatible with lecture-based content, 2) meets the mobility and security requirements for non-PHI health content distribution, and 3) is easy to use for administrators and end users. To be implemented effectively, an iTunes U course should have a large catalog and a broad audience.This pilot program proved content migration to iTunes U to be a feasible task, even for a staff of one.

Poster Number: 20

Time: Tuesday, May 17, 2016, 2:00 PM – 2:55 PM

Building a Shared Modern Hospital Web Portal for a Health System

Basia Delawska-Elliott, Medical Librarian, System Library Services, Providence Health & Services, Portland, OR; Isaac Huffman, Director of System Library Services, Library Services, Providence Health & Services, Renton, WA; Heather J. Martin, AHIP, Director of Regional Library Services, Health Sciences Libraries, System Library Services, Portland, OR

Objectives: Digital resources have provided the opportunity to expand services. In 2014 Providence Health & Services libraries embarked on creating a shared web portal for the large not-for-profit health system that spans 5 states. Faced with legacy web portals, limited resources, and mandated technical requirements, the system-wide library aimed to create a new universal web portal to serve our mobile savvy patrons.

Methods: An assessment of the current digital landscape uncovered many information gaps. These included hospitals with no library website, resources controlled by a librarian gatekeeper, out of date holdings, and challenging interfaces. Using user feedback and staff expertise, the library web portal team identified a number of specifications. Technical requirements included a simple visible URL, a single sign-on for all users, easy remote access, a mobile access interface, and user-friendly navigation. Interface goals included local library staff links, a discovery search page and meeting IS mandated requirements. Content goals included the ability to create curated content and a single universal digital collection. The ultimate goal was to create a library web platform that provided access to resources with limited training. Using a digital mock up the library group tested six successive interface designs which helped inform further design specifications.

Results: In the first 6 months since launch the main page views increased from 2,581 to 22,078 views per month. At the current 5% growth rate we will reach our target of 30,000 monthly user sessions in 2016. While difficulties navigating internal IT structures originally delayed offsite access, standard URL, and mobile design, all three access points launched in February 2016. Growth and user satisfaction were aided by meeting library design specification, using a catchy superhero theme, and creating an easy interface that highlighted search.

Conclusions: Creating a shared web portal has increased user satisfaction in library services and has grown library page traffic. The site has been widely praised as an improvement in look and functionality. The library looks forward to increasing future offerings over the next few months and meeting the user

goals. Despite the challenges of bringing together diverse online library environments, it is possible to phase out legacy web portals and create a universal access point to all resources in a large health system.

Poster Number: 23

Time: Tuesday, May 17, 2016, 2:00 PM – 2:55 PM

Marketing Hospital Libraries: The Ideal and the Real

Beverly McLeod, Manager of Library Services, Kaiser Permanente, Santa Clara, CA; Marina T. Aiello, Mgr of Library Services, Tech and Instr Design, Kaiser Permanente, Stockton, CA; Wm. Brian Elliott, Technical Services Coordinator, Kaiser Permanente, Clackamas, OR; Jennifer McBride, NW Regional Libraries Coordinator, Kaiser Permanente, Clackamas, OR; Belen Thornfield, Assistant Medical Librarian, Kaiser Permanente, Anaheim, CA

Objectives: To explore real-world approaches to marketing undertaken by librarians working in a hospital medical library setting. The purpose of this project is to discover and share "best practices" and "lessons learned" from successful and unsuccessful marketing initiatives to help hospital librarians develop practical and achievable "good enough" marketing plans.

Methods: Many books, articles, and reports discuss principles of library marketing and describe step-by-step processes for creating marketing plans, but there are fewer accounts of actual marketing programs, particularly in a hospital library setting. To attempt to fill that gap, a team of medical librarians at Kaiser Permanente health sciences libraries searched the medical librarianship literature to reveal the scope of published descriptions of hospital marketing efforts, surveyed hospital librarians to gather unpublished accounts of such initiatives, and detailed the evolution of a marketing program at our multi-hospital organization during the past four years.

Results: A major objective of this project was to ascertain the extent to which medical librarians have followed recommended procedures for creating and implementing marketing plans. However, it turns out that published accounts of hospital library marketing are rare, and reports of actual comprehensive marketing plans are practically nonexistent. An analysis of articles published during the past decade revealed that most described only one aspect of marketing, such as promotion of specific library services or resources or time-limited communication and outreach efforts. Survey responses echoed the published accounts and provided insight into the challenges impeding "ideal" library marketing in a hospital setting. Similar challenges exist in our own organization, but we have made strides toward the development of an integrated and sustained approach to marketing in our health sciences libraries.

Conclusions: The "state of the art" regarding hospital library marketing is that a huge gap exists between the ideal and the real. Despite the availability of books and articles offering encouragement, guidance, examples, and strenuous exhortation to engage in marketing, few hospital librarians have been able to

follow the advice to incorporate systematic marketing into the operation of their library. We share our own organization's barriers and successes in our library marketing journey in the hope that, along with the information gathered in this study, our story will contribute to a knowledge resource for use in future hospital marketing programs. Like previous advice-dispensers, we believe that marketing is vital to the future health of hospital libraries and to the professional lives of our fellow health sciences librarians.

Poster Number: 26

Time: Tuesday, May 17, 2016, 2:00 PM – 2:55 PM

Reflecting on the First Steps: A Structured Analysis of Medical Subject Headings (MeSH)

Blake Hawkins, Graduate Student, University of British Columbia, Master's of Library and Information Studies, Vancouver, BC, Canada; Laura Bartlett, Technical Information Specialist, National Library of Medicine, Specialized Information Services, Outreach and Special Populations, Bethesda, MD; Sigrid Brudie, Medical Reference Librarian, Consortium Library, Alaska Medical Library, Anchorage, AK; Kathleen Murray, Alaska Medical Library Manager, University of Alaska Anchorage, Alaska Medical Library, Anchorage, AK

Objectives: The University of Alaska Anchorage, is trying to facilitate needed changes regarding MeSH terms pertaining to Indigenous / Arctic Health. We will describe the process associated with doing a structured analysis of MeSH. Our goal is to build a case that MeSH needs to be expanded to better meet the needs of Arctic peoples and researchers with an Arctic focus.

Methods: The project involves multiple stages of investigation before it is possible to make any recommendations concerning Indigenous / Arctic MeSH terms. We began by reviewing NLM policies concerning MeSH terms, and what is required to successfully make term revisions. Using existing searching hedges, we also investigated current MeSH terms to learn which can be included for future searches. Concurrently, we wanted to find which terms are most commonly used to index articles pertaining to Indigenous health. New MeSH terms with a focus on Indigenous Arctic peoples and perhaps regions within the Arctic will be recommended.

Results: We have now reached the point in this project that we are writing a white paper for the NLM that demonstrates why there is a need for a new search term. It is no longer just rationalized on personal biases to create a new MeSH heading. Instead, we have gone through hundreds of articles regarding Alaska Native health, and there is a gap in how they are categorized.

Conclusions: We are now waiting for a feedback on the paper and hope that it will be accepted by the NLM. We were required to meet certain standard set out by the NLM to advocate for a new MeSH term. Through the steps we completed, we are confident that it will result in needed change. Furthermore, we hope our model can be followed by other groups who believe there should be new MeSH terms to better represent their population(s).

Poster Number: 28

Time: Tuesday, May 17, 2016, 2:00 PM – 2:55 PM

Searching the Literature: One Size Does Not Fit All

Brooke Ballantyne Scott, Librarian, Royal Columbian Hospital, Fraser Health, Royal Columbian Hospital Library, New Westminster, BC, Canada; Jacqueline M. MacDonald, Privacy Officer, Quality, System Performance and Legal Services, Nova Scotia Health, Bridgewater, NS, Canada; Lori Leger, Regional Manager of Library Services, Horizon Health Network, Horizon Library Services (Moncton Area), Moncton, NB, Canada; Susan Baer, Director of Libraries & Archives, Regina Qu'Appelle Health Region, Regina General Hospital, Health Sciences Library, Regina, SA, Canada; Ashley Farrell, Medical Librarian, Cancer Screening, Cancer Care Ontario, Toronto, ON, Canada; Marcus Vaska, Librarian, Holy Cross Centre, Knowledge Resource Service, Calgary, AB, Canada; Pat Lee, Librarian Educator, Library Services, Nova Scotia Health , Halifax, NS, Canada

Objectives: Library services managers, professional searchers and search instructors lack a standard to support mediated search service instruction and accountable search service delivery. A standard is needed to establish a consistent approach to executing different types of searches, provide a framework against which search service performance may be measured and support cost benefit analysis of expert, mediated search services demonstrated.

Methods: Two approaches were used to inform this pan-Canadian work. The first was an environmental scan using several listservs to identify current search-related researchers and practice leaders. The second was an iterative literature review of research and other literature on mediated searching. Content analysis of all documents gathered identified a variety of search types and methods. The search types, methods and related terms were defined in a search glossary, organized in a matrix and then validated through a Delphi study with search researchers, authors publishing on search topics and professional searchers.

Results: Results included identification of essential and optional steps in recommended approaches for different types of searches. The first draft of this work will be shared as a consensus-building step in standard development.

Conclusions: The research literature on search methods is sparse and fragmented, lacking in currency and a shared vocabulary. A standard would provide clarity in terminology, approach and methods align with the accreditation of other health care professionals and maintain the relevance and value of health services libraries within the health care system. Standards enable us to provide a consistent service experience to our users, especially

within multi-site services with both physical library and virtual environment settings.

Poster Number: 29

Time: Tuesday, May 17, 2016, 2:00 PM – 2:55 PM

Lesbian, Gay, Bisexual, Transgender, Questioning (LGBTQ) People, Health Information Seeking, and the Role of Health Librarians: A Scoping Review

Blake Hawkins, Graduate Student, University of British Columbia, Master's of Library and Information Studies, Vancouver, BC, Canada; Luanne Freund, Associate Professor, University of British Columbia, School of Library, Archival, and Information Studies, Vancouver, BC, Canada; Martin Morris, Liaison Librarian: Life Sciences, Schulich Library of Science and Engineering, McGill University, Montréal, PQ, Canada

Objectives: There are gaps in the health information literature concerning LGBTQ people and the role of health librarians in information seeking. The available scholarship is emblematic of earlier perceptions regarding interactions between LGBTQ people and their health information needs. This paper will demonstrate why it is necessary for health librarians to better understand the unique needs of LGBTQ youth, and provide recommendations to cultivate a better awareness about their needs.

Methods: The research questions guiding our scoping review are "what are the health information needs of LGBTQ people" and "what is the role of health librarians with respect to LGBTQ information needs?" We completed a review using Embase 1974 to present, Embase 1974 to present, CINAHL and PubMed with Full Text, and Ovid MEDLINE(R) In-Process & Other Non-Indexed Citations and Ovid MEDLINE(R) 1946 to Present. We limited the results to publications after 1990 – August 2015. We found 112 articles across these databases, and we reduced to 66 after culling our research due to the relevance of the research question. After the scoping exercise, we mapped the results into the themes that have framed our discussion.

Results:
Currently, there is a limited amount of literature regarding the role of health librarians with LGBTQ people health information seeking. The current literature is associated with the challenges many younger people face when first trying to engage with the literature as fearful and/or closeted youth. Additionally, there are narratives regarding the problematic information seeking process many LGBTQ people use (i.e., online blogs or social networks) that could potentially result in unhealthy information sharing.

Conclusions: We advocate for further engagement by health librarians with

LGBTQ patrons and their information needs. This is a population that has historically been stigmatized and unrecognized as having different health needs. The health librarian can have a greater role in supporting proper information seeking practices by LGBTQ people.

Poster Number: 32

Time: Tuesday, May 17, 2016, 2:00 PM – 2:55 PM

Liaisons versus the Machine: Leveraging Relationships to Increase Participation in an Open Access Policy

Caitlin Pike, AHIP, Nursing and Medical Humanities Liaison Librarian, Indiana University-Purdue University Indianapolis (IUPUI), IUPUI University Library, Indianapolis, IN; Jere Odell, Scholarly Communications Librarian, University Library, University Library, Indianapolis, IN

Objectives: A large, mostly health science campus in the Midwest adopted an open access (OA) policy with implementation guided by the libraries. Most faculty authors will receive generic email notifications prompting participation. Would liaison advocacy increase participation in the policy? Do relationships matter?

Methods: Liaison guidance for OA policy participation was piloted with promising results in the School of Nursing. Using the pilot as a model, a cohort of departments will receive liaison guidance for OA policy participation. Another cohort of comparable departments will receive generic email notifications prompting participation. Differences in participation rates will be measured and reported.

Poster Number: 37

Time: Tuesday, May 17, 2016, 2:00 PM – 2:55 PM

The Role of Library Services as Tesserae in the Healing Environment Mosaic

Catherine M. Boss, AHIP, Coordinator, Library Services; Darlene Robertelli, Librarian; Chunwei (Charlie) Ma, Systems Librarian; Booker Health Sciences Library, Booker Health Sciences Library, Neptune, NJ

Objectives: Creating, funding and maintaining library services that interplay together, system-wide, is crucial to improving outcomes and to creating a healing environment within an expanding hospital system. The Coordinator of Library Services, working with Administration and with the diverse clinical, educational and research needs of the hospitals, the community, and the in-home healing environment of her 6-hospital health system, created a mosaic of services that can easily grow and expand as the health system grows and expands.

Methods: In crafting the mosaic of library services, the library culture within the health system was changed to one that promotes library services for patients, staff and the community. Suggestions were solicited to reduce and eliminate services that were of little value. Focus was placed on purpose and in doing what was considered important, communicating that purpose and plan throughout the system. Resources are licensed and financed for the system through the library.

Results: Four of the library's computers were reconfigured to create a pod of social media-enabled computers. Archival pictures delivered significant community-oriented tesserae for a local author's book as did our reference librarian appearing in a system marketing video. Other impressive tesserae included a 107% growth in the number of documents provided from the library's collection, a 70% growth in the library's electronic book collection and a 20% increase in the number of nursing literature searches compiled. Over 400 business services were provided for team members from our campus as well as over 200 literature searches for team members from other campuses.

Conclusions: The positive impact of the Library's tesserae of services on Meridian Health's healing environment is evidenced by the unsolicited kudos:
¨ "I am so proud to work for an organization that promotes library services for patients, staff and the community. The access to journals, the up to date texts and the accessibility for patient teaching will help us provide our patients with excellent evidence- based care"
¨ "Thank you so much for the information you provided for me so quickly. I'm

home now doing well. ¨ "Thank you for sending me the info...Very informative! My children also send their thanks."

Poster Number: 43

Time: Tuesday, May 17, 2016, 2:00 PM – 2:55 PM

Search Smart for Your Health: A Statewide Health Literacy Campaign

Christine A. Willis, Director of Knowledge Management & Learning Resources, Noble Learning Resource Center, Shepherd Center, Atlanta, GA; Shannon Glover, Supervisor, Library Services, Wellstar Health System, Kennestone Hospital, Health Sciences Library, Marietta, GA; Tara Douglas-Williams, Division Head for Information Services, Library Manager, MSM Library, M. Delmar Edwards, M.D., Library, Atlanta, GA

Objective – To raise health literacy awareness across the state of Georgia, the Georgia Health Sciences Library Association (GHSLA) and the Georgia Alliance for Health Literacy (GAHL) partnered to educate residents about reliable health information online and to create models that will encourage educators in Georgia to teach consumers, patients, and youth about online health information seeking.

Methods –GHSLA members were invited to give a presentation on health information resources at a GAHL meeting in 2015. The presentation was well received and the librarians reached a group they normally would not have interactions with. This presentation evolved into a partnership whereby a website and "smart teaching tools" were created and showcased during National Health Literacy month. The organizations received a gubernatorial declaration that October 2015 is Health Literacy Month in Georgia. Information was posted on both organizations' websites with "smart searching" tips and links to reliable health information websites. Demonstration workshops were held in a variety of locations with different age groups to address online health information seeking needs. Press releases were also distributed to statewide news organizations along with flyers to state health educators.

Results - Outreach efforts by GHSLA and GAHL resulted in two events that reached 16 adults at The Atrium at College town, a senior living facility in downtown Atlanta, and 24 teens at the Center for Pan Asian Community Services during an after school program in north Atlanta. Presentations to both groups were derived from the "smart searching" tips developed by GAHL and GHSLA.

Conclusions - Networking with other health literacy groups has provided opportunities that resulted in mutual benefits. Through this collaboration the professionals involved in GAHL were exposed to the resources that the GHSLA members access frequently and they learned that the GHSLA members are also advocates and educators of health literacy efforts in the community. This partnership has also provided a means for health science librarians across the state to partner with local organizations and hopefully have a greater impact in addressing the vital health information needs of the citizens of Georgia. We

have plans for future projects and our goals is educated as many individuals as possible, especially those in health disparate areas across the state.

Poster Number: 49

Time: Tuesday, May 17, 2016, 2:00 PM – 2:55 PM

Collaborative Curriculum Design with the Vice President's Clinical and Translational Research Scholars Program

Darell Schmick, Research Librarian, University of Utah, Spencer S. Eccles Health Sciences Library, Salt Lake City, UT; Jan Abramson, Assistant Director for Clinical Research Development, University of Utah, Office of the AVP for Faculty & Academic Affairs, Salt Lake City, UT

Objectives: To describe librarian integration into the curriculum of a two-year, competitive research scholars program for junior faculty on a health sciences campus.

Methods: The Vice President's Clinical & Translational (VPCAT) Research Scholars Program offers intensive mentorship and support to junior faculty committed to careers in clinical or translational research. The two-year program incorporates mentoring; structured curriculum focused on leadership competencies, essential knowledge and practical skills to be an effective researcher; and access to resources to facilitate appropriate study design, collection of pilot data and preparation and submission of competitive grant applications. VPCAT Program staff reached out to the library to collaborate on developing and implementing librarian-led research skills workshops during monthly peer networking meetings. The classes are designed as building blocks for effective research practice. In keeping with the philosophy of mentor support, the librarian serves as a staff mentor and resource for the VCPAT Research Scholars continuously throughout the two year program and beyond.

Results: Partnering with the VPCAT Research Scholars Program provides a robust platform for the library and librarian to positively impact practices of emerging researchers. The skills and knowledge provided by the librarian were so positively received that a monthly Building Blocks for Research skill development series has been implemented. Building Blocks are offered during peer networking meetings; this facilitates researchers recognizing the librarian as a uniquely qualified resource.

Conclusions: Librarian involvement within the VPCAT Program proves to be a successful ongoing partnership. Curriculum development, teaching Building Blocks, being 'known and accessible' has furthered the mission of the library to support research and innovation. In this novel partnership, the library is able to provide opportunities for hands-on research skills sessions for the scholars as they shape their research ambitions within the VPCATS program.

Poster Number: 51

Time: Tuesday, May 17, 2016, 2:00 PM – 2:55 PM

Strategic Planning for a Single-Person Medical Library

David E. Coleman, Medical Librarian/Informationist, Medical Library Services / Hawaii Pacific Health, Honolulu, HI

Objectives: This paper describes the process and development of a library master plan that brings value to a single person library within a non-profit multi-hospital organization. The paper discusses differences between strategic and long-range planning as they relate to the management of single person libraries. The uses of strategic planning documents and library management are explored. The relationship between organizational values and library relevancy are discussed.

Methods: We began with a discussion and understanding of the organizational mission and vision statement. From there we looked at the primary organizational values and goals. Based on this discussion we developed a library mission and vision statement that paralleled and supported that of the organization. This is essential so that the library is not seen as a separate entity but rather an integral part of the organization and an essential element in achieving its mission. We developed a desired end state defined by the library mission and vision statement and then determined the steps that would lead to that end state. This is starting from the future and working backward rather than from status quo and going forward.

Results: What ultimately developed was a set of documents that serve as a crosswalk between the strategic steps being taken by the organization to achieve its mission and the parallel steps taken by the library to assist the organization in meeting that end and supporting the organizational values.

Conclusions: What we have now is a document set that includes a mission statement, a vision statement, and a strategic plan broken down into goals. Each goal is then linked to the relative organizational value that it supports. Within those goals are objectives that are the focus of each fiscal year for three years.

Poster Number: 53

Time: Tuesday, May 17, 2016, 2:00 PM – 2:55 PM

New Service Rollout: Marketing, Outreach, and Assessment

Debra Werner, Librarian for Science Instruction and Outreach, John Crerar Science Library, John Crerar Library, Chicago, IL; Ricardo Andrade Jr., Biomedical Librarian, John Crerar Science Library, The John Crerar Library, Chicago, IL; Michelle B. Bass, Science Research Services Librarian, University of Chicago, The John Crerar Library, Chicago, IL

Objectives: The objective of this presentation is to discuss the process of launching new research support services at an urban academic health sciences library, including marketing, outreach efforts, and assessment. Over the 2015-16 academic year, we are introducing new research support, for example, a systematic review service, to engage with patrons across departments and skill-levels to create new partnerships.

Methods: We will present case studies on each of the new research support service initiatives: systematic review service, NIH Public Access Policy support, and data management education. The case studies will focus on our marketing and outreach efforts and subsequent assessment. Marketing techniques will include tweets and conversations on our reinvigorated library Twitter account, the creation and updates of library guides, and blog posts. Outreach efforts will include communicating to individual department chairs, targeted biomedical centers on campus, and attending relevant graduate student and faculty meetings. Assessment will be conducted using analytic information from social media and email marketing, authorship acknowledgements, and follow-up requests.

Poster Number: 57

Time: Tuesday, May 17, 2016, 2:00 PM – 2:55 PM

Health Sciences Librarians Use In-Depth Interviews to Evaluate a REDCap Training Program

Don P. Jason, III., Clinical Informationist, Health Sciences Library , Donald C. Harrison Health Sciences Library / University of Cincinnati Libraries, Cincinnati, OH

Objectives: This poster examines how librarians conducted interviews with researchers to evaluate and improve a Research Electronic Data Capture (REDCap) training program. The findings from this research project provided librarians with a clearer understanding of how researchers use REDCap. Librarians are using this knowledge to improve the REDCap training program. The ultimate goal of this project is to align REDCap training with the needs of researchers.

Methods: Librarians from the Donald C. Harrison Health Sciences Library's (HSL) conducted interviews with University of Cincinnati (UC) researchers who attended the HSL's REDCap training workshops. All workshop participants who completed training in the past six months were invited to participate in the in-person interviews. The Interviews consisted of eight questions. These questions asked researchers to explain how they use REDCap to conduct their research. The interview continued with questions that asked the researchers to reflect on what they liked and didn't like about REDCap. Finally, the questions encouraged researchers to talk about the training and tech support they received for REDCap, and how they would improve it.

REDCap is a robust online tool that allows for the secure collection, storage and dissemination of research data. The library's training program started after UC's Center for Clinical and Translational Science and Training uncovered a need for broader REDCap training across the university. In addition, UC's Institutional Review Board strongly suggested that researchers use REDCap as opposed to less secure data storage methods. These suggestions prompted a need for additional training on REDCap. The HSL became a collaborator and partner in the development of REDCap training workshops.

Results: Researchers want to learn REDCap through a training program that appeals to many different learning styles. They value online and in-person training offered by the library. Nevertheless, they value learning from their peers. They want to connect with fellow researchers who are using REDCap. These meetings can occur online or in-person, but they only want to meet with other researchers who are working on similar research projects. Researchers value flexibility in the REDCap support model. They want in-person tech

support, but they also want ways to share their screen, and get assistance remotely from IT staff.

Conclusions: Librarians must use a multipronged approach for providing training and tech support for REDCap. One training and support model does not serve the needs of every researcher.

Poster Number: 60

Time: Tuesday, May 17, 2016, 2:00 PM – 2:55 PM

Innovations, Challenges, and Opportunities within Regional Health Libraries in British Columbia, Canada

Shannon Long, Librarian, Vancouver Coastal Health Library Services, Richmond Hospital Library, Richmond, BC, Canada; Elisheba Muturi-Kihara, Policy Analyst, BC Ministry of Health, Policy Outcomes and Evaluation, Burnaby, BC, Canada; Chantalle Jack, Librarian, Vancouver Coastal Health Library Services, Lions Gate Hospital Library, North Vancouver, BC, Canada

Objectives: Faced with continual change, regional health libraries must innovate in order to survive. To understand how these libraries are evolving to deliver innovative services to geographically spread users, this study: 1) describes and compares the libraries, 2) analyzes how they are evolving and innovating in order to deliver value, and 3) identifies gaps and opportunities in the current landscape.

Methods: Setting/Participants: Librarians working in regional and Ministry of Health libraries in British Columbia (BC). Data Collection and Analysis: Librarians will complete an online survey and be interviewed regarding eight themes drawn from the literature: overview of the library environment, research services, teaching role, centralized vs distributed service delivery model, prioritization, evaluation, innovative services, and the provincial landscape.

Results: Libraries range considerably in size and staffing levels, although all are well positioned within the organization's reporting structure. A mix of centralized and distributed service delivery models are in practice. Reference and literature searching was identified by all as the most important service provided. Offering instructional services to those who work in remote areas is a common challenge. Libraries are carrying out regular needs assessment and evaluation activities but seek to find outcomes and indicators that prove their worth within the organizations they serve. Innovative, non-traditional activities are being embraced as a means to improve delivery of information and resource. Barriers to further innovation were universally expressed.

Conclusions: Regional health libraries in BC differ significantly in size, staffing and service model but share the challenge of delivering innovative services to geographically spread users. In a landscape of library closures and service consolidation, these findings promote information sharing on innovative best practices and highlight collaborative opportunities to address existing gaps.

Poster Number: 63

Time: Tuesday, May 17, 2016, 2:00 PM – 2:55 PM

NExT: Comparing the Effectiveness of Public Health Nursing Evidence-Based Practice In-Person Education to an Online Asynchronous Tutorial

Emily M. Johnson, Regional Health Sciences Librarian & Assistant Professor, Library of the Health Sciences- Peoria, Library of the Health Sciences- Peoria, Peoria, IL; Carmen Howard, Regional Health Sciences Librarian & Visiting Assistant Professor, Library of the Health Sciences - Peoria, Library of the Health Sciences - Peoria, Peoria, IL; Rebecca Raszewski, AHIP, Associate Professor & Information Services Librarian, University of Illinois at Chicago, Library of the Health Sciences, Chicago, IL; Patricia Eathington, Assistant Proffessor, School of Nursing, School of Nursing, Macomb, IL; Krista Jones, Interim Regional Director and Clinical Assistant Professor, UIC College of Nursing Urbana Region , UIC Library of Health Sciences, Broadlands, IL; Naomi M. Twigg, Clinical Assistant Professor, University of Illinois at Chicago, College of Nursing, Chicago, IL

Objectives: Reaching the public health nursing (PHN) workforce for continuing education training poses multiple barriers including time commitments and geographic accessibility. An interdisciplinary team of librarians and nurses, Nursing Experts: Translating the Evidence (NExT), was formed to provide this training. Two modalities of instruction on Evidence-Based Practice (EBP) and Knowledge Translation were developed, an in-person session and an asynchronous online tutorial.

Methods: The instructional goals for the training includes introducing high quality, free government resources available from the NLM/NIH, summarizing Brownson's model for evidence-based public health, and applying a case study to demonstrate knowledge translation into daily practice, policy development, and grant writing. For in-person sessions, nurse and librarian dyads provide a three-hour educational session to Illinois public health departments. The online asynchronous tutorial was developed with the same objectives to provide access to a wider geographic audience. Training effectiveness was measured using pre/post survey methodology. Measures will include: familiarity with EBP content, EBP inquiry, resource allocation, confidence with EBP discovery and application, and perceived understanding of the information provided.

Results: Preliminary results for the in-person session indicate all measures of frequency before and anticipated use with the EBP concepts and resources use show improvement. The participants (n=68) were able to develop new skills (m=4.06, SD=.968) and were able to find evidence-base literature (m=4.16,

SD=.980). The online asynchronous tutorial was launched in January of 2016 and is currently being advertised to the public health nursing workforce. The comparative analysis will be expanded using relevant statistical calculations when sample size is larger and tested for normality.

Conclusions: At this time, initial results indicate positive findings with participants attending the in-person sessions. Additional data will be added and analyzed after four more in-person education sessions and as more participants take the online tutorial. Comparative analysis will occur once data is procured and will be presented in the conference poster. Findings will help inform further development of continuing education training for the PHN workforce.

Poster Number: 66

Time: Tuesday, May 17, 2016, 2:00 PM – 2:55 PM

Evaluating Clinical Librarian Services Provided by a Solo Librarian

Elizabeth Laera, AHIP, Medical Librarian, McMahon-Sibley Medical Library, Princeton Baptist Medical Center, Birmingham, AL

Objectives: In 2012, a solo hospital librarian began attending patient rounds with a teaching team. The service has been generally well received but there is no solid evidence to demonstrate the effectiveness of the clinical librarianship program. This study seeks to determine if the services provided by the librarian were effective, practice-changing, and educational to residents on the monthly service.

Methods: The clinical librarian at a 499-bed urban community teaching hospital participates in rounds twice weekly and accompanies a general inpatient medicine team consisting of a hospitalist, an upper level internal medicine resident, three internal medicine or transitional year interns, and a clinical pharmacist. The librarian's main role is to answer point-of-care questions and make notes on topics that require further research. Information is synthesized and delivered with accompanying literature to the team.

Using literature on evaluating clinical and hospital librarian services, a survey was created using SurveyMonkey. The survey was distributed monthly, for nine months, to participants via email. Follow up emails were sent after a week to ensure maximum response rate. Residents were asked to rate the effectiveness and usefulness of the librarian's services, as well as the level of impact the services had on his or her practice.

Results: No results have been reported, but the author expects the results to yield quantifiable data that speaks to the study's objective and the continuation of the clinical librarianship program.

Conclusions: Health science librarians have always sought to determine the effectiveness of their services. Several recent studies and systematic reviews have continued this trend. This particular study, conducted by a solo librarian and focusing on residents, seeks to contribute another perspective and add to the body of data validating the usefulness of clinical librarians.

Poster Number: 68

Time: Tuesday, May 17, 2016, 2:00 PM – 2:55 PM

Nuts as a Healthy Food: How to Search This Popular Research Topic in PubMed

Eric Rumsey, Librarian, Hardin Library for the Health Sciences, Hardin Library for the Health Sciences, Iowa City, IA; Janna Lawrence, AHIP, Deputy Director, Hardin Library for the Health Sciences, University of Iowa–Iowa City; Xiaomei Gu, Clinical Education Librarian, Hardin Library for the Health Sciences, Hardin Library for the Health Sciences, Iowa City, IA

Objectives: Nuts as a healthy food is a very popular research topic. Two of the top eight articles in an Altmetric.com survey of the top 100 research articles in 2013 were on nuts in the diet. With the importance of nuts, we will offer tips on how to efficiently search this subject in PubMed.

Methods: Like searching many plant-based foods, locating articles about nuts in PubMed is tricky. "Nuts" is a Medical Subject Heading (MeSH) in PubMed, but because it is not an explodable term, it often does not retrieve articles on specific kinds of nuts. As an example, most articles mentioning specific types of nuts, like walnuts, almonds, or hazelnuts, in the title are not indexed with the MeSH term "Nuts." Instead, they are often indexed only by their botanical name and have no food- or nutrition-related MeSH terms. In our poster, we will look at search examples for different kinds of nuts and discuss strategies for maximizing retrieval.

Poster Number: 71

Time: Tuesday, May 17, 2016, 2:00 PM – 2:55 PM

Tailored Customer Service in a Consumer Health Library Setting

Erin Culhane, Information Specialist/Librarian, UHN Patient & Family Education Program , Peter and Melanie Munk Patient & Family Learning Centre and Library, Toronto General Hospital, University Health Network , Toronto, ON, Canada

Objectives: To tailor an existing generic organizational customer service initiative to engage patients, families, and community members seeking consumer health information as partners in their care. In addition to evaluating customer satisfaction, this initiative aimed to evaluate the existing library volunteer training program.

Methods: Electronic and paper surveys were used to gain feedback on the implementation by library staff and volunteers of the "Tailored 4As" framework of customer service in a small consumer health library/learning centre in a large, acute-care hospital. The original "4As" - Acknowledge, Ask, Act, Ask Again were extended to personalize library patron interactions, considering patron's time, personality, mood, social determinants of health, past interactions, health literacy level and more. .

Results: Online and in-person surveys indicated that library patrons were extremely satisfied with the service they received in the consumer health library. In most cases, respondents reported that they were welcomed and offered assistance by library staff or volunteers (Acknowledge, Ask), their requests were acted on in a timely and professional manner (Act), and that it was explained to them how to follow up if they needed more information (Ask Again). Informal feedback provided in-person or via email echoed these comments. Respondents also commented on their appreciation tailored aspects of the service of a private setting, welcoming staff, speed of response to health information inquiries, provision of information in other languages, and appreciation for the availability of computers in the library for self-directed research. Volunteers also expressed that the framework provided a useful guide for effective interactions with patrons, especially those who were not as experience in a customer service role or were not confident in their English-speaking skills. These surveys, in combination with in-person real-time feedback, reinforced current volunteer and staff training techniques.

Conclusions: A generic customer service framework can be a useful tool in training library staff and volunteers if appropriately tailored to meet the diverse and often sensitive needs of patients, family caregivers and community members seeking consumer health information.

Poster Number: 73

Time: Tuesday, May 17, 2016, 2:00 PM – 2:55 PM

Strategic Planning for Interagency Academic Health Libraries

Erin L. Menzies, Southern Medical Program Librarian, University of British Columbia Okanagan Library, University of British Columbia Okanagan Library, Kelowna, BC, Canada; Kristina McDavid, MD Undergraduate Librarian, University of British Columbia, Vancouver, BC, Canada

Objectives

Our objective was to develop a fiscally responsible and innovation-centred five year strategic plan for our interagency library program. Librarians play a substantive role in the University of British Columbia's undergraduate medical program. Medical Undergraduate Library Committee (MDULC) membership includes librarians from University of British Columbia's Vancouver and Okanagan campuses, the University of Victoria, the University of Northern British Columbia, as well as librarians from health authorities across the province.

Methods

In June 2015 our committee was asked by the leadership of our medical program to outline our plans for the five year period of September 2015 to September 2020. This was the first opportunity for the MDULC to formally articulate long-term goals and programmatic intentions, and as such we approached the matter systematically. First, university librarians focused on gathering relevant information on library collections, services and staffing, information technologies and pedagogy, while health authorities (which receive some medical program funding to support staffing and collections) were asked to report to the committee's executive on the challenges and opportunities at clinical sites. Second, the Committee executive analyzed and condensed reports from all parties into one cohesive document, collating themes and ensuring alignment with the existing strategic plans from both UBC and the Faculty of Medicine.

Results

Thematic analysis indicated that our undergraduate library committee should prioritize the following during the period from 2015-2020: 1) Facilitate Student Success 2) Facilitate Research Excellence 3) Enhance Culture of Assessment 4) Foster Environment of Continuous Learning.

Conclusions

The five year plan was submitted for consideration to our overseeing body, the Curriculum Committee, and was accepted. The plan was then presented to the University Medical Education Executive and was approved. This document has helped the MDULC to clarify goals and has assisted us in developing a project timeline for the next five years. Furthermore, it has allowed us to articulate major issues, such as budget shortfalls and the negative effect of foreign exchange on our collections budgets.

Poster Number: 76

Time: Tuesday, May 17, 2016, 2:00 PM – 2:55 PM

Teamwork Builds the Compassionate-Care Mosaic for Medical Library and Foundation Collaborators

Evelyn Kobayashi, AHIP, Manager of Library Services, Kaiser Permanante Greater Southern Alameda Area, Kaiser Permanente-San Leandro Medical Center, Health Sciences Library, San Leandro, CA; Naomi T. Saks, Director Spiritual Care, Kaiser Permanente Greater Southern Alameda Area, Kaiser Permanente - San Leandro Medical Center, Spiritual Care, San Leandro, CA; Kimberly S. Welty, HealthCare Ombudsman Mediator, Kaiser Permanente Greater Southern Alameda Area, Kaiser Permanente - San Leandro Medical Center, Administration, San Leandro, CA

Objectives: Our poster describes an innovative collaboration between a Kaiser Permanente medical librarian and The Schwartz Center for Compassionate Care at a new Kaiser campus. Selected library resources paired with monthly meeting topics support our common mission to encourage compassionate care, to enrich the quality of staff interactions, and to increase awareness of library services. Cultural and linguistic competence are emphasized in serving our diverse community.

Methods: The case-based Schwartz Rounds program focuses on the emotional experience of caregivers and was brought to Kaiser Permante-San Leandro in July 2014. An explicit effort was made to reach out to all staff - including administrators and clinicians, nurses, environmental services, security, and hospital volunteers. The medical librarian was invited to attend, and she offered to select and provide journal articles related to monthly topics, lists of subject-related book titles, and journals accessible through the medical library. Provision of background reading consistently integrated with the total program has not been done before in any of the approximately 375 Schwartz Rounds-participating hospitals. We have recently changed from print format to pdfs linking articles as “Resources” on the hospital’s intranet home page, and we are tracking the influence of this change. Participants are being surveyed twice, and a sample will also be interviewed.

Results: The collaboration of the KP-San Leandro medical library and the Schwartz Rounds program is popular with hospital staff who attend - typically between 80 and 120 persons. Recently 96% who replied to a survey rated the collaboration of significant benefit, and 31% reported downloading articles from GSAA's Schwartz Rounds "Reading Resources" on the facility's home page. Conclusions: We will continue to collaborate in presentation of useful resources and marketing to our audience. Drawing in more participants is expected to

continually benefit quality of care and compassion throughout the hospital community.

Poster Number: 79

Time: Tuesday, May 17, 2016, 2:00 PM – 2:55 PM

Building Connections: Outreach During the First Year as a Liaison Librarian

Francisco J. Fajardo, Clinical Engagement Librarian, Florida International University, Florida International University Medical Library, Miami, FL; Gregg A. Stevens, AHIP, Public Services Librarian, Monroe F. Swilley, Jr. Library, Mercer University, Atlanta, GA

Objectives: To describe the outreach efforts of two health science liaison librarians within the first year of their new positions, and to assess the effectiveness of these activities.

Methods: Because serving patrons is a challenge as a librarian new to an institution, two new liaison librarians in different library settings engaged in multiple outreach activities in order to connect with health sciences faculty and students during the first months in their positions. These activities included arranging and attending faculty meetings, setting up outreach tables, and creating email distribution lists. Each librarian recorded and analyzed his reference transaction information to assess the effectiveness of his efforts. Early contacts led to further activities to increase the librarians' visibility, such as library orientation sessions and research symposiums.

Results: Through early and frequent engagement, both librarians met a significant portion of their faculty, residents, and students within the first year. Patron contacts for both librarians were dominated by students and residents, comprising approximately 70% of the contacts for both librarians.Over the course of the year, the reference transactions for each librarian increased exponentially (16.75% rate of increase for the pharmacy and health professions librarian and 12% for the medical librarian). They also correlated their activities to the increase in interactions by using SPSS statistical analysis, which indicated a slight statistical significance.

Conclusions: Increased engagement has led not only to more reference interactions but it has also led to increases in requests for instruction, library access, literature searches, and other forms of research help and collaboration. However, as awareness of librarian services increases, there may be challenges in providing high-quality service to increasing numbers of patrons. The collaborations with faculty, residents, and students should lead to improved access to medical information.

Poster Number: 82

Time: Tuesday, May 17, 2016, 2:00 PM – 2:55 PM

Preserving Departmental History: Development and Implementation of a Photo Archive for the Kellogg Eye Center

Gale A. Oren, Librarian, Kellogg Eye Center, Kellogg Eye Center, Ann Arbor, MI

Objectives: The University of Michigan's Department of Ophthalmology was originally established in 1872 as part of Ophthalmology / Otolaryngology, and later became the Department of Ophthalmology and Visual Sciences. Known since 1985 as the Kellogg Eye Center, the department has a very rich history of people, events, building projects, milestones, and growth.

The photos and images documenting this rich history, however, were scattered over dozens of locations within the eye center, and the prospects of finding an image of a particular person or event were quite challenging. More often than not, this type of time-consuming inquiry ended up with the library. It was also found that frequently, when faculty/staff retired, historically valuable photo collections retired as well.

In response to this predicament, a departmental photo archive was developed, populated, and made available to users. The benefits are many, including the ability to easily identify and retrieve images for speaker introductions, presentations, slideshows, memorials, and events, etc.

This poster will describe the implementation process, challenges, and uses of this archive.

Methods: After gathering support and resources for the initiation of this project, a number of platform options were considered. Several models of indexing, image storage, and retrieval were reviewed and decided upon. Photos and images of interest were identified, workflow processes for scanning, indexing, and storage were established and implemented for students assisting with the project. Methods for maintaining and updating the archive are currently under discussion.

Results: As of September 2015, most of the scanning has been completed, and approximately 25% of the images have been indexed in the archive. Even though the archive has not officially been rolled out to the department, word has gotten out, and a number of requests have been filled. Feedback has been very positive.

(To be updated)

Conclusions: TBD

Poster Number: 85

Time: Tuesday, May 17, 2016, 2:00 PM – 2:55 PM

Building Collaboration: Librarians from Six Institutions in Two Countries Forming a Team to Support the Cochrane Urology Group

Gretchen M. Kuntz, Director, Borland Health Sciences Library, Borland Health Sciences Library University of Florida-Jacksonville, Jacksonville, FL; Mary E. Edwards, AHIP, Assistant University Librarian, Reference and Liaison Librarian, Health Science Center Library, Biomedical and Health Information Services, University of Florida Health Science Center Library, Biomedical Health and Information Services Department, Gainesville, FL; Jennifer Lyon, AHIP, Biomedical and Translational Research Librarian, Health Sciences Library, Stony Brook University Health Sciences Library, Stony Brook, NY; Bernadette Coles, Library Manager, Cardiff University, Cancer Research Wales Library, Cardiff, United Kingdom; Anne Cleves, Assistant Library Manager, Cardiff University, Cancer Research Wales Library, Cardiff, United Kingdom

Objectives: Trial Search Coordinators are integral roles in Cochrane Working Groups, collaborating with authors to review and develop search strategies and updating the Cochrane Register of Studies used to publish the Cochrane Library Controlled Trial database. In 2013 our institution became host to the Prostatic Diseases and Urologic Cancers Group. Concerns regarding time restraints led to development of a distributed model.

Methods: In 2013 we were invited to become Trial Search Coordinators (TSCs) for the Cochrane Prostatic Diseases and Urologic Cancers Group (PDUC), newly based on our campus. Two librarians who had worked closely with authors at a satellite PDUC office in the UK also expressed interest in working as the PDUC TSCs. We formed a TSC group with one lead and 4 assistants. In 2014, we learned that the PDUC group would be taking over urologic topics, so in anticipation of expanded protocols/reviews, we recruited an additional 8 librarians from 3 other universities/institutions to build a team of 13. The advantages of our work include increased visibility and collaboration opportunities on our own campuses, the ability to share projects and resources across institutions, and the opportunity to peer review search strategies to improve output quality.

Poster Number: 88

Time: Tuesday, May 17, 2016, 2:00 PM – 2:55 PM

Seeing the Big Picture: Exploring Health Sciences Librarians' Participation in Non-Library Conferences

Holly J. Thompson, Informationist; Lisa M. Federer, Research Data Informationist; National Institutes of Health Library, Bethesda, MD

Objectives: This study investigates health sciences librarians' (HSLs) participation in non-librarian conferences and meetings. Aims include illuminating how HSLs keep current with information relevant to their patron groups, apply knowledge and experience attained at non-library conferences, and obtain funding for these outside activities. This study will also address why HSLs choose to either supplement or opt out of library conferences.

Methods: Two librarians with experience in research design and survey deployment developed a survey on conference attendance for HSLs, which will be distributed by email to relevant library listservs. Responses will be collected electronically and will provide quantitative and qualitative data on attendance, preferences, and other key considerations made by HSLs when choosing to attend a non-library conference. Data were analyzed using quantitative and qualitative methods to identify themes and trends in HSLs' attendance at and participation in non-library conferences, including the perceived benefits of attending and the relevance of knowledge and skills gained at these conferences to HSLs' daily work. These findings can help identify gaps in library conference offerings and provide ideas to HSLs interested in exploring non-library conferences.

Results: The survey was distributed over national, regional, and local professional librarian listservs. Results were collected electronically and all resposnes remained anonymous.

Conclusions: Health Sciences Librarians attend non-library conferences for a wide variety of reasons. Issues of travel funding, taking time off of work, and perceived benefits all impact an HSL's motivation to participate in a non-library conferences. Further exploration into this area will provide a more substantial understanding into how library conferences can better meet HSLs professional development needs and interests.

Poster Number: 99

Time: Tuesday, May 17, 2016, 2:00 PM – 2:55 PM

Evaluating Fair Use: Creating and Customizing a Checklist for Your Library

John D. Jones, Jr., Instruction & Curriculum Librarian | Associate Professor | MSIS |, University of Colorado Anschutz Medical Campus, Health Sciences Library, Education & Reference, Aurora, CO

Objectives: The main goal was to find and evaluate different Fair Use checklists to create and customize a checklist for our library. Seeing our process may help other librarians, libraries or institutions who wish to embark on this same journey.

Methods: Using different websites, books, articles and training materials, several librarians from our institution collaborated to create a checklist to help evaluate Fair Use. This checklist was made available to faculty and staff via our Copyright guide as a PDF form for printed or online then print completion. The form was also used in an education session concerning the facts and fictions of Fair Use and Copyright.

Results: Our Fair Use checklist does not really look a whole lot different than most of the others out there. We identified specific health sciences examples when we could and general education examples to help illustrate different ways that materials interact with a Fair Use checklist.

Conclusions: While you might not need to create a Fair Use checklist for your institution (there are many out there and most of them are very similar), you probably should find a way to check-in with your educators concerning their use of materials in your educational or corporate environment. Use a poster like this at their research day or at educational conferences like American Association of Medical Schools national or regional meetings. Staff high traffic areas promoting Fair Use and Copyright feedback.

Poster Number: 102

Time: Tuesday, May 17, 2016, 2:00 PM – 2:55 PM

STARTL: Science Teacher Access to Resources Taught by Librarians!

Joy A. Russell, Systematic Review and Grants Education Librarian, UT Southwestern Medical Center at Dallas, UT Southwestern Medical Center - Dallas / Health Sciences Digital Library and Learning Center, Dallas, TX

Objectives: The STARS Summer Science Camps began in 2008 with a grant from the Howard Hughes Medical Institute's Education Pre-College Division. Our objectives are to provide high school science teachers with experience and increase their confidence in laboratory exercises, and to give a head-start to students who will take the corresponding science classes the following school year.

Methods: Science Teacher Access to Resources was developed in 1991 to improve the quality of science education in our region. The STARS Science Camps are an opportunity for high school teachers to gain experience teaching high school biology, chemistry, and physics, especially in lab exercises that accompany classroom learning. Each camp consists of two master teachers, ten teachers, and thirty students. The students are rising 9th, 10th, or 11th graders who will take the subject in school next fall. Priority is given to teachers with less than 3 years' experience teaching the subject of the camp, especially if they teach underrepresented minorities in science. This session will inform Health Science Librarians how to integrate their computation skills in biomedical science, medicine, and assist faculty in teaching students using hands-on learning in the classroom.

Poster Number: 105

Time: Tuesday, May 17, 2016, 2:00 PM – 2:55 PM

Comparison of Information Fluency Attainment Between One-Shot Instruction and For-Credit Courses in Nurse Anesthesia Program

Kaeli Vandertulip, AHIP, Health Science Liaison Librarian, University of Texas at Arlington Libraries, Euless, TX

Objectives: I will analyze the information fluency attained by students in the Graduate Program in Nurse Anesthesia to determine if students who receive one-shot instruction sessions differ from students who take a credit-bearing research methods course.

Methods: I will analyze the information fluency attained by students in the Graduate Program in Nurse Anesthesia to determine if students who receive one-shot instruction sessions differ from students who take a credit-bearing research methods course. Determination of their fluency will be based on faculty interviews and surveys, student surveys of their own perceived fluency, and an rubric-based analysis of the end product of their literature searches (final papers) to determine if there is a significant difference between the information literacy attainments in both groups. This is particularly important because students who receive their Master's degree from this institution are not required to take the Doctoral health informatics course.

Poster Number: 107

Time: Tuesday, May 17, 2016, 2:00 PM – 2:55 PM

The Mosaic of Translation: An Analysis of Translational Medicine Publications

Karen E. Gutzman, Impact and Evaluation Librarian, Galter Health Sciences Library, Galter Health Sciences Library, Chicago, IL; Pamela L. Shaw, Biosciences & Bioinformatics Librarian, Galter Health Sciences Library, Galter Health Sciences Library, Chicago, IL; Ehsan Mohammadi, Post Doctoral Fellow, Northwestern University, Feinberg School of Medicine, Department of Preventive Medicine, Health and Biomedical Informatics Division, Chicago, IL; Kristi L. Holmes, Library Director, and Associate Professor of Preventive Medicine, Health and Biomedical Informatics Division, Galter Health Sciences Library, Galter Health Sciences Library, Chicago, IL

Objectives: Research impact and evaluation services are incredibly important in the area of translational medicine. One method for evaluation is publication analysis, which plays a valuable role in helping an institution gain insights about its research and clinical activities that might not be discernible from other means, including some of the available institution-level data stores.

Methods:
We used publication analysis and information visualization to evaluate the outputs of translational medicine. We discussed the results of harvesting bibliographic metadata from literature databases, analyzing percentile rankings of publications, and reviewing topical trends over time.
Results:
We found that coverage of journals with a translational focus varies by resource. Additionally, each resource allows for different types and quantities of exportable metadata. Publications were analyzed based on the available metadata, and visualizations were created to show the results of the publication analysis.

Conclusions:
The insights gained through evaluation, in particular using publication analysis, allow both investigators and institutions to convey the benefits and impact of their research and clinical efforts to stakeholders. Publication analysis also provides valuable information that can be used for benchmarking, forecasting, and strategic planning activities.

Poster Number: 112

Time: Tuesday, May 17, 2016, 2:00 PM – 2:55 PM

Institutional Review Boards: A Great Opportunity for Librarians

Kate Krause, AHIP, Research Specialist, Research Services, UT MD Anderson Cancer Center Research Medical Library , Houston, TX; Philip Montgomery, Head of McGovern Historical Center, Texas Medical Center Library, McGovern Historical Center, Houston, TX

Objectives: Two of our librarians currently serve on Institutional Review Boards for two different medical schools. Although serving on an IRB requires extensive training and is a serious commitment of time, we've found it to be a rewarding experience in many ways and we highly recommend other librarians volunteer to serve on IRBs.

Methods: IRBs review proposed experiments to ensure that the rights and welfare of human subjects are protected. Many experiments ("protocols") test new pharmaceuticals, vaccines, medical devices, and surgical procedures that are new and unknown. IRB groups are required to have a "non-scientist" member and librarians can fit into this role perfectly. Librarians do not have to be physicians or have extensive medical knowledge. One of our main tasks is to review the consent form the human subjects sign to make sure that it is written in a language that an average person without a medical background can understand. We also check to make sure that everything in the protocol description is included in the consent form. Our purpose is to make sure people fully understand the experiment they are volunteering for and understand all the possible risks and benefits involved.

Results: Many IRBs have a difficult time finding non-scientist members and are very appreciative of librarian involvement. Most IRB members are appointed for a three-year term. Members receive extensive initial training and ongoing continuing education to keep up to date with the latest issues in biomedical ethics and the research review process. Learning about these topics helps us understand and serve our library users better.

Conclusions: Despite the amount of time required to train for Institutional Review Boards and review protocols, IRBs are a great opportunity for librarians to serve on an institutional-wide committee, network with researchers and administrators, learn what research the schools specialize in, learn how clinical research trials are conducted, learn about medical ethics, contribute to medical science, and promote the library.

Poster Number: 114

Time: Tuesday, May 17, 2016, 2:00 PM – 2:55 PM

Systematic Search and Reporting Techniques Applied to the Gray Literature: A Review of Canadian School Breakfast Program Guidelines

Jackie Stapleton, Liaison Librarian, University of Waterloo, School of Public Health and Health Systems, Kinesiology, Waterloo, ON, Canada; Katelyn M. Godin, PhD Candidate, University of Waterloo, School of Public Health & Health Systems, Guelph, ON, Canada; Sharon I. Kirkpatrick, Assistant Professor, University of Waterloo, School of Public Health & Health Systems, Waterloo, ON, Canada; Rhona M. Hanning, Professor, University of Waterloo, School of Public Health & Health Systems, Waterloo, ON, Canada; Scott T. Leatherdale, Associate Professor, University of Waterloo, School of Public Health and Health Systems, Waterloo, ON, Canada

Objectives: To describe systematic review search methods that were developed and applied to complete a case study systematic review of grey literature that examined guidelines for school-based breakfast programs in Canada.

Methods: A grey literature search plan was developed to incorporate four different searching strategies: i) grey literature databases, ii) customized Google search engines, iii) targeted websites; and, iv) consultation with contact experts. These complementary strategies were used to minimize the risk of omitting relevant sources. Since abstracts are often unavailable in grey literature documents, items' abstracts, executive summaries, or table of contents (whichever was available) were screened. Screening of publications' full-text followed. Data were extracted on the organization, year published, who they were developed by, intended audience, goal/objectives of document, sources of evidence/resources cited, meals mentioned in the guidelines, and recommendations for program delivery.

Results: The search strategies for identifying and screening publications for inclusion in the case study review was found to be manageable, comprehensive, and intuitive when applied in practice. The four search strategies of the grey literature search plan yielded 302 potentially relevant items for screening. Following the screening process, 15 publications that met all eligibility criteria remained and were included in the case study systematic review.

Conclusions: This presentation demonstrates a feasible and seemingly robust method for applying systematic search strategies to identify web-based resources in the grey literature. The search strategy we developed and tested is amenable to adaptation to identify other types of grey literature from other

disciplines and answering a wide range of research questions. This method should be further adapted and tested in future research syntheses.

Poster Number: 115

Time: Tuesday, May 17, 2016, 2:00 PM – 2:55 PM

Seeing the Big (Art) Picture: Developing a Rotating Art Program in an Academic Health Sciences Library

Katherine Chew, Research/Outreach Services Librarian, Health Sciences Libraries, Health Sciences Libraries / University of Minnesota, Minneapolis, MN; Michelle Orr, Assistant to the Director, Health Sciences Libraries, University of Minnesota, Health Sciences Libraries, Minneapolis, MN

Objectives: Develop a rotating art exhibit program to turn an outdated, aesthetically unpleasing 1960s style building interior into a stimulating, thought-provoking, intellectually inspiring atmosphere for studying and learning. This space would foster community-building with the Academic Health Center and campus art community by showcasing student and faculty artwork and serving as an inspirational focal point on healing and the arts.

Methods: In summer 2013, a working group was established to identify spaces for an art gallery in a traditional library building. The group consisted of staff from the Library as well as the University Libraries Art & Architecture librarian. Walk-a-rounds were conducted to inventory and photograph available “white spaces” and discuss each space’s suitability as an art gallery for the display of rotating art collections. Each potential space was evaluated on the degree of security (probability of theft or vandalism), accessibility to patrons, visibility of artwork, versatility of the space (wall art, three dimensional, digital) and the amount of facilities work needed to convert the space into one suitable for displaying art. Concurrently, art exhibit polices and application forms from other health sciences libraries were located and studied; other campus art galleries were visited for inspiration and networking potentials.

Poster Number: 120

Time: Tuesday, May 17, 2016, 2:00 PM – 2:55 PM

Training a Diverse Team on Critical Appraisal Using the AGREE II Instrument

Kelly Lang-Robertson, Information Research Manager, Guidelines & Evidence, Centre for Effective Practice, Toronto, ON, Canada; Hannah Loshak, Project Coordinator, Guidelines & Evidence, Centre for Effective Practice, Toronto, ON, Canada; Claudia Warner-Romano, Project Coordinator, Centre for Effective Practice, Guidelines & Evidence, Toronto, ON, Canada; Apurva Shirodkar, Project Coordinator, Centre for Effective Practice, Knowledge Support Services, Toronto, ON, Canada; Sharmilaa Kandasamy, Project Manager, Centre for Effective Practice, Guidelines & Evidence, Toronto, ON, Canada; Katie Hunter, Manager, Centre for Effective Practice, Guidelines & Evidence, Toronto, ON, Canada; Lindsay Bevan, Project Coordinator, Centre for Effective Practice, Guidelines & Evidence, Toronto, ON, Canada; Jess Rogers, Director, Centre for Effective Practice, Guidelines & Evidence, Toronto, ON, Canada; Claire Stapon, Project Coordinator, Centre for Effective Practice, Toronto, ON, Canada

Objectives: Centre for Effective Practice works to enable appropriate health care through the development and implementation of relevant, evidence-based programs and tools. To support these efforts, our information team developed a comprehensive training program designed to educate staff on critical appraisal and to ensure consistency in guideline evaluation.

Methods: Training was developed by an information manager in order to support staff learning, current work in guideline evaluation and development, and projects that require critical appraisal of evidence. Participants receive in-depth education on the AGREE II Instrument and complete practice exercises that highlight areas that are commonly difficult to appraise. A qualified librarian reviews the exercises, and team members' scores are compared to generate discussion on differences in understanding and interpretation of the AGREE II Instrument's evaluation measures. AGREE II scores are aggregated through a concordance process that flags areas of inconsistencies.

Results: To date, 7 team members successfully completed the training and moved forward to complete over 550 appraisals of guidelines to support a project with the Canadian Partnership Against Cancer. After completing training, 100% of the guideline evaluations reviewed met the predetermined concordance criteria. The training improved the consistency of AGREE II scores and reduced the number of third, independent reviews required to assess the guidelines.

Conclusions: Appraising guideline quality through a validated process with the AGREE II Instrument is widely accepted. However, the consistency of AGREE

scores between reviewers can vary due to differing interpretations and understanding of the criteria, leading to a lack of concordance when comparing scores from different reviewers. Comprehensive training by the information specialist team increased critical appraisal skills and led to a more consistent appraisal of clinical practice guidelines. This program has wider implications for health librarianship, highlighting opportunities to develop training programs that drive greater and more consistent adoption of evidence-based practice.

Poster Number: 121

Time: Tuesday, May 17, 2016, 2:00 PM – 2:55 PM

Marketing a Consumer Health Service: A Mosaic of Cultures to Promote Patient Education

Kelsey Leonard, AHIP, Health Information Services Librarian / Assistant Professor; Sandra Oelschlegel, AHIP, Library Director / Associate Professor; Martha F. Earl, AHIP, Assistant Director / Associate Professor; Donna Doyle, Communications and Programs Coordinator; Preston Medical Library / Health Information Center, Knoxville, TN

Objectives: To demonstrate how combining three distinct cultures—medical center marketing, a private marketing firm, and a medical library's communications and marketing—can promote patient and community engagement in health information.

Methods: Due to the success of the Consumer and Patient Health Information Service, Preston Medical Library relocated from an adjacent building to a space inside the medical center. The new location includes an area designed for health professionals along with a dedicated area for patients and their families. An internal and external marketing plan was developed to promote the new Health Information Center. The plan included: developing brochures and table tents, a new website, banners, a Healthy Tips series authored by a library staff member, and patient room signage. Of note is the success of local television appearances by the Health Information Services Librarian. Each month the librarian proposes a timely topic and creates an information piece to be shared by marketing with the news anchor. Topics to date have been complementary and alternative medicine, melanoma, prostate cancer, health literacy, COPD, and influenza.

Results: Brochures and table tents have been placed in 27 prime locations throughout the medical center and are replenished monthly by a librarian. A large banner near the entrance of the Health Information Center promotes the services. Healthy Tips are published weekly on the hospital's website, e-newsletter, and the CEO's newsletter, with some tips distributed via social media. The tips are also issued via press release to 21 counties. After each television appearance, there is an increase in calls to the library on the topic. For example, following the prostate cancer segment the library received ten calls from community members requesting related information.

Conclusions: Working with marketing has created a unique opportunity to broaden promotion of the new Health Information Center. Television appearances are an effective way to connect to consumers. By combining three

distinct cultures, the Health Information Center's services are more widely known. Community members can play an active role in their healthcare.

Poster Number: 123

Time: Tuesday, May 17, 2016, 2:00 PM – 2:55 PM

Library on Demand: Developing an Education Outreach Webinar Series

Kim Mears, AHIP, Scholarly Communications Librarian, Robert B. Greenblatt, MD Library, Augusta University, Augusta, GA; Kathy Davies, Associate Director of Research, Augusta University Libraries , Robert B. Greenblatt, M.D. Library, Augusta University , Augusta, GA; Lindsay Blake, AHIP, Clinical Information Librarian, Robert B. Greenblatt, MD Library, Augusta University, Augusta, GA; Darra Ballance, AHIP, Assistant Professor, Robert B. Greenblatt, M.D. Library, Augusta University, Evans, GA; Maryska Connolly-Brown, Technical Services Librarian, Hampden-Sydney College, Walter M. Bortz III Library, Hampden-Sydney, VA; Ansley Stuart, Allied Health Information Librarian, Georgia Regents University, Robert B. Greenblatt, M.D. Library, Augusta, GA

Objectives: To describe a collaborative project to host a series of live, recorded instructional webinars highlighting search skills for specific information resources and promoting underused library services for an academic medical center. The series was designed to build on existing knowledge of health databases and introduce new service options to the university and health system.

Methods: Librarians investigated the potential of developing an online webinar series as an alternate instructional venue. The initial step was surveying library employees regarding commonly asked questions or recurring patron learning issues to identify potential webinar topics. Survey results were used to establish a priority order of topics balancing resource training and library services education, and a calendar was established for the webinar series. A major component of the process was identifying the technology platform and determining best practices for the delivery of online instruction. The committee researched and developed guidelines for webinar development. The committee members volunteered to provide technical assistance for both viewers and lecturers, coordinate scheduling, and serve as instructors for the initial webinars. Marketing was a central consideration and a checklist of recommend promotional procedures was created for the webinar instructors.

Results: The webinar series launched in August 2014 with topics scheduled bimonthly. Each webinar is approximately 30 minutes and focuses on topics such as database searching techniques and innovative library services. For example, PubMed citation alerts and NIH compliance were featured. Library faculty and staff have currently completed seven webinars which are available from an online webinar LibGuide as well as the Libraries' YouTube channel. A review of the usage data indicates 663 patrons have viewed the series content

within the past year.

Conclusions: Developing an online webinar series proved to be a viable method to expand the Libraries' educational program across campuses. Resolving technical issues led to greater understanding of tools and limitations of existing software for online webinars. The webinar instructors and the committee members gained skill and confidence in using online tools to highlight key aspects of information resources and library services. Usage data and user surveys will assess the value of content and determine future topics, as well as the potential for expansion of the series to the undergraduate library at the institution.

Poster Number: 125

Time: Tuesday, May 17, 2016, 2:00 PM – 2:55 PM

Development of an Informatics Support Team

Kristen Burgess, Assistant Director for Research and Informatics , Health Sciences Library, University of Cincinnati, Cincinnati, OH; Leslie Schick, Associate Dean of Library Services & Director of the Health Sciences Library, University of Cincinnati, Health Sciences Library, Cincinnati, OH

Objectives: The delivery of health informatics services and resources is a key initiative of the University of Cincinnati Health Sciences Library's (HSL) strategic planning process. The goal is to develop a niche role in creating informatics programming that will advance discovery and innovation in medical care and knowledge through the collaborative development of resources, tools and services.

Methods: In order to develop informatics support at the HSL, a Health Informatics Strategic Initiative (HISI) team researched the opportunities and made recommendations for library informatics programming, including the following: integrate library services into translational science center suite of services as a grant-funded member of the team, teach a for credit informatics course in the UC College of Medicine, and open a lab focused on informatics services to support faculty and graduate students in an academic health center. A white paper with recommendations for steps forward and several key collaborative partnerships has led our research and implementation focus.

Results: During 2015-2016, the HISI reformed with a diverse team from the HSL. An Informatics Lab has opened in the HSL providing access to new software, resources, and consultation services. Subgroups are focusing on the development of a makerspace and marketing new health informatics focused services and spaces. Members of the HISI are now members of UC's Institutional Animal Care and Use Committee, the Institutional Review Board, are grant funded by the Center for Clinical and Translational Science and Training (CCTST), and serve on several CCTST committees.

Conclusions: While there are many groups involved within Health Informatics at UC, the HSL is developing expertise and joining key committees related to informatics work at UC. There is definitely room and willingness for the HSL's informationists and librarians to become increasingly involved in this exciting field.

Poster Number: 128

Time: Tuesday, May 17, 2016, 2:00 PM – 2:55 PM

How Long Does It Take to Paint Your Part of the Big Picture: The Time Librarians Spend on Systematic Review Tasks

Krystal Bullers, AHIP, Assistant Librarian, Shimberg Health Sciences Library, University of South Florida, Tampa, FL; Allison M. Howard, AHIP, Cataloging / Reference Librarian, Shimberg Health Sciences Library, University of South Florida, Tampa, FL; Kristen Sakmar, GME Librarian, Shimberg Health Sciences Library, Shimberg Health Sciences Library, Tampa, FL; Randall L. Polo, Reference/Instruction Librarian, Shimberg Health Sciences Library, Shimberg Health Sciences Library, University of South Florida, Tampa, FL; John J. Orriola, Assistant Librarian, Shimberg Health Sciences Library, Shimberg Health Sciences Library, Tampa, FL

Objectives: Librarians perform a variety of tasks in the systematic review (SR) process. The study objective was to identify those tasks and the time health sciences librarians spend on each portion of the systematic review. This illuminated the librarian's role in the big picture as systematic review collaborators and co-authors.

Methods: The investigators identified librarians or equivalents who work at Association of American Medical Colleges (AAMC) or American Osteopathic Association (AOA) colleges and universities, using a combination of library websites, emails, and phone calls. The IRB-approved, confidential online survey instrument was administered to those identified in the search process. The survey included questions regarding individual tasks and the amount of time dedicated to each.

Results: Of the 185 participants, 143 (77%) indicated they had worked on an SR in the last five years. The total number of SRs (i.e., career SRs) that participants reported ranged from 1 to 500, with a median of five. Reported median time spent on standard SR tasks (in hours) were as follows: initial and follow-up interviews (2), search strategy development (5), search strategy translation (3), search documentation (2), result delivery (2), and writing the methodology (1). Average aggregate time for standard tasks was 26.9 hours with a median of 18.5. The greatest aggregate time reported for these tasks was 217 hours; the least was one hour. Median time spent on instruction was two hours. In addition to the standard tasks, the most frequent SR tasks identified by survey respondents were: inclusion/exclusion criteria development or evaluation, protocol development, and result deduplication. The total SRs worked on did not correlate to task time. However, librarians with more years of SR experience

spent less time on standard tasks. They

were more likely to report pearling references, searching trial registries, and conducting grey literature searches.
Conclusions:

Librarians spend the most time developing the search strategy and translating that strategy to other databases. The librarian’s role in the SR, ranging from mentor to principle investigator, has an important impact on task time. Future surveys should include questions to clarify the librarian's role. The inverse relationship between experience and time spent on tasks suggests a learning curve to the SR process. In addition to years of experience, we speculate that task times vary due to the unique nature of each SR, such as complexity of subject, topical background knowledge, and co-authors’ experience levels.

Poster Number: 131

Time: Tuesday, May 17, 2016, 2:00 PM – 2:55 PM

Engaging Library Users in Transforming Library Design and Experiences

Laurissa Gann, AHIP, Manager, Education and Reference; Maianh Tran. Phi, Education Librarian; Research Medical Library, Houston, TX

Objectives: PURPOSE: The library used a series of attitudinal and behavioral research methods to gather user experience information in an effort to justify our application for institutional funds to improve the library space.

Methods: METHODOLOGY: Over a period of fifteen months, an academic medical library gathered user attitudes, opinions and behaviors on their experience of the library's physical space. Data was collected on our users experience of the library's physical space by employing traditional print and electronic surveys in addition to visual white board surveys. These tools were used to help identify our users' desire for furniture, as well as environmental and technology changes in the library. Beyond traditional attitudinal methods, the library employed behavioral research methods including observing users in the space, and bringing in furniture pieces to allow the users to test and vote for their preference. Data collection methods were varied in order to capture our users diverse communication preferences when responding to assessment techniques.

Poster Number: 132

Time: Tuesday, May 17, 2016, 2:00 PM – 2:55 PM

Building the Mosaic: Challenges and Opportunities of Being Fully Embedded in a User Culture

Lin Wu, AHIP, Instructional Associate Professor and Pharmacy & South Health Science Center Campus Librarian, Texas A&M University, Medical Sciences Library, Kingsville, TX; Joel B. Thornton, Head, Instruction & Liaison Services , Instruction & Liaison Services , University of Arkansas Libraries , Fayetteville, AR

Objectives: An academic health science center library examined ways to better support its College of Pharmacy (COP) located some 250 miles from the main campus library. A needs assessment was conducted to better understand the COP's information needs from the perspectives of administrators, faculty, and students. The information gained highlighted areas where the library could enhance its support model to the COP. An on-site health sciences librarian was physically and culturally integrated into the college. This study will describe the experiences, successes, challenges, and opportunities of the embedded librarian operating within the College of Pharmacy.

Methods: This case study will outline or summarize the librarian's first year interactions with COP faculty, staff, and students. Specific topics to be addressed include techniques used to build librarian-faculty relationships, collaboration with faculty in teaching and enhancing student learning, and involvement in COP activities as a faculty member. Most importantly, the embedded librarian's role and impact as a team member in the college will be discussed.

Results:
The patron demographics of the College of Pharmacy is composed of 46 full time faculty members, 29 staff members, 409 current students, 12 postdocs, fellows, and graduate research associates, and 300 preceptors. Building positive relationship and gaining faculty trust is the first step to create value-added service for the college. Techniques include finding opportunities to participate in college events, communicating with the faculty in different ways about their needs, and requesting access to course syllabi to identify librarian's possible roles. By spring 2016, the librarian delivered 5 lectures to 3 credit courses, spoke at the college's Brown Bag Series on research impact, delivered in-person EndNote classes, and conduct several literature searches for the faculty. The higher level of involvement with the college the more chances will be presented to the librarian.
Conclusions: Many reports exist on embedded librarians being integrated into online courses and delivering stand-alone lectures, but few reports exist on

health science librarians who are physically and culturally fully embedded into academic units. Examining this unique approach will bring freshness to current library liaison programs and to other embedded librarian projects.

Poster Number: 134

Time: Tuesday, May 17, 2016, 2:00 PM – 2:55 PM

Workflow and Testing Processes for Incorporating Custom Patient Education in an Electronic Health Record (EHR)

Linda M. Matula Schwartz, Director, Educational Infrastructure, Library Services, Lehigh Valley Health Network, Allentown, PA

Objectives: To develop a workflow process for integrating 1200 custom written LVHN patient education items into an EHR including metadata collection, HTML preparation, image display, testing procedures, quality review, and incorporation of selection guides to guide providers in selection of appropriate patient education content.

Methods:
During 2015, a variety of approaches were researched to incorporate custom patient education into a new EHR rollout for our institution. Third party vendor module to build custom content tested but proved unusable. Initial experiments with HTML documents was promising. Building upon workflows from MLA colleagues from the University of Michigan and University of Virginia, initial testing provided proof of concept. Display of pictures in documents required a solution.

Results:
•Developed/tested process for identifying keywords and ICD codes to ensure relevant display for patients with a particular diagnosis.
•Metadata stored in the materials database.
•Inserting an asterisk as the first character of custom titles ensured display at the top of the Relevant Document list.
•Software was developed to automate conversion of Word documents with accompanying images into HTML documents and to harvest metadata from materials database into the HTML code.
•EHR upload and testing processes established.
•Document formatting and image display tested. Uploading image folders with documents successful in maintaining picture display.
•Guide sheets to assist providers in the selection of appropriate materials created.
•Sheets to document the use of print items from vendors or too large for bedside printing were created.
•Quality checking done by testing in sandbox prior to uploading to live environments using pre-upload and post-upload checklists.
•Master index feature used to link to education sources not integrated into the

EHR's patient education activity.
•Educational tip sheets developed to teach providers to select, assign, and print custom materials and linked resources.

Conclusions: Incorporating custom patient education into an EHR can be difficult but is feasible for medical librarians with integrated third party content and Information Services and/or EHR vendor support. Documents can be successfully uploaded with image content intact and automatic documentation in the EHR with printing upon discharge or with the after-visit summary. Current testing underway to display custom documents selected for patients in the patient's online portal EMR for repeated viewing. Coding inconsistency still requires investigation.

Poster Number: 137

Time: Tuesday, May 17, 2016, 2:00 PM – 2:55 PM

iPads for Patients: Sharing Authoritative Health Care Resources

Lisa A. Marks, AHIP, Director of Libraries, Mayo Clinic, Staff Library, Scottsdale, AZ; Carol Ann Attwood, AHIP, Medical Librarian/RN, Patient and Health Education Library, Patient and Health Education Library, Scottsdale, AZ; Nicole Pettenati, Program Associate, PCORI, Bethesda, MD; Diana Almader-Douglas, Librarian, Mayo Clinic, Hospital Library, Phoenix, AZ

Objectives: In a large multispecialty group health practice in the Southwestern United States, a plan was developed to teach and assist consumers to find authoritative health information using mobile devices/tablets to research and utilize health care information to meet their health information needs.

Methods: Helping those with limited health literacy in their use of mobile devices/apps for health information searches was a primary goal of this project. This poster will focus on the methods utilized to connect consumers with the devices, the interdisciplinary team members involved, the communication process, the identification of priority needs, evaluations completed and future directions that the group/project plans to pursue. Methods included working in a multidisciplinary group to define the issues, brainstorm possible solutions, develop a plan to implement the process, and the development of an evaluation tool that would be used throughout the pilot that would reflect needed changes in the process.

Results: Despite focused marketing efforts in our 6-month pilot time frame, there were few checkouts. Our plan is to re-pilot the project in our new consumer health library on a different campus to see if there will be more patron interest in borrowing mobile devices.

Conclusions: Consumers will be better served in their search for health information if they are assisted to find reliable, authoritative health information on disease topics as well as clinical research being undertaken, with the assistance of a medical librarian and with assistance on navigating sites and apps on a mobile device. With the re-pilot, a redesign to determine patients' specific needs in relation to mobile devices will be explored. The re-pilot will also make note of location comparing the initial ambulatory setting to a hospital setting.

Poster Number: 139

Time: Tuesday, May 17, 2016, 2:00 PM – 2:55 PM

Seeing the Big Picture in Big Data: Using National Institutes of Health Funding Opportunity Announcements to Track the Use of "Common Data Elements" in Awarded Grants and Scholarly Literature

Liz A. Amos, Librarian, National Library of Medicine, National Information Center for Health Services Research and Health Care Technology, Bethesda, MD

Objectives: • Identify and analyze National Institutes of Health (NIH) funding opportunity announcements (FOA), grants and publications that encourage, recommend, or require use of NIH "Common Data Element" (CDE) collections and initiatives. • Develop strategies to track publications from awarded grants that require/encourage CDEs in data collection and methodology.

Methods: A mixed methods approach applying content and citation analysis will use NIH resources to identify and analyze funding announcements and awards. NIH funding opportunity announcements (FOAs) that specify language encouraging, recommending or requiring the use of NIH Common Data Elements will be identified from the NIH's Office of Extramural Research's grant guide (grants.nih.gov). NIH RePORTER (Research Portfolio Online Reporting Tools) will be used to generate a list of awarded grants by fiscal year to match by FOA number. NIH RePORTER will also be used to identify awarded grants by project titles, key terms, and abstracts. Finally, a literature search in PubMed and PubMed Central will create a list of PMID/PMCIDs and associated grant numbers to compare against previously identified grant numbers to analyze gaps and determine additional avenues for tracking NIH funded research in this topic area.

Poster Number: 142

Time: Tuesday, May 17, 2016, 2:00 PM – 2:55 PM

Impact of Hospital Librarian's Participation in the Magnet Program: A Solo Librarian's Journey

Luzviminda N. Sinha, AHIP, Medical Librarian, Dayton Children's Hospital, Dayton, OH

Objectives: Background: When Dayton Children's Hospital embarked its journey to Magnet status in August 2009 and finally earning it in November 2013, opportunity unfolded before the medical librarian to be part of the journey. As "Magnet requires organizations to develop, disseminate and enculturate evidence-based criteria that result in a positive work environment for nurses…,"1 the medical librarian's knowledge of literature searching comes in handy. As part of the dissemination of evidence-based practice in nursing, EBP mentors from each department form a group of mentees, called EBP scholars, to do a certain project. After they finalized their PICOT questions, they are now introduced into doing literature search. This is where the medical librarian comes in. Objectives: To evaluate the impact of the medical librarian's participation with regards to: a. Searching skills of the nurses participating in the EBP program b. Role of the medical librarian and services of the medical library c. Collaboration between the medical librarian and the nursing staff 1http://www.nursecredentialing.org/magnet/programoverview

Methods: Methods: The medical librarian gives a lecture/demonstration session on how to do literature search to a group of EBP scholars in the training computer laboratory in the hospital. After the lecture/demonstration, each mentee does her own literature search with the guidance of the medical librarian. After the session, a short survey questionnaire is used to collect data regarding the mentee's comfort level in doing the search themselves. Observations on how the mentees compose the search terms, select articles and the types of articles selected are also noted. Anecdotal data from interviews are also taken.

Poster Number: 145

Time: Tuesday, May 17, 2016, 2:00 PM – 2:55 PM

Connecting Kids and Health Information via Community Partnerships

Margaret A. Hoogland, AHIP, Distance Support Librarian, A.T. Still University of Health Sciences, A.T. Still Memorial Library, Kirksville, MO; Ashley Love, Associate Professor, A.T. Still University of Health Sciences, College of Graduate Health Studies, Public Health Faculty Member, Kirksville, MO

Objectives: A.T. Still Memorial Library received $1500 from the National Networks of Libraries of Medicine K-12 School Partnerships grant program. Money will be used to update the NEMO Heart Health Corporation website during Fall 2015. Knowledge of a sixth grade student regarding health information will be assessed at William Matthew Middle School in February 16-March18th, 2016, during Melissa Kinney's Library Class.

Methods: A pre-test will be conducted to assess a sixth grade student's knowledge on accessing and using health related information. Then, students will choose to do a minimum of one module and potentially more, if they are interested and have time, on Fitness, AEDS and CPR, Nutrition, or Obesity. Lastly, the post-test will help determine, if the students learned anything by participating in the study. These activities will be conducted during library period in February 2016 under the direction of William Matthews Middle School Teacher, Melissa Kinney. Modules, pre-test, post-test, and informed consent will be obtained before the testing period for all participants and the study was approved by the A.T. Still University of Health Sciences Internal Review Board on August 31, 2015. Dr. Love was instrumental in providing advice for creating valid questions for both the pre-test and post-test.

Poster Number: 146

Time: Tuesday, May 17, 2016, 2:00 PM – 2:55 PM

Piecing It Together with the Outreach Evaluation Resource Center's (OERC's) Planning and Evaluating Health Information Outreach Projects Booklets

Margot G. Malachowski, AHIP, Community Outreach Librarian, Health Sciences Library, Health Sciences Library, Springfield, MA

The Outreach Evaluation Resource Center (OERC) provides support for health information outreach evaluation through the National Network of Libraries of Medicine (NN/LM). In 2013, OERC released the second edition of the *Planning and Evaluating Health Information Outreach Projects* booklets.
The Health Sciences Library at Baystate Health identified an objective to use these booklets to plan and evaluate a community engagement project done in collaboration with The Literacy Project, an adult literacy organization operating in Franklin County, Massachusetts.
The structure of our community engagement project, guided by the OERC booklets:
Step 1: Discovered lack of library-initiated health information outreach to Franklin County, MA.
Step 2: Learned that non-profit The Literacy Project received hospital community benefits funding for health literacy project focusing on print materials.
Step 3: Confirmed the most prevalent diseases and social determinants of health in the 2013 Community Health Needs Assessment for Franklin County, Massachusetts.
Step 4: Identified inputs, activities and outcomes for community engagement project with The Literacy Project to teach online health information resources.
Step 5: Based assessment questions on short-term desired outcomes (e.g. increased awareness of library resources, increased confidence in searching for health information).
Step 6: Designed a blend of opinion scale survey questions with open-ended survey questions.
The OERC booklets provide practical suggestions for planning and evaluating health information projects.
***Lessons Learned:* Chose the elements that best meet the circumstances of the project—no need to do everything listed in the booklets!**
***Impact*: Writing the final report was simplified by adhering to the structure provided by the booklets.**
The OECR booklets are available for download at https://nnlm.gov/evaluation
Additional resources are located at http://guides.nnlm.gov/oerc/tools
The community engagement project was funded in part with federal funds from

the National Library of Medicine, National Institutes of Health, under contract #HHSN-2762-0110-0010-C.

Poster Number: 148

Time: Tuesday, May 17, 2016, 2:00 PM – 2:55 PM

Combining Active Learning and Technology to Teach Evidence-Based Practice: Opportunities for Library Involvement

Mariana Lapidus, Associate Professor, Reference Coordinator, Henrietta DeBenedictis Library, Henrietta DeBenedictis Library, Boston, MA

Objectives: - To introduce innovative instructional methodology that utilizes active teaching techniques in order to: o enhance library instruction integrated into the evidence-based decision making course for dental hygiene students o reinforce learners' knowledge of research databases o develop core information competencies - To present ways to assess the effectiveness of bibliographic instruction and study students' perceptions of in-class activities

Methods: Library instruction, incorporated into the required hybrid Oral Health Research course on evidence-based practice, has a goal to teach dental hygiene students research methods and database searching techniques. In 2015, the traditional didactic lecture format of bibliographic instruction was completely revised in order to include problem solving exercises with a particular emphasis on case studies. 65 students from two campuses, enrolled into the course, utilized secondary databases, previously introduced by a librarian, to solve clinical scenarios and find the best evidence. Videoconferencing technology and Blackboard course management system were used to create a virtual classroom, provide easy access to course materials, and create a student-centered learning environment. A set of additional questions pertaining to bibliographic instruction activities was included into the online course evaluation survey to assess the effectiveness of the new instructional methodology and student satisfaction level.

Results: Students expressed positive attitude towards participating in in-class activities, but, due to the extremely low online survey response rate, only anecdotal data exists indicating that their knowledge of online resources and evidence-based searching skills have somewhat improved. It was not possible to statistically measure the impact of the library instruction session on students' performance, and more formal evaluation of the new teaching methodology is necessary in 2016. Conducting a paper-based pre-and post-testing of dental hygiene students enrolled into the course next year is proposed as an effective assessment instrument of students' learning satisfaction and the effectiveness of the class design.

Conclusions: Interprofessional faculty collaboration between the Department of

Dental Hygiene and the Library led to the creative integration of active learning methods into the course curriculum and significantly enhanced student learning experience. Even though the formal assessment of the new instructional techniques has not been successful yet, there are reasons to believe that this approach, combined with the use of technology, could be effectively applied by academic librarians in a variety of courses in order to engage students from multiple campuses into synchronous learning process, improve their problem-solving abilities, and provide high-quality information literacy instruction.

Poster Number: 153

Time: Tuesday, May 17, 2016, 2:00 PM – 2:55 PM

Designing Cross-Program Curricular Support: The Library Supports Evidence-Based Medicine Across Graduate Medical Education

Megan von Isenburg, AHIP, Associate Director, Research and Education, Medical Center Library & Archives, Duke Medical Center Library, Durham, NC

Objectives: The EBM in GME project seeks to investigate all trainees' current evidence-based medicine (EBM) skills and attitudes; to inventory current program educational opportunities; to develop additional educational materials; to create a cross program in-person course, and to make available a shared repository of course materials to foster and support EBM training across all programs in Graduate Medical Education (GME).

Methods: The library created an advisory group of key faculty from GME programs across the institution, representing multiple specialties. With support and guidance from this multidisciplinary team, two surveys were disseminated to (1) trainees and (2) program leadership. Results were analyzed by specialty and by role/level. A focus group was held with trainee volunteers to further study the perceived importance of EBM, their confidence in practicing EBM, the resources most often utilized, current teaching methods, and preferred learning methods. Data was transcribed and coded. In addition, accreditation milestones relevant to EBM were analyzed for all programs. Results indicated that trainees strongly prefer in-person education opportunities over online tutorials. Therefore, a multidisciplinary, in-person course was developed and launched, and assessed.

Poster Number: 154

Time: Tuesday, May 17, 2016, 2:00 PM – 2:55 PM

Isn't MeSH Enough? Medical Subject Headings for Systematic Review Searching: A Preliminary Look

Melanie Anderson, Information Specialist, Library and Information Services, University Health Network, Toronto, ON, Canada

Objectives: Librarians performing searches for systematic reviews rely on subject headings in major databases to differing degrees, and the extent to which keywords are used has a significant impact on the time required for searching. We will perform a preliminary test of the effectiveness and reliability of Medical Subject Headings for retrieving key papers indexed in OVID Medline for systematic reviews.

Methods: We selected six systematic reviews published between 2012 and 2015 from those identified by DARE as having performed adequate or better searches. Three strategies were tested for each review: the original Medline strategy; a second composed of only the MeSH used in the original strategy; a third composed of the MeSH a current search for the topic would use. The results for each strategy were compared on the basis of which and how many Medline indexed key papers (as selected for inclusion by the reviewers) are not retrieved, the reasons the missing papers may not have been retrieved, the quality of the missing papers as assessed by the reviewers, and the amount of data that would be missed using each strategy on its own.

Results: The strategies using only MeSH terms frequently, though not always, miss key articles identified by the reviewers. The missed articles are sometimes "large," relative to the studies reviewed, and have frequently been rated by the reviewers as being of good quality. Keywords that could have retrieved the missed articles are identified in each case.

Conclusions: In this preliminary test, MeSH only strategies were not reliable in returning all of the key articles identified by the original reviewers. More intensive testing into reviews in different subjects, or involving different population groups, may provide more information about the extent to which keywords must be used when doing reviews in these areas.

Poster Number: 156

Time: Tuesday, May 17, 2016, 2:00 PM – 2:55 PM

"If the Library Genie Granted You Three Library Wishes, What Would They Be?": Results and Lessons Learned from an Annual User Feedback Campaign

Melissa De Santis, AHIP, Interim Director; Vivienne Houghton, Web Services Librarian; Cathalina Fontenelle, Web Applications Developer; Univ of Colorado Anschutz Medical Campus, Health Sciences Library, Aurora, CO

Objectives: This poster describes the creation, results, and lessons learned from an annual "Library Wishes" user feedback campaign.

Methods: From 2013 to 2015, the Health Sciences Library at the University of Colorado Anschutz Medical Campus has done an annual "Library Wishes" campaign. The library first started soliciting wishes in 2013 as part of the strategic planning process and plans to continue the campaign as an annual program. Combining a clever marketing theme with a quick online form, the library has been able to easily obtain feedback from their busy users. This poster presents the process, results, and lessons learned from this program. We share the creative process and technical tools that we used to collect, tag, and share the wishes with users.

Results: Each form submission allowed the user to enter up to three separate wishes. The initial campaign in 2013 generated 254 wishes from 108 submissions. The 2014 campaign generated 349 wishes (37% increase) from 152 submissions (41% increase). The 2015 campaign generated 253 wishes (20% decrease) from 121 submissions (28% decrease).

Conclusions: The wishes were tagged then sorted by tag in order to identify exact counts and clear patterns. The wishes were displayed in a tag cloud to visually differentiate the importance of each tag by font size. Discover which wishes were the most popular and which the "Library Genie" was able to grant. Learn about the wishes that gave the library insight as to which existing services needed additional marketing as well as how a decrease in wishes in 2015 was likely due to a very popular wish from previous years being granted.

Poster Number: 159

Time: Tuesday, May 17, 2016, 2:00 PM – 2:55 PM

Putting "Academic" in Academic Health Sciences Libraries: A Case Study of Two Libraries

Melissa L. Rethlefsen, AHIP, Deputy Director / Associate Librarian, Spencer S. Eccles Health Sciences Library, Spencer S Eccles Health Sciences Library, Salt Lake City, UT; Holly Shipp Buchanan, AHIP, FMLA, Executive Director, Health Sciences Library and Informatics Center, University of New Mexico Health Sciences Center, Health Sciences Library and Informatics Center, Albuquerque, NM; Monica Rogers, Health Information Literacy Coordinator, Creighton University, Health Sciences Library, Omaha, NE; Jean P. Shipman, AHIP, FMLA, Director, Spencer S. Eccles Health Sciences Library, Spencer S. Eccles Health Sciences Library, Salt Lake City, UT

Objectives: Strengthen librarians' roles in the academic environment through seeking equal faculty status and rank. This exploratory qualitative research uses a case study of experiences at two academic health sciences libraries that sought to increase their value by better understanding academic structures and professorial titles, and re-codify how the library and library faculty are defined in local academic environment.

Methods: Research exists on the evolution of U.S. academic libraries as academic units, but little is written about the history and structure of health sciences libraries. The authors are: reviewing the literature regarding the history of academic health sciences libraries and faculty status for librarians; examining institutional historical records and guidelines for creating academic units within their respective institutions; especially regarding faculty designations (tenure status, professorial or equivalent titles) and are using faculty charters to codify their library faculty within the two institutions. This research information will be used to generate a whitepaper about academic health sciences librarians as faculty; identify potential strategies to increase campus perception of the librarians as equal academic unit members; and highlight areas for future research.

Poster Number: 161

Time: Tuesday, May 17, 2016, 2:00 PM – 2:55 PM

An Exploration of iPad Minis in the Clinic and the Classroom

Melynda Ozan, Health & Human Services Librarian, Assistant Professor, Atkins Library, University of North Carolina at Charlotte, Greensboro, NC; Spencer N. Gallant, Student Researcher, J. Murrey Atkins Library, University of North Carolina at Charlotte, Denver, NC; Donna Lanclos, Associate Professor for Anthropological Research, Atkins Library, University of North Carolina at Charlotte, Charlotte, NC

Objectives: A health & human services librarian and an anthropologist conduct an observational study to learn how undergraduate Kinesiology students use iPad minis in lecture class, lab, and field assignments. The purpose of the project is to determine the extent to which the availability of mobile technology influences students' practices.

Methods: This is an exploratory, observational study. A cohort of 20 undergraduate Kinesiology students received school-issued iPad minis for use in clinical and non-clinical academic settings. At the start of the semester (pre-iPad) researchers interviewed students about how they use technology and the web. Later in the semester students were interviewed again on the same topic. The instructor was interviewed about his expectations around technology, learning, and pedagogy at the beginning of the academic year, and then later in the year to track any changes he observed in his own or his students' practices. Researchers also conducted field observations in lab and lecture classes. Because iPad use was restricted in most clinical field placements, these particular observations were discontinued.

Results: Students' decisions and methods for using the devices depended largely on the setting and type of activity they were engaged in. The iPads were used most actively in lab classes where clinical apps were integrated into lab exercises. They were used for information display and reference in both lab and lecture classes. Students appreciated the portability of the iPads for ebook use in class, but many still preferred the print book for more focused studying at home. The majority of students continued to use laptops and notebooks for note-taking and writing.

Conclusions: IPads can be effective learning tools, especially in active learning environments such as lab classes. In order to make full use of these tools, they need to be intentionally and systematically integrated into the curriculum. Policies, training, and support for faculty and students are also needed. Students will see the most unique educational benefit of iPads in active class settings where clinical apps are incorporated into the lessons.

Poster Number: 164

Time: Tuesday, May 17, 2016, 2:00 PM – 2:55 PM

Famous and Lesser Known Members of the University of Michigan Medical School Who Have Made a Difference: A Historical View

Merle Rosenzweig, Informationist, Taubman Health Sciences Library, Taubman Health Sciences Library, Ann Arbor, MI; Anna E. Schnitzer, Outreach Coordinator, University of Michigan, Taubman Health Sciences Library, Ann Arbor, MI; Stephanie M. Swanberg, AHIP, Assistant Professor, Information Literacy & eLearning Librarian , Medical Library, Oakland University William Beaumont School of Medicine, Rochester, MI

Objectives: The University of Michigan Medical School (UMMS) opened its doors in 1850. Since that time it has become renowned for numerous alumni who have made an impact in the world. Although some are well known, others have played a role that most people are unaware of. Here we will highlight the diverse individuals and their achievements.

Methods: The contents of this poster were searched for and recovered from: the Bentley Historical Library, the archives of the University of Michigan; the University of Michigan Medical School's Center for the History of Medicine whose primary objective is to foster a broader understanding and deeper appreciation of the pioneering role of the University of Michigan, its alumni, and Michigan as a state in advancing knowledge of disease and promoting human health.

Results: Through our research, we have been able to highlight several well-known and lesser-known University of Michigan alumni who have had an impact on various areas of life, both medical and political. In addition, some of them have conquered barriers of ethnicity and gender.

Conclusions: In this poster we have brought to light a number of the notable alumni of the University of Michigan Medical School.

Poster Number: 169

Time: Tuesday, May 17, 2016, 2:00 PM – 2:55 PM

Creating the Region 7 Mosaic: Three Conversations for a Better Community

Montie' Dobbins, Head, Access Services, Health Sciences Library, LSU Health Shreveport, Shreveport, LA; David C. Duggar, AHIP, Head, Library Liaison Program, LSU Health Shreveport, Health Sciences Library, Shreveport, LA; Paula Craig, Head Librarian, Library, NSU University Libraries, Shreveport, LA

Objectives

Librarians are partnering with key organizations in the Louisiana "Region 7" to offer feedback, services, and symposium opportunities addressing three factors that impact the seven northwestern parishes. By inserting themselves into the conversations of Homeland Security and Local Emergency Preparedness Planning committee, and Healthy Louisiana meetings, a mosaic of community health and safety preparedness is forming in Region 7.

Methods

Librarians have the opportunity to work with community personnel on three projects that will impact northwest Louisiana. In 2015, the Red River experienced near-record flooding in the region. As waters recede, librarians are working with responders and planning committees to develop "situational awareness" resources and tools for personnel who respond to disasters. Next, the Louisiana Office of Public Health is developing the Healthy Louisiana plan to assess health priorities and determine an action plan in each region. Librarians are at the table, offering feedback and support to stakeholders. Finally, through a 2015 Disaster Preparedness Award, a symposium for local law enforcement, emergency personnel, and librarians will address potential intruder threats and how to respond. This symposium will culminate in a mock scenario. When communities are in need, it is a great time for librarians to insert themselves into the conversation.

Results

While librarians and responders worked together to define "situational awareness" and how to best facilitate a tool for responders to utilize, a national disaster funding award was not received. The Healthy Louisiana assessment is still in the development phases as it was put on hold in light of the need to direct state efforts to flood response. Now back on track, librarians are present to provide input as the State Health Improvement Plan (SHIP) moves forward. The "Intruder Alert" symposium has had a positive regional response. Within two weeks of opening, registration is near maximum capacity and a waiting list is expected. Through all of these efforts, librarians remain an active part of the mosaic of Louisiana Region 7 community health and safety.

Conclusions

When the relationship between Region 7 responders and the librarians began, it was limited to serving as two information sources testing potential usefulness. Through these and past projects, these groups have gone from “source” to “partnership”. The symposium will be a culmination of the efforts of this partnership’s commitment to the information needs of Region 7 responders and librarians.

Poster Number: 172

Time: Tuesday, May 17, 2016, 2:00 PM – 2:55 PM

Building the CORE: Reassembling Pieces to Train Ophthalmologists Locally and Globally

Nancy T. Lombardo, AHIP, Associate Director for Information Technology; Christy Jarvis, AHIP, Head of Information Resources and Digital Initiatives; Spencer S. Eccles Health Sciences Library, Spencer S. Eccles Health Sciences Library, Salt Lake City, UT

Objectives: Create a significant training resource for clinical ophthalmologists, using locally produced content and representing the expertise of the eye center Support training of students, residents and fellows at the local institution and at ophthalmology training programs internationally Identify and make accessible the vast wealth of knowledge and information generated at the eye center

Methods: This academic health sciences library was approached by the institution's eye center to collaborate with them in developing a comprehensive online tool for training clinical ophthalmologists. Many valuable resources exist, but are unavailable because they have not been collected, organized or made accessible. An organizational structure was developed and refined in partnership with the eye center faculty. Flexibility is built in to accommodate variations in local and global training programs. Content sources were identified and systematic collection procedures were established to capture those outputs. The library established procedures and manages the process for submission, review and inclusion as materials are produced. This process provides opportunities for students, residents and fellows to build their publication portfolio by contributing to a peer-reviewed online educational repository.

Results: At the end of year one, the library has been instrumental in establishing the infrastructure for the resource, and for establishing the submission and peer review processes. The poster will provide usage and collection statistics that reflect the growth and wide spread adoption of the resources collected.

Conclusions: This project is an example of a successful library and department collaboration. Libraries are uniquely positioned to assist departments in at their institution to collect, organize and publish intellectual output into meaningful digital educational resources. These resources not only enhance the local teaching mission, but can have global impact as they also support the institution’s international outreach initiatives.

Poster Number: 174

Time: Tuesday, May 17, 2016, 2:00 PM – 2:55 PM

Prevalence of Link Rot in 2016 Health Journals

Nancy Schaefer, AHIP, Associate University Librarian, Health Science Center Library, Biomedical and Health Information Services, Health Science Center Libraries, Gainesville, FL

Objectives: Link rot (a broken/dysfunctional link) complicates the tracking of a source. This project aims to collect data on link rot prevalence to present locally to increase use of repositories and permanent links in citations. It may lay the foundation for a larger study to provide quantitative rather than anecdotal data to influence journal standards and reviewer training.

Methods: Prevalence rates over time, discipline and nature of journal for three journals targeting health professional readership will be compared. Information on re-direction and methods of effective full-text recapturing (such as the Wayback Machine) will be collected and categorized. This information will be shared with relevant local audiences, with short pre-/post-surveys on willingness to locate permanent links and utilize the local institutional repository administered. Methods that facilitated recapture of rotted links will be shared. The project's effectiveness in raising awareness and changing author behavior will be evaluated via survey results and citation questions from the targeted groups.

Results:

References in original and research articles in the January issue of one professional association journal each in family medicine, otolaryngology and occupational/environmental medicine in 2016, 2014 and 2011 were examined. Citations containing http or www demonstrated some link rot in the very month published, with the percentage of link rot increasing in the second year after publication and as much as 50% of links rotted (dysfunctional) by 5 years after publication. Journals that had instituted clickable reference links in PDFs differed in method and appearance of the linked full-text. Clickable links were sometimes more reliable in delivering full-text than typing the URL into a new browser. No DOI functioned as a clickable link. HTTP 404 ("Page not found") and 400 B ("Bad request") were the most frequently-occurring messages.

Conclusions:

This pilot project raised faculty awareness of the speed and prevalence of link rot and of methods to recapture "lost" references. Its narrow focus on well-known journals in a convenience sample of health specialties (those which the

author serves as liaison) should be expanded into non-association journals in other allied health and medical disciplines to increase generalizability.

Poster Number: 176

Time: Tuesday, May 17, 2016, 2:00 PM – 2:55 PM

Pinterest as a Virtual Curation Tool for Health Resources

Nathalie Reid, AHIP, Manager, Information and Resources & Manager, CE Program, National Child Traumatic Stress Network, National Child Traumatic Stress Network, Los Angeles, CA; Cathryn Chiesa, Resource Librarian, National Child Traumatic Stress Network, National Child Traumatic Stress Network, Durham, NC

Objectives: This paper/poster examines how the social media platform Pinterest can be used in libraries/information centers as an appealing method to curate digital resources because of its visual format, ease of access to resources and searchability of the site. In addition, we will demonstrate how this platform is ideal for the dissemination of health information particularly resources for mental health professionals.

Methods: This paper/poster examines how librarians at the X X X X Network strategically expanded their use of social media in disseminating children's mental health resources from Facebook and Twitter to Pinterest. Using data from Google Analytics, the librarians discovered based on traffic redirects that the main audience of their resources, mental health professionals in the field of child trauma, were already accessing the Network's resources via user generated posts on boards. Once the librarians discovered users were currently using Pinterest to feature X X X X X resources, they created a Pinterest account and website; developed content for all X X X X X resources; marketed the website; and monitored website traffic. Currently, the X X X X X Pinterest page has over 400 pins across 21 subject related boards (e.g. domestic violence resources; juvenile justice resources; complex trauma; etc.).

Poster Number: 179

Time: Tuesday, May 17, 2016, 2:00 PM – 2:55 PM

The Integrated Library System-Less Library: Providing Efficient Access and Managing the E-Resource Life Cycle

Nicole Mitchell, Information Technology Librarian and Associate Professor of Library & Information Sciences; Lisa Ennis, Information Technology Librarian and Professor of Library & Information Sciences; Alabama College of Osteopathic Medicine, Library/Learning Resource Center, Dothan, AL

Objectives: The aim of this project was to evaluate the need for a traditional ILS in a new and 99%, fully electronic medical library and to assess alternative systems for providing the smoothest access and management of resources.

Methods: Librarians at a new medical school (just two classes in progress) analyzed and evaluated the systems put into place early in the school's development. Since the library does not have a print collection and does not circulate materials, a traditional ILS proved to not be the best system for resource discovery or management. The library used the catalog for access to e-books and EBSCO's A-to-Z for e-journal access. Given the nature of the library collection, librarians decided to research tools that were designed to manage and provide access to electronic resources from the outset. Both commercial and open source products were considered and evaluated.

Results: We ultimately chose Intota which is designed to manage and provide access to electronic collections with no legacy print components to take up systems space and time. The integrated A-Z list is fully customizable and offers title and subject searching for both e-journals and e-books. Summon provides a discovery layer that includes whatever content we want and system administration is streamlined and provides both search and usage statistics at various levels. In addition, Intota is fully integrated with Springshare's LibGuides, providing dynamic content for course pages and mobile devices.

Conclusions: Librarians ultimately decided in favor of implementing Intota which includes Summon, 360 Link, Intota Asessment, and 360 Resource Manager. By streamlining the management of and access to library resources, the librarians also streamlined the work for themselves, the students, faculty, and staff. Usage statistics and lessons learned from implementation will be included in the poster data.

Poster Number: 181

Time: Tuesday, May 17, 2016, 2:00 PM – 2:55 PM

Snowed Under: Geospatial Data Literacy for Health Students

Francine Berish, Geospatial Data Librarian , Queen's University, Joseph S. Stauffer Library, Kingston, ON, Canada; Paola Durando, Co-Acting Head, Queen's University, Bracken Health Sciences Library, Queen's University, Kingston, Kingston, ON, Canada; Sandra Halliday, Health Sciences Librarian, Queen's University, Bracken Health Sciences Library, Kingston, ON, Canada; Amanda Ross-White, AHIP, Health Sciences Librarian, Queen's University, Bracken Health Sciences Library, Kingston, ON, Canada

Objectives: To integrate geospatial literacy into graduate level public health library instruction in order to develop data and information visualization skills.

Methods: The ability to represent large and complex data as easily understandable “infographics” contributes to the rising popularity of information visualization technology and its increasing utilization in academic research. Geographic information systems (GIS) and geospatial data are being leveraged for research in health sciences including; disease prevention, healthcare delivery, social determinants of health, epidemiology, health promotion and for multivariate analysis of health outcomes. As part of their embedded information literacy curriculum, in the 2015 Fall Term, 35 Master of Public Health students were introduced to the use of geospatial data in public health. The session included examples of health-specific information visualizations,and how to discover, access, and use open and licensed data. Students were also introduced Library GIS software and training opportunities.

Results: Although some struggled with the identification and description of tools, students demonstrated a clear understanding of how geospatial data can be leveraged to address public health issues.

Conclusions: Instruction on geospatial data, tools and data visualizations is a good fit in public health and has broad potential in health sciences education.

Poster Number: 184

Time: Tuesday, May 17, 2016, 2:00 PM – 2:55 PM

Supporting Comics and Graphic Novels in a Health Sciences Library

Patricia F. Anderson, Emerging Technologies Informationist, Taubman Health Sciences Library, Taubman Library, University of Michigan, Ann Arbor, MI; Whitney A. Townsend, Informationist, Taubman Health Sciences Library, UM Library, Ann Arbor, MI

Objectives: And increasing number of healthcare providers and patients are working in what has become known as "Graphic Medicine," a publishing space focused through the lens of comics and graphic novels as a tool for storytelling, case studies, education, awareness, and outreach. Requests for services in support of these creative and learning activities led to our engagement in this space.

Methods: Services provided in the comic and graphic medicine space range from basic collection support (in partnership with other campus and community libraries) through resource selection, support, events, to actual comic creation. The library has a regular workshop on comic creation as a form of digital literacy, integration of comics in education, and includes a substantial resource guide of mobile apps to support sustainable comic creation for non-artists. The LibGuide created to support our faculty working in this space includes the mobile apps as well as recommended titles by topic, resources, and examples of published faculty works. The library hosted a campus-wide event on comics in the creation of awareness around stigma, stereotypes, and bullying. Our most exciting creative efforts include a collaboration with the Emergency Medicine department, and a proof-of-concept experiment creating a webcomic about a librarian superhero.

Results: [in progress]

Conclusions: [in progress]

Poster Number: 185

Time: Tuesday, May 17, 2016, 2:00 PM – 2:55 PM

Systematic Review Services on Health Sciences Library Websites

Patricia F. Anderson, Emerging Technologies Informationist, Taubman Health Sciences Library, Taubman Library, University of Michigan, Ann Arbor, MI; Emily C. Ginier, Health Sciences Informationist, University of Michigan, Taubman Health Sciences Library, Ann Arbor, MI; Nandita S. Mani, AHIP, Assistant Director, Academic & Clinical Engagement, Taubman Health Sciences Library, Taubman Health Sciences Library, Ann Arbor, MI

Objectives: In order to inform development of a marketing plan for our systematic review services, we undertook a complete review of how systematic review services are discussed and displayed on the web sites for health sciences libraries.

Methods: Working from a list of United States health sciences libraries provided by AAHSL, the web sites for all listed libraries were reviewed for the presence or absence of an official systematic review support service (SRSS). Verification was done by two reviewers and three methods: homepage review; services page review; and text word search within the site. When a question arose as to whether or not a library provided a SRSS, the reviewers either achieved consensus or referred the final decision to a third party. In addition to review for SRSS, the web sites were also reviewed for alternative novel services. For all libraries for which the web site provided an identifiable SRSS, patterns were identified, and best practices were gathered.

Results: [in process]

Conclusions: [in process]

Poster Number: 187

Time: Tuesday, May 17, 2016, 2:00 PM – 2:55 PM

The Metric Mosaic: Evaluation of Research Impact

Virginia M. Carden, AHIP, Administrative Research Librarian, Duke University, Medical Center Library, Durham, NC; Susan H. Keesee, AHIP, Research Impact Librarian, Duke University, Medical Center Library, Chapel Hill, NC; Patricia L. Thibodeau, AHIP, FMLA, Associate Dean for Library Services & Archives, Medical Center Library & Archives, Medical Center Library & Archives, Durham, NC

Objectives: When making decisions and requesting additional resources, departmental chairs require objective measures for evaluating faculty and their departmental research impact. Administrators are seeking ways to compare and track productivity and impact beyond citation counts for a specific person or set of articles. Library skills and knowledge can be used to leverage new and existing resources to provide additional metrics.

Methods: The Library had developed an extensive faculty publication database that provided disambiguation of names, as well as identified departments, divisions, and faculty appointments. The chair of surgery wanted information beyond this 10 year database and traditional citation numbers. This involved looking at H-index factors for entire careers as well as immediate work, and then developing comparisons by individual researcher, division, and highly ranked journals. Surgery also wanted to explore alternative metrics that might indicate broader impact in the field. Because the university's alternative metric tool requires current publications in the faculty profile system, a process was created for updating the citation records.

Results: The Library created search strategies for culling the citations for an individual over their career as well as recent work, while generating H-indexes for both. Current CVs were used to track prior institutional affiliations. EndNote libraries were created for each faculty member. These searches were also developed so that they could be repeated and updated annually. Individual reports on citation counts and H-indexes were generated. The Library then reviewed publications in faculty profiles to ensure they were accurate and complete to support the analysis conducted by the metrics tool.

Conclusions:

The project leveraged the library's expertise, while exploring new ways to compile data from existing and new resources that support programmatic decisions in the medical center. While this provided very complete and accurate data on faculty publications, it's a very time-consuming process that could not be sustained without additional financial and staff support.

Poster Number: 189

Time: Tuesday, May 17, 2016, 2:00 PM – 2:55 PM

Systematic Review Classes for Non-Librarians

Rachael Posey, Pharmacy Librarian, University of North Carolina at Chapel Hill, Health Sciences Library, Raleigh, NC; Jennifer Walker, Cancer Information Librarian, University of North Carolina at Chapel Hill, Health Sciences Library, Chapel Hill, NC; Brenda Linares, AHIP, Outreach Librarian, Health Sciences Library, Chapel Hill, NC

Objectives: To evaluate a class series on conducting systematic reviews (SR) for non-librarians. Classes were aimed at external constituents of the health affairs schools at the University of North Carolina at Chapel Hill. The primary audience included early career researchers, post-docs, graduate students, and faculty new to the SR process. All classes were conducted at the Health Sciences Library.

Methods: The series consisted of six stand-alone sessions designed to enhance participants' knowledge of the individual parts of the SR process. Subjects covered included history and definition of SRs, topic refinement, literature searching, citation management, study selection and assessment, and presenting results. This series was conducted over three months and co-taught by two librarians per session. Classes combined lecture with interactive activities on parts of the SR process. During these interactive activities, class attendees participated in screening studies, using the citation managers EndNote and RefWorks, and evaluating a published SR. Four of the six classes were streamed to an online audience via Blackboard Collaborate. Two of the most interactive classes (citation management and study selection and assessment) were not streamed due to the dependence on in-class participation in those classes. Participants received post-class surveys via email following each session

Results: In total, 47 people attended the classes, averaging 16 participants per class: 15% faculty; 34% staff; 45% students, fellows, post-docs, or residents; and 6% "other." Approximately one-third of attendees were from the School of Medicine and one-third were from the School of Public Health, with smaller groups attending from the other schools, health care system, and the library. Post-class surveys were emailed to attendees following each session. Approximately 30% of attendees returned the post-class survey. Instructors' knowledge and responsiveness to questions received the highest ratings, with a median score of "Excellent." Overall, 88% of respondents agreed that the classes improved their knowledge of the subject area, and 89% said they would recommend the class to a colleague.

Conclusions:

Due to positive feedback from class participants and high demand, a third series

is currently being offered. The series has been modified from a six-class series to a two-class series in order to better accommodate student and faculty schedules and to increase retention throughout the series.

Poster Number: 192

Time: Tuesday, May 17, 2016, 2:00 PM – 2:55 PM

Life After "The Big Deal": An Examination of Methods and Usage in the Wake of Cancellation of a Comprehensive E-Journal Package

Randall Watts, AHIP, Assistant Director for Resource Management, Medical University of South Carolina Library, Medical University of South Carolina Library, Charleston, SC

Objectives: This paper will examine the impact of MUSC's cancellation of our "Big Deal" with Wiley.

Methods: As a result in the worldwide economic downturn, MUSC Library cancelled access to our comprehensive package of ejournals from Wiley beginning in January 2010. The Library previously had access to over 1600 Wiley titles and reduced that number to 70. This was supplemented with "Article Select Tokens" from Wiley. The paper will discuss how the Resource Management Services (RMS) department was required to alter workflows. We will examine usage statistics from the titles within this collection from both before and after the cancellation will be compared. Additionally, we will discuss the impact on our users and the potential to duplicate the results with other publishers.

Results: A review of statistics from both before and after the cancellation reveals the impact upon our users. The RMS department was able, through trial and error, incorporate article "token" use into its existing workflow and successfully deliver non-subscribed content to our users. As such, the impact upon our users has been minimal while the savings have been tremendous.

Poster Number: 195

Time: Tuesday, May 17, 2016, 2:00 PM – 2:55 PM

Piecing Together the Mosaic of Rural Clinician Information Practices over a Twenty-Year Period

Rick Wallace, AHIP, Assistant Director, Quillen College of Medicine Library, Quillen College of Medicine Library, Bristol, TN; Nakia Woodward, Sr. Clinical Librarian, Quillen College of Medicine Library, Quillen College of Medicine Library, Johnson City, TN

Objectives: The purpose of this study is to determine how the information practices of rural clinicians in fifteen counties have changed over the last twenty years. This data is needed to design programs to meet the information needs of the population.

Methods: This study is a longitudinal cross-sectional study. A validated survey methodology was used to gather data at a specific point in time. Physicians' names were gathered from the state licensing verification database and librarians' personal knowledge. Advanced practice and registered nurses were identified from a list from the state center for nursing. The questionnaires were sent by mail with a self-addressed stamped return envelope with a cover letter explaining the purpose of the survey. Returned surveys were accepted for a 6 week period. The physicians surveyed were the complete population of a fifteen county area and nurse/nurse practitioners were a random sample of the population. Previous iterations were done in 1998 and 2009.

Results: In 1997, names of physicians and nurses in 17 rural Tennessee counties were obtained. A random sample (p=.05) was surveyed for a total of 707-(357 RNs and 350 MDs). In 2009, this exact procedure was replicated with slight adjustments to the survey instrument (477 RNs and 312 MDs=789). Eighteen percent (125) of the information questionnaires were returned in 1997. In 2009, sixteen percent (124) of usable surveys were returned. In 2015,one hundred thirty useful surveys were collected. Clinicians were measured as to information barriers, resources, access points, smartphone use, and practice demographics.

Conclusions: We need to be out in the community improving access to health information. A longitudinal, cross-sectional study is a good methodology to map progress and trends. Making changes in the community is hard. Hospital purchases by large corporations affect outreach opportunities.

Poster Number: 198

Time: Tuesday, May 17, 2016, 2:00 PM – 2:55 PM

Adding More Tiles to the Mosaic: The Library and Evidence-Based Medicine in a Time of Curriculum Revision

Roberta Bronson Fitzpatrick, Associate Director, George F. Smith Library of the Health Sciences, Newark, NJ

Objectives: Librarians contributed evidence-based medicine curriculum. The CATs project provides instruction on basic EBM concepts: the evidence pyramid and levels of evidence, formulating a searchable clinical question, using the PICO format, searching PubMEd. Articles were read, synthesized, and turned into a CAT. These skills will be used in clinical rotations and residency. The resulting CATs would populate a database/institutional repository.

Methods: Medical students enrolled in their "Foundations" course were simultaneously enrolled in an "Evidence Based Medicine" Moodle course. Module 2 covers developing searchable clinical questions, breaking them into a PICO format and selecting from among the available library resources when conducting a search. Librarians have embedded exercises within the course.

Students are divided into small groups and paired with a partner. The small groups convene in the library for one hour for more focused training on searching PubMed, as well as a quick review of concepts covered in the Moodle course. Students work with their partner to write a focused clinical question, break it down into PICO, connect to PubMed, execute a search, identify at least four relevant articles. They obtain the articles, read and synthesize them, and write a CAT with a clinical bottom line.

Results: An online form was developed and is used to write the CATs, which are submitted directly to the library and to the course director. The completed CATs are presented by each pair to their small group and preceptor. Medical school faculty preceptors have commented on the quality of scholarship exhibited, students' facility with PubMed, and their understanding of article types and the PICO format exhibited during small group discussions.

The library developed a relational database to house the completed CATs but is in discussion to import them into RUCore, the institutional repository.

Conclusions: There is a high degree of interest in this project. Students feel that

they are developing a skill that has application for the duration of their career. Faculty and administrators are enthusiastic about the fact that this is not EBM in a vacuum, but for lifelong learners.

Poster Number: 202

Time: Tuesday, May 17, 2016, 2:00 PM – 2:55 PM

Leaving the Lecture Behind: Putting PubMed Instruction into the Hands of the Students

Rose Louise. Turner, Research & Instruction Librarian, University of Pittsburgh, Health Sciences Library System, Pittsburgh, PA; Andrea M. Ketchum, AHIP, Reference Librarian, Health Sciences Library System, Health Sciences Library System, Pittsburgh, PA; Melissa A. Ratajeski, AHIP, Coordinator of Data Management Services, Health Sciences Library System, Health Sciences Library System, Pittsburgh, PA; Charles B. Wessel, Head of Research Initiatives, Health Sciences Library System, Research, Instruction, and Clinical Information Services, Pittsburgh, PA

Objectives: OBJECTIVE: This project's objective was to develop a new method to deliver PubMed instruction to first-year medical students in order to increase learning and engagement.

Methods: METHODS: Reference librarians in an academic health sciences library revised the standard lecture-style PubMed training session typically provided to first year medical students. The librarians developed four step-by-step exercises, each of which focused on an important aspect of PubMed: Automatic term mapping, MeSH Database, Advanced Search Builder, and MyNCBI. After the librarians provided a brief PubMed introduction, students were given time to work through one of these exercises in a small group. During the working session the librarian was available for clarification and to facilitate discussions. Once the exercises were completed, students taught their peers the assigned PubMed topic while the librarian interjected with additional explanation as needed. Two questions relevant to the library instruction were included in the final course evaluation to measure success.

Poster Number: 208

Time: Tuesday, May 17, 2016, 2:00 PM – 2:55 PM

Using Report of No Retrieval to Improve Search

Sara Pimental, AHIP, Senior Consultant, Care Management Institute, Care Management Institute, PETALUMA, CA

Objectives: Clinical Library, the internal Kaiser website for clinicians was experiencing a large amount of no (zero) results every time a search was done on Kaiser content. We investigated ways to improve this.

Methods: We changed the way search worked in two way. First, instead of just searching titles, headers and keywords we broadened the search to full text. This was not completely successful. There is a thesaurus that works with search and we have the ability to add synonyms. In this way we don't have to add keywords to every document. The zero results reports were analyzed to see if there were keywords that could be added to the thesaurus to improve retrieval.

Results: We had a reduction of 25,636 in searches that resulted in 0 results.

Conclusions:

Poster Number: 220

Time: Tuesday, May 17, 2016, 2:00 PM – 2:55 PM

The Outreach Panorama: How Engaging Are Your Library's Digital Communications Campaigns?

Siobhan Champ-Blackwell, Health Sciences Librarian, Disaster Information Management Research Center, National Library of Medicine, Bethesda, MD; Jamie Peacock, Management Analyst--Communications, Office of Strategic Business Operations, HRSA--Health Resources and Services Administration, Rockville, MD

Objectives: To discover the impact of increased breadth of digital communications from the library to users. The staff at a national library moved all their digital communications from a handful of listservs and rss feeds to an email subscription service. The main goal of the platform migration was to gather better delivery, open, and click metrics from each bulletin sent through the service. Analysis of this data allows staff to identify user interests and behaviors, and thus better align the library's communications with user needs.

Methods: Staff researched and identified a communications tool that would allow for gathering delivery, open, and click data within content delivered via email subscription. Staff then identified the tools the library was already using to communicate with its various user populations (listserv, RSS, social media) and set up parallel communications within the new tool. After six months, staff was able to measure the increase in subscribers via the new tool, identify what type of information was most “clicked," and determine the organization's ROI from its digital communication effort.

Poster Number: 222

Time: Tuesday, May 17, 2016, 2:00 PM – 2:55 PM

Awareness, Adoption, and Application of the Association of College and Research Libraries Framework for Information Literacy in Health Sciences Libraries

Stephanie J. Schulte, Associate Professor/Head, Research and Education Services, Health Sciences Library, The Ohio State University, Columbus, OH; Maureen Knapp, AHIP, Research Support & Education Librarian III, Rudolph Matas Library of the Health Sciences, Tulane University - Matas Library, New Orleans, LA

Objectives: To determine 1) if health sciences librarians are aware of the recent Association of College and Research Libraries Framework for Information Literacy; 2) if they have used the framework to change their instruction or communication with faculty and if so, what changes have taken place; and 3) if certain librarian characteristics are associated with the likelihood of adopting the framework.

Methods: This study used a descriptive survey methodology, distributed electronically, targeting health sciences librarians in general academic, academic health sciences, and hospital library settings. Appropriate email distribution lists associated with the Medical Library Association were used to recruit participants. Questions related to the study objectives in addition to demographic characteristics were included. Demographics included type of library setting, average amount of time spent teaching, professional memberships, previous use of ACRL information literacy standards, and service to curriculum committees at their institutions. All responses were anonymous. Analyses included descriptive statistics, cross-tabulations and chi-square tests of pairs of questions.

Results: Preliminary analysis indicates that academic health sciences librarians (AHSLs) represented more than 60% of respondents, while a quarter of respondents were hospital librarians (HLs). About half of all respondents were aware of and were using or had plans to use the framework. AHSLs and general academic librarians were more likely than HLs to be aware of the Framework; however, most HLs and about half the AHSLs were not using nor had plans to use the framework in their instruction efforts. Those with more than 20 years of experience as a librarian were also less likely to be aware of the Framework and more likely to have no plans to use it. Respondents who were members of ALA or ACRL were more likely to be aware of and tended to be more likely to be using or have plans to use the framework. Those using the Framework were

mostly revising and creating content, revising their teaching approach, and learning more about the framework. This group commented that it was influencing how they thought and talked about information literacy in classes and with faculty, especially the authority frame. Common reasons for not using the Framework nor having plans to use it were being unaware and not being involved in formal instruction activities.

Conclusion

This exploratory descriptive study indicates that there is room to improve awareness and application of the Framework for Information Literacy among health sciences librarians.

Poster Number: 226

Time: Tuesday, May 17, 2016, 2:00 PM – 2:55 PM

Quantifying the Systematic Search: An Analysis of Retrieval in Published Cochrane Systematic Reviews

Tara Landry, Librarian, McGill University Health Centre, MUHC Libraries, Montreal General Hospital, Montreal, PQ, Canada; Alex Amar-Zifkin, Librarian, McGill University Health Centre, MUHC Libraries - Montreal General Hospital, Montreal, PQ, Canada

Objectives

Searches in support of systematic reviews aim to be as extensive as possible in order to minimize bias. As a result, database searches for systematic reviews generally retrieve a large number of results. The primary objective of this study was to extract relevant statistical data on the retrieval of searches in support of published Cochrane systematic reviews.

Methods

The methods sections and PRISMA flowcharts (when available) of all Cochrane reviews published since 2011 were analyzed to extract the number of records identified through database searching. Data on the number of databases searched, reported use of grey literature sources and clinical trial registries, and citation databases such as Web of Science and Scopus was also extracted. The Cochrane Review Group responsible for each included review was noted, based on the hypothesis that retrieval is influenced by the topic of the review. Extracted data was analysed in Excel.

Results

My results are not complete yet. Descriptive statistics on the retrieval for literature searches in support of systematic reviews, both in general and by review group, method of citation searching and use of core and specialized databases will be presented.

Conclusions

My results are not complete yet.

Poster Number: 229

Time: Tuesday, May 17, 2016, 2:00 PM – 2:55 PM

FOSCEs: Adding Another Library Tile to the Medical School Mosaic

Tom Harrod, Reference/Instructional Librarian, Himmelfarb Health Sciences Library, Himmelfarb Health Sciences Library, Washington, DC; Alexandra Gomes, AHIP, Associate Director for Education, Information, and Technology Services, Himmelfarb Health Sciences Library, The George Washington University, Washington, DC

Objectives: The 2014 launch of the revised medical school curriculum provided new opportunities for librarians to collaborate with faculty. Due to past informatics instruction in the first year curriculum, we were invited to expand this content as part of a new formative Objective Structured Clinical Examination (FOSCE) initiative. This poster will describe the development and implementation of the FOSCE informatics curriculum

Methods: In FOSCEs, small groups of students rotated through simulated patient encounters in order to demonstrate their clinical knowledge and skills. Due to simulation center logistics, students alternated between skills demonstration and informatics activities. The informatics component consisted of fifty minute small group active learning sessions, often including a standardized patient. In these sessions students utilized various information tools and resources to clarify the clinical picture. Two reference librarians worked closely with faculty to develop original case-based scenarios that were taught by a team of six reference librarians over the course of a year. Our goal was to both supplement our existing instructional efforts as well as to expand the range of informatics topics that we cover with students in their first year of medical school.

Results: We feel that our efforts were successful as we were asked to continue these efforts for the subsequent year's FOSCEs. In the process of doing these sessions, we learned many lessons. Among these is that a lone librarian is sufficient to handle the sessions unless the number of students was increased for some reason. In addition, it is important to work with the faculty who are creating the course materials for the more clinical part of the FOSCE session to make sure we are integrating our efforts. Also, having a standardized patient really increased the buy-in from students.

Conclusions: In conclusion, we feel that this was a worthwhile experience for our librarians. It increased our role within the curriculum development process and it gave us another opportunity for face-to-face time with our pre-clinical medical students to teach them important informatics lessons. Additionally,

this effort helped us to extend our role within the curriculum as it gave us the confidence to seek more opportunities and it showed clinical faculty that we could be valuable collaborators.

Poster Number: 232

Time: Tuesday, May 17, 2016, 2:00 PM – 2:55 PM

A Program Evaluation to Determine Preferences of Readers of an Online Newsletter of a Health Sciences Library

Trey Lemley, AHIP, Information Services Librarian, Biomedical Library, Biomedical Library, Mobile, AL; Andrea Wright, Information Services Coordinator & Technology Librarian, Baugh Biomedical Library, Baugh Biomedical Library, Mobile, AL

Objectives: To determine which articles (and category of article) are read the most in an online newsletter produced by librarians and staff of an academic health sciences library, in an effort to ascertain the interests and preferences of our readers, so that the library can provide better service in the future by including articles on popular topics.

Methods: Setting: An academic health sciences library serving a school of medicine, a college of nursing, eight allied health sciences programs, and the general public. Population: Readers of an online newsletter produced by personnel of an academic health sciences library. Research design: In this program evaluation, data from Google Analytics was used to provide information on preferences of readers of the library's online newsletter to determine popularity by topic of article. Researchers ranked over three hundred articles from a 6-year period both by number of pageviews per article and by average time on each page. Articles were further classified by subject, including library services, mobile resources, outreach, reference, resource news, scholarly communications, and web tools. The time of year of publication and number of articles per issue were examined to determine if these factors had any effect on reader behavior.

Poster Number: 235

Time: Tuesday, May 17, 2016, 2:00 PM – 2:55 PM

Publication Metrics: Services and Support

V P. Nagraj, Research Data Analyst; Andrea H. Denton, Research and Data Services Manager; Claude Moore Health Sciences Library, Charlottesville, VA

Objectives: To implement a suite of services in support of the scholarly output assessment needs of individuals, departments and the institution as a whole.

Methods: The [BLIND: LIBRARY NAME] has positioned itself a source of expertise for the [BLIND: INSTITUTION NAME] institutional faculty profiles software. Librarians have demonstrated the system to faculty and met with administrative staff to provide training and assistance in generating relevant reports. These interactions have led to one-on-one consultations with faculty members and administrators interested in specific metrics such as times cited, impact factor, and journal rank. In response, the library's Research and Data Services department, which includes a newly created Research Data Analyst position, has developed and hosted custom web applications to assist in retrieving, processing and analyzing publication data. These apps facilitate a smoother reporting workflow and provide insightful visualization and analysis of publication metrics.

Results: The implementation of a publication metrics support service at [BLIND: LIBRARY NAME] has been extremely successful. Librarians have fielded requests from numerous faculty members regarding assistance with identifying metrics for promotion and tenure applications. Administrators have also approached the library for help generating publication reports for faculty recruitment and retention. Senior-level health system administrators have explicitly recognized the value of the library's service model.

Conclusions: Given that many libraries administer access to database providers of publication metrics, it is within the librarian's purview to offer consultations and support not only for retrieving but also analyzing these indicators. The library can add further value by offering patrons interactive applications for exploring and summarizing scholarly output. With that in mind, it is worth acknowledging possible limitations of this approach. The size of an institution may prohibit its library from unilaterally administering a reporting mechanism or profiling software. But as the landscape of reporting tools and bibliometric indicators shifts, the library can and should be developing new services.

Poster Number: 238

Time: Tuesday, May 17, 2016, 2:00 PM – 2:55 PM

Optimization of Systematic Review Searches: When Can You Stop?

Wichor M. Bramer, Biomedical Information Specialist, Medical Library, Erasmus MC - Medical Library, Gouda, N/A, Netherlands

Objectives: Information specialists confronted with the task of searching for a systematic review try to identify all articles on the desired topic. This requires restructuring the search several times based on input from the researchers. Still it is uncertain when one can stop. Is simply retrieving all known sentinel articles enough or is more effort needed?

Methods: For several mediated searches performed by information specialists from our library the concept search strategy is saved at various intervals during the creation. After the researchers determined the included references for their reviews, these concept search strategies are tested for the retrieval of those references. Does the retrieval in terms of sensitivity and specificity change over the process? Is there a tipping off point where in general most (if not all) included references are retrieved.

Results: Results and conclusions are not available yet.

Poster Number: 241

Time: Tuesday, May 17, 2016, 2:00 PM – 2:55 PM

Teach and Tell: Access Services' Role in the Big Picture

Yini Zhu, Managing Librarian, Head of Access Services, George F. Smith Library of the Health Sciences, Rutgers, the State University of New Jersey, Newark, NJ

Objectives: Within the library, Access Services staff stand at the convergence of users and services and are armed with core functional skills. Despite this, they have traditionally played a passive role in patron-centered outreach. Our objective: to see if Access Services could move into an active role in the bigger picture of promoting library services to enhance academic learning and research.

Methods: The "Teach and Tell" project began with identifying each Access Services Department's (Circulation, ILL, and Media/Computer) unique services and expertise. We aimed to leverage their positions and skills to develop and implement programs to the targeted populations – the faculty, students, clinicians, educators, and staff on the Rutgers Biomedical and Health Sciences campus in Newark, NJ. Potential projects were then identified for each target population. It was determined that new students needed stress-relief programs during exam periods; research faculty and students lacked awareness of ILL services; and new residents, and first year students needed help with technology and connectivity set up. The three departments each then developed their own specialized activities based on their individual expertise to meet these demands: Pop Your Stress Out! (Circulation), Need an Article or Book? (ILL) and Technology Briefing: Go Mobile (Media/Computer).

Results: The three departments' activities were warmly received by students and faculty. Many students commented that the stress release effort helped relieve exam pressure and encouraged that the activity be repeated annually. Approximately 200 students, faculty, and researchers attended the ILL Info session and many requested additional materials for dissemination. The technology briefing was vital to nearly the entire population of new students and crowded the Media Center during the first few weeks of the semester. Via these outreach activities, the Access Services staff contributed significantly to raising awareness of library services and promoting a positive library image.

Conclusions: This project demonstrated that Access Services can go far beyond its traditional, passive desk functions. The success of the project built confidence and motivation among the staff to take on more active roles in patron-centered activities in the future, with multiple project ideas now currently in the pipeline. The "Teach and Tell" project elevated Access Services to get

involved in the bigger picture of enhancing academic teaching, learning, and researching.

Poster Number: 243

Time: Tuesday, May 17, 2016, 2:00 PM – 2:55 PM

Expanding the Clinical Librarian's Role in Education: Partnering with Residency Programs to Support Scholarly Activity

Young-Joo Lee, Senior Clinical Librarian, Louis Stokes Health Sciences Library, Howard University , Washington, DC

Objectives: This study will explore how clinical librarians can further expand their roles as educators in supporting residents, fellows and faculty in their scholarly activity. The model presented here shows a librarian as an instructor teaching within a department regularly throughout the year and keeping office hours. Outcomes of this model will also be measured using surveys and interviews.

Methods: At the author's institution, the educational role of the Clinical Librarian has been limited to a general orientation for new residents and periodic workshop requests by clinical departments. To push beyond this limited involvement and provide improved learning outcomes, the author implemented three new programs: a regular presentation on grand rounds on varying topics related to research, search strategies, writing and publishing; coordination of presentations by guest speakers on writing and publishing; weekly office hours for residents or specific departments. To measure the success of these programs, participants will provide survey evaluations. Program directors will also take part in in-depth interviews to express their views on how the perceived contributions of the librarian have evolved. In addition, the study will report any further positive outcomes achieved as a result of these innovative programs.

Poster Number: 250

Time: Tuesday, May 17, 2016, 2:00 PM – 2:55 PM

Cancer Librarians Section Poster

Poster Number: 251

Time: Tuesday, May 17, 2016, 2:00 PM – 2:55 PM

Collection Development Section Poster

Poster Number: 252

Time: Tuesday, May 17, 2016, 2:00 PM – 2:55 PM

Consumer and Patient Health Information Section Poster

Poster Number: 253

Time: Tuesday, May 17, 2016, 2:00 PM – 2:55 PM

Dental Section Poster

Dental Section, Dental Section, MLA Dental Section, Los Angeles, CA

Poster Number: 254

Time: Tuesday, May 17, 2016, 2:00 PM – 2:55 PM

Educational Media and Technologies Section Poster

Poster Number: 255

Time: Tuesday, May 17, 2016, 2:00 PM – 2:55 PM

Health Association Libraries Section Poster

Poster Number: 256

Time: Tuesday, May 17, 2016, 2:00 PM – 2:55 PM

History of the Health Sciences Section Poster

History of the Health Sciences Section, Melissa Nasea, Chair, Mary Helms, Chair-Elect, NA, N/A

The purpose of the section is to stimulate and support interest in the history of the health sciences among libraries and librarians.

Poster Number: 257

Time: Tuesday, May 17, 2016, 2:00 PM – 2:55 PM

Hospital Libraries Section Poster

Sheila Hayes, Retired, MLA, Broad Brook, CT

Poster Number: 258

Time: Tuesday, May 17, 2016, 2:00 PM – 2:55 PM

International Cooperation Section Poster

Poster Number: 259

Time: Tuesday, May 17, 2016, 2:00 PM – 2:55 PM

Leadership and Management Section Poster

Leadership and Management Section, Executive Board, Leadership & Management Section, MLA, Chicago, IL

Poster Number: 260

Time: Tuesday, May 17, 2016, 2:00 PM – 2:55 PM

Medical Informatics Section Poster

Medical Informatics Section, Medical Informatics Section (MIS), Medical Library Association, Chicago, IL

MIS provides a forum for education and communication among medical librarians, health professionals and other information science professionals. Medical informatics focuses on broad-based computer applications involved in the transfer of information for health care and for the education, research, and administration that support it. MIS helps members keep up with issues and ideas related to informatics.

Poster Number: 261

Time: Tuesday, May 17, 2016, 2:00 PM – 2:55 PM

Nursing and Allied Health Resources Section Poster

Poster Number: 262

Time: Tuesday, May 17, 2016, 2:00 PM – 2:55 PM

Pharmacy and Drug Information Section Poster

Poster Number: 263

Time: Tuesday, May 17, 2016, 2:00 PM – 2:55 PM

Public Health/Health Administration Section Poster

Poster Number: 264

Time: Tuesday, May 17, 2016, 2:00 PM – 2:55 PM

Public Services Section Poster

Poster Number: 265

Time: Tuesday, May 17, 2016, 2:00 PM – 2:55 PM

Relevant Issues Section Poster

Poster Number: 266

Time: Tuesday, May 17, 2016, 2:00 PM – 2:55 PM

Research Section Poster

Research Section, Section , Medical Library Association, Chicago, IL

Poster Number: 267

Time: Tuesday, May 17, 2016, 2:00 PM – 2:55 PM

Technical Services Section Poster

Poster Number: 268

Time: Tuesday, May 17, 2016, 2:00 PM – 2:55 PM

Veterinary Medical Libraries Section Poster

Poster Number: 271

Time: Tuesday, May 17, 2016, 2:00 PM – 2:55 PM

Medical Library Group of South California and Arizona Chapter Poster

Medical Library Group of Southern CA and Arizona. Chapter, MLGSCA, MLGSCA, Los Angeles, CA

Poster Number: 272

Time: Tuesday, May 17, 2016, 2:00 PM – 2:55 PM

Mid-Atlantic Chapter Poster

Poster Number: 273

Time: Tuesday, May 17, 2016, 2:00 PM – 2:55 PM

Midcontinental Chapter Poster

Poster Number: 274

Time: Tuesday, May 17, 2016, 2:00 PM – 2:55 PM

Midwest Chapter Poster

Midwest Chapter, Membership Secretary, Midwest Chapter of MLA, Columbus, OH

The purpose of the Midwest Chapter, Medical Library Association (MLA) is to stimulate and foster interest in health sciences libraries and librarianship; to increase the knowledge of its members by sponsoring educational programs and courses: to encourage development of and cooperation among health sciences libraries; to provide a forum for the exchange of ideas and the discussion of mutual problems and concerns; and to acquaint persons interested in health sciences libraries and librarianship with MLA.
The Midwest Chapter includes the great states of Illinois, Indiana, Iowa, Kentucky, Michigan, Minnesota, North Dakota, Ohio, and Wisconsin. The Chapter welcomes both members and non-members of MLA who are interested in health sciences libraries and librarianship.

Poster Number: 275

Time: Tuesday, May 17, 2016, 2:00 PM – 2:55 PM

New York-New Jersey Chapter Poster

Poster Number: 276

Time: Tuesday, May 17, 2016, 2:00 PM – 2:55 PM

North Atlantic Health Sciences Libraries Chapter Poster

North Atlantic Health Sciences Libraries Chapter, na, na, na, N/A

Poster Number: 277

Time: Tuesday, May 17, 2016, 2:00 PM – 2:55 PM

Philadelphia Regional Chapter Poster

Philadelphia Regional Chapter, Chapter Council Representative, MLA-Phil, Philadelphia, PA

Lean about the MLA Philadelphia Regional Chapter. Join over 80 members in Delaware and the eastern half of Pennsylvania for networking, continutin education, grants, awards and professional development.

Poster Number: 278

Time: Tuesday, May 17, 2016, 2:00 PM – 2:55 PM

South Central Chapter Poster

South Central Chapter, Chapter, SCC/MLA, Shreveport, LA

Poster Number: 279

Time: Tuesday, May 17, 2016, 2:00 PM – 2:55 PM

Southern Chapter Poster
