# Supplementary material for: 116th Annual Meeting, Medical Library Association, Inc., Toronto, ON, Canada, May 13–18, 2016
Source: J Med Libr Assoc. 2017 Jan;105(1):E1–E20. doi: 10.5195/jmla.2017.123 (PMC5234456; doi:10.5195/jmla.2017.123)
Supplement: Appendix [file mosaic16_program_presentations.pdf]

Medical Library Association
Mosaic ’16 Program Session Abstracts

Mosaic | Mosaïque
Be part of the big picture
Faites partie du portrait global
MLA’16 CHLA/ABSC ICLC
May 13–18, 2016 Toronto
www.mlanet.org #mlanet16
Medical Library Association

Any unsolicited abstracts for the annual meeting undergo a process of blind peer review. Abstracts of papers intended for section programs are reviewed by members of a panel of reviewers from the sections sponsoring the programs. The final decision on program speakers rests with the section program planners.

Sunday, May 15, 2016, 3:00 PM – 4:25 PM
Room: 201D

Session: Lightning Talks #1

Around the World in Sixty Minutes: Launching an English Language Learners Conversation Group

Emily Couvillon
Liaison Librarian, TMC Library, Houston, Texas

Rachel R. Helbing, AHIP
Liaison Librarian, Client Relationship Management Department, Houston, Texas

Objectives: Our goal for the English language learners conversation group is to provide international students and staff within our medical center the opportunity to practice English for free in a low-stress environment. We also aim to facilitate networking and cross-institutional collaboration among our international patrons by offering them an informal space in which they can discuss their research interests.

Methods: We identified a need in our medical center for programming that supports practice of conversational English. By attending several sessions of a successful ESL conversation group at a nearby organization, we received advice and resources to help us prepare conversation topics and marketing materials for the launch of our own group. We contacted international student services departments at our patron institutions to spread the word and received enthusiastic responses. We launched the program in the fall semester. English language learners meet for one hour every week in a library conference room. At least one librarian facilitator attends each session to lead students through a variety of conversation topics and ice-breaker activities. Facilitators also answer questions about pronunciation, vocabulary, and idioms. We provide additional information about ESL classes available through local universities for those interested in more formal training.

Results: Although growth in attendance has been slower than expected, retention for patrons who attend is very good. The group has shed light on questions international patrons have about language as well as job seeking, etiquette, and American customs. Each discussion reveals information gaps the library can work to fill for future patrons. The group has also opened opportunities for closer collaboration with international services departments in our governing institutions.

Conclusions: Hosting a conversation group in the library provides international

patrons with a relaxed and supportive environment for improving their English language skills. It also reveals ways to better support the information needs of this population and improves their perception of library services.

Crafting a Mosaic: Partnerships Between Health Sciences Libraries, Postdocs, and More

Karen Gau, AHIP
Research and Education Librarian, Tompkins-McCaw Library for the Health Sciences, Richmond, Virginia

Pamela M. Dillon
Research Liaison, Center for Clinical and Translational Research, Richmond, Virginia

Teraya Donaldson
Assistant Director of Educational Programs, Center for Clinical and Translational Research, Richmond, Virginia

Carrie L. Iwema, AHIP
Information Specialist in Molecular Biology, University of Pittsburgh, Pittsburgh, Pennsylvania

Objective: To describe the benefits of a How-to Talks by Postdocs series that was launched at two health sciences libraries to support their academic research communities.

Methods: Libraries at two public research universities implemented a recurrent workshop series, How-to Talks by Postdocs, for which postdocs were invited to submit talk proposals and present on “how to do” something related to the health sciences. Past talks include “How to detect and isolate stem cells,” “How to use 3D printing for tissue engineering,” and “Ethical considerations for scientific image manipulation.” Through this series, libraries are able to offer their health sciences campuses specialized education on important tools, and a space for researchers to engage in interdisciplinary discussion and networking. Additionally, postdocs are able to add formal teaching experience to their CVs, an opportunity that is not often available due to time primarily spent on lab work. This talk will focus on the benefits of this program for postdocs, other researchers, and the library.

Microaggression in Medical Libraries RESEARCH

Lynn Kysh
Clinical & Research Librarian, Children's Hospital Los Angeles, Los Angeles, California

Nancy Olmos
Head, Metadata and Content Management, Norris Medical Library, Los Angeles, California

Annie M. Thompson
Director, Wilson Dental Library, Los Angeles, California

Rebecca O. Davis
Information Services Librarian, Wilson Dental Library, University of Southern California/ Jennifer Ann Wilson Dental Library, Los Angeles, California

The amount of academic literature published on racial microaggressions has increased drastically since the early 2000s. However, the majority of the literature published is in the psychology field. There is a gap in the current library literature regarding racial microaggressions in the medical library community. A recent study in the Journal of Academic Librarianship measures racial microaggression experiences in the academic library setting, however, the study does not include medical librarians as part of the population. The speakers will share information about what microaggressions are, the group's research process, and their work towards developing a good tool to measure racial microaggressions among medical librarians and library staff from colleagues and the library's community of users. The speakers will also share their future plans for the project which include an outreach plan for the tool and a call for participation in the study.

On the Right Foot: Development of an Onboarding Program for New Staff

Jeff D. Williams, AHIP
Associate Director, NYU Health Sciences Library, New York, New York

Aileen M. McCrillis, AHIP
Head, Information Services and User Experience, NYU Health Sciences Library, New York, New York

Objectives: To promote rapid, thorough, and successful training and acculturation of new staff, the NYU Health Sciences Library developed a required onboarding program. New staff follow a customized, multi-month program designed to help them quickly become comfortable and productive in their roles. Responsibility for success is shared between the new employee, supervisor, team and/or unit colleagues, and the entire library staff.

Methods: Supervisors develop the onboarding plan in advance of the new employee's arrival. The plan sequences training and orientation, as well as promotes social interaction with colleagues and development of overall familiarity with the Library. As the staff member becomes comfortable in the new environment, the plan's focus moves to new staff meeting with units across the Library to understand their diverse responsibilities. They then meet with the director to learn about the overall medical center, and how the Library fits within the organization. Formal training for the new staff member runs in parallel with the onboarding process, with emphasis placed on learning tasks and responsibilities through exposure and mentoring from colleagues. The supervisor is responsible for ensuring the new staff member maintains progress on the plan, and supports their success through collaborative refinement of the program.

Results: As of early 2016, five staff have progressed through this onboarding program. In each case the supervisor has worked closely with the new staff member to adjust the program's pace and content based on individual needs and preferences. Feedback was solicited from staff that underwent the onboarding program to determine elements perceived as most useful and gather suggestions for improvement.

Conclusions: From the perspective of the supervisors, this required onboarding program has helped new staff become comfortable and productive in their roles more quickly than former ad hoc methods. The emphasis on balancing social interactions with training and orientations has diminished the typical stress of a new position, and this allowed new staff to absorb and retain more from their training. Another benefit is supervisors have objective measures for progress and development, and the process of working with new staff to adjust the plan helps build trust and comfort in the supervisor/supervisee relationship.

Mentoring: Making a Difference

Tami A. Hartzell
Senior Librarian, Werner Medical Library, Rochester, New York

Michelle L. Burda
Network and Advocacy Coordinator, Health Sciences Library System, University of Pittsburgh, Pittsburgh, Pennsylvania

Objectives: The Hospital Library Special Advisory Group of NN/LM MAR wanted to provide a mentoring program for hospital librarians within the Middle Atlantic Region. This presentation will highlight the development process for the program which includes:
• Identifying and recruiting mentors/mentees

• Thoughtful matching of mentors with mentees
• Establishing roles, expectations and goals of the participants
• Project evaluation

Methods: A variety of methods were employed to develop the mentoring program. A call went out to National Network of Libraries of Medicine, Middle Atlantic Region (NN/LM MAR) members to recruit both mentors and individuals seeking the experience and expertise of a mentor. Once mentors and mentees were identified, surveyed and matched, an agreement was drafted and signed by participants. Each team met virtually to identify goals and to establish an action plan that addressed the mentee's goals. The goals and expectations for both the mentor and mentee were shared among the group. Monthly progress reports provided by each pair helped to monitor the progress of each team with a final post-expectation survey to be distributed at the end of the first year.

Results: My results are not complete yet. If selected, I will come back into the system and enter my results after February 1, 2016 at 6:00 pm

Conclusions: My results are not complete yet. If selected, I will come back into the system and enter my results after February 1, 2016 at 6:00 pm

Student Awareness of the Big Picture: Teaching and Promoting Skills for Identifying Funding Opportunities

Judith Smith
Informationist, Taubman Health Sciences Library, Ann Arbor, Michigan

Kate Saylor
Health Sciences Informationist, Taubman Health Sciences Library, Ann Arbor, Michigan

Objectives: To introduce Public Health students to resources and information-seeking techniques for finding funding for summer internships through a class session and research guide. The goals were to:1) help students with their immediate funding needs; 2) prepare students as future researchers; 3) partner closely with the School of Public Health's internship coordinators

Methods: Internship coordinators at the University of Michigan's School of Public Health (SPH) reached out to health sciences informationists at the Taubman Health sciences Library for assistance with their students' funding needs. A SPH survey of students indicated that many were having trouble finding funding for their summer internships. The informationists took a multi-pronged approach to meeting these needs. The informationists began by teaching a grant-seeking workshop to raise

resource awareness and teach specific techniques for searching funding databases and taking advantage of university services. The informationists also revamped a student funding guide to clearly direct students to resources and strategies covered in the workshop. Finally, to reinforce learning and meet students at their point of need, informationists offered a “Library on the Road” office hour at SPH focused on funding.

ResultsAnecdotal evidence from internship coordinators indicated that a strengthened partnership between the coordinators at SPH and the informationists has vastly increased visibility of these services. Coordinators actively promote funding databases such as COS PIVOT, the tailored office hours, and refer students to the guide several times a week.

Conclusions: While the heightened awareness of the funding consultation services is a great first step in working with students on their funding needs, more information about the impact of the guide and associated instruction and consultations is needed. Informationists were encouraged by the anecdotal feedback from SPH, but must continue to partner closely with SPH internship coordinators to understand whether students secured funding as a direct result of what they learned from informationists. Informationists are also interested in better assessing student perceptions of how the skills learned will help them with future funding and research needs.

Writing Together: An Interprofessional Boot Camp to Support Scholarly Writing

Megan von Isenburg, AHIP
Associate Director, Research and Education, Duke Medical Center Library, Durham, North Carolina

Objectives: Writing for publication is an integral skill for both sharing research findings and career advancement, yet many faculty lack expertise, support, and time to author scholarly publications. Health professions educators identified writing as an area in which a new educators' academy could offer support.

Methods: To address this need, a writing task force consisting of a School of Medicine faculty member, a School of Nursing faculty member, and a librarian was formed. The task force launched an initiative to motivate and support faculty writing and publication by offering a "jump start" panel followed by a structured Boot Camp designed to motivate writing through a sequenced, modularized approach to manuscript completion. Nineteen faculty and one student enlisted in the Boot Camp. The task force members broke the participants into small groups based on topic and served as both guide and cheerleader to their groups. The Boot Camp schedule included regular deadlines and small group meetings over a four-month period. Participants received feedback from both peers in their group and from their group guide.

Results: Nine of the original 20 Boot Camp members completed a manuscript for submission by the end of the Boot Camp. Qualitative feedback was collected on the experience, and new plans are under development to address them, including creating an online community and "shut up and write" space, and incentivizing participation by offering professional editorial assistance to those that participate.

Librarian Involvement at Biomedical Informatics Journal Club: A Case Study

Darell Schmick
Research Librarian, Spencer S. Eccles Health Sciences Library, Salt Lake City, Utah

Melissa L. Rethlefsen, AHIP
Deputy Director / Associate Librarian, Spencer S Eccles Health Sciences Library, Salt Lake City, Utah

Objectives: To describe librarian involvement with a unique problem-oriented, credit-based journal club for biomedical informatics graduate students.

Methods: In Spring of 2015, the Department of Biomedical Informatics hosted a unique, problem-oriented journal club credit course for graduate students and interested faculty. The Chair approached the Library with the intention of creating a partnership that would enhance students' searching ability, while using journal literature to answer questions that faculty, clinicians, and staff were asking. The cohort met weekly and invited hospital and health system professionals to discuss challenges and request the cohort research and present findings on these questions. During class, the librarian shaped the topic into an answerable question and recommended key literature sources. Questions were assigned to students to research and meet individually with the librarian to develop a literature search on their topic. All students then shared in reading and reporting the selected key articles from the librarian consultation, which were shared at the following meeting.

Results: The librarians met with one student per week, whichever student had the literature review assignment for that week. Consults occurred every week of the semester and provided learning opportunities for the students as they learned how to locate quality literature of relevance to the topic discussed. Inquisitors were diverse, ranging from faculty within the department to hospital executives, and were impressed with the level of information provided by the student teams in each presentation. Collected student remarks were positive, and credited librarians for allowing them to find "valuable information [that] helps a lot" in the literature search process.

Conclusions: The journal club partnership was a successful way for librarians to develop individual time with graduate biomedical informatics students with research and literature searching obligations and improve on their previous search abilities. The

class was executed in a way that would be easily replicable for subsequent semesters in which the biomedical informatics journal club class will be offered.

A Part-Time Business Informationist on the Staff of an Academic Medical Library: Successes and Near-Successes

Alan Zuckerman
Librarian III/Academic Liaison Librarian for Business, Sheridan Libraries/Reference Services, Havre De Grace, Maryland

Objectives:
• Improve quality of medical library informationists' work on behalf of patrons when business/management aspects are involved
• Increase informationists' facility with business-oriented library resources
• Shoulder share of the workload faced by medical library informationists
• Improve business librarian's medical/science information literacy skills to better serve business constituencies
• Serve as go-between to Increase communication, mutual awareness, and coordination among librarians across the university
• Contribute to Johns Hopkins University's "One University" strategic goal

Methods:
• Direct report to Basic Sciences and Public Health informationist supervisor
• Participate in medical library informationist meetings, visits from and to outside entities, celebratory events
• Volunteer to participate in various informationist initiatives
• Proactively seek and develop relationships with targeted medical campus faculty and students
• Develop and present classes, orientations, and information sessions to medical campus groups as part of informationists' outreach activities
• Participate in classes as part of informationist team

Results:
• Improved business informationist academic research skills, including facility with Systematic Reviews
• Improved business informationist knowledge of the medical campus faculty, student, and staff populations—their capabilities, needs, expectations, and work flows
• Improved business research capabilities of medical, basic science, and public health informationists
• Improved reference assistance for some medical campus patrons
• Better information literacy instruction and reference services to business-oriented programs and classes on the medical campus, such as the Masters of Health

Administration program, various strategic planning courses, and several medical economics courses
• Higher level of cross-university librarian awareness of activities and capabilities of colleagues
• Better quality research support by the business informationist for business school faculty researchers coordinating projects with medical campus faculty
• Some difficulty stretching business librarian time and attention between business program and medical campus constituencies' needs

Conclusions:• By sharing the salary and time of the business librarian, the medical campus especially, and the main university campus to some degree, benefited in a number of ways, as outlined in the "Results" section.
• Johns Hopkins University's "One University" goal, focused on comprehensively knitting together the various campuses and programs of the university for the sake of greater synergies and achievement, was furthered by this initiative.
• This collaboration was made possible by a set of somewhat unusual circumstances:
• The business informationist has extensive health care management and consulting experience, which was beneficial for working with medical campus students and researchers.
• The two Johns Hopkins library ecosystems (the Welch Medical Library and the Sheridan Libraries) were comfortable sharing salary and FTEs.

Embedding an Informationist in Departmental Curriculum: Building Blocks for Successful Publication in a School of Public Health Environmental Health Sciences Program

Lori Rosman, AHIP
Public Health Informationist, Welch Medical Library, Baltimore, Maryland

Ellen Silbergeld
Professor, Department of Environmental Health Sciences, Baltimore, Maryland

Objectives: Successful publication in high quality peer reviewed journals is a skill that can take many years of trial and error to master. Curriculum was developed to give PhD students enrolled in a year-long Environmental Health Sciences writing seminar experience in advanced information search and management skills as one of the core foundations to developing successful publications and grant proposals.

Methods: First year PhD students in the Department of Environmental Health Sciences (EHS) participate in a four quarter, four credit writing seminar to develop critical skills for successful publication and grant proposals. The seminar consists of (1) Searching and Information Management, (2) Submitting for Publication, (3) Grant Writing, and (4) Hands-on Practicum. An informationist and an EHS faculty member

co-teach the first two quarters. Through problem-based learning and hands-on assignments, students learn to identify core resources; produce syntactically correct, reproducible search strategies in multiple databases; manage search results; apply best practices in data management; and identify the elements of successful scientific papers, including formats, data presentation, citations, and acknowledgements. Students' publications will be tracked over time. Interest in the course has grown over its three year history. An indirect result of the seminar has been increased informationist participation in departmental research.

Results: My results are not complete yet. If selected, I will come back into the system and enter my results after February 1, 2016 at 6:00 pm

Conclusions: My results are not complete yet. If selected, I will come back into the system and enter my results after February 1, 2016 at 6:00 pm

Embracing the Big Picture: Librarians Supporting Clinical InQuERI for Patient- and Family-Centered Care

Michelle Lieggi Henley
Clinical Research Librarian, UCSF Medical Center, Fishbon Library, San Francisco, California

Daphne Stannard
Director & Chief Nurse Researcher, Institute for Nursing Excellence (INEx), San Francisco, California

Objectives: In 2011, the Nursing Research and Innovation Council developed Clinical InQuERI, a model highlighting different approaches to problem solving in clinical practice. Quality improvement, evidence-based practice, research, and innovation activities are integral components, contributing to patient and family centered care. The author's objectives are to describe the model and to demonstrate how librarians are participating in these activities.

Methods: The authors searched PubMed, CINAHL and LISTA, for articles on librarian support for Clinical InQuERI activities within clinical practice. Articles from the last 10 years that noted quality improvement, evidence-based practice, research, or innovation, were selected for review. Articles primarily on medical or nursing students, graduate medical education, residency programs or continuing medical education were excluded. The authors also surveyed librarians on the MEDLIB-L listserv to understand other ways they support Clinical InQuERI. After examining the literature and survey results, the authors categorized the librarian activities and summarized how they contribute to Clinical InQuERI and patient and family centered care.

Results:

Results indicate that almost all librarians are involved in activities related to evidence-based practice (EBP). A significant number also support research, quality improvement or innovation. Of the 47 respondents to the survey, 98% support EBP, 83% support research, 80% support quality improvement, and 73% support innovation. Main EBP activities include literature searching for and teaching about EBP, followed by support for systematic reviews and guideline development. Main research activities include working with researcher profiling/collaboration tools and assisting with authorship issues, while quality improvement activities are mainly literature searching for quality improvement initiatives or participating in quality improvement committees. Although innovation is supported, librarians primarily provide literature searching on innovative practices. The literature from the last 10 years also indicates that librarians are heavily involved in EBP, but there is only marginal reporting on quality improvement, research, or innovation activities.

Conclusions: Based on the research, it is clear librarians are continuing to support EBP, and branching out to contribute to other activities in the Clinical InQuERI model. Research and quality improvement appear to be well supported by librarians in clinical settings, but there is room for more involvement in innovation. Literature searching in support of all these activities is still the most significant role for librarians in clinical settings. But involvement in quality improvement, research or innovation activities, although growing, is still fairly limited.

Zombies Ate My Evidence: The Evidence-Based Medicine Diagnostic Challenge to Save the World

Amy Blevins
Associate Director for Public Services, Ruth Lilly Medical Library, Indianapolis, Indiana

Elizabeth Kiscaden, AHIP
Head, Hardin Library Services, Hardin Library for the Health Sciences, Iowa City, Iowa

Jason Bengtson
Innovation Architect, Computing, Houston, Texas

Objectives: Medical/physician assistant students have been utilizing an evidence-based clinical practice (EBCP) curriculum for several years. Two of the librarians decided to create a proof of concept module on diagnostic principles using an interactive online zombie game. The ultimate goal of this project is to create a fun and engaging game that reinforces and assesses knowledge of EBCP concepts.

Methods: Two librarians hired an artist and application developer to help with the development of an EBCP zombie game using funds from an Innovation Award provided by local library system. They also enlisted the help of a clinical EBCP expert to critique the accuracy of the scenarios and EBCP activities. A storyline and two

activities based on diagnostic principles were created. The game takes place during a zombie epidemic, and the main character is a resident at an academic hospital who is tasked with selecting a screening tool and a diagnostic tool based on sensitivity, specificity, and likelihood ratios. The game will be presented to students in a pediatrics clerkship, and those students will be asked to fill out an anonymous survey with questions regarding the entertainment value, difficulty of activities, and overall effectiveness of the game as a learning tool.

Results: The game has been hosted on the University of Iowa's web server. Preliminary feedback from teaching faculty has been positive, and plans are underway to gather anonymous feedback from students on clinical rotations.

Conclusion: The game developers are pleased with results so far and are planning to create additional modules in the future.

Supporting Knowledge Synthesis Methods Training: Review of the Evidence for Online Systematic Review Instruction RESEARCH

Robin M. N Parker
Evidence Synthesis and Information Services Librarian, WK Kellogg Health Sciences Library, Halifax, Nova Scotia, Canada

Sarah M. Visintini
Evidence Synthesis Coordinator, Maritimes SPOR Support Unit and Nova Scotia Site of Cochrane Canada, Halifax, Nova Scotia, Canada

Leah M. N Boulos
MLIS Candidate and Student Intern, WK Kellogg Health Sciences Library, Halifax, Nova Scotia, Canada

Krista Ritchie
Assistant Professor, Educational Psychology, Faculty of Education, Halifax, Nova Scotia, Canada

Jill A. Hayden
Associate Professor, Dept. of Community Health & Epidemiology, Dalhousie University, Halifax, Nova Scotia, Canada

Objectives: Health librarians' support for systematic reviews includes directing reviewers to high-quality educational resources. Extensive research into online learning exists, but is it used to guide online delivery of systematic review training? In order to recommend high-quality training this project addresses the question: Are

online approaches for teaching systematic review methodology based on best practices for online instruction?

Methods: We conducted a comprehensive review of published educational and biomedical literature to identify reports of online instruction in systematic review methods. The paucity of academic literature precipitated an environmental scan to inventory existing web-based approaches. Online training resources were located using strategic web searches. We included instructional material for which we could access the course content freely online or by contacting the creators. After screening to ensure relevance to systematic review methods we extracted data on course characteristics. Using a detailed evaluation rubric developed by Foster et al. (2014) for online evidence-based practice instruction, two reviewers assessed the included courses and tools using the following criteria: 1) design; 2) format; 3) content; 4) degree and type of interactivity; 5) general usability. This evaluation revealed the extent to which the training resources followed accepted best practices in online instruction.

Results: We present the results of our evaluation of online courses, modules, and videos that provide instruction on some or all of the steps of conducting systematic reviews. Resources assessed varied in means of delivery, type of access (free or for-fee), and intended audience. The content was similarly diverse, with some courses or series of modules covering all steps of the systematic review process, while others, particularly video tutorials, frequently addressing only a portion of the stages of conducting a review and having minimal interactivity.

Conclusions: There is a range of resources available those learning how to conduct systematic reviews or other knowledge synthesis projects. The most appropriate training will depend on the needs and resources of the individual researcher: freely available videos and training modules may give a helpful overview of the process or a reminder of common challenges while online systematic review courses offered by research or academic institutions provide more in-depth and interactive coverage of each step and will help reviewers complete a systematic review in real-time, however they are frequently costly or require a more significant commitment of time.

Systematic Reviews: Evidence-Based Searching to Improve Recall and Precision RESEARCH

Susan A. Fowler
Medical Librarian, Washington University in St. Louis School of Medicine, St. Louis, Missouri

Lauren H. Yaeger
Medical Librarian, School of Medicine, Washington University in St. Louis, Saint Louis, Missouri

Angela C. Hardi
Clinical Resources Librarian, Bernard Becker Medical Library, Saint Louis, Missouri

Laura E. Simon
Clinical Librarian, Bernard Becker Medical Library / Health Information Services, Fenton, Missouri

Michelle Doering
Clinical Librarian, Bernard Becker Medical Library, St. Louis, Missouri

Objectives: Systematic review research often involves combing through many irrelevant citations. We propose that using adjacency instead of phrase searching will increase the recall and precision of search strategies, thus reducing irrelevant citations. Our objective is to test this hypothesis by measuring the difference in precision and recall between a search using adjacencies and one using phrases.

Methods: Search results will be measured against all articles published in top medical journals within the past few years to show results. Top medical journals will be chosen based on impact factors reported in InCites Journal Citation Reports and overall subject applicability to our search topic. We will read each title and abstract in the pool and code each article as “on topic” or “off topic”. Two search strategies will be executed in Medline; one using phrase searching and one using adjacency searching. Search results will be measured for recall and precision using the likelihood ratios for positive and negative results. Results will provide evidence regarding the effectiveness of adjacency versus phrase searching. These findings may aid searchers with evidence and applicable methods to produce search results with high levels of recall and precision for their systematic reviewers.

Results: My results are not complete yet. If selected, I will come back into the system and enter my results after February 1, 2016 at 6:00 pm

Conclusions: My results are not complete yet. If selected, I will come back into the system and enter my results after February 1, 2016 at 6:00 pm

Locating Systematic Reviews Comprehensively and Efficiently RESEARCH

Robert W. Sandieson
Professor, Faculty of Education, London, Ontario, Canada

Rachel M. Sandieson
Senior Library Assistant, Science Allyn and Betty Taylor Library, London, Ontario, Canada

Objectives: Systematic reviews have proliferated and evolved recently to include many types of research methods. Difficulties result locating reviews due to indexing, terminology, methods or textual context. The present investigation tested a search filter development theory, known as Pearl Harvesting, in order to find all of the unique linguistic markers related to systematic reviews in order to create comprehensive searching.
Method: Two texts on systematic reviews, one quantitative and one qualitative, were analyzed for linguistic content pertinent to systematic reviews. Some terms were noted for the lack of precision, e.g., “review” so were disambiguated to create a specific rich text filter. The terms were validated as necessary to the filter by searching each term separately and subtracting citations produced by all the other terms to see if any remaining were unique and relevant.

Existing search strategies for systematic review use low precision polysemic terms, such as “review”. Precision is measured at the level of individual term not as used in real world searches that contain a number of search filters. Therefore, a further analysis was done to create the complete set of polysemic terms for systematic review as a second, equivalent filter. The question was how extensive was the loss of precision at the real world search level where population and intervention filters were included.
Results: There were 110 specific linguistic markers found for a specific, rich text filter. Another filter contained three polysemic terms, i.e., review*, synthesi*, systemat*, plus search*, and variations of meta-analysis. Studies that developed search strategies for systematic reviews vary considerably and do not contain all of these terms; either the specific or polysemic. The two filters here were equivalent in finding systematic reviews. An analysis of real world searches found a minimal loss of precision using the polysemic filter in some cases, but not in others if the number of citations found was extensive. Further, an analysis of a Cochrane review of reviews demonstrated that the filters developed here could locate its 78 primary systematic reviews.
Conclusions: The Pearl Harvesting Information Retrieval Theory was valuable in developing two equivalent search filters for systematic reviews. The polysemic filter has an ease of use. The rich text filter is more precise and of value if the search yields a high volume of citations. This study indicated that search precision is a relative phenomena depending on context.

Sunday, May 15, 2016, 3:00 PM – 4:25 PM
Room: 201F

Session: Hospital & Health Care System Libraries

Bibliotherapy in a Hospital Setting: Promoting Health and Well-Being to Health Care Providers

Natalia Tukhareli
Librarian, Health Sciences Library, Rouge Valley Health System, Oakville, Ontario, Canada

Objectives: Within a context of benefits of a healthy workplace, bibliotherapy is seen as an effective way of promoting health and wellness to hospital employees. The objective of this study is to present a detailed description of an innovative informational and recreational bibliotherapy-based service for healthcare providers developed and implemented by a medical library, in collaboration with the Occupational Health department.

Methods: Methodology included an extensive literature review to explore current applications of bibliotherapy that address the needs of diverse client groups in a wide variety of settings. The analysis of bibliotherapy-based schemes successfully implemented in medical and public libraries in North America and the UK helped to identify the ways bibliotherapy could be used to promote health and well-being to healthcare providers. A case study was included to describe the Rouge Valley Hospital library's experience in developing, implementing, and evaluating an innovative informational and recreational service for staff and affiliated physicians. The mechanics, benefits, and challenges of the program will be discussed. The program evaluation included an internal survey to the hospital employees and semi-structured interviews with staff members. Feedback from participants will be considered as valuable information in view of future topics that could be addressed through the program.

Results: The data collected through an internal survey and interviews with the hospital staff indicates a wide interest in the program from both clinical and non-clinical hospital groups. The participants have acknowledged the recreational value of literary materials (particularly poetry) included into thematic compilations of readings as well as highlighted the importance of the informational component of the program. They suggested specific topics to be addressed in the future. Overall, the evaluation results

show that the bibliotherapy program has provided a new venue to address wellness and healthy workplace topics within the organization.

Conclusions: The case study shows that bibliotherapy can fill a unique niche within the context of services and programs traditionally offered in a hospital setting to address healthy workplace. It is considered to be an effective, simple, and cost-effective way of addressing recreational and informational needs of healthcare providers, managing work-related stress, and promoting healthy lifestyle. Finally, the bibliotherapy service helps expand opportunities for collaborative projects and partnerships for the library as well as increase visibility of the library within the organization.

The Library's Role in Patient Portal Implementation in an Urban, Academic Safety Net Hospital

Stephen Kiyoi, AHIP
Library Director, UCSF at ZSFG, San Francisco, California

Courtney Lyles
Assistant Professor, General Internal Medicine, San Francisco, California

Shobha Sadasivaiah
Assistant Clinical Professor, Internal Medicine, San Francisco, California

Objectives: This project evaluates whether a library-run program can successfully engage underserved patients with patient portal systems. This work is significant because 1) Meaningful Use has accelerated patient portal uptake nationally 2) Limited literacy patients are less likely to use patient portals, and 3) Hospital and public libraries are a well situated, yet underutilized resource to lead patient portal engagement efforts.

Methods: The Library Director led an interdisciplinary hospital workgroup responsible for patient engagement planning, including: 1) development of patient education materials, 2) creation of patient portal test account, 3) development of data collection strategies and structure, 3) integration of enrollment activities into clinical workflows, and 4) calculations of necessary staffing levels.

After the planning and pilot period, the library assumed primary operational responsibility for patient engagement operations. The library's "User Services Coordinator" recruited, trained, and coordinated a group of "Volunteer Health Technology Patient Educators," who visited hospitalized patients to: 1) Provide individualized health and computer literacy training 2) Assist with patient portal account creation, and 3) Collect data to track implementation metrics. Metrics included: account creation status, patient age and gender, English reading ability, and

reasons for portal decline.

Results: Between January 2015 and August 2015, approximately 8,500 eligible patients were discharged. Of these, 1,676 patients (20%) were visited by a patient portal volunteer. Of the patients who were visited, 1213 (72%) declined, and 463 (28%) agreed to create an account. Primary reasons for patient portal decline included limited computer access and ability (26%), limited English language ability (19%), preference of in person healthcare communication (11%), lack of time (9%) and concerns about privacy or security (5%).

Volunteers did not approach 80% of eligible patients. Reasons include lack of evening and weekend coverage, clinical interruptions, and limited volunteer availability.

Conclusions: Patient Portal systems present a unique opportunity for hospital and public libraries to better integrate in healthcare systems to facilitate increased patient access to their personal health information.

However, challenges in volunteer recruitment and retention, staff training, and targeted curriculum development must still be addressed. The Library has received a 3 year G08 grant from the National Library of Medicine, which will facilitate the development, delivery, and evaluation of an online patient portal curriculum for low literacy learners, in partnership with the city's Public Library System.

Implementing an Evidence-Based Decision-Making Model in a Health Care System

Jim Bulger
Manager, LIbrary Services/ Allina Health, Minneapolis, Minnesota

Anna Kleckner
Evidence-Based Practice Consultant, Allina Health Library Services, Minneapolis, Minnesota

Pamela Barnard
Knowledge Consultant, Library Services, Minneapolis, Minnesota

Andrew Crow
Knowledge Consultant, Library Services, Minneapolis, Minnesota

Anita von Geldern
Knowledge Consultant, Library Services, Minneapolis, Minnesota

Objectives: Hospital libraries are an essential part of the "big picture" of evidence-based decision-making. This paper describes the creation and implementation by

library staff of an evidence-based decision-making process to be used in establishing clinical guidelines for a 13-hospital healthcare system.

Methods: The process of evidence-based decision-making (EBDM) is key to the creation of valid clinical guidelines. The need was identified within the Allina Health system for a consistent, rigorous process to search and appraise the evidence, formalize the clinical guideline, and put the guideline into practice. The objectives were to 1) create a reproducible and measurable decision-making model; and ultimately to 2) reduce clinical variation and improve the care provided to patients. This process was supported by a system-wide policy on guidelines and implementation of a checklist system for guideline peer review and approval. Library staff has been instrumental in creating and implementing the EBDM model, working with clinical service lines to produce literature reviews and facilitate the development and implementation of the clinical guidelines.

Results:
• Development and implementation of an evidence-based decision-making model for guideline creation
• In 2015 a total of 19 guidelines were completed and implemented following this model
• Development and approval of a system-wide policy to promote compliance with the model

Conclusions: Allina has created and adopted an effective, system-wide EBDM model that facilitates collaborative guideline creation, resulting in an overall increase in understanding of, and appreciation for, systematic approaches to EBDM.

Did You Remember to Wipe? An Exploration of the Attitudes of Clinical Staff and Patients Toward Library-Supported, Shared Mobile Devices in Hospitals

Katie D. McLean, AHIP
Librarian Educator BA, LIT, MLIS, Nova Scotia Health Authority, Halifax, Nova Scotia, Canada

Lara Killian, AHIP
Librarian Educator, Patient Pamphlets, MA, MLIS, Nova Scotia Health Authority, Dartmouth, Nova Scotia, Canada

Katie Quinn
Library Technician, Hospital Library Services, Halifax, Nova Scotia, Canada

Objectives: The library's role in coordination, setup and maintenance of mobile devices is on the rise. In some situations mobile devices are set up for shared use by staff and patients. What are the privacy and usage implications of staff and patients sharing access to mobile devices? How should these devices be maintained, from both infection prevention and personal privacy perspectives?

Methods: Library staff coordinated with clinical or administrative team leads on two shared-access projects to develop iPad use procedures and policies, including the initial setup of iPads with selected apps and the ability to sync and manage content. Two surveys were developed to explore staff and patient attitudes towards sharing mobile devices and privacy. A scan of the literature on mobile device use and attitudes was carried out to inform survey questions. The survey was distributed on paper with the assistance of clinical staff.
Results: Results will be presented from surveys administered in two clinical locations: Nova Scotia Rehabilitation Centre (Musculoskeletal & Physical Medicine) and Nova Scotia Hospital (Mental Health & Addictions services). Authors will discuss the major content types that both patients and care-providers are seeking and accessing from shared iPads. Issues emerging from questions focused on privacy and hygiene, including appropriate use, will be discussed.

Conclusions: Overall patients and clinical staff have positive associations with the shared iPads and have made useful suggestions for content additions and usage guidelines. Grey areas remain present around issues of privacy, with training staff to maintain "digitally clean" devices being a continuous concern. To further investigate the physical cleanliness additional research is needed.

Merger Mosaic: The Impact of Mergers Among Health Care Institutions on Their Medical Libraries: Best Case: Multiple Libraries Meld into Single Mosaic

Susan M. Robishaw, AHIP
Director, Health Sciences Libraries, Danville, Pennsylvania

Patricia Ulmer, AHIP
E-Systems Management Librarian, Health Sciences Libraries, Danville, Pennsylvania

Mary Jo Devereaux
Health Sciences Librarian, Health Sciences Libraries, Scranton, Pennsylvania

Edith Asbury, AHIP
Health Sciences Librarian, Holy Spirit Hospital-A Geisinger Affiliate, Camp Hill, Pennsylvania

Objectives: To share best practices and processes for integrating the resources and services of libraries affected by health care system mergers. The perspectives of both the merging and merged libraries will be presented.

Methods: Health care institutions continue to merge. Mergers have an impact on the institutions' libraries as well as on institutions where no library exists. Worst case: Some libraries may be closed; displacing library employees and dispersing collections. Best case: Some libraries come together to consolidate their resources, services, and collections, forging an integrated mosaic that is more robust and stronger than the individual pieces. In doing so, they must overcome logistical, operational, and philosophical barriers, while managing organizational, financial, and cultural changes. Concurrently, they must continue to serve existing customers, expand services and products, and plan for the future. In this paper, the authors describe and document the planning, practices, and processes of mosaic building they developed through practical experience. Seven institutions with six libraries have merged since 2007. Another merger is well underway for fall 2015, with more to follow.

Results : The successful outcome of merged institutional libraries is an enhanced network of library services and resources, beneficial to all parties. ;">Achieving a successful outcome requires hard work, negotiating skills, and open and effective communication. This complicated and time consuming venture can be made easier by utilizing a checklist for pre-merger planning (primary library) and post-merger integration (both libraries). Networked library services and resources are extended to institutions in the system that don't have libraries. Education and training for all potential users is paramount.

Sunday, May 15, 2016, 3:00 PM – 4:25 PM
Room: 205B

Session: Systematic Review Services

Moderator: Rachel Pinotti

Evaluation of Systematic Review Knowledge and Training Needs: Supporting Systematic Review Research Capacity Development RESEARCH

Catherine Boden
Liaison Librarian, Leslie and Irene Dube Health Sciences Library, Saskatoon, Saskatchewan, Canada

Laurie Hellsten
Associate Professor, College of Education, Saskatoon, Saskatchewan, Canada

Objectives: Infrastructure and support to develop systematic review research capacity is available. However, few studies have explored researchers' training needs. The aim of this study is to examine the learning experiences, barriers and facilitators, and preferred mechanisms for teaching librarians and subject experts to conduct systematic reviews. This data will contribute to improved researcher development.

Methods: Experts in conducting systematic reviews will be identified purposively and through snowball sampling and electronically invited to participate in a series of focus groups. Experts will have, at minimum, 5 years of systematic review experience resulting in at least 2 published systematic reviews. Digitally-recorded, semi-structured 60-minute online synchronous focus groups (supported through conferencing software) will be conducted. Each group will consist of 5-8 individuals and will be held separately with subject experts and librarians. Resulting data will be transcribed and analyzed using thematic analysis following the recommendations of Braun and Clark (2006). Data will be organized and analyzed using NVivo software (QSR International). An inductive approach to theme identification will be employed at a semantic level within a realist paradigm.

Results: Five librarians and 7 researchers in non-librarian positions participated in the focus groups from across Canada (British Columbia, Saskatchewan, Ontario). Participants represented a spectrum of workplace contexts, levels of experience and roles in systematic reviews. Initial analysis reveals divergent and convergent themes for librarians and researchers. Further thematic analysis is

underway.

Conclusions: This data will contribute to improved researcher development for researchers and librarians interested in using systematic review methodology. It will inform our understanding of librarian and researcher barriers to acquiring systematic review expertise, and identify training mechanisms and supports that will enable librarians and researchers to transition successfully from receiving training to conducting systematic reviews.

The Value of Scoping Reviews: Mapping the Literature to Inform Clinical Practice and Future Research RESEARCH

Sarah M. Visintini
Evidence Synthesis Coordinator, Maritimes SPOR Support Unit and Nova Scotia Site of Cochrane Canada, Halifax, Nova Scotia, Canada

Andrea Smith
Project Coordinator, Department of Community Health and Epidemiology, Halifax, Nova Scotia, Canada

Jordan Edwards
MSc Candidate, Research Assistant, Community Health and Epidemiology, Halifax, Nova Scotia, Canada

Jennifer L. Cartwright
Research Coordinator, Department of Community Health & Epidemiology, Dalhousie University, Halifax, Nova Scotia, Victoria, British Columbia, Canada

Rachel L. Ogilvie
Research Coordinator, Community Health & Epidemiology, Halifax, Nova Scotia, Canada

Jill A. Hayden
Associate Professor, Dept. of Community Health & Epidemiology, Dalhousie University, Halifax, Nova Scotia, Canada

Objectives: Scoping reviews are a relatively new evidence synthesis method. They have rapidly been gaining popularity, but may not always be used effectively. We discuss Arksey & O'Malley's (2005) framework for conducting scoping reviews and describe methods and results of an exemplar scoping review that incorporated all six steps to the benefit of clinical decision making and research planning.

Methods: We conducted a scoping review on the management of low back pain (LBP) in the emergency department (ED). Scoping review was identified as the most appropriate evidence synthesis method because little had been done to synthesize the field and it facilitated mapping the literature on the topic. We searched bibliographic databases and grey literature trial registries. Studies meeting inclusion criteria were grouped according to pre-established categories related to patient flow and decision-making. Drawing on evidence mapping and Cochrane prioritization methodologies, we built preliminary evidence maps describing the depth of the research for each subcategory. We will compare our team's assessment of the research to that of our Low Back Pain Advisory Group consisting of ED healthcare providers, administrators, and patients. Through engagement of the Advisory Group we intend to validate our evidence map and identify opportunities for future locally relevant research.

Results: Our search identified 1746 results after duplicates were eliminated. Title/abstract and full-text screening excluded all but 117 studies. Studies were categorized by patient flow in the ED – presentation, diagnosis, treatment, and prognosis – and further subcategorized based on clusters of research objectives in the included studies. Next, we generated a question that summarized the types of research evidence that each category contained. Advisory Group members will be asked to rate how comfortable they are in answering each of the questions, based on their current education, training, and personal health experience. Their responses will be compared against our evaluation of how well developed the research field was for each subcategory. We will engage our Advisory Group to discuss and compare our assessment to theirs, and to identify opportunities for future primary and synthesis research

Conclusions: Incorporating evidence mapping techniques enabled us to synthesize the literature in a clear and interpretable way and helped shape and focus discussion with our Advisory Group. Stakeholder consultation is expected to add value to our scoping review by externally validating and interpreting our results as they are relevant to our local context.

Systematic Reviews: The Evolution of a New Library Service

Kate Krause, AHIP
Research Specialist, UT MD Anderson Cancer Center Research Medical Library, Houston, Texas

Beatriz G. Varman, AHIP
Manager, Client Relationship Management Deparment, Houston, Texas

Marianne Galati
Liaison Librarian, Client Relationship Management Department, Houston, Texas

Nha Huynh
Liaison Librarian, Client Relationship Management Department, Houston, Texas

Objectives: Two years ago our library began offering a free systematic review service. Since then, we realized that many of our researchers don't fully understand what systematic reviews are and underestimate the amount of work and time they take. Many of the systematic reviews we worked on were never finished or published by the researchers. Our current goal is to create policies to increase the number of completed and published systematic reviews with librarians listed as authors.

Methods: We implemented new guidelines to streamline our service and our ability to handle the high number of requests. We developed more stringent policies and are more discerning about whom we provide the service to. We require more face-to-face meetings with researchers and spend more time in our initial meetings to make sure they understand what a systematic review is, the amount of work and time they take, and what exactly they can expect from us – and what we expect from them. We implemented a two-tiered system offering different levels of service. For full service, we require researchers to sign a Memorandum of Understanding and include librarians as authors. We teach monthly classes on systematic reviews to give researchers, faculty, and students an overview of the process.

Results: We are still developing new work flows and policies but are well on our way to creating a service that works better for both researchers and librarians. Systematic Reviews take a long time to be published, so we are waiting to see if our next few years' statistics show an increased number of published systematic reviews.

Conclusions: Systematic Reviews require a great deal of librarians' time to complete. Educating researchers on the process and requiring them to sign a Memorandum of Understanding saves us from investing time and effort in systematic reviews that will never be published.

Blinded Ambition: Misperceptions and Misconceptions About Systematic Reviews from Teachers to Learners

Robert E. Johnson
Clinical Services Librarian, Norris Medical Library, Los Angeles, California

Lynn Kysh
Clinical & Research Librarian, Children's Hospital Los Angeles, Los Angeles, California

Objectives: A team of four librarians share the process of formalizing a systematic review service for an academic health science community and survey user perceptions about the service. A case series format highlights the successes and struggles of the process to educate and support systematic review research efforts.

Methods: The process includes a preliminary request form, an in-person worksheet establishing a working agreement between librarians and researchers, and a follow-up survey examining researcher perceptions. Formalizing a systematic review service exposed librarians to deficiencies needing attention; describing the differences between systematic reviews and literature reviews, working with teaching faculty to set realistic research goals within their curriculum, generating a comprehensive search for researchers, establishing a fee-based service model to access Embase, and effectively communicating with researchers. A survey sent to researches highlighted misconceptions about the systematic review process from the user perspective.

Results: My results are not complete yet. If selected, I will come back into the system and enter my results after February 1, 2016 at 6:00 pm

Conclusions: My results are not complete yet. If selected, I will come back into the system and enter my results after February 1, 2016 at 6:00 pm

The Librarian's Participatory Role in Rapid Reviews as Compared to Systematic Reviews: A Pilot Project

Maylene (Kefeng) Qiu
Systematic Review Coodinator and Evidence-Based Healthcare and Clinical Liaison Librarian, Biomedical Library, University of Pennsylvania, Philadelphia, Pennsylvania

Sherry Morgan
School of Nursing and Graduate Study Librarian, Biomedical Library, Philadelphia, Pennsylvania

Barbara Bernoff Cavanaugh
Associate Director of Health Sciences Libraries and Director of Biomedical Library, Health Sciences Libraries, Philadelphia, Pennsylvania

Objectives: This paper will describe a pilot project in which librarians were invited to participate in generating Rapid Reviews (RRs) with analysts at a center for evidence-based practice. Comparison and contrast of the librarian’s role in the RRs process with that of Systematic Reviews (SRs) will be presented.

Methods: Biomedical Library has a longtime partnership with a center for evidence-based practice which supports healthcare quality and safety through the summarizing of scientific evidence for decision-making. While SRs are the gold standard in

knowledge synthesis, they are considerably time-consuming and often focus on narrow clinical questions. Decision-makers often require access to contextualized resources that succinctly summarize a broader scope of scientific evidence quickly. The RR is an emerging methodology to addresses this need. The center’s analysts are facing an increase in RR requests with shorter turn-around time. To address this problem, librarians were invited to work with the center’s analysts in a new way, using RR guidelines to conduct literature searches, data screening, and the generation of annotated bibliography reports, which would then be either distributed to decision-makers or serve as an intermediary product for the analysts to further develop.

Results/Conclusions:

A collaborative project with CEP was conducted on the topic of Geriatric Consultations in Acute Care Hospitals. In contrast to working on SRs projects where librarians were required to search a broad range of resources to reach maximized sensitivity in retrieval within a lengthy timeframe, in the RR project, on the other hand, the librarians searched on targeted resources, retrieved results with the best balance between sensitivity and specificity, performed data screening and selection, and generated a report in a short time turnaround. The project yielded a positive result. Suggestions for the librarians who intend to participate in RRs were provided.

Sunday, May 15, 2016, 3:00 PM – 4:25 PM
Room: 202B

Session: Open & Public Access

Can They Find the Evidence? Accessibility of the Veterinary Medical Research to Unaffiliated Practitioners RESEARCH

Jessica R. Page, AHIP
Head, Hodesson Veterinary Medicine Library, Ohio State University Libraries, Columbus, Ohio

Objectives: This study aims to determine: (1) The open access (OA) and author's rights retention policies of the core veterinary journals; (2) What percentage of articles published by researchers at US colleges of veterinary medicine from 2000-2014 are freely available online to unaffiliated practitioners. Sources (e.g., institutional repositories, publisher websites) and trends in article availability over time will also be evaluated.

Methods: This study uses three existing sets of data. The titles on the core list of veterinary serials (http://www.ncbi.nlm.nih.gov/pubmed/20936066) will be cross-referenced with their entries in the Sherpa/RoMEO database to determine the OA and author archiving policies for this set of journals. The set of journal articles indexed in Web of Science that were published by researchers at 28 US colleges of veterinary medicine from 2000-2014 (N=54,391) will be cross-referenced with the corresponding journal or publisher data from Sherpa/RoMEO to determine the potential percentage of veterinary articles that could be made available to unaffiliated readers via open access or author archiving. A random sample of 5763 articles across 15 year-groups from the Web of Science dataset will be searched in Google Scholar from an off-campus location to determine availability to unaffiliated users and the source of available articles.

Results: Among core veterinary journals for which there is a title-level policy in Sherpa/RoMEO, 61% can be archived by the authors in their final manuscript form. The same percentage applies the full set of journals in which CVM researchers publish.
57% of articles published by CVM researchers are available online for free to unaffiliated users. 58% of those available articles are found on the publishers' websites. ResearchGate and PubMed Central are the next most common sources.The online availability of articles in this field increased steadily between 2000 and 2013. Fewer articles from 2014 are currently available online, possibly due to

publisher and archiving embargoes.
Conclusions: There are many articles freely available online, but opportunity exists to make more older articles freely available within the strictures of existing copyright agreements.
Articles in journals that narrowly cover veterinary medicine are less likely to be freely available online than those in auxillary fields. Larger publishers with broader coverage often make backfiles free at no cost.New requirements for federally funded research will likely lead to greater availability of articles, though much CVM research is not federally funded.

Using Process Mapping to Simplify a Complex Task

Merle Rosenzweig
Informationist, Taubman Health Sciences Library, Ann Arbor, Michigan

Nandita S. Mani, AHIP
Assistant Director, Academic & Clinical Engagement, Taubman Health Sciences Library, Ann Arbor, Michigan

Chase Masters
Enabling Technologies Informationist, Taubman Health Sciences Library, Ann Arbor, Michigan

Objectives: The National Institutes of Health (NIH) is the largest sponsor of researchers at our institution and the continuance of that funding is dependent on compliance with the NIH Public Access Policy (NIHPAP). Our objective was to investigate a solution to aid researchers in navigating the complex processes associated with the NIH Public Access Policy (NIHPAP).

Methods: Utilizing the Six Boxes™ model for performance improvement, we held conversations with key stakeholders to determine at what point and why there was confusion around the NIHPAP. Through our analysis, we determined that the best solution to addressing the complexity around the NIHPAP was to use process mapping. With a solution in mind, we broke down each component step, visualized the flow of information, tested the process map with key stakeholders, and developed process maps that could be provided in both multiple environments; online, printed handout, etc.

Results: We have shared the Process Map (PM) with various units at our institution involved in the NIH grant process including enabling and safeguarding the conduct of sponsored activities; the committee that provides oversight and information to staff who prepare and route NIH grant proposals; and the office which as the primary responsibility for research policy, oversight of administration and support of research by our faculty. These units have reported that the PM has helped in addressing the

NIHPAP compliance issues and they have incorporated it into their NIH grant processing and compliance workflow.

Conclusions: The development of the PM as a tool for simplifying the complex task of complying with the NIHPAP has established and highlighted a non-traditional role for librarians and can serve as a model for others. We are exploring the possibility of applying it to other complex tasks to assist our researchers.

Partnership in the Big Picture: Librarian Involvement in the Development and Publication of a New Interdisciplinary Open Access Journal

Elizabeth V. Weinfurter
Liaison Librarian, Bio-Medical Library, Minneapolis, Minnesota

Objectives: To describe the role of a health sciences librarian in the development and launch of a highly interdisciplinary open access journal. This paper offers a model for librarian involvement on interdisciplinary publishing teams, and provides practical examples and lessons related to publishing an open access journal on both a standalone website and through an institutional publishing platform.

Methods: In 2013, a nursing librarian was approached by a faculty member to discuss developing an open access journal to advance the scholarship and practice of the new interdisciplinary field of Partnership Studies. This paper follows the role of the librarian in a multi-institution publishing collaboration: from the conception and development of the journal, to hosting the first issue on a standalone website in 2014, to the migration to an institutional publishing platform in 2015, to the current ongoing publication of the journal. This paper outlines a mechanism for facilitating the free flow of knowledge to anyone across the world, which has immediate practical and ideological implications for health sciences librarians.

Results: The journal is in its third volume of publication, and lessons continue to be learned about sustainability and optimal publishing practice for an open-access journal staffed entirely by volunteers. The librarian continues to serve an important role in ensuring scholarly integrity of the publication, and in making connections between content and process experts.

Conclusions: Librarians are well-positioned to advise on and facilitate the development of open-access journals. Expertise and corresponding roles within the publishing team should be clearly defined to make the process run as smoothly as possible.

Using a Campus-Wide Open Access Policy to Increase Public Access Policy Compliance

Elizabeth Whipple, AHIP
Research Informationist, Ruth Lilly Medical Library, IU School of Medicine, Indianapolis, Indiana

Jere Odell
Scholarly Communications Librarian, University Library, Indianapolis, Indiana

Kacy Allgood, AHIP
Research and Community Engagement Librarian, Ruth LIlly Medical Library, Indianapolis, Indiana

Caitlin Pike, AHIP
Nursing and Medical Humanities Liaison Librarian, IUPUI University Library, Indianapolis, Indiana

Objectives: One Midwestern institution successfully passed a campus-wide Open Access (OA) Policy. The policy was adopted, in part, to enhance compliance with the NIH and other public access policies. Furthermore, library-led outreach to faculty about the OA policy provides another opportunity to assist with public access compliance. We seek to measure the impact of the OA policy on public access compliance.

Methods: A large portion of our campus scholarly output is the result of NIH-funded research, requiring many of our faculty to comply with the NIH Public Access Policy (PAP). Nevertheless, some authors continue to struggle with the compliance process. To see if the OA policy (OAP) increased public access compliance, we conducted a longitudinal analysis of NIH PAP compliance. After identifying a cohort of faculty articles published in journals that require author participation to complete the PA compliance process (NIH PAP Method D), we compared the NIH PAP compliance-rates prior to and following the adoption of the OAP. As a qualitative supplement to this study, we requested feedback from faculty that received library-led assistance with both the PAP and OAP. Responses will help to identify barriers to PAP compliance and may be incorporated into future implementations of the OAP service.

Results: My results are not complete yet. If selected, I will come back into the system and enter my results after February 1, 2016 at 6:00 pm

Conclusions: My results are not complete yet. If selected, I will come back into the system and enter my results after February 1, 2016 at 6:00 pm

It's the Law: Impact and Response to an Unfunded Open Access Act at a Small Public University RESEARCH

Paul M. Blobaum
Full Professor and Health and Human Services Librarian, Governors State University, Park Forest, Illinois

Objectives: This paper addresses the unanswered question: Do state mandates, especially without funding support, result in more access to faculty research output at public universities in Illinois? This paper will discuss the impact of the 2013 Open Access to Research Articles Act, which impacts the dissemination of faculty research at Illinois state universities during an era of intense competition for declining financial resources.

Methods: The Illinois Open Access to Research Articles Act of 2013 (Public Act 098-0295) mandates that public university boards appoint an Open Access Task Force to recommend and implement local and statewide policies to promote open access to research articles produced by their faculty by January 2015.

Results: At Governors State University (GSU), and other universities, Task Forces were appointed, Open Access policies were developed, and reports to the General Assembly were filed. The GSU Faculty Senate approved a policy that mandates Open Access rights to faculty work except under an “Opt Out” option, effective January 2015. The GSU Task Force recommended additional funding be appropriated specifically to support an institutional repository, or a consortial repository be adopted. A Scholarly Communications librarian was designated in March 2014, and Digital Commons, hosted by Bepress, Berkeley, California was implemented as the new institutional repository platform.

Conclusions: For the short term, the answer to the unanswered question is “no” at Governors State University. Implementation of open access policies have been hampered by larger funding and structural issues at our local University and statewide. At GSU, an Open Access committee has yet to be appointed and charge developed. The Faculty are largely unaware of the policy despite outreach efforts throughout 2015, but the Library is ready to work with Faculty on supporting Open Access articles and other materials in its digital repository, OPUS (Open Portal to University Scholarship) at opus.govst.edu . In fiscal year 2015-16, state Universities have been operating (through February 2016) with no state funding, resulting in threatened closures, furloughs, and workforce reductions for the short term. More research is needed to learn of the impact of Open Access on all state campuses in Illinois.

Sunday, May 15, 2016, 3:00 PM – 4:25 PM
Room: 104C

Session: Developing the Mosaic: Translating Education from Theory to Practice

Session: Developing the Mosaic: Translating Education from Theory to Practice

Mary E. Edwards, AHIP
Assistant University Librarian, Reference and Liaison Librarian, University of Florida Health Science Center Library, Biomedical Health and Information Services Department, Gainesville, Florida

Librarians, in support of the educational missions of their institutions, need to be efficient and effective in the design and delivery of instructional sessions. Utilizing an instructional design approach with sound pedagogical principles will bolsters a librarians' ability to create and deliver instruction that more effectively meets the needs of learners. However, translating instructional theories into practice can be challenging. The purpose of this program is to demonstrate how librarians can bridge the gap between instructional theory and practice. A panel of librarians will present instructional techniques they have found to be successful alongside an expert in human learning and instructional design that will unpack how and why the instruction was effective. The panel will conclude with a “stump the instructional designer” question and answer session.

Sunday, May 15, 2016, 3:00 PM – 4:25 PM
Room: 202D

Session: Beyond the Library Walls: Informationists and Pop-Ups

Moderator: Jennifer Lyon, AHIP

An Evolving Biomedical Research Informationist Program: Current Value, Future Development RESEARCH

Victoria G. Riese, AHIP
Chief, Education Services Branch, National Institutes of Health Library, Bethesda, Maryland

Kathleen B. Oliver
Program Manager, National Institutes of Health Library, Bethesda, Maryland

Keith W. Cogdill, AHIP
Director, National Institutes of Health Library, Bethesda, Maryland

Objectives: A biomedical library undertook an assessment of services, particularly its established Informationist program characterized by long-term, embedded collaboration or problem solving of limited duration. The study sought answers to the questions: 1) Why do investigators chose to work, or not to work, with the Library, and, specifically, Informationists; 2) What do they value; and, 3) Are there unmet information needs?

Methods: The study's methodology combined both qualitative and quantitative data collection from research leaders and investigators. It consisted of 30-minute interviews of scientific research and science policy leaders, and, a short, online questionnaire completed by principal investigators and research fellows at a major U.S. research institution. The target population was asked about library services, and more specifically, the expert information services in the context of their research.

Results: More than half the survey respondents had used the Informationist program for a year or more. Time is reported as the biggest obstacle to getting work-related information. Questions arise most frequently while working in their office. Answers are sought most frequently from their office. However, a significant number identified questions or sought answers during times of transition, while mobile, particularly when

in a clinical setting. Respondents value search expertise and training most. While leadership interviews indicate a lack of awareness of the program at higher levels, all end-users report strong levels of satisfaction, would use Informationist services again and would recommend them to colleagues. Productivity, quality, and speed are seen as key benefits of the program.

Conclusions: Among respondents, informationist services are highly used and valued, increasing productivity, satisfaction, and confidence. Results indicating some information seeking during times of transition have implications for developing and planning technical support for mobile applications. Results will be shared with NIH leadership to heighten awareness.

Assessing the National Library of Medicine's Informationist Awards RESEARCH

Ariel Deardorff
Assessment and Data Management Librarian, NLM Associate Fellow, UCSF Library, San Francisco, California

Objectives: To assess whether NLM-funded informationists enhanced NIH-funded biomedical research and evaluated their contributions to the research team, and to understand the overall experience of informationists and researchers who worked together.

Methods: Research methods consisted of a survey of principal investigators (PIs) on the funded research teams, and a focus group of informationist awardees. The survey, which was directed only at the PIs from the 2012 awards, focused on the impact of the informationist on the team's research, and specifically asked about the informationist's effect on the data management areas outlined in the funding announcement. The focus group, which was held at the 2015 MLA conference, invited informationist recipients of the 2012 and 2014 rounds of the award to share their contribution to biomedical research, state whether or not they were able to evaluate their role's value, and share their overall experience.

Results: Informationists had a positive impact on their team's research, especially in the areas of data storage, data management planning, data organization and literature searching. In addition, many informationists felt that their involvement had increased their research skills and made them more effective research partners. Assessment of the impact of the informationists on the research was a challenge for the award recipients and questions remain about the best evaluation methods. The overall experience of the informationists and researchers was mixed but largely positive.

Conclusions: The NLM-funded Informationist Supplement Awards were a successful

mechanism for immersing informationists into research teams and building data management skills in the projects supported.

Informationist Integration in Health Professions Education

Nancy J. Allee, AHIP
Deputy Director, Taubman Health Sciences Library, Ann Arbor, Michigan

Deborah Lauseng
Assistant Director, Academic & Clinical Engagement, Taubman Health Sciences Library, Ann Arbor, Michigan

Carol Shannon
Informationist, Taubman Health Sciences Library, Ann Arbor, Michigan

Barbara L. Shipman
Informationist, Taubman Health Sciences Library, Ann Arbor, Michigan

Zachary Wickline
Administrative Research Assistant, Taubman Health Sciences Library, Ann Arbor, Michigan

Objectives: Libraries are engaging as collaborative partners in many ways to promote learning. This project describes an innovative approach to help evaluate the integration of informationists into a new, competency-based master's in health professions education program. The process includes examining aspects relevant to the role of the informationist, information/communication flow between informationists and the learner community, and library skills and competencies.

Methods: In the new master's program, learners are paired with faculty mentors and work to complete 20 competencies, or earned professional activities. Informationists form part of the learning community, helping to facilitate the learners' educational experience. To evaluate informationist integration and to address the question: how can informationists better serve learners' information needs?, informationists partnered with a group of graduate information science and health informatics students. Working together, the informationists and graduate students designed an evaluation plan, utilizing a contextual inquiry method to conduct a series of interviews with program administrators, mentors, learners, and informationists. Interview data was analyzed to identify challenges for the learners in researching their written activity reports and to identify gaps in understanding of the ways in which informationists could contribute to the learning process. Findings from this project will benefit libraries engaged in collaborative learning initiatives.

Results: We found that there was confusion among learners and faculty about the role

of the informationist in the program and that there were communication gaps in reaching learners. There was a need for an improved research guide for faculty and learners to more easily navigate to library and informationist resources and services. Additionally, more comprehensive collaboration with the new degree program required expanding the number of informationists linked to learners.

Conclusions: Learners would benefit from connections to informationists at an earlier point in beginning their studies in the new competency-based program. The contextual inquiry method proved very useful in revealing information for rethinking and redesigning informationist services.

Effectiveness of New, Informationist-Led Curriculum Changes at the College of Pharmacy

Carol Shannon
Informationist, Taubman Health Sciences Library, Ann Arbor, Michigan

Objectives: To improve students' knowledge of locating evidence-based literature through revised integrated library instruction within the College of Pharmacy and to document the change in student knowledge using a formal assessment process across classes.

Methods: When my Pharmacy faculty partners and I reviewed student performance searching databases for evidence-based literature in January 2014, it was clear that the students did not have enough time to learn skills and, importantly, apply their new knowledge. To remedy the situation, we decided to pilot a new class: in February, we added an active learning session as an option in a new class. Based on that success, in May I proposed a new curriculum, with active and engaged learning experiences plus assessment throughout. Both the content and method of instruction were changed: 1) increasing the number of classes and deliberately scaffolding the sessions; 2) including clinical scenarios that paralleled their work in their therapeutics classes; and 3) using a flipped-classroom model, providing online instruction before class and using team-based problem solving and peer-to-peer teaching to reinforce learning in class.

Results: The pretest confirmed that students had at most a basic understanding of PubMed, and few had any knowledge of Embase or of indexing terms. After completing the pre-work for the first class, 70% of students demonstrated a knowledge of the basics of searching, and they retained this knowledge into the winter term. In the second class, which did not use any pre-work assignments, only about half of the students had a firm understanding of the class material.

Conclusions: These results clearly demonstrate that adding active learning components to classes increased students' skills in literature searching. The difference

in the acquisition of knowledge in the 2 classes also confirmed that pre-work is an essential component of the curriculum.

Pop-Ups!: Extending Consultation Services Beyond the Primary Library

Julia K. Kochi
Director, Collections, Education, and Research Services, Library and Center for Knowledge Management/University of California, San Francisco, San Francisco, California

Kemi Amin
Communications Manager, Library and Center for Knowledge Management/University of California, San Francisco, San Francisco, California

Ariel Deardorff
Assessment and Data Management Librarian, NLM Associate Fellow, UCSF Library, San Francisco, California

Anneliese S. Taylor
Assistant Director, Scholarly Communications & Collections, Library and Center for Knowledge Management/University of California, San Francisco, San Francisco, California

Evans Whitaker
Education and Information Consultant for Medicine, Library and Center for Knowledge Management/University of California, San Francisco, San Francisco, California

Objectives: The purpose of the program is to provide regular in-person, in-depth consultation services to students, faculty, and staff at the University's rapidly growing second campus and to increase the Library's presence at an otherwise underserved location.

Methods: In Fall 2014, an unstaffed Library space was opened at the University's second campus to augment a small, minimally staffed location. Pop-ups were originally conceived as a way to meet with librarians normally located on the main campus about specific topics and tools on a drop-in basis and were scheduled to occur once or twice a week for a 3-hour period. Topics included EndNote for collaboration, open access, and assistance with PubMed searches. The program has evolved throughout the past year, and the presentation will describe marketing strategies (both successful and not), lessons learned as well as challenges faced in extending service without increasing staff. The presentation will also feature the results of an evaluation based on program data and user feedback that will establish the impact of the program on perceived Library services at the second campus.

Results: My results are not complete yet. If selected, I will come back into the system and enter my results after February 1, 2016 at 6:00 pm

Conclusions: My results are not complete yet. If selected, I will come back into the system and enter my results after February 1, 2016 at 6:00 pm

Sunday, May 15, 2016, 3:00 PM – 4:25 PM
Room: 203B

Session: Research Support

Moderator: Rebecca Raworth

Librarian Use and Advocacy of Author Tools to Determine Research Impact RESEARCH

Tara Brigham
Medical Librarian, Winn-Dixie Foundation Medical Library, Jacksonville, Florida

Leah Osterhaus Trzasko
Librarian, Plummer Library / Public Services, Rochester, Minnesota

Ann Farrell
Librarian V, Plummer Library, Rochester, Minnesota

Melissa L. Rethlefsen, AHIP
Deputy Director / Associate Librarian, Spencer S Eccles Health Sciences Library, Salt Lake City, Utah

Objectives: To determine personal Librarian use of institutional repositories, citation tracking, citation alerts and author digital identifiers (eg. ORCID, ResearcherID, Google Scholar Citations profile).

Methods: Proactive authorship involves an individual exercising the right to post their work in an institutional repository and tracking their research impact. Using tools such as ORCID, ResearcherID, and Google Scholar Citations, help Librarians track their own research impact and familiarize them with the resources so they can more easily educate their patrons on using these tools. We conducted a web-based survey to investigate whether health science librarians are using tools for tracking authorship and research impact.

Results: My results are not complete yet. If selected, I will come back into the system and enter my results after February 1, 2016 at 6:00 pm

Conclusions: My results are not complete yet. If selected, I will come back into the system and enter my results after February 1, 2016 at 6:00 pm

Mining for Gems: Gathering Research Impact Metrics with a DIY Approach

Karen E. Gutzman
Impact and Evaluation Librarian, Galter Health Sciences Library, Chicago, Illinois

Pamela L. Shaw
Biosciences & Bioinformatics Librarian, Galter Health Sciences Library, Chicago, Illinois

Stacy Konkiel
Outreach & Engagement Manager, Altmetric, London, United Kingdom

Objective: Researchers are increasingly curious about the ways in which their scholarly outputs are represented in scholarly, social and mass media platforms. While many databases track citations and other scholarly metrics of impact (though their coverage differs), the options for tracking public consumption is often fragmented, and gaining a clear picture can be difficult.

Methods: In this study we compare and contrast the metrics available to researchers, and how librarians can help them utilize specific resources in tracking these metrics. We discuss the complexity of gathering and maintaining these metrics. Additionally, to provide a deeper understanding of these metrics, we describe the processes by which free and subscription-based resources capture data. Lastly, we comment on strengths and common uses of these metrics, and the gaps in coverage.

Results: We found the availability and quality of scholarly and alternative metrics varies depending on the resources available. Elsevier's Scopus, Thomson Reuter's Web of Science, and Google Scholar are major sources of scholarly metrics, while alternative metrics are available through Altmetric, PlumX, ImpactStory, and various scholarly social networking platforms. Each resource has different methods for aggregating data and making it available for librarians and researchers to use. Additionally, each metric has common uses and strengths that should be considered when determining metrics for specific contexts.

Conclusions: Scholarly and alternative metrics are increasingly being used as a proxy to measure scientific quality, collaboration, innovation and impact. Librarians can help researchers find these metrics, and understand their common uses, while creating an awareness of how this data is captured, and made available for use. A solid understanding of the strengths, challenges and availability helps librarians and researchers be better consumers and users of these metrics.

Medical Student Publications: A Forgotten Piece of the Scholarly Communications Mosaic

Patricia L. Thibodeau, AHIP, FMLA
Associate Dean for Library Services & Archives, Medical Center Library & Archives, Durham, North Carolina

Megan G. Van Noord
Research & Education Librarian, Duke Medical Center Library & Archives, Durham, North Carolina

Emma Heet
Associate Director Collection Services, Medical Center Library & Archives, Durham, North Carolina

Virginia M. Carden, AHIP
Administrative Research Librarian, Medical Center Library, Durham, North Carolina

Beverly Murphy, AHIP, FMLA
Assistant Director, Communications and Web Content Management; Hosptial Nursing Liaison, Medical Center Library & Archives, Durham, North Carolina

Objectives: Medical students often have the opportunity to be involved in research projects or experiences. Students want to generate a published manuscript to assist them in their future career, including applying for residency. However, this group is not always targeted as potential authors by the library. The question arose as to whether and where medical students publish.

Methods: The Library maintains a database of institutional publications containing unique institutional identifiers for all affiliated authors. By matching that database against a list of medical student identifiers we created a database of student publications over five years. The library was then able to study the database for average number of publications per year and per student, trends in the number of publications per year, subject areas, and journals selected for publication. The library will also explore citation rates, journal impact factors, and open access publications. This data will provide feedback to the school of medicine about its research experiences, but also inform library services and training for medical students in the areas of selecting publications, open access, and related scholarly communication issues.

Results/Conclusions: The rate of papers appears to be increasing from year to year with a large number of students having 2 or more 5 papers across their years in medical school. This may reflect the importance some fields are placing on publications for residency applications. While one might expect that students enrolled in the longer Medical Scientist Training Program (dual MD-PhD) would have the

highest number of citations this is not the case. Because students are publishing with their research mentors they are also able to have manuscripts accepted in major journal titles. Given the importance of publishing for career tracks and the high volume of papers, the Library will explore how students are currently seeking support in writing manuscripts and identify gaps that the Library can fill.

Assessing the Impact of Individualized Research Consultations on Students' Search Techniques and Confidence Levels RESEARCH

Lindsey Sikora
Acting Head, GSG and Social Sciences Library, University of Ottawa Library, Ottawa, Ontario, Canada

Karine Fournier
Head, Reference Services, Health Sciences Library, Ottawa, Ontario, Canada

Objectives: Studies dedicated to the assessment of individualized research consultations (IRCs) are scarce, with few using pre- and post-test methodologies. Our study's primary goal is to evaluate the impact of IRCs on health science students' search techniques and confidence levels, before and after meeting with a librarian. Secondary objectives include identifying factors influencing students' self-perceived search techniques' proficiency and confidence levels.

Methods: Our study's population included students completing an undergraduate or graduate degree, undertaking a research or thesis project. In order to assess the impact of IRCs on students' search techniques, a mixed methods approach was selected. Pre- and post-testing were used, as well as interviews. Participants were invited to complete two questionnaires, before and after meeting with a librarian. The questionnaires consisted of both open-ended and self-reflective questions. The open-ended questions assessed students' search techniques (keywords, subject headings, Boolean operators). The self-reflective questions were used to assess students' self-perceived search techniques proficiency, their confidence level, and lastly, their expectations (before) and their satisfaction (after) of the IRC. A rubric was used to score students' open-ended questions, and a statistical test was used to demonstrate the impact of the intervention. Self-reflective questions were coded and analysed for content.

Results:
With a small sample size (n=9) generated from our first round of data collection, we decided to conduct a second round of data collection (January to June 2016). Preliminary results from Round 1 indicated a slight increase in the mean score (comparing pre- and post-tests), demonstrating an improvement in the student's appropriate use of keywords and search string structure. Several of the students,

however, did not provide their keywords and search string; therefore, our results are extremely skewed.
Self-reflective questions indicated that students had mixed views on their confidence level before meeting with a librarian, especially regarding locating relevant sources. However, once they met with a librarian, and were able to have a better understanding of their question, their confidence levels improved.
Conclusions: We believe that we will be better able to quantify our results with our second round of data collection. That being said, while our results are preliminary, this is a start at quantifying individual research consultations' impact on students' search techniques, in order to better understand how to help students select appropriate keywords (and subject headings) and build a more accurate search string. Further research into disciplines beyond health sciences and medicine should be explored, as the resources to search are vastly different.

Medical Librarians in Practice: Research-Related Activities and Resource Use RESEARCH

Carol L. Perryman
Assistant Professor, Texas Woman's University, Denton, Texas

Ted Babin
Doctoral Student, Dept. of Sociology & Social Work, Denton, Texas

Objectives: 1. To better understand medical librarians' research-related activities (RRAs) and 2. to learn about resources consulted, including published and unpublished information.

Methods: An IRB approved, open- and closed-ended survey conducted on Psychdata.com was distributed through national and regional medical library electronic mailing lists, with questions including institutional affiliation, roles, types of support provided, RRAs over the last 2 years, and resources consulted. Cross-tabular analysis was used to identify significance and strength of associations between RRAs, resources, and institutional affiliations.

Results: After cleaning, 219 responses were analyzed. Respondents' affiliations were self-identified as academic (36%), hospital (43%), and 'other' (21%), a catchall that included government, public, corporate, and mixed (e.g., both academic and hospital). Most were employed full time (89%) were classified as staff (43%), and had worked more than 11 years in libraries (66.4%).

In descending order, librarians reported involvement over the past two years in RRAs identified as supporting library projects or project planning; planning for library (or other) programs (e.g. workshops, seminars); evaluating outcomes or effectiveness of library programs, collections, or services; measuring needs/surveying library

stakeholders or users; strategic planning for a library; and library association planning, projects, or research.

Descriptive statistics were generated to describe connections between institutional affiliation and RRAs, resulting in associations between affiliation and project planning, needs assessment, outcomes analysis, and program planning.

Across all affiliations and RRAs, the most frequently consulted resources were librarians' own experiences and information from peers, followed by peer reviewed literature, listservs and listserv archives, information found online, visits to other locations, and the use of consultants. Associations were found between institutional affiliation and the use of peer- and non-peer reviewed literature, personal experience, and peer consultation.

Conclusions: This study, part one of two, attempts to approach a more granular understanding of medical librarians' involvement in research by examining activities performed and resources used. Findings add to and expand upon prior research, helping to support education and practice in a rapidly-changing environment. Responses about resources used indicate clearly that librarians are not only using research literature, but also a wide variety of traditional and nontraditional resources to support their work.

Sunday, May 15, 2016, 3:00 PM – 4:25 PM
Room: 203D

Session: People-Centered Health Care: Consumer Health Librarians on the Front Line

Moderator: Stevo Roksandic, AHIP

People-Centered Health Care: Consumer Health Librarians on the Front Line

Michele Spatz
Project & Mobility Manager, Mid-Columbia Econimic Development District

Melinda Orebaugh
Director, Library & Health Information Services, Gundersen Lutheran Health System, LaCrosse, Wisconsin

Stevo Roksandic, AHIP
Regional Director, Mount Carmel Health System Library Services, Columbus, Ohio

The program will have a primary speaker for 30 minutes and two secondary speakers for 10 minutes each. Ten minutes will be allocated to a brief discussion between the speakers and audience will be invited to ask questions for 30 minutes following the discussion. Each speaker will give a conclusive summery at the end.

All three presenters will address major elements of consumer health information (CHI) resources and services, sharing insights about CHI fundamentals (i.e. literacy, diversity, ethics, cultural sensitivity).The primary speaker will share experiences, lessons learned and other insights with regards to providing CHI services for over thirty years in the healthcare field being consistently people-centered. The primary speaker will focus on sharing personal learning and practical experiences in planning, organizing and implementing CHI services. Addressing operational and organizational components (i.e. financials, staffing, technology, service model, partnerships, marketing), the primary speaker will also share some insights about the difference of engagement and meeting needs between two major groups: patients, as persons which are under medical care or treatment, and consumers as persons which acquire health information resources and informational services for their own wellbeing,

prevention and healthy-living.The secondary speakers will share their accomplishments, challenges, strategies and models of organizing and providing CHI services to patients and the community-at-large based on a People-Centered Healthcare approach. The primary focus will be on presenting their current business operational models, shared best practices, lessons learned and visions for future development of CHI resources and services.

Monday, May 16, 2016, 10:30 AM – 11:55 AM
Room: 201D

Session: Teaching Evidence-Based Practice: Methods and Outcomes

Moderator: Gregg A. Stevens, AHIP

Teaching Evidence-Based Medicine Using "Questions Patients Ask"

Abraham Wheeler, AHIP
Health Sciences Librarian, MSU Libraries, East Lansing, Michigan

Objectives: Does shifting the teaching of information literacy skills away from traditional library sessions and towards practical patient encounters increase students understanding and valuation of evidence based medicine? The librarian embedded these patient encounters called "Questions Patients Ask" longitudinally into a medical school curriculum. This new method has increased student engagement with and understanding of EBM and biostatistics.

Methods: In collaboration with medical school faculty, the librarian developed a series of patient encounter scenarios. These graded curriculum activities require medical students to first, understand the patients' actual concern, locate information about the concern, and finally interpret the information to make a clinical decision. These "Questions patients Ask" grow gradually more complex as the student progresses through the curriculum. They simulate a professional encounter where clinical knowledge, patient communication skills, EBM, practical biostatistics, and information literacy all merge. The sessions are taught jointly with the librarian providing the statistical and research interpretation expertise, and a clinician faculty providing the medical and practical expertise. This co-teaching method models the value of EBM in everyday medical practice. The liaison's efforts will be evaluated using student performance data, and tracking COMLEX scores. Expected outcomes are students with increased competence in EBM and biostatistics.

Results: Formal assessment has been difficult, with few students' responses to course surveys. This is not unique to "Questions Patients Ask", as response rate to surveys by students is low across all courses in the curriculum. Anecdotal reports from faculty are that they have noted an improvement with students' presentation, analysis and synthesis of research. Additionally, this new approach has led to greater

involvement of the librarian in all aspects of the 4 year curriculum.

Conclusions: Student engagement in EBM and biostatistics has been increased by placing them in a practical, professional context. Student surveys report better understanding of the topics after the sessions, as well as better understanding their value to a medical professional. Faculty report seeing a longitudinal increase across the curriculum in students use of evidence and comfort engaging with research methods and interpreting statistical results. Although early reports are promising, further study is needed to track the impact of this new approach to student COMLEX scores and other measures of student success.

Real-Time Education on Location: Developing a Modular Clinical Pediatrics Evidence-Based Practice Curriculum

Nicole Capdarest-Arest
Librarian, Stanford University School of Medicine / Lane Medical Library, Stanford, California

Alice A. Walz
MD/Clinical Fellow, Division of Pediatric Critical Care, Nashville, Tennessee

Caroline E. Rassbach
Clinical Assistant Professor, Division of Pediatric Hospital Medicine | Associate Program Director, Pediatric Residency Program | Pediatric Hospital Medicine Fellowship Co-Director, Pediatrics, Palo Alto, California

Context: In 2010, the Accreditation Council for Graduate Medical Education (ACGME) and the American Board of Pediatrics (ABP) introduced a new milestone-based method of program evaluation, the Pediatrics Milestone Project. The single milestone classified under medical knowledge is MK1: “Critically evaluate and apply current medical information and scientific evidence for patient care”. The 5 levels of competency delineated in MK1 align with advancing information literacy and evidence-based practice (“EBP”) skills from factual to metacognitive knowledge and simple (e.g., remember) to more challenging and self-directed (e.g., create and teach) cognitive processes. Generally, residents have identified poor searching skills and insufficient time as barriers to EBP. To facilitate learners’ achievement of competency in incorporating EBP and information literacy skills into clinical care, the Stanford pediatrics residency program collaborated with Lane Medical Library’s clinical librarian to develop a program to enhance these skills.

Purpose/Objective: This paper describes a program developed to aid learners in the inpatient clinical setting to more effectively and efficiently formulate answerable clinical questions and search for quality evidence using advanced search methods and tools.

Setting/Participants: An educational program was developed as a 4-part modular curriculum designed to be delivered weekly to pediatrics residents and clerkship rotation students in the resident workroom of the general inpatient pediatrics team in the Lucile Packard Children's Hospital.

Program Description: The clinical librarian in collaboration with the department of pediatrics developed a 4-part modular curriculum in searching for clinical evidence. Overall structure was guided by the MK1 Milestone. The 4 modules cover: 1) identifying knowledge gaps, structuring a clinical question, and tips for transferring a question into a search strategy; 2) understanding the evidence landscape (i.e., available resources and the hierarchy of evidence); 3) advanced search methods for time-pressed situations; and 4) mobile tools for pediatric clinical care and evaluation of mobile resources. Each module is delivered weekly during afternoon post-rounds workroom time so that real-life, real-time clinical questions can be used as examples for instruction. This brings immediate clinical relevance to improving EBP skills. After the 4-module cycle has been delivered, the curriculum starts again with the next rotating group of learners. Each module is independent of the others to accommodate staggered rotation schedules.

Results: Since the conception and implementation of this modular clinical EBP curriculum in July 2014, approximately 150 medical students and residents have experienced this program. The curricular modules are continuing to be refined, and strategies for further developing learners' skills in these areas in order to advance their clinical practices and progress on the MK1 competency are being examined. Common themes in aggregate MK1 Milestone progress achievement will help inform where learners excel and struggle in regard to this milestone and will help inform further program development.

Conclusion: The integration of clinical librarian teaching of clinical EBP skills into the regular workflow of an inpatient hospital care team can be an approach to helping this group of learners grow in competence on the pediatrics MK1 Milestone.

Assessing Medical Student Evidence-Based Searching Skills Through Direct Observation: Development and Validation of a Scoring Rubric RESEARCH

Joey Nicholson
Education and Curriculum Librarian, NYU Health Sciences Library, New York, New York

Dorice Vieira
Clinical Outreach and GME Librarian, NYU Health Sciences Library, New York, New York

Jeff D. Williams, AHIP
Associate Director, NYU Health Sciences Library, New York, New York

Objectives: Medical librarians have historically assessed medical students' effectiveness in literature searching using self-reporting or assessment of the end result of the process. A recent review of the literature reveals no instances of direct observation to evaluate this skill. This research project aims to find a reliable and scalable way to evaluate effectiveness of evidence-based searching using direct observation.

Methods: As part of AAMC's Entrustable Professional Activities (EPA) #7 skills pilot, three medical librarians developed a pilot literature searching scoring rubric assessing seven categories: question articulation, PICO formulation, database selection, search terms, search strategy, searching filtering, and article selection. They then tested the rubric through direct observation of 35 fourth-year medical students conducting literature searches as part of a simulation-based Objective Structured Clinical Examination (OSCE). Each student had seen a simulated patient and then had to use a computer to formulate and answer his or her most important clinical question related to this patient. Two librarians observed the students via screencasting and evaluated them using this rubric.

Inter-rater reliability was evaluated for each category within the rubric. Qualitative guided discussion among the rating librarians was used to refine and revise the rubric category scoring guidelines.

Moving from Searching to Synthesis

Abraham Wheeler, AHIP
Health Sciences Librarian, MSU Libraries, East Lansing, Michigan

Objectives: The librarian collaborated with faculty to develop and integrate a longitudinal evidence based medicine thread throughout the four year medical school curriculum. We shifted the focus from training searching skills and instead emphasized interpreting and synthesizing information. The classes were used to teach the students practical biostatistics, research methods, the foundations of science based medicine, and patient interviewing skills.

Methods: Working collaboratively with medical school faculty, the librarian redesigned the EBM and biostatistics curriculum. The librarian developed classes have moved away from teaching traditional library information gathering skills to focus on practical biostatistics, research methods, foundations of science based medicine, and patient information interviewing skills. We have shifted from mechanics of searching resources to understanding and interpreting information. The librarian teaches the

biostatistics and research methods necessary to make an informed interpretation of journal articles and point of care tool content. The ultimate goal of this ongoing longitudinal curriculum is repeatedly practicing and perfecting these knowledge domains in order to directly answer patient questions and improve patient care. This new content direction is being evaluated using faculty feedback and student performance data. Expected outcomes are increasing integration of the librarian into the curriculum, and students with increased skills in EBM.

Results: Formal assessment has been difficult, with few students' responses to course surveys. This is not unique to this content, as response rate to surveys by students is low across all courses in the curriculum. Additionally, this approach has led to greater involvement of the librarian in all points in the 4 year curriculum.

Conclusions: Student engagement in EBM and biostatistics has been increased by placing them in a practical, professional context. Student surveys report better understanding of the topics after the sessions, as well as better understanding their value to medical professionals. Faculty are reporting a longitudinal increase across the curriculum in students' use of evidence and comfort engaging with research methods and interpreting statistical results. Although early reports are promising, further study is needed to track the impact of this new approach to student COMLEX scores and other measures of student success.

Engaging Medical Student Learning with Grand Rounds Case Presentations: A Tale of Two Libraries

Lori Fitterling
Digital Services/Reference Librarian, D'Angelo Library, Kansas City, Missouri

Erin Palazzolo
Library Director and Assistant Professor of Medical Informatics, Health Sciences Library, Parker, Colorado

Objective: Information based decision making and clinical presentation skills are important aspects of medical education. By adding a medical informatics course taught by librarians in first-year curriculum, two osteopathic university libraries have been instrumental in teaching strategies used in critical thinking, information literacy, and case presentation skills. This project shows the steps two libraries took to develop medical informatics curriculum.
Methods: Two osteopathic university librarians have documented their experiences with medical informatics courses, beginning with defining learning objectives and determining course format/content, and including a grand rounds case assignment which student groups present. Both programs utilize lectures, exams, assignments, case prep and case presentation as instruction methods. At both universities, students must demonstrate the ability to critically review health information, apply literature

search principles, answer clinical questions, and incorporate osteopathic treatment, medical ethics and patient education aspects of clinical cases. Librarians facilitate the collaboration between basic science and clinical faculty as mentors in a collaborative learning experience. Some differences in each university's methods have also been noted.

Results: Assessment of learning outcomes at both universities that were measured through course exams, student focus groups, surveys, library resource usage, etc., show that students were able to work through clinical diagnosis and make informed decisions for grand rounds cases utilizing information literacy skills. Comparison of each universities approach to grand rounds show distinct differences, but learning outcomes are similar. Collaboration between the library, the Dean/Provost, Academic Affairs and faculty has allowed an innovative course model in both university environments. Additionally, the shifting role of the traditional medical librarian to a classroom intensive, medical informatics instructor has further opened the doorway for inclusion in other aspects of the medical school curriculum.

Conclusions: At two osteopathic universities, the acceptance of medical informatics courses taught by librarians in first-year curriculum has been overwhelmingly positive but it could not work without administrative support. This model of library instruction expands on the historical library instruction session by shifting the focus of student learning from a lecture format to an independent, self-directed learning model focused on finding medical information, critically evaluating that information and applying the knowledge in a clinical setting. It provides key experience in navigating digital resources and presenting cases in a grand rounds format, skills which are extremely beneficial for student doctors.

Monday, May 16, 2016, 10:30 AM – 11:55 AM
Room: 205B

Session: Using Assessment Data to Drive the Big Picture

Moderator: Stewart Brower, AHIP

Using Assessment Data to Drive the Big Picture

Katie A. Prentice, AHIP
Associate Director for User Experience and Assessment, Schusterman Library, University of Oklahoma-Tulsa, Tulsa, Oklahoma

David A. Nolfi, AHIP
Health Sciences Librarian & Library Assessment Coordinator, Duquesne University, Pittsburgh, Pennsylvania

Lorie Kloda, AHIP
Librarian, Concordia University Library, Montreal, Quebec, Canada

Suzanne Shurtz, AHIP
Associate Professor/Instructional Services Librarian, Medical Sciences Library, Texas A&M University, College Station, Texas

This program will bring together a panel of speakers to explore the many facets of library assessment. To illuminate and develop a knowledge of assessment across health science libraries, the panel will share experiences with evidence-based librarianship, using existing data effectively with administration, leveraging specific assessment tools, as well as experiences with assessing library programs. Participants will have opportunities to ask questions and explore ideas and discuss how assessment works. The panelists will focus on driving library success and creating an assessment-friendly environment.

Monday, May 16, 2016, 10:30 AM – 11:55 AM
Room: 104C

Session: Where Does Grey Fit into Mosaic? A Discussion of the Use, Value, and Practicality of Grey Literature in Systematic Reviews

Moderator: Caitlyn Ford

Where Does Grey Fit into the Mosaic? A Discussion of the Use, Value and Practicality of Grey Literature in Systematic Reviews

Caitlyn Ford
Research Information Specialist, CADTH, Ottawa, Ontario, Canada

Kelly Farrah, AHIP
Research Information Specialist, CADTH, Ottawa, Ontario, Canada

Carol Lefebvre, HonFCLIP
Independent Information Consultant, Lefebvre Associates Ltd, Oxfordshire, United Kingdom

Melissa L. Rethlefsen, AHIP
Deputy Director / Associate Librarian, Spencer S Eccles Health Sciences Library, Salt Lake City, Utah

Margaret Sampson, AHIP
Manager, Children's Hospital of Eastern Ontario, Ottawa, Ontario, Canada

Join this special content session for an interactive discussion and debate on the value, practicality, and methodology of grey literature in the systematic review search process. The profession appears to have differing views on considering grey literature as part of the systematic search; regarding the questionable validity and quality of non-commercially published literature, the non-standardized search methods, and time spent searching vs value of retrieved documents. To start the session, the facilitator

will introduce the subject of grey literature, and explain how it is a potentially contentious issue among systematic review searchers. Discussants with differing views on grey literature will offer their opinions, views, and arguments derived from their professional practise, as well as any supporting literature. The discussion will be facilitated with questions on methodology, use, and practicality of grey lit in systematic reviews. The audience will be asked for questions, comments, and opinions throughout the 90 minutes, creating a dialogue between the panel and audience. The discussion will be extended to an audience beyond the room through taking questions, and comments via Twitter, using the hashtag #GreyInMosaic.

Monday, May 16, 2016, 10:30 AM – 11:55 AM
Room: 201F

Session: Nursing Support

Moderator: Shannon Long

Teaching Nursing Students Information Literacy Skills: Faculty's and Instructor's Perception of Collaborating with Librarians RESEARCH

Mary Chipanshi, AHIP
Librarian, University of Regina Library, Regina, Saskatchewan, Canada

Chau Ha
Librarian, Saskatchewan Polytechnic Library, Saskatoon, Saskatchewan, Canada

Ann-Marie Urban
Assistant Professor, Faculty of Nursing, Regina, Saskatchewan, Canada

Objectives: The objectives of the study were to examine the extent to which nursing faculty at two post-secondary education institutions, the University of Regina and the Saskatchewan Polytechnic (who jointly offer the Saskatchewan Collaborative Bachelor of Science in Nursing (SCBScN) program), consider information literacy (IL) as being important to the professional and academic success of students and to identify barriers to collaboration with librarians.

Methods: A descriptive cross-sectional pilot study was carried out to examine the perceptions of faculty and instructors working with librarians, in the context of teaching IL to nursing students. The nursing program was selected based on the diversity in their faculty and instructors, the collaborative nature of their programs, and their ability to yield enough participants to meet sample requirements. Participants were randomly selected. Using Survey Monkey software, an online survey was distributed to eligible participants resulting in a sample realization of forty participants (n = 40). Data was analyzed using the Software Package for the Social Sciences (SPSS: version 19; IBM Corporation, 2010). Descriptive statistics frequencies and percentages for respondents' answers were calculated. Results revealed that collaboration is seen as a fundamental component to teaching IL skills to nursing students.

Results: Results revealed three significant themes: collaboration of nursing

faculty/instructors and librarians is seen as a fundamental component to teaching IL skills to nursing students; that librarians are key to enhancing nursing student's IL learning; and faculty/instructors would be interested in including librarians in their teaching strategies.

Conclusions: Nursing faculty recognize the importance of IL skills to students' academic and professional success and value their collaborative work with librarians, but few collaborate with librarians due to lack of awareness of librarians' roles and library services. This pilot study's findings have not only significant potential value in informing curriculum development in the nursing program to include IL acquisition skills but can also inform libraries and librarians on how to promote their services and strengthen collaboration with faculty.

Piecing Together a Bigger Picture: Scaffolding Research Skills into the Nursing Curriculum

Kathryn M. Houk
Health & Life Sciences Librarian, San Diego State University Library and Information Access, San Diego, California

Objectives: This presentation will describe the creation of a scaffolded Information Literacy and Evidence-Based Practice learning plan for a BSN nursing program, as well as the learning gains and perceived efficacy of integrating the first-tier of research and evidence-based practice skills into a 200-level nursing course.

Methods: Background: The new health sciences librarian at a large public, undergraduate institution created a scaffolded learning plan for the nursing BSN degree by consulting the Information Literacy Standards for Nursing, the new ACRL Frameworks for Information Literacy, and the EBP research process. The learning objectives of the first tier in a multi-year scaffold of research skills education were met by partnering with an adjunct faculty member in the School of Nursing. The two faculty worked together to integrate increasingly challenging research and evidence-based practice skills into a semester-long class.

Exposure: Students completed a variety of tasks, attended two research-focused classes, and had graded assignments involving the library and research skills throughout the semester. Pre- and post-tests and end of year evaluations were administered to gauge knowledge gains and student reactions to the program.

Results: Significant changes in knowledge were measured between pre- and post-test question means utilizing t-tests for uneven sample sizes. Those questions without significant change had a large percentage of the students answering it correctly during the pre-test. Those students who took the end of course evaluation self-assessed their searching skills prior to the course at a 3 (75%), which they felt had increased to a 4

(50%) or 5 (50%) on a scale from 1-5. Students commented that they found learning about Boolean and the importance of generating keywords was the most useful aspect of the class, but they also felt that the tasks throughout the class remained about the same in level of difficulty. 7 of 8 evaluation respondents felt that the research skills taught in the class were “important” or “extremely important” on a 5-point Likert Scale.

Conclusions: Students showed an increased understanding of search strategy and resource-types available to them from the pre-class and in-class research sessions. While only a few students responded to the evaluation, those who did felt that the skills taught were important. The class is in a holding pattern for the spring semester, but over the summer, the instructor and I will be going over how to take all of the student feedback and results to make improvements.

Assessment of Knowledge and Skills in Nursing and Allied Health Student Information Literacy Instruction: Results from a Scoping Review RESEARCH

Jill Boruff
Associate Librarian, McGill University Library, Montreal, Quebec, Canada

Pamela Harrison
Librarian, Rockyview General Hospital, Calgary , Alberta, Canada

Objectives: To determine how knowledge and skills are being assessed in nursing and allied health student information literacy instruction. To determine whether these assessment methods have been tested for reliability and validity. To provide librarians with guidance on assessment methods that could be used in their own instruction.

Methods: A scoping review of the literature was conducted, following the methods outlined by Arskey and O’Malley. A systematic search strategy was constructed by one author and reviewed by the second author. This strategy was then run in Ovid Medline, and adapted for CINAHL, EMBASE, ERIC, LISA, LISTA, and Proquest Theses and Dissertations from 1990 to January 16, 2015. 4366 articles were found, with 2747 articles remaining after duplicates were removed. The title and abstract of these articles were screened, resulting in 2143 articles being excluded. The remaining 604 full-text articles were screened for eligibility. 137 articles were included for data extraction. This paper will primarily address the results of the data extraction

Results: The data analysis for the 137 included articles is being finalized. Of these articles, the health professions discipline breakdown is as follows: Nursing, 107 articles; Occupational Therapy--Physical Therapy--Speech-Language Pathology, 21 articles; Multidisciplinary, 5 articles; Other health sciences disciplines, 4 articles. Many different examples of performance measures (such as validated tests, locally developed pre-test/post-tests, and course assignments) and attitude measures (such

as surveys, interviews, and focus groups) were employed by all of the disciplines. **Conclusions:** While there are few validated assessments for information literacy instruction in evidence-based practice, there are still opportunities for librarians to improve assessment in these contexts.

Listening to the Conversation: Examining the Dialogue on Scholarly Communication in Nursing RESEARCH

Lisa Demczuk, AHIP
Reference and Nursing Liaison Librarian, University of Manitoba, Winnipeg, Manitoba, Canada

Mayu Ishida
Research Services Librarian, University of Manitoba, Winnipeg, Manitoba, Canada

Objectives: To examine the current dialogue on scholarly communication amongst nursing academics and scholars in order to identify the frequency and vehicles used for discussion of the topic of scholarly communication and to identify themes within the content of the dialogue.

Methods: Data collection: A comprehensive literature search of published and unpublished nursing literature by the following methods: searches of relevant nursing literature databases; hand-searching of selected scholarly nursing journals; searching academic nursing conference proceedings; and, searching for unpublished grey literature produced by scholarly or academic institutions. Study selection: No restriction was placed on type of literature selected. Literature types include editorials, original articles, commentaries, conference abstracts and discussion papers produced between 2011 and 2016 in English. Data analysis: A quantitative and qualitative approach is used by mapping the type and number of sources of scholarly communication discussion and by performing content analysis of the text data.

Monday, May 16, 2016, 10:30 AM – 11:55 AM
Room: 202B

Session: Evidence-Based Medicine Education: Sources and Effectiveness

Moderator: Stephanie M. Swanberg, AHIP

Evaluating the Impact of Evidence-Based Practice Online Learning Modules on Interprofessional Education (IPE)

Elizabeth Stellrecht
Clinical Librarian and Liaison to the School of Dental Medicine, Health Sciences Library, Buffalo, New York

Nell Aronoff
Librarian and Liaison to the School of Medicine and Biomedical Sciences, Health Sciences Library, Buffalo, New York

Jeremiah Grabowski
Online Programs Coordinator, School Of Public Health And Health Professions, Buffalo, New York

Michelle L. Zafron
Coordinator of Reference Services, Health Sciences Library, Buffalo, New York

Amy G. Lyons
Interim Director, Health Sciences Library, Buffalo, New York

Maryruth F. Glogowski
Associate Vice President for Information Services and Systems, Information Services and Systems, Buffalo, New York

Patricia J. Ohtake
Associate Professor, Department of Rehabilitation Science, Buffalo, New York

Objective: This study examined the impact of online learning modules that were designed to improve health professions students' evidence-based practice (EBP) and basic PubMed research skills in anticipation of participation in an interprofessional

EBP activity.
Methods: A team of three librarians, a faculty member, and an instructional designer developed two one-hour online modules designed to provide students with the EBP skills necessary for an in-person, case-based interprofessional EBP activity. Students from eight health professions programs at two institutions completed the two modules before the in-person EBP interprofessional activity. Module 1 introduced students to concepts of EBP, including evidence appraisal. Module 2 focused on procedures for locating evidence using PubMed. The effectiveness of the modules was assessed by a quiz at the end of each module and the Adapted Fresno Test of Competence in Evidence-Based Practice (AFTC) which was administered before and after accessing the modules. Qualitative data were obtained via survey at the end of the project.
Results: Thirty-nine students from dentistry (n=5), dietetics (n=6), medicine (n=5), occupational therapy (n=6), pharmacy (n=4), physical therapy (n=6), social work (n=4), and speech language pathology (n=3) participated in the project. Quiz scores for Module 1 and Module 2 were 83±10% and 76±15%, respectively. Following completion of the learning modules, AFTC scores improved (p=0.001), indicating the modules improved EBP skill competence. Survey data indicated the majority of students felt they learned new information (84%) that helped them contribute to developing a plan of care for a patient (66%) and that they would use the information they learned in the future (92%).
Conclusions: Online learning modules were effective in developing EBP skills for a variety of health professions students. Providing consistent EBP skills education to all health professions students ensured they were equally capable of participating in IPE learning activities involving accessing evidence to develop a patient's plan of care. Student feedback indicated the modules were valued and beneficial. Our findings indicate teaching the same EBP skills to a variety of health professions students using online resources is effective, ensures consistent knowledge, and enables each student to fully engage in interprofessional evidence-based activities.

Implementing a Longitudinal Evidence-Based Medicine Sequence for Pharmacy

Franklin D. Sayre
Pharmacy Librarian, Health Sciences Libraries, Minneapolis, Minnesota

Shannon Reidt
Assistant Professor, College of Pharmacy, Minneapolis, Minnesota

Kristin Janke
Professor, College of Pharmacy, Minneapolis, Minnesota

Keri Hager
Assistant Professor, College of Pharmacy, Duluth, Minnesota

James Beattie
Director MEDS/FCT, Medical School, Minneapolis, Minnesota

Objectives: The objective of this program was to move away from the conventional strategy of teaching drug information and drug literature evaluation to Doctor of Pharmacy (PharmD) students through stand-alone courses and sessions and instead to create an integrated, longitudinal evidence-based medicine (EBM) sequence for first-year students that culminated in a meaningful assessment of EBM skills.

Methods: The EBM Framework—Ask, Acquire, Appraise, and Apply—was used as the basis for defining seven EBM skills that were targeted in an evidence-based, integrated design of learning activities and assessments. This resulted in a longitudinal EBM sequence with 17 learning episodes delivered with eight faculty members and two librarians through six courses in the first year. A Milestone Assessment, called the Modified Fresno Test, was adapted from an evidence-based assessment tool used in medicine and physical therapy.

Results: When compared to third-year students who did not experience the Longitudinal Sequence, first-year students demonstrated a higher level of EBM skill, thus providing evidence that this initiative was effective. This initiative has been reviewed positively by students and faculty and has led to broader faculty engagement in the scholarly teaching of EBM and further expansion of EBM in the Doctor of Pharmacy Curriculum.

Analysis of First-Year Research Poster Projects: An Evidence-Based Approach to Improving Information Literacy Instruction for First-Year Health Sciences Students

Xan Goodman, AHIP
Health & Life Sciences Librarian, Lied Library, Henderson, Nevada

Rogelio Arenas
Doctoral Candidate, Lied Library, Las Vegas, Nevada

John Watts
Undergraduate Learning Librarian, Lied Library, Las Vegas, Nevada

Rachelle Weigel
Library Technician II, UNLV Libraries, Educational Initiatives, Las Vegas, Nevada

Objectives: This paper describes an analysis of sources used by first-year, health sciences students in their final research poster projects. In addition to describing the process for analyzing student posters, we will explain how a team of librarians and a

course coordinator used results from the analysis to improve library instruction in a first year seminar for the health sciences.

Methods: This study sought to answer: a) How well do students select relevant, authoritative sources? b)How well do students follow or adhere to use of correct citation of sources in a final poster project? c) How well do students summarize selected relevant and authoritative sources used to support their research topic? A 50-minute, mandatory library instruction session was conducted from Fall 2013-Spring 2015 to teach how to search for and identify research articles. To evaluate student work, pictures were taken of final project posters for each semester. A total of n=120 posters were randomly selected and analyzed by four team members. A rubric was developed by the team to rate the students' information literacy skills. Inter-rater reliability of posters was established using Krippendorff's alpha coefficient. Posters were rated by individual score and total combined score from the four evaluators.

Results: Each criterion was evaluated in relationship to the total score. This is to say that a value was synthesized by taking the relationship between an individual score for a poster and the total score the poster was given. For example, if a poster was given a 3 for Relevance, and a totally score of 14, then a proportion was formed that could elucidate the nature of each individual score with relation to overall performance (ex. When criterion score was X, then total score was Y). This allowed evaluation of individual criterion with relation to overall performance. From these findings, the weakest areas for students were Summary and Citation.

Conclusions: Students showed weakness with being able to summarize their scholarly articles and difficulty with citing correctly using the 6th edition of the American Psychological Association (APA) citation style. The results demonstrated that students may need more practice with writing annotated bibliographies to connect the topic of their scholarly article to their annotated bibliography coherently. Additionally, students appear to need more guidance and training with using using APA style. The library instruction session for this class was altered to meet some of the some of the needs found in the research process.

Librarians as Part of the Big Picture: Collaborating with Faculty and Instructional Designers to Create a Rubric for Students on How to Use Evidence-Based Medicine Resources

Shalu Gillum, AHIP
Head of Public Services, Harriet F. Ginsburg Health Sciences Library, Orlando, Florida

Deedra J. Walton, AHIP
Head of Electronic Resources, Harriet F. Ginsburg Health Sciences Library, Orlando, Florida

Pamela Herring, AHIP
Electronic Resources Librarian, Harriet F. Ginsburg Health Sciences Library, Orlando, Florida

Nadine Dexter, AHIP
Director, Harriet F. Ginsburg Health Sciences Libraryi, Orlando, Florida

Objectives: To collaborate with Practice of Medicine module faculty to introduce first year medical students to the use of evidence-based medicine resources when answering clinical questions developed during patient encounters. Students in the module were not using evidence-based resources to answer clinical questions in their Patient Encounter Logs (PELs), and instead were relying on internet search results, notes from classes, or consults with preceptors.

Methods: After each preceptor session, students completed a PEL for two patients using LiveText e-portfolio. For each patient, students identified a patient-related clinical question to investigate. Students had to document the question, write a brief answer, and cite their resources. Six librarians were assigned twenty students each. Librarians reviewed the assignment and gave students feedback on the chosen resources. Feedback was structured by a rubric, created by librarians in collaboration with module directors, module coordinator, and an instructional designer. The rubric allowed librarians to give consistent and constructive feedback, determine how much knowledge students had of library resources, determine students' resource preferences, and whether they correctly cited their resources.

Results: Librarians need a face to face meeting with all students prior to students completing PELs. Directions on the original assignment needed to be clearer. Many students did not know how to draft a clinical question, the correct evidence-based resources to use, or how to cite resources. The rubric needs to be reexamined and tailored to include the abnormal situations received. The rubric was necessary in order for the librarians to provide consistent feedback to students.

Conclusions: Librarians’ collaboration with Practice of Medicine faculty resulted in the development of a guide for students to determine which types of evidence-based library resources to use when answering clinical questions. This ultimately led to students having better answers to their clinical questions, more use of evidence-based resources, and familiarity with library resources.

How Does Instruction by Medical Librarians on Using a Federated Search Tool Impact the Result of Medical

Information Research: A Mixed-Method Descriptive Quantitative Study RESEARCH

Stevo Roksandic, AHIP
Regional Director, Mount Carmel Health System Library Services, Columbus, Ohio

Donald S. Pearson
Library Technology Specialist, Mount Carmel Health Sciences Library, Columbus, Ohio

Objectives: The purpose of this study was to examine the effectiveness of using a federated search tool (eSearcher) in a medical library, over other methods of online information search. As a secondary aim, the study examined the impact of library instruction on using eSearcher.

Methods: An anonymous survey was given to graduate students in a nursing college and residents in a graduate medical education program. Its purpose was to gather information on the use of the library's website, including eSearcher. A subset of the surveyed students joined one of two study groups. The experimental group received 10 minutes of instruction on eSearcher; while the control group received none. Each group was provided with a prompt for a guidelines search, and then asked to find the best result using either their usual resources, specifically excluding eSearcher (control), or eSearcher exclusively (experiment).

Results:
Survey
The anonymous survey was taken by 83 individuals roughly evenly divided between nurses and residents. Forty-two percent said they use Google daily to find clinical information, however 71% said they preferred to search for clinical information on the library's website. About 60% were familiar with eSearcher, but only about 30% used it often.
Study
There were a total of 42 graduate students participating in this study (n=42); 23 nurses and 19 residents. Twenty students were placed in the control group and 22 students were placed in the experimental group, roughly evenly divided between nurses and residents.
Three major metrics were tracked: time to complete the guidelines search, correctness of the answer and respondent confidence in their answer. The mean time expended for the control group was 5 minutes, and 7 minutes for the experimental group. The mean correctness score for the control group was 1.90, and 1.68 for the experimental group. The mean confidence level for the control group was 5.95, and 5.27 for the experimental group.

Conclusions: For graduate students eSearcher does not seem to add value to their searching in time, correctness or confidence level. In all three metrics the control group which did not use eSearcher was superior. The groups which used eSearcher

took longer on average to complete their search, got worse results and had a lower confidence that their searches returned clinically useful information. Study limitations had to do with the high level of academic maturity of the study group, the search prompt and the novelty of eSearcher.

Monday, May 16, 2016, 10:30 AM – 11:55 AM
Room: 202D

Session: Collection Management

Moderator: Erin Watson

Saskatchewan Health Information Resources Program (SHIRP): The Next Decade

Susan A. Murphy
Head, Health Sciences Libraries, University Library, University of Saskatchewan, Saskatoon, Saskatchewan, Canada

Valerie Moore
SHIRP Librarian, Saskatchewan Health Information Resources Program, Saskatoon, Saskatchewan, Canada

Objectives: The Saskatchewan Health Information Resources Program (SHIRP) transitioned from a Partnership to a Program of the University of Saskatchewan's (UofS) University Library in July 2014. This paper will provide a brief history of SHIRP, including the changes that brought SHIRP to its present iteration, and how it has been integrated into the operations of the University Library.

Methods: SHIRP licenses a sub-set of the UofS electronic resources to enable health care practitioners in the province to be more effective in their work with UofS health sciences students during placements, internships, etc. Changes to the funding for SHIRP initiated its realignment as a program of the University Library; a program that continues to see partnerships as a critical component. Improvements to administrative practices and technological infrastructure reflect an innovative approach to providing access to a common set of electronic resources to health care practitioners in a complex environment. Outcomes of the realigned program will be of interest to librarians exploring the role of collections in furthering the teaching of health sciences students in a distributed learning environment, as well as those studying models of how Academic Health Sciences Libraries provide resources to health care practitioners.

Results: The realignment and integration will identify which operational activities within the SHIRP office are similar to those of the University Library and can be more effectively managed centrally. It will also bring SHIRP policies and procedures into compliance with fiscal requirements and risk management practices of the University

of Saskatchewan. It is not anticipated that any of the structural changes within SHIRP will negatively impact its collections and services. The realignment will provide a way forward in terms of demonstrating the value of SHIRP both within the university and to the province.

Conclusions: It is anticipated that the realignment will result in efficiencies in financial management, licensing, and e-access problem solving, as well as a greater understanding by University Library employees of how SHIRP's collections and clients intersect with, but differ from, the University Library's. More broadly, the realignment will also clarify province-wide the context for SHIRP's governance, accountability, and budgetary context.

A Consortial Breakup with Elsevier: Deciding Not to Subscribe to ClinicalKey

Sarah McClung
Collection Development Librarian, UCSF Library, San Francisco, California

Rikke S. Ogawa, AHIP
Team Leader for Research, Instruction, and Collection Services, Louise M. Darling Biomedical Library, Los Angeles, California

Bruce Abbott
Reference Librarian, Blaisdell Medical Library, Sacramento, California

Objectives: The planned discontinuation of MDConsult by its publisher, Elsevier, caused the subscribing University of California (UC) Libraries to make decisions regarding whether or not to migrate to ClinicalKey, MDConsult's successor, and how to communicate those changes to the platform with our users.

Methods: The UC Libraries' health sciences selectors spent several months evaluating usage reports, title lists, pricing, and content access options, as well as negotiating with Elsevier representatives, in order to make an informed decision about migrating to ClinicalKey. In anticipation of the ClinicalKey offer falling through, each campus prepared a contingency plan, which included communication with campus stakeholders and steps to fill in collection gaps through journal subscriptions and book purchasing. This paper will discuss the results obtained from walking away from ClinicalKey and the variety of approaches the system's libraries took to meet consortial and local campus needs.

Results: The UC Libraries ultimately decided not to subscribe to ClinicalKey due to the untenable cost increase and unfavorable content model. The contingency plans were put into place with communications going out to our respective campuses and further decisions being made regarding filling in collection gaps. Journals were added

to the multi-campus contract several months after MDConsult was discontinued. Some campuses selected ebooks on alternate platforms, while others decided to purchase only print copies. Regardless of method; however, there have been remarkably few complaints, to date, from our users as a result of losing a top clinical resource.

Conclusions: Our largely positive experience unsubscribing from a large, well-known medical education database is encouraging as we continue to analyze and evaluate existing and future resources in order to be good stewards of UC funds, meet user needs, and uphold the standards of the profession. Our undertaking demonstrates both the benefits and complications of a collaborative approach to licensing a core clinical and educational information resource.

Putting the Pieces Together: Finding a Point-of-Care Solution for an Academic Medical Center

Denise Hersey
Clinical Support Librarian, Cushing/Whitney Medical Library, Yale University, Cheshire, Connecticut

Janene Batten
Reference Librarian for Nursing, Cushing/Whitney Medical Library, New Haven, Connecticut

Mark Gentry
Clinical Support Librarian, Cushing/Whitney Medical Library, New Haven, Connecticut

Holly K. Grossetta Nardini
Research and Education Librarian, Cushing/Whitney Medical Library, New Haven, Connecticut

Nathan Rupp
Head of Collection Development and Managemen, Cushing/Whitney Medical Library, New Haven, Connecticut

Objectives: In recent years, price increases have outpaced library funding, so librarians need to make evidence-based decisions regarding which resources they acquire and keep. To this end, our academic medical library aassembled a task force composed of librarians, physicians, nurses, and hospital IT staff to compare and evaluate DynaMed Plus and UpToDate and then decide which product best meets the needs of our associated hospital, informing the subscription renewal process for 2016-2017.

Methods: The task force conducted seven focus groups. Participants represented a cross section of hospital staff including nurses, residents, physicians and

hospitalists. Librarians on the task force wrote a report summarizing the findings of the focus groups which they presented to the larger group. In addition, the librarians created a chart comparing the features of both resources, and gathered information on which of these resources peer-institutions licensed. With this information, the task force came up with four viable options for licensing UpToDate and/or DynaMed Plus in the 2016-17 fiscal year.

Results: Results from the focus groups indicated that at least one decision support resource was necessary for the Yale New Haven Hospital. Participants felt that DynaMed Plus would be most useful for quickly finding clinical information, however, they felt that UpToDate provided them with necessary information in a context particularly useful to a tertiary hospital with an academic affiliation. Additional opinions on features of both resources were also recorded. Task force members noted the difference in breadth of topics between the two resources, with UpToDate containing many more clinical topics than DynaMed Plus. However, UpToDate's significantly higher licensing cost also contributed to the recommendations made by the task force.

Conclusions: Once the task force had ranked the four resulting options , the group wrote up a recommendation given to the hospital and university administrators to inform their final decision on future licensing of these products.

Beyond Patching It Together: Developing and Applying an Evaluation Algorithm for Serials RESEARCH

Rikke S. Ogawa, AHIP
Team Leader for Research, Instruction, and Collection Services, Louise M. Darling Biomedical Library, Los Angeles, California

Juan Jaime
Public Service Supervisor, Louise M. Darling Biomedical Library, Los Angeles, California

Objectives: To develop an evaluation algorithm for local print and online serial subscriptions which would be consistently maintained and applied for regular evaluation of collections.

Methods: In a library system, where 75% of collections budgets are spent on consortial purchases, UCLA Biomedical Library has a limited resources with which to administer local purchases or subscriptions in support of multiple health sciences and life sciences disciplines. Local subscriptions may be a result of many factors – e.g., publishers not wanting to deal with consortia, small niche subject areas supported by one campus, no online equivalents, etc. Historically, comparisons of several prestige and use factors have been evaluated at a single subscription level without objectively evaluating subscriptions within the larger subject collection context. With tightening

budgets, it is necessary to compare and evaluate effectiveness of subject collections on a larger scale, with a sustainable, subject sensitive evaluation metric.

Results & Conclusions: A description of the algorithm’s development will be presented along with the potential workflow impact and cost savings to the library.

Monday, May 16, 2016, 10:30 AM – 11:55 AM
Room: 203B

Session:Planning For Your Library's Future

Moderator: Jeff Mason, AHIP

“Act Like a Librarian, Think Like an Administrator”: Using Data to Communicate with Administration

Elizabeth Laera, AHIP
Medical Librarian, Princeton Baptist Medical Center, Birmingham, Alabama

Objectives: Hospital librarians have long sought ways to prove their value and keep their doors open, but struggled to communicate with administrators. The goal of this review is to present a range of ideas and methods for collecting data and numbers that can be used in conversations with and presentations to hospital administrators.

Methods: BRIEF DESCRIPTION When faced with a battle over budgets, new resources or services, or even their own jobs, hospital librarians often turn to anecdotes, personal stories, or “elevator speeches” to sell themselves to various administrators. While useful and often successful, many administrators are number crunchers and respond better to hard facts and data.

Based on personal experience and information gathered from the literature, this author seeks to review the various methods for collecting data and using numbers as a method of communication. Budget analyses, return-on-investment calculations, and usage statistics, among others, will be highlighted. Also featured are tips on picking the right administrators to work with, data visualization, and ideas for thinking like an administrator.

Results: PARTICIPANTS AND SETTING Librarians in hospitals of all sorts and sizes who are seeking new ways to connect with hospital administrators and speak to their chief concern: the financial bottom line.

Conclusions: Hospital librarians are well aware of their own value and their potential to benefit many aspects of healthcare. But even good librarians struggle to prove this

to administrators who see only expenditures, not income. Hospital librarians must find ways to use numbers, the language of business, to advocate on their own behalf.

Medical Library Marketing: An Investigation of Current Definitions and Practices RESEARCH

Martha E. Meacham
Medical Librarian, VISN1 Knowledge Information Service Veteran Affairs Medical Center - Manchester, NH & Bedford, MA , Nashua, New Hampshire

Objectives: Marketing is essential for medical libraries. However, there has been no profession wide analysis of marketing definitions and incorporation. This study examines the current state of marketing understanding and practice in medical libraries. Specifically, this study looks at individual, institutional, and profession wide marketing definitions, practices, trends, and gaps, thereby creating a solid foundation and framework for future work.

Methods: To study current profession wide opinions and practices concerning medical library marketing, a survey targeting professional medical librarians was distributed through listserves and targeted emails. The 22 multiple choice and open-ended question survey focused on an individual's perceptions and definitions of marketing for themselves, within their institutions, and for medical librarianship as a whole.

Results: The survey had 146 respondents, primarily from hospital/clinical (54%) and academic (39%) librarians representing a full range of professional experience. 97% of respondents thought marketing was important for medical/health science libraries. 95% also said that they participated in marketing activities within their library. There was a very wide range of individual definitions for marketing, most focusing on promotion or singular activities. Marketing was reported as being very (40%) or extremely (25%) important to respondent's institutions, but incorporation of marketing principles and practices into daily and strategic activities was reported as only limited (35%) or average (35%). 76% reported that their library had no documented, formal, or specific marketing plan. Most institutional definitions of marketing focused on specific activities such as posters, emails, or social media. Respondents rated the amount of information about marketing for medical/health science libraries as mostly poor (13%), fair (35%), or average (19%). Quality was also rated as mostly poor (7%), fair (29%), or average (29%).

Conclusions: Results demonstrated the perceived importance of marketing in medical/health science libraries, while showing a very wide range of definitions, understanding, and approaches to actual practice and implementation. Respondents reported that marketing influenced their work or affected their library, but most had no or minimal understanding, documentation, or guidance. While most institutions engage in marketing activities, and it is perceived as strategically important, little has been made explicit or standardized. Informal or non-existent planning and documentation

leads to inconsistent and ineffective marketing efforts. This was reflected in many open-ended responses. Results clearly indicate a gap in practice and information available to assist work in this area. Time, money, and effort can be saved by systematically incorporating marketing process in to everyday work.

Exploring Tomorrow: Development of a New Technology Exploration Program and Space

Fred W. Z LaPolla
Knowledge Management Librarian, NYU Health Sciences Library, New York, New York

Sergey Zeygerman
Senior Technician, Technology Support, NYU Health Sciences Library/Medical Center IT, New York, New York

Jeff D. Williams, AHIP
Associate Director, NYU Health Sciences Library, New York, New York

Objectives: To support exploration of new technologies by students, educators, and researchers, a technology exploration space and program will be part of a new academic medical center library facility. This space invites exploration of data visualization, educational technology and instructional design, 3D printing, and large-scale interactive displays using a combination of hardware, software, and consultation with librarians and educational technologists.

Methods: A group of library staff arranged a series of individual and small-group discussions with prospective users to discuss potential ways of empowering their exploration and development of expertise in emerging technologies and innovative instructional design. Librarians also reached out to colleagues working in these areas within their medical center, affiliated libraries, and other academic health science libraries. Further steps included 1) pursuing formal and informal education and experience in potential technology areas to assess feasibility of providing support and resources, 2) formalizing partnerships with librarian colleagues and staff at affiliated libraries around collaborative program expansion, 3) space planning, 4) dedicating staff effort toward the program, and 5) exploration of initial and longer-term funding partnerships.

Results: As of February 1, 2016, construction of the new technology exploration space is ongoing. A librarian is now dedicated to providing data visualization support, and is making connections with staff in the Medical Center and affiliated libraries. A team of faculty and staff is supporting the new 3D printing service. Independent of the space, online promotion of new technology exploration services, in particular 3D printing and data visualization, is generating interest within the medical center. The

Library is providing in-person and online consultations in these areas, and will offer introductory workshops to capitalize on this interest within the community.**Conclusions:** Despite a longer time horizon for completion of the Technology Exploration Space, the positive response of the medical center community to the available new services is giving the Library confidence that technology exploration represents a viable library service opportunity.

Understanding User Needs Through Focus Groups RESEARCH

Rick Wallace, AHIP
Assistant Director, Quillen College of Medicine Library, Bristol, Tennessee

Nakia Woodward
Sr. Clinical Librarian, Quillen College of Medicine Library, Johnson City, Tennessee

Objectives: The objective of this study was to better understand the needs of users of an academic health sciences library.

Methods: A focus group was conducted using structured interviews. The groups interviewed consisted of three students each from Medicine, Pharmacy, and Physical Therapy. Interviewees were probed as to their experience with the library facility and the information training they received in their professional schools. Another focus group was conducted with six participants. Two faculty members each from Medicine, Pharmacy, and Physical Therapy were interviewed. Faculty were asked about the information training they provided students. The data was analyzed for trends using qualitative software.

Results: Better relationships were developed between library users and staff. Staff enjoyed learning the focus group methodology and thought it was a valuable tool. New insights were gained for current and future library operations.

Conclusions: A focus group is an excellent research tool for assessing the information needs of users, the degree to which the library is filling those needs, and learning of new needs.

A Five-Year Information Technology (IT) Roadmap for a Health Sciences Library RESEARCH

James King
Information Architect, National Institutes of Health, Bethesda, Maryland

Ben Hope
Branch Chief, National Institutes of Health Library / Information Architecture Branch, Bethesda, Maryland

Kathleen B. Oliver
Program Manager, National Institutes of Health Library, Bethesda, Maryland

Lisa M. Federer
Research Data Informationist, National Institutes of Health Library, Bethesda, Maryland

Keith W. Cogdill, AHIP
Director, National Institutes of Health Library, Bethesda, Maryland

Objectives: The NIH Library, a health sciences library serving a national biomedical research institution asked: What is the state of information technology (IT) infrastructure for biomedical libraries? What is the state of IT infrastructure and use at our institution beyond the library? Can we develop a library IT roadmap to meet the requirements for serving a major research institution for the next five years?

Methods: To describe current information technology infrastructure and use in health sciences libraries, we collected data from internal and external sources, using key informant interviews and structured observation. We developed case studies by interviewing eight peer institutions selected from an international pool as reference points. The interview questions included information technology infrastructure, delivery schema, security requirements, and anticipated future trends. Home institution data emerged from both key informant interviews and direct observation of hardware, software, staff, work settings and workflow, internal and external mobility, and technology use in library, clinical and basic research settings. Informationists used new and established relationships to collect observational data in clinical and laboratory research settings. Home institution workflow, IT infrastructure and use were analyzed in the context of external case studies and home institution requirements, and a five year IT Roadmap developed.

Results: Interviews of both peer institutions and NIH Library technology end-users identified 1) accessibility (the ability to get information anywhere, anytime), 2) data management and correlation, and 3) support for collaboration as key needs. Best practices identified from case studies included 1) structured, organized processes around IT development, 2) close collaboration of libraries with IT departments, 3) rigorous and regular needs assessment with results folded into library planning, 4) significant effort to embed services in end-user workflow, and 5) innovative use of social media. Technical capabilities, responsive to results and structured around “swim lanes” such as data management, communication, publishing (website, mobile apps, etc) were mapped to existing and required technology infrastructure.

Conclusions: The effort enabled the development of a flexible 5 year plan. The plan

should prove to be an effective tool in seeking budgetary support for and staged implementation of technology development, initiatives and support appropriate to a major research institution such as NIH.

Monday, May 16, 2016, 10:30 AM – 11:55 AM
Room: 203D

Session: Working with Researchers

Moderator: Jennifer Dinalo

Design, Implementation, and Evaluation of a Medical Library Program to Support Biomedical Research in the 'Omics Era

Rolando Garcia-Milian
Biomedical Sciences Research Support, Yale Medical Library / Curriculum & Research Support Department, New Haven, Connecticut

Janis Glover
Education Services Librarian, Cushing/Whitney Medical Library/ Curriculum & Research Support Department, New Haven, Connecticut

John Gallagher
Director of the Cushing/Whitney Medical Library (interim), Cushing/Whitney Medical Library, New Haven, Connecticut

Objectives: Design and implement a sustainable program that supports biomedical research at Yale University. Our program responds to the challenges posed by omics (e.g. genomics, transcriptomics, proteomics, etc.) technologies, identifies the role and place of the Yale Cushing/Whitney Medical Library in the high-throughput omics research cycle, and is based on the organizational culture of the institution.

Methods: Key challenges posed by omics technologies on preclinical and clinical research are: rapidly generating high amount of diverse omics data; integrating these diverse data; exponential increase in the biomedical literature and databases; use of pathway and network-based methods to analyze and visualize information; and low reproducibility of preclinical research and its translation into the clinical setting, among others. Based on these challenges, a program was designed and implemented to support biomedical researchers including faculty, clinicians, grad students and postdocs in three areas: instruction, services, and resources/tools. Program evaluation consisted of carefully recording statistics for each of the three main components; feedback was elicited, collected and analyzed. Based on feedback, a grassroots community, “End-user Bioinformatics Network" (EBNET) and its advisory group, was also created with the mission of collaborating on the professional development and research support of its members.

Results: During the first 19 months of the research support program, 43 training sessions were directly provided or hosted by the medical library. A total of 1035 individuals have attended these sessions out of 1720 registered, including 296 waitlisted. Topics of training included: Natural Language Processing tools, genome browsers, networked data visualization tools, enrichment analysis, genetic variation resources, etc. Based on user feedback, the library began providing institutional access to proprietary bioinformatics software for the functional analysis of high-throughput data (e.g. MetaCore and Ingenuity Pathway Analysis, TRANSFAC, etc.). A total of 96 consultations (average time duration 1 hour) were provided to 106 researchers.

Conclusions: Yale Cushing/Medical Library has created a program that supports biomedical research in the omics era from an end-user perspective. This program fills an identified gap in the high-throughput data life cycle in terms of data annotation of research results, and hypothesis generation/narrowing. This presentation will discuss on the strategies used to implement this program.

Bringing Bioinformatics into the Library with an Informatics Workshop Series

C. Tobin Magle
Biomedical Sciences Research Support Specialist, Health Sciences Library, Aurora, Colorado

Objectives: Starting informatics services in the library can be daunting, even for those who are familiar with useful databases and tools. Often, the biggest issue is convincing researchers that the library is useful informatics resource. This presentation discusses a simple way to begin associating the library and informatics: a workshop series.

Methods: Developing robust informatics content to include in library classes takes time. However, the University of Colorado Anschutz Medical Campus is fortunate to have a wealth of local informatics experts who are willing to share their knowledge with others. Thus, the Health Sciences Library began a monthly Informatics Workshop Series (HSL-IWS). Local experts are contacted and asked to present an interactive session on informatics tools and databases that they have created or are familiar with to a novice audience. Additionally, HSL-IWS is used to test run new library classes before they are offered regularly. These workshops are advertised campus wide, and attendees are asked to rate the effectiveness of the session through a post course survey. These surveys are used to inform the content of future sessions.

Fostering a Collaborative Research Culture: An Exploratory Case Study on the Librarian's Contribution to Team Science

Young-Joo Lee
Senior Clinical Librarian, Howard University, Washington, District of Columbia

Objectives: Biomedical research has become an increasingly complicated, collaborative endeavor among basic, clinical, translational and behavioral research. Such multi-disciplinary research is now referred to as "team science." The aim of this study is to explore how librarians can contribute to this new and exciting challenge for researchers, what difficulties librarians may face in doing so and how to overcome these difficulties.

Methods: The author will examine the research questions using her experience of joining the oral cancer research team on her campus. The research, funded by NIH, is a true example of team science in that it is comprised of three different projects (basic, clinical and behavioral), and each project is composed of researchers from two different institutions. The author was tasked with building a system that would streamline the data collection and future sharing of the data. Using this case, the author aims to evaluate her contribution to the collaboration of the research team and determine how her work has contributed to fostering team science. The methods will be both quantitative (survey) and qualitative (interview of researchers). The study will also explore how this case can be used to educate campus researchers on the benefits and challenges of team science.

Promoting a Culture of Research Through a Research Interest Group

Lilian Hoffecker
Research Librarian, Health Sciences Library, Aurora, Colorado

Lisa K. Traditi, AHIP
Head of Education and Reference, University of Colorado Health Sciences Library/Education & Reference, Aurora, Colorado

Dana Abbey
Health Information Literacy Coordinator NNLM/MCR, Health Sciences Library, Aurora, Colorado

Heidi Zuniga
Digital Resources Librarian, Health Sciences Library, Aurora, Colorado

C. Tobin Magle
Biomedical Sciences Research Support Specialist, Health Sciences Library, Aurora, Colorado

Ben Harnke
Education & Reference Librarian, Health Sciences Library/Education and Reference, Aurora, Colorado

Deirdre Adams-Buckely
Library Technician II, Health Sciences Library, Aurora, Colorado

Objectives: This presentation will describe the activities of the Research Interest Group, a library committee interested in learning and teaching about the research process in order to establish a "culture of research." Our goal is to encourage librarians and staff alike to engage in research and quality improvement projects.

Methods: Inaugurated in Spring 2014, after several brainstorming sessions to generate ideas, the Research Interest Group initiated a series of workshops where members took turns facilitating the discussions. Meetings took place monthly and relate to one or more of the components of the research process, namely: topic selection and literature review; study design; grants and funding; data collection; data analysis; dissemination; and impact analysis. In addition to organizing the meetings, each facilitator provided one or more evaluation question which will be used to assess the program in the future. To date, we've had or plan to have sessions covering several components of the research process including sessions: where a research idea was pitched (Topic Selection); devoted to the critical appraisals of research methods (Study Design); discussing poster presentations (Dissemination); and on raising one's research profile (Dissemination, Impact Analysis).

Results: The interactive workshop format has been beneficial in making interest group members participate as facilitators and as audience members. However, time commitment to the interest group has been a challenge. Web conferencing technology is useful allowing distant guest speakers to present live or other librarians to participate.

Conclusions: A research interest group may offer support to academic librarians who are expected to be involved in research for promotion purposes but have little understanding of how to get started. It can also inform all library personnel on what bench scientists, field scientists, clinical investigators, and other researchers experience in order to assist them more effectively.

Open Insight: Facilitating Conversations and Building a Community Around Open Science

Jacqueline Wirz
Director of Career & Professional Development and Graduate Student Affairs, Research Data Ninja, Assistant Professor, Oregon Health & Science University, Portland, Oregon

Robin Champieux
Scholarly Communication Librarian/Assistant Professor, Oregon Health & Science University, Portland, Oregon

Objectives: The Oregon Health & Science University Library has recently launched Open Insight - a year long project designed to educate and engage our research community about open science and open data initiatives. This paper will highlight the development of the programming (including invited speakers, open forums, and project development), and discuss the challenges and opportunities this program has experienced.

Methods: The Open Insight project is funded by the NN/LM Pacific Northwest Region. The funding is being used to develop programming, specifically 1) inviting speakers, 2) developing opportunities for community education, 3) project development, and 4) sending a student and a postdoc from our institution to the international OpenCon in the Netherlands. To execute the project, OHSU has developed an advisory board of interested students, postdocs and researchers. The multimodal approach to open science education and community engagement will help develop a stronger program.

Monday, May 16, 2016, 1:00 PM – 2:25 PM
Room: 201D

Session: Lightning Talks #2

"Throwing That Away? We'll Take it" -- How One Library Created an Archive by Documenting and Preserving the Organization's History

Lori Fitterling
Digital Services/Reference Librarian, D'Angelo Library, Kansas City, Missouri

Marilyn J. DeGeus
Director of the Library, D'Angelo Library, Kansas City, Missouri

Robyn Oro
Cataloger/Serials Assistant, D'Angelo Library, Kansas City, Missouri

Objectives: Traditionally, libraries are custodians of an organization's history, but some institutions have fallen short in this area. This project developed as librarians responded quickly when historical materials were marked for removal. Librarians filled the need for compiling, cataloging, preserving, and making university historical materials publicly available. The Library Archives was created and has become an established service of the library.

Methods: Various methods were used in this project beginning with obtaining permission to clean out storage areas and transport archival materials to the library. The sorting, cleaning, cataloging, indexing, photographing, researching, and making some determination as to restoration needs were all accomplished over a two year period, and the work continues. As items were completed, they were transferred to permanent storage shelves within the library or put on display. A planetary scanner was purchased in 2013 for the digitization of historical documents, osteopathic pamphlets, and other archival and library materials. CONTENTdm digital management software was purchased to present higher resolution images and fully searchable text and Librarians continue to update the catalog of archival/historical items housed in the library.

Results: Through the efforts of the Kansas City University librarians, historical photos, artifacts, documents, objects, etc., were successfully transferred from other areas on campus to the library for cleaning, preservation, and proper handling. Exhibit cases

were purchased for the display of historical materials, and InfoGuides were created of the digital photographic collection of university buildings, hospitals, and various other photographs. Yearbooks and obscure osteopathic pamphlets have been made available from the library's website and a library staff member is now responsible for the archives collection. This repository has become a vital resource to our institution and others interested in the history of Kansas City University.
Conclusions: The *Library Archives* has become an integral part of the library, the university, and a clearinghouse for historical information about the Kansas City University of Medicine and Biosciences. It has filled the role of archival repository for the rich history of an osteopathic university and into its' 100th year, has been used heavily for the centennial celebration. Going forward, this library collection and service will continue to document the imprint KCU has on medical education.

Using Omeka for Online Exhibits

Andrew Hickner
Web Services Librarian, Cushing/Whitney Medical Library, Yale University, New Haven, Connecticut

Melissa Grafe
John R. Bumstead Librarian for Medical History, Cushing/Whitney Medical Library, New Haven, Connecticut

Francesca Livermore
Digital Projects Librarian, Olin Library, Middletown, Connecticut

Kerri Sancomb
Exhibits Production Coordinator, Sterling Memorial Library, New Haven, Connecticut

Objectives:
Medical libraries with significant special collections can expand access to their rare materials by creating and hosting online exhibits. The need for an online exhibits platform at Yale University was demonstrated by the number of "legacy" online exhibits using a variety of applications which are hard to support, update, or maintain. Our objective is to evaluate Omeka (http://omeka.org) as a platform for online exhibits.
Methods: The Cushing/Whitney Medical Library, in collaboration with the other Yale University Libraries (YUL), conducted a pilot of the open-source platform Omeka. To track the impact of the project, we collected Google Analytics data including number of sessions, users, and pageviews over a one-year period starting February 2015. Other metrics include number of exhibits created, number of objects made available to the public, and number of users creating exhibits. Our evaluation also included qualitative measures, including assessments of the tool's theming capabilities; ease of use by library personnel; technical and staffing requirements; and need for documentation. Both aggregate data and data specific to our medical historical exhibits was collected.

Results: As of January 2016, our Omeka instance had 15,165 pageviews from 1,978 users over the course of 2,819 sessions (February 6, 2015 - January 13, 2016). As of January 2016, 1648 items were made publicly available across 15 exhibits, with 32 users. In terms of theming capabilities, out of the box theme alternatives are currently fairly limited. Compared to more commonly used content management systems such as Drupal, any custom theming requires more advanced web development and design expertise. There were a number of technical and staffing requirements required to host, customize, maintain, and train users for the system. We also found it necessary to develop local documentation and training, in order to enforce standards and quality control across exhibits.
Conclusions: YUL users have found Omeka to be an effective platform for online exhibits, particularly when large numbers of potential exhibits and curators are involved. However, the product's technical and training requirements are non-trivial. Our experience offers some guidance on what librarians should consider when selecting a platform for showcasing their rare objects online.

Guiding Concepts Lead to Successful Outcomes in a Library Site Redesign

Susan Bridgers
Distance Education Librarian, Chamberlain College of Nursing Library, Asheville, North Carolina

Valerie Meyer
Clinical Library Specialist, Library, maryland heights,, Missouri

Objectives: This spring our library website overhaul became imminent with the release of Springshare's v2 LibGuides platform. We considered the impact a redesign could have for the audience we serve and aimed to maximize this opportunity. This lightning talk reviews the guiding concepts, considerations, and outcomes of the adopting the structure we chose for the new site.

Methods: Because we align our library's mission with our college's, our mandate is to take extraordinary care of students, expecting that our care leads to better student outcomes and experiences. This in turn is more likely to lead to our nurses taking extraordinary care of patients and families. We translated "taking extraordinary care of students" to mean our library site had to be highly accessible and functional. To affect our mandate, we chose the working concepts of simplicity, clarity, and multi-point accessibility to librarian support.

Results: We built pages rich with content on a simple three level site structure: Home or Resource Search pages, Program Directory pages, and Course Guide pages. This included the inviting access points of librarian help by email, chat and phone across all time zones. The best student and faculty support, though, we put into the site structure

was our choice to build a course guide page for each class in each curriculum. These pages contain the links to the required readings so students don't have to search for the assignments, have topical resources gathered already and relevant database tutorials.

Conclusions: Since the launch of the new site early July 2015, the number of frustrated user emails, calls, and chats has diminished. We call that success won from taking extraordinary care when we planned for the use of the library using the big picture concepts.

CISSWeb: Creating an Online System for Storing and Sharing Literature Search Results

Pip Divall
Clinical Librarian Service Manager, Education Centre Library, Leicester, England, United Kingdom

Objectives: To create an online repository, CISSWeb (Clinical Information Search System) of completed searches, summaries and strategies, which emails results directly to requesters and provides a "brand" for the University Hospitals of Leicester Clinical Librarian Service.

Methods: We designed an in-house online database (CISSWeb) which stores literature search requests, and can be used to assign these to librarian responders. The librarian undertakes the literature search, using NHS Healthcare databases and other resources, and uploads the results plus a completed summary of the evidence to CISSWeb. This is then forwarded by CISSWeb to the requester, including our Clinical Librarian Service/Library and information service branding. The system also contains a feedback request form, which gives a star rating for completed requests. We have since identified improvements we would like to make to the system and have engaged an external software development team to redesign CISSWeb and create features that will make it a more powerful and useful system.

Results: CISSWeb allows users of the system to assess whether similar searches have been undertaken before and to utilise previous search strategies in responding to new literature search requests. A new version of the software has been developed, and it is anticipated that the system will be complete and ready for use in Spring 2016. We plan to market CISSWeb as a commercial product to other healthcare libraries.

Conclusions: The CISSWeb system is due to launch the new version of the software in Spring 2016. After complete testing of the software, we aim to market this resource to healthcare libraries.

Creating an Easy-to-Use Searchable, Customizable, Online Bibliography

Jason Reed
Head, Library User Services, Kansas State University Libraries, Manhattan, Kansas

Objectives: Create an online, searchable bibliography for a research association that would highlight their academic achievements. I was recruited for this project because one the board members is a faculty member at my institution and she said "we need someone who finds this exciting". I created the bibliography using a jQuery plugin and Google spreadsheets, both free to use products.

Methods: I was tasked with identifying a method of creating an online bibliography that included no continuing costs, and preferably little up front cost as well. I researched existing products and services, ultimately determining that products and services designed for large library digital collections would not be cost effective or require more technical knowledge and maintenance than this group or I could offer. Instead I found an existing jQuery plugin that allows users to take information from Google spreadsheets and convert it into a searchable table that can be inserted into webpages. The beauty of this approach is that once the jQuery is setup, which requires very basic level coding, the end user can update their bibliography by adding records to the Google spreadsheet, with the jQuery live updating the webpage.

Results: The database has been built using a jQuery script and a Google spreadsheet. With the use of very basic html coding, the database can be customized to include any fields desired by the association. This process is ongoing as the association gets a chance to see what the searchable database looks like live. At the moment the database has the standard citation fields, plus I'm working on adding keywords to the database to allow users to identify articles by subject and not just the terms used in the article titles.

Conclusions: his project demonstrates an easy to create, highly customizable, searchable database of citations that anyone can use. The end result is a database that is searchable across all fields and can be easily updated by anyone with access to Google spreadsheets. The use of this jQuery script provides a free option for creating a searchable, sortable database of citations for any organization that wishes to share information with information seekers. The American Academy of Health Behavior members plan to use the database to both share their work with interested parties, including potential new members, but also allow their members to see what topics other members are interested in so that they can identify potential collaborators.

Information Literacy on the Move: Delivering Library Training to Hospital Staff with a Mobile Computer Lab

Katie D. McLean, AHIP
Librarian Educator BA, LIT, MLIS, Nova Scotia Health Authority, Halifax, Nova Scotia, Canada

Vivien Gorham
Library Technician, Hospital Library Services, Halifax, Nova Scotia, Canada

Katie Quinn
Library Technician, Hospital Library Services, Halifax, Nova Scotia, Canada

Objectives: Reliable access to well-maintained computer labs and a dependable Internet connection can be hard to come by in the hospital environment. With the majority of information resources being online, and local resources failing to support easy access, the library needed a better option for delivering instruction. Would a mobile computer lab maintained by library staff provide a practical solution?

Methods: Library staff researched other libraries using mobile labs and purchased four laptops, an Internet Hotspot, four mice, and a carrying case on wheels. The mobile lab was used for all standard library training over a period of one year. Training attendance, evaluations and overall cost were used to measure the success of the intervention.

Results: Prior to delivery of training events through the mobile lab, clients consistently provided negative feedback regarding the state of hospital computers. One year after mobile lab implementation, negative feedback citing computer issues dropped by over 90%. Numbers of persons trained remained relatively consistent with previous years, at approximately 720 persons, during the first year of mobile lab use. Cost per person trained was about $6.00 in the first year.

Conclusions: Use of a mobile computer lab increased client satisfaction with library training, and was found to be well worth the initial cost. Other hospital libraries may find this approach a worthwhile investment.

Reading Behavior of Graduate Veterinary Students: How Big (or Small) Is Their Picture? RESEARCH

Erin R. B Eldermire
Veterinary Outreach and Scholarly Services Librarian, Cornell University Library, Ithaca, New York

Emma Davies
Senior Lecturer in Veterinary Neurology and Neurosurgery

Objectives: To determine the proportion and sections of scientific papers that are typically read by graduate veterinary students and whether any differences in reading behavior of scientific literature exists between class cohorts.

Methods: A survey was conducted to understand how veterinary students read and synthesize scientific literature. The survey asked students to read a scientific paper and then answer questions relating to the paper, such as which sections they read, how long they spent reading the paper, and details on if and how their reading activities of the paper in the survey generally reflect how they read scientific papers. Students were divided by class cohort (incoming, first-years, second-years, third-years and fourth-years) so that differences in reading behavior by cohort, if any, can be detected. The survey was conducted over the summer of 2015. An incentive to take the survey was included (a random drawing for Amazon gift cards) to increase participation.

Results: 488 graduate veterinary students were invited to participate in the survey and 233 participated. While no difference was detected in the sections of scientific papers that students read, incoming students generally read a significantly higher proportion of scientific paper content than first- and second-years, and they generally enjoy reading scientific papers more than all other cohorts.

Conclusions: This study uncovered an interesting divide between incoming and current students. First- and second-year students are immersed in an intense curriculum in which they are directed to learn a considerable amount of information and to maximize their time and effort they may read a lower proportion of content. This might suggest that students consider information literacy a skill for college rather than a skill for their future veterinary careers. How can the veterinary curriculum improve the information literacy skills of future veterinary professionals?

Utilization of Networking and Profile Platforms by Clinical and Translational Researchers RESEARCH

Amy Suiter
Scholarly Publishing Librarian, Bernard Becker Medical Library, St. Louis, Missouri

Elizabeth Palombo
Evaluation Specialist, Institute of Clinical and Translational Sciences (ICTS), Saint Louis, Missouri

Jae Allen
Associate Business Director, Institute of Clinical & Translational Sciences, Saint Louis, Missouri

Cathy Sarli, AHIP
Senior Librarian for Evaluation and Assessment Services, Bernard Becker Medical Library, Saint Loui, Missouri

Objectives: We performed an initial project to gather information on profile and networking platforms currently utilized by clinical and translational researchers. The objectives of the project included: 1. To understand the current utilization of profile and networking platforms by CTSA members. 2. To identify if CTSA members are associating their ORCID identifiers with their work in Elsevier Scopus.

Methods: The setting of this study was the Clinical and Translational Sciences Award (CTSA) Institute at a school of medicine comprised of 20 academic departments, 11 programs and divisions, and 25 specialized research centers. The CTSA Institute also has partner and affiliate institutions whose researchers may become members. Using CTSA membership roster information, manual searches were performed to determine if members had established a profile or account on Google Scholar, LinkedIn and ResearchGate. In addition, CTSA members were searched for in Elsevier Scopus to determine that they had an ORCID identifier associated with their Scopus profile. Additional resources, such as academic websites, were used to assist in researcher disambiguation. Descriptive statistics were calculated for all of the platforms investigated.

Results: Based on the sample of 1654, 13.7% (n=227) had a Google Scholar profile, 57.1% (n=945) had a LinkedIn profile, and 48.6% (n=804) had a ResearchGate profile. Only 3.7% (n=62) had an ORCID identifier that was associated with their Scopus profile.

Conclusions: CTSA members are encouraged to utilize LinkedIn, Google Scholar and ORCID identifiers. The high percentage of LinkedIn accounts may be a positive finding in terms of promoting members and their work beyond the academic community. The Scopus database is a key tool in locating and evaluating research outputs from our CTSA members, yet few members had an ORCID identifier associated with their Scopus profile. In contrast to the other platforms, the study showed that a high percentage of CTSA members have ResearchGate profiles.

Encouraging members to create and associate their ORCID identifier with their Scopus profile is one way to improve our ability to identify and promote their research outputs. We have a better understanding of the tools being used by our CTSA members and hope to use this information for training and outreach. The study also provided baseline values before additional efforts to promote the use of ORCID identifiers.

Future work may include evaluating the usage of these platforms based on discipline and career stage or investigating the use of other social networking platforms.

Evaluating Electronic Lab Notebooks for Health Sciences Research Laboratories

Franklin D. Sayre
Pharmacy Librarian, Health Sciences Libraries, Minneapolis, Minnesota

Objectives: The objective of this project was to evaluate potential Electronic Lab Notebooks (ELNs) to see if they met the requirements of four independent health science laboratories and to facilitate ELN evaluation and selection. A secondary objective was to establish close working relationships with the laboratories and to learn more about their data management needs.

Methods: Functional requirements for each laboratory were determined using semi-structured interviews with the Primary Investigators and representatives from each laboratory. An initial list of potential ELNs was created through a literature review and web search. Product reviews, trials, and interviews with vendors were used to identify products that met basic requirements. Vendors were then asked to provide in-person or online demonstrations of the ELN for representatives from the laboratories and feedback was collected following these sessions. Finally, each laboratory selected products for full trials in order to identify strengths and weaknesses of each product. It is planned that further interviews will be held with each laboratory that selected an ELN product to see if it is meeting their requirements and in order to evaluate the success of this project and any changes that need to be made to the process.

Reaching out on a Dime: Using Inexpensive Webinar Technology to Reach Faculty

Charlotte Beyer, AHIP
Instruction and Reference Librarian, Boxer Library, North Chicago , Illinois

Objectives: To describe how a library transformed their in-person resource sessions geared towards faculty into a live/on demand webinar series using inexpensive webinar technology in the absence of a campus wide video conferencing solution.

Methods: From 2012-2014, the library partnered with the Office of Faculty Development to provide electronic resource demonstrations directly to university faculty. In 2015, the librarians noticed a lack of participation in these sessions, and with small staff needed a more efficient way to reach these important advocates. Currently, the University does not have a campus wide video conferencing solution,

and purchasing an individual license seemed cost prohibitive. However, the librarians discovered AnyMeeting, which for a minimal cost would allow small webinars with a maximum of 25 participants that could be recorded immediately after completion. Between January to June 2015, the library hosted four webinars during the lunchtime hour that were recorded at their conclusions. The faculty could either participate in the webinars in real time or review the recordings on demand via a LibGuide.

Results: From January - June 2014, the library provided 4 sessions with 14 participants. From January - June 2015 there were 56 participants between the real time participants and those that watched it at later date which indicates a significant increase. One of the other unexpected benefits was the ability to reach out to the clinical faculty, who could benefit from the instruction even though not physically on campus. Faculty commented informally they appreciated they could access the webinars from their desk or on demand when they had a free period.

Conclusions: Overall the webinar format was a success as faculty were exposed to resources they might have not known about before, and it required minimal time from the instruction librarian who could do the sessions at her desk. One of the ways to get the best return on investment is to expose the library's resources to those who directly shape the curriculum, and interact with the students on a daily basis. Instruction using this technology could be a great solution for institutions with small budgets but a big desire for outreach.

Search.bioPreprint: A Tool for Discovering Cutting Edge, Yet-to-Be Published or Reviewed Biomedical Research Articles

Carrie L. Iwema, AHIP
Information Specialist in Molecular Biology, University of Pittsburgh, Pittsburgh, Pennsylvania

John LaDue
Head of Knowledge Integration, Health Sciences Library System, Pittsburgh, Pennsylvania

Ansuman Chattopadhyay
Head of Molecular Biology Information Service, Health Sciences Library System, Pittsburgh, Pennsylvania

Objective: The time it takes for a completed manuscript to be published traditionally can be extremely lengthy. Preprint servers are open access online databases that enable authors (1) to make their research immediately and freely available and (2) to receive commentary and peer review prior to journal submission. We created a search

engine to quickly locate these unindexed articles.

Methods: Article publication delay, which occurs in part due to constraints associated with peer review, can prevent the timely dissemination of critical and actionable data associated with health concerns such as Zika virus. There is a growing movement of preprint advocates who want to change the current journal publication and peer review system, proposing that preprints catalyze biomedical discovery, support career advancement, and improve scientific communication. Until the creation of search.bioPreprint, there has been no simple way to identify biomedical research published in a preprint format, as they are not typically indexed and are only discoverable by directly searching the specific preprint server Web sites. This lightning talk will introduce search.bioPrint and quickly show how easily it can be used for literature searches and systematic reviews to locate grey lit articles that would otherwise fall through the cracks.

Mosaic Collaborations: Research Speed Networking for Clinicians, Engineers, and Scientists

Karen Gau, AHIP
Research and Education Librarian, Tompkins-McCaw Library for the Health Sciences, Richmond, Virginia

Pamela M. Dillon
Research Liaison, Center for Clinical and Translational Research, Richmond, Virginia

Objective: To share our experiences with connecting clinicians, engineers, and scientists through a three-way speed networking event.

Methods: Among our health sciences library's core values are to advance health information research and encourage collaboration within and outside the library profession. To encourage collaborations and advance scientific inquiry at our university, a speed networking event was hosted by our library and our Center for Clinical and Translational Research that specifically targeted clinicians, engineers, and basic scientists. Three-way speed networking was organized instead of the traditional one-on-one in order to maximize interactions and minimize time spent. Twenty-one faculty researchers attended the speed networking event and most of the participants (n=17) felt that they met a potential collaborator through the event; a lunch was held immediately after the speed networking event to encourage follow-up with potential collaborators.

Research Impact and Evaluation Services in the Library: One Piece at a Time

Karen E. Gutzman
Impact and Evaluation Librarian, Galter Health Sciences Library, Chicago, Illinois

Kristi L. Holmes
Library Director, and Associate Professor of Preventive Medicine, Health and Biomedical Informatics Division, Galter Health Sciences Library, Chicago, Illinois

Objectives: Over the past year, our library implemented a cohesive set of services around research evaluation and impact assessment, formally structured in the library as the Metrics and Impact Core (MIC). Many of the services utilize the library's expertise in bibliographic data, which can be mined, analyzed and visualized using a variety of techniques to gain a better understanding research impact.

Methods: This study discusses the operationalization of these services from the standpoint of a modern medical library and presents the partnerships, resources and tools needed to make this vision a reality. We discuss the types of reports and activities we have developed to suit the needs of our stakeholders using bibliographic data, and the challenges and successes we have found along the way.

Results: We found that we often have repeat customers suggesting new projects or requesting updated numbers. Presentations to departments and new faculty, and our close association with our clinical and translational science institute, NUCATS, have created an overall awareness of our services and a synergy that keeps us moving forward.

Conclusions: While still in its infancy and often not broadly available, many libraries are committing resources and staff to support research evaluation and impact assessment work on behalf of their institution. Libraries offer the campus a perfect combination of expertise, perspective, and resources to help support and advise their assessment, evaluation and visualization of research impact across the peer-reviewed literature and beyond.

Library Return-on-Investment: Model for Establishing the Value of a Health Sciences Library in Support of Research RESEARCH

Douglas Varner, AHIP
Senior Associate Director / Chief Biomedical Informationist, Dahlgren Memorial Library, Washington, District of Columbia

Jennifer Kluge
Chief of Staff, Georgetown University, Washington, District of Columbia

Nancy L. Woelfl
Professor Emeritus, McGoogan Library of Medicine, Omaha, Nebraska

Jett McCann
Director and Senior Associate Dean for Knowledge Management, Dahlgren Memorial Library, Washington, District of Columbia

Objectives: Libraries are increasingly required to assess value of services in the global context of importance to users expanding beyond qualitative models to develop quantitative methodologies. This presentation describes library collaboration with Research Administration and research principle investigators to develop a return-on-investment model generating data calculating the amount of grant income earned for every dollar the institution invests in library budgets.

Methods: Dahlgren Memorial Library at Georgetown University Medical Center plans to evaluate and refine the established return-on-investment model developed by Luther and adapted for use in health sciences libraries by Woelfl through modified data collection techniques.
The return-on-investment calculation is derived from compilation of data including approved NIH grant dollars, the number of applications using citations in the reference section of the grant application, the number of citations available via the library collections in the grant application reference section and additional quantitative values to be discussed in the presentation.

Library staff collaborated with a consultant who developed the health sciences library model, the Dean for Research, Research Administration staff and research principle investigators to arrive at a return on investment calculation providing a quantitative measure of the impact of institutional investment in library resources and services on grant income.

Documenting and Demonstrating Policy and Media Impact

Caitlin Bakker
Biomedical/Research Services Liaison, Bio-Med Library, Minneapolis, Minnesota

Objectives: In demonstrating the impact of research, both to institutions and to funding agencies, faculty are increasingly interested in looking beyond evaluative metrics to incorporate impact on policy and media coverage. This talk describes the process of establishing a streamlined system for identifying and reporting diverse forms of research impact.

Methods: Using both subscription-based and publicly available tools, a workflow has been developed to track the impact of grant-funded projects. Through the development of an intake questionnaire, resource-specific

workflows, and report templates, the creation of policy and media impact reports is made scalable and sustainable with limited staffing resources. Strategies for identifying researchers and effectively establishing contact will also be discussed.

Monday, May 16, 2016, 1:00 PM – 2:25 PM
Room: 202B

Session: Creating a Needed Dialogue: A Discussion About Lesbian, Gay, Bisexual, Transgender, Questioning (LGBTQ) Health Librarianship in 2016

Moderator: Blake Hawkins
Blake Hawkins
Martin Morris

Creating a Needed Dialogue: A Discussion About Lesbian, Gay, Bisexual, Transgender, Questioning (LGBTQ) Health Librarianship in 2016

Blake Hawkins
Graduate Student, Master's of Library and Information Studies, Vancouver, British Columbia, Canada

Blake Hawkins
Graduate Student, Master's of Library and Information Studies, Vancouver, British Columbia, Canada

Martin Morris
Liaison Librarian: Life Sciences, McGill University, Montréal, Quebec, Canada

Tony Nguyen, AHIP
Access/Communications Coordinator, National Network of Libraries of Medicine, Southeastern/Atlantic Region, Baltimore, Maryland

Emily Vardell
PhD Candidate and Teaching Fellow, University of North Carolina at Chapel Hill, Chapel Hill, North Carolina

John Siegel
Student Success Librarian, University of Arkansas at Little Rock, Little Rock, Arkansas

D. Ryan Dyck

The scholarly literature of health librarianship contains two mentions of the information needs and information-seeking behaviours of LGBTQ (Lesbian, Gay, Bisexual, Transgendered, and Queer) health professionals - a 2004 JMLA article, and two presentations in 2013/2014 at CHLA and IFLA of a related research project conducted by one of the proposers of this session (a journal article is currently undergoing peer review). Meanwhile, the broader literatures of academic and public librarianship contain various studies into the needs and attitudes of our LGBTQ patrons. At a time when attitudes towards LGBTQ people are changing, and where greater attention is being paid to their specific health needs, we believe that the time has come for our profession to initiate a dialogue into how we respond to these needs.We propose a session to initiate a dialogue on how health librarianship should best respond to the distinct needs of our LGBTQ patrons. This session would include interested health librarians and community members, and discuss the current issues in LGBTQ health information, information needs of our LGBTQ patrons, and how health librarians can develop and improve our practice within this context. Panel members would give lightning talks about a particular issues related to LGBT health and health librarianship. A subsequent moderated discussion would develop these themes, which we hope would be further developed after the conference. The session would be at an introductory level, in order to make it welcoming for both LGBTQ and non-LGBTQ health librarians who may be interested in attending.

Monday, May 16, 2016, 1:00 PM – 2:25 PM
Room: 104C

Session: Beyond the Search: Expanding the Role of the Librarian in the Systematic Review Process

Moderator: Marie T. Ascher

Beyond the Search: Expanding the Role of the Librarian in the Systematic Review Process

Marie T. Ascher
Lillian Hetrick Huber Endowed Director, Health Sciences Library - New York Medical College, Valhalla, New York

Margaret J. Foster, AHIP
Systematic Reviews and Research Coordinator/ Associate Professor, Texas A&M University, College Station, Texas

Mark MacEachern
Informationist, Taubman Health Sciences Library, Ann Arbor, Michigan

Whitney A. Townsend
Informationist, UM Library, Ann Arbor, Michigan

Librarians are increasingly involved with systematic reviews, most frequently as creators of the exhaustive search strategy as recommended by the Institute of Medicine guidelines, and also as organizers, archivers, and writers of the search method component. In this session a distinguished panel of systematic review experts will present strategies and tools for expanding the role of the librarian "beyond the search," to the project and data management aspects of the next steps during systematic review process- selection, appraisal, and data extraction. This session will dispel the notion that librarians are only suited for the search component of the systematic review process by describing some of the methods to select and assess studies, extract data, and present results.. Topics that will be covered include:

- Overall systematic review process

- Quality assurance through project management principles

- Screening and selection of studies, including how to calculate inter-rater agreement

- Risk of bias assessment tools, focusing on validated tools

- Data extraction tools and techniques

- Presentation of results and archiving data

Participants will emerge from this session with an increased understanding of these components with confidence to move forward to expanding their roles in the overall systematic review process.

Monday, May 16, 2016, 1:00 PM – 2:25 PM
Room: 202D

Session: Public Health Support

Moderator: Vicky Duncan, AHIP

State Libraries and the State Public Health Workforce: Exploring Services and Information Access RESEARCH

Robert M. Shapiro, II.
Public Health Librarian, Chandler Medical Center Library, Lexington, Kentucky

Teresa M. McGinley
Graduate Student Intern, UK Medical Center Library, Lexington, Kentucky

Matthew N. Noe
Graduate Student, Medical Center Library, Lexington, Kentucky

Jesse M. Fagan
Doctoral Candidate, Management Department, Gatton College of Business and Economics, Lexington, Kentucky

Objectives: Despite a long history of attempts to provide access to information to the public health workforce, there still remains a great deal of uncertainty regarding how to approach information access, promotion, and use. This study investigates the resources and services that state libraries provide to state health department employees.

Methods: The project proceeded in two phases. During phase one, state library websites were manually scraped by three project staff for the following: access to core databases, reference/research service, interlibrary loan, specialized access/services for state public health employees, and other services or training. In phase two a survey was distributed to the directors of each state library. The survey supplemented phase one findings, by determining service usage frequencies and identifying what additional services are provided. Responses were collected using Qualtrics and analyzed using Microsoft Excel and the statistical software, R.

Results: Phase one data indicate 92% of state libraries (n=46) provide access to databases core to public health; 86% (n=43) provide explicit references

services; 90% (n=45) provide interlibrary loan to state employees. The survey had a 58% overall response rate. Of the respondents 60% (n=18) reported providing access to databases targeted to health professionals, and 73% (n=22) reported providing full-text access to content. Although 87% of respondents reported providing training, only 19% stated that they offer instruction targeted to health professionals. Almost 97% of respondents reported providing ILL, with only 3% being fee-based.

Conclusions: Results indicate most state libraries not only provide access to core databases, but additional services such as free interlibrary loan, tailored instructional sessions, and reference consultations. Furthermore, many state libraries are providing services and access specifically tailored for state public health employees. Medical libraries have a proven history in providing information access and services to the public health sector; however, they may be duplicating efforts already put forth by state governments.

Connecting Two Worlds: Expanded Roles for Librarians in Disasters: Has It Worked?

Elizabeth F. Norton
Technical Information Specialist, Disaster Information Management Research Center Specialized Information Services U.S. Library of Medicine, Bethesda, Maryland

Cynthia B. Love
Tecnnical Information Specialist, Disaster Information Management Research Center, Bethesda, Maryland

Objectives: To determine if funding support provided to libraries and librarians to work in collaboration with organizations with disaster-related responsibilities has had a sustainable effect on integrating librarians into the disaster workforce and increasing the awareness of authoritative disaster health information resources.

Methods: Seventeen collaborations between libraries and disaster-related organizations were funded between 2011-2014 with the goal of improving disaster medicine and public health information access for the disaster workforce and promoting new and creative collaborations on disaster health information needs among and to the mutual benefit of librarians and the disaster workforce. To determine if this program has had any sustainable impact on the awareness and use of disaster health information resources and the creation of new roles for librarians, the authors conducted an evaluation of the program using document review and a structured telephone interview process with the representatives from the funded projects. Documents and notes were imported

into qualitative research software for analysis. The evaluation results were used to assess the effectiveness of the funding and to identify ways to improve the process for future applicants. .

Results: Twenty-two interviewees representing 12 collaborations were asked ten specific research questions. The results culminated in recommendations in the following areas: application process, partnership and activities, change in knowledge/access/use, and sustainability/integration.

Conclusions: Funding for collaborations between libraries and disaster-related organizations provided unique and successful ways for these two groups to connect and share expertise. All participants interviewed recognized the value of each other's roles in supporting disaster planning and response and a number of interesting and unique relationships were developed between partner organizations. Most notably, disaster and emergency workers discovered the benefits that librarians could bring to the table, something many had not been aware of before the collaborative project. Librarians discovered that in addition to teaching their partners about disaster information tools and resources, they could play expanded roles in helping their communities or organizations in times of crisis.

Monday, May 16, 2016, 1:00 PM – 2:25 PM
Room: 201F

Session: Outside of the Box Services and Instruction

Moderator: Rebecca O. Davis

Digital Natives' Dilemma: Using Social Media While Keeping It Professional RESEARCH

Gisela Butera
Reference and Instructional Librarian, Himmelfarb Health Sciences Library, Washington, District of Columbia

Alexandra Gomes, AHIP
Associate Director for Education, Information, and Technology Services, The George Washington University, Washington, District of Columbia

Terry Kind
Assistant Dean of Clinical Education, Associate Professor of Pediatrics, Pediatrics, Washington, District of Columbia

Katherine Chretien
Dean for Clinical Education, Associate Professor of Medicine, Department of Medicine, Washington, District of Columbia

Objectives: To measure the impact of the Social Media and e-Professionalism session, attended by first-year George Washington University medical students. The session focused on exploring their attitudes and beliefs toward health professionals using social media, while discussing potential behavior to avoid as well as opportunities to enhance their future medical careers.

Methods: As digital natives, first-year medical students need to reflect upon appropriate/inappropriate methods of integrating social media with their new professional identities. An IRB study was conducted from 2012-2014 on Himmelfarb Library's instructional/educational session on social media and e-professionalism, co-taught by a librarian and medical faculty using active learning methodology followed by a panel discussion led by faculty mentors and an attorney. The session provided guidance and discussion on avoiding

risky behaviors using while encouraging students' to explore opportunities through social media. A post-session assignment asked students to reflect on the impact of the session. The study team qualitatively analyzed and coded the reflection papers and a follow-up quantitative Likert scale survey was sent to the 2014 cohort to evaluate the longer-term impact. The session has been revised as an interprofessional session involving both medical and physician assistant students.

Results: Short term results from reflection assignments showed 60% (2012), 44% (2013) and 57% (2014) planned to make changes to their social media accounts such as privacy settings, removal of potentially damaging photos/content or greater participation (e.g. reading blogs, Twitter accounts). Over sharing, and generational viewpoints differences were identified as challenges in using social media. A post-session survey of cohort 2014 six months later showed 42% opted to avoid potentially harmful behaviors that might impact their career and/or patients, while 12% indicated engaging in potentially positive beneficial social media behaviors helpful for their career, getting into residency program, and the public (e.g. following health professional's Twitter feeds, or posting health-related information). Sixty-three percent of the respondents felt it was extremely important for students to learn about e-professionalism, and 96% of the group thought this should occur during first year of medical school.

Conclusions: The short term results of the session from all cohorts showed an impact in raising students' awareness, and the 2014 post-session results indicate a long-term effect in changing students' social media awareness and behavior. One of the key objectives of the session was to demonstrate positive use of social media by medical professionals and it seems clear that this message began to resonate with the students.

Fact or Fiction: Understanding and Using Fair Use and Copyright

John D. Jones, Jr.
Instruction & Curriculum Librarian | Associate Professor | MSIS, University of Colorado Anschutz Medical Campus, Aurora, Colorado

Ben Harnke
Education & Reference Librarian, Health Sciences Library/Education and Reference, Aurora, Colorado

Objectives: With all of the different materials and opinions concerning Fair Use & Copyright available to health sciences professors, our main purpose was to create a 60 minute education session to help sort fact from fiction so faculty

would be more comfortable and confidant with their use of course materials.

Methods: Our goal was to present a series of brief situations and have participants discuss their knowledge and understanding of copyright and fair use for these scenarios. Each vignette was followed with a current best practice suggestion. Potential solutions included things like linking to online articles, uploading to secure course management systems, making freely available via the web but overall by always assessing risk using a Fair Use evaluation document.
An education session survey instrument was used to capture feedback concerning the presentation and identify participants who might need or want individualized follow-up concerning Fair Use & Copyright. Our secondary purpose was share our slides and materials with other health sciences librarians to adapt and reuse for similar purpose at their institutions.

Results: The results will be reported at the 2016 Medical Library Association meeting in Toronto. The classes are scheduled for February 18th, March 17th and April 7th. We will report on the success or failure of these 3 classes including information from the class evaluation.

Conclusions: We will report our conclusions at the 2016 Medical Library Association meeting in Toronto. We anticipate that participants will be unfamiliar with a Fair Use Checklist and also that some of their current practices may flirt with edge of Fair Use or may be a copyright violation. We hope the participants will also help identify unique situations to be considered and incorporated in subsequent classes.

Creating a Mosaic of History Lectures for the Health Sciences

Sandra L. Bandy, AHIP
Chair, Content Management, Robert B. Greenblatt, M.D. Library, Augusta, Georgia

Renee Sharrock
Curator, Historical Collections and Archives, Robert B. Greenblatt, M.D. Library, Augusta, Georgia

Objectives: Unlike many health sciences libraries, our library has a large and far-reaching Historical Collection and Archives (HCA) room housing hidden treasures. Showcasing this collection, and the history of the health sciences, has often been a challenge. This paper examines the development and implementation of a History of the Health Sciences Lecture Series.

Methods: The recent addition of historical donations from alumni and other health professionals has resulted in an increase interest in the library's historical collections and archives. Making these collections discoverable is the primary goal. The library hosted a mosaic of lectures that focused on the library's historical collections. Lectures have been tied to current library events and university courses. Creating this historical lecture series is a collaborative planning process which included many obstacles and creative solutions. Steps in this process include: (1) connecting historical collections with faculty or alumni to design the lecture; (2) developing marketing strategies across the health science campus that encourage attendance and interest; and (3) assessing the effectiveness of the lecture series.

Bringing the Writing Center to the Library: Report of a Pilot Project

Terry Ann Jankowski, AHIP, FMLA
Assistant Director for User Experience, University of Washington, Seattle, Washington

Jamie M. Gray, AHIP
Director for Research & Instruction, Lane Medical Library & Knowledge Management Center, Stanford, California

Lilly Campbell
Assistant Director, Odegaard Writing & Research Center, Seattle, Washington

Objectives: The goal of the project was to expand writing support for students, faculty and staff in the health sciences at the University of Washington..

Methods: The undergraduate library has provided writing support by peer tutors for ten years. Although open to all, the 10-minute walk for services has proven to be a significant barrier in their utilization by health sciences students. Another barrier is the perception that peer tutors lack sufficient skills to support the scientific writer. In 2015, the UW health sciences library conducted a pilot project designed to expand writing support for students, faculty and staff in the health sciences with appointments at the Health Sciences Library. By partnering with the writing center we felt we could better support the development of writing skills in disciplines that may not traditionally be viewed as writing-intensive. Additionally the pilot included educational opportunities for the peer tutors. This presentation describes the steps taken to implement and evaluate the project as well as describe its benefits and lessons learned.

Results: Tutors were hired and space identified for the service. Opportunities

were provided for tutors (and librarians) to increase their knowledge of support for writing in the sciences. Health sciences students took advantage of the sessions offered on site.

Conclusions: Adding this service to our portfolio was well received and should be continued. Next steps might include exploration of supporting distance students.

Mobile Game Development Program to Support Health and Science Information Provision to Adolescents

Ying Sun
Computer Scientist, NLM, Bethesda, Maryland

Karen Matzkin
Contractor, NLM, Bethesda, Maryland

Judy Kramer
Contractor, NLM, Bethesda, Maryland

Andrew Plumer
Outreach Librarian, NLM, Bethesda, Maryland

Alla Keselman
Senior Social Science Analyst, Specialized Information Services, Bethesda, Maryland

Objectives: Helping teachers find quality information that links biology, chemistry, and environmental science to human health is extremely important and very challenging. One emergent way to deliver information in a way that is both engaging and conducive to learning is through educational gaming. The program described here develops games supporting health and science information provision to adolescents in the science classroom.

Methods: This program, conducted by a K-12 team of a federal medical library, is collaboration among librarians, science teachers, computer scientists, and gaming researchers. The K-12 team maintains several websites that collect and organize classroom resources in environmental health, toxicology, and genetics. Teacher feedback on the sites frequently asked to add games. As few quality games could be found, the team decided to start a games development program, linking curriculum requirements with the resources on the team's sites. To date, four iOS games were built, two of which are already available free-of-charge through App Store. The games teach about toxic chemicals in the environment, DNA base pairings, and Bohr Model of the atom. Development

process involves curriculum review and consultations with teachers and gaming experts, programming in Swift, and focus groups with students and teachers. Future evaluations will include classroom testing.

Monday, May 16, 2016, 1:00 PM – 2:25 PM
Room: 203B

Session: Focus on Data Management

Moderator: Jamie Saragossi

BD2K: Big Data to Knowledge and the Library

Jacqueline Wirz
Director of Career & Professional Development and Graduate Student Affairs, Research Data Ninja, Assistant Professor, Oregon Health & Science University, Portland, Oregon

Objectives: The NIH Big Data to Knowledge (BD2K) initiative aims to enable research, to facilitate discovery and support knowledge, and to maximize community engagement. The Oregon Health & Science University Library has partnered with our Department of Medical Informatics and Clinical Epidemiology on BD2K initiatives to develop workshops and online educational materials to teach major concepts in biomedical big data science.

Methods:OHSU currently has two BD2K grants: 1) to develop a suite of online educational resources (OERs) and 2) to develop workshops for trainees on big data concepts. These grants involve library researchers, information specialists and instructional designers working closely with primary investigators from our informatics department. Our final products will include several iterations of an in-person workshop designed for trainees as well as a comprehensive educational resource library. This presentation will cover highlights of the projects and include information on how libraries can use these resources in their communities.

Author Behavior and Attitudes Toward the PLOS Data Availability Policy RESEARCH

Kimberly Powell
Life Sciences Informationist, Woodruff Health Sciences Center Library, Atlanta, Georgia

Jennifer Doty
Research Data Librarian, Robert W. Woodruff Library, Atlanta, Georgia

Jeremy Kupsco
Research Informationist, Woodruff Health Sciences Center Library, Atlanta, Georgia

Objectives: In 2014, PLOS journals implemented a data availability policy requiring authors to provide a reliable location for underlying data. The recommended method is to deposit data in an appropriate public repository. This study aims to determine how our institution's researchers have responded to the PLOS requirement, utilized library data management services, and perceived specific challenges to making data reliably available.

Methods: To determine changes in publication and data sharing preferences, institutional publications in PLOS journals were examined during the twelve months following the policy change. Three librarians reviewed 126 articles published since the policy to determine if the data statement conformed to recommended guidelines and allowed the reader to reliably locate referenced data. Findings were compared against library data management service requests.

Next, 84 institutional authors were surveyed for their attitudes and data sharing processes. The survey was administered to researchers who submitted to PLOS only after the data requirement went into effect.

Results: A review of articles from the first year of the PLoS data policy revealed the majority report "relevant data included in the paper and supplementary materials" (~43%), while only 10% deposited data into a repository. When reviewed for conformity to policy standards and actual data discoverability, claims for "data available from a 3rd party" and "data available within manuscript" scored the worst for providing full data citations and actual data availability, respectively. Conversely, depositing data in a repository had the best score for data discoverability.

A survey revealed the data policy did not affect the amount of raw data included in the manuscripts; with over 70% of authors saying they did not add additional data. However, approximately 30% of researchers stated the policy added extra work to manuscript preparation and approximately 20% reported they were contacted to add more data to the paper. Finally, 19-23% reported they would not publish in the PLOS family of journals in the future due to the data policy.

Conclusions: Despite the implementation of a data availability policy, three information professionals who reviewed 126 PLOS data availability statements found many articles did not provide sufficient information for reliable data accessibility. Likewise, over 70% of authors reported making no change to the amount of data included in their publications. However, due to the policy, approximately 20% of respondents would not publish in PLOS journals

again. These observations represent a clear need for data management outreach and potential areas for library services.

Open Data, Big Health: Global Open Data for Health Care Research RESEARCH

Charles J. Greenberg, AHIP
University Library Director, Wenzhou-Kean University, Wenzhou, Zhejiang Province, N/A, China

Sangeeta Narang
Librarian Grade-I, National Drug Dependence Treatment Centre Library, New Delhi, India

Objectives: In 2007, open government advocates issued a set of eight principles of open data. Open health data has implications for clinical care and research, drug discovery and development, public health and health policy at regional, national, and global levels. Where is the global leadership in the community of nations on implementing and unlocking the benefits of open health data?

Methods: Using the W.H.O list of member states (n=194), an inspection of web sites was conducted to identify and record the presence of open health data or initiatives. With each country, an attempt was made to locate the following types of repositories on an official government web page: a Ministry of Health or equivalent government agency; a conspicuous link on a government web page to open health data; Additional national government health web sites, with or without data; national government-sponsored open data repositories, not necessarily health; unique attributes of national health data web sites or conspicuous absences; and adherence to the principles of open government data, for health data. Google tools provided efficient ways to collect and store research results. A shared Google Sheets™ page allowed both authors to work at independently, while being able to observe work in progress.

Results:Open health data is easily discoverable though links on a website or a separate open data collection in less than one-third of the W.H.O. member nations in a search done by two experienced information researchers. 12 nations demonstrate the principle to provide comprehensive open data. In most of these nations the power of electronic health record (EHR) systems to provide primary exportable data that can at the same time protect the privacy of individualized patients is a key to their accomplishments. Only 14 nations distribute primary, non-aggregated health data. Nearly 25% of the W.H.O. member states investigated are providing some health data in a non-proprietary format such as CSV. The sixth, seventh, and eighth open government data principles, representing universal access, non-proprietary formats, and non-

patent protection, are observed for health data in about one-third of the W.H.O. member states.

Conclusions: While there was ample examples of exceptionally well organized national open health data distributors, there was no more than a one-third minority of the world's nations with principled, organized portals set up to systematically share open data. At least 15 countries do not even have a health ministry representation online. Our complete Google Sheets data is available at: https://goo.gl/Kwj7mb.

Framing the Role of the Librarian in Big Data: A Multifaceted Approach to Encouraging the Use of National Institutes of Health-Supported Common Data Elements (CDEs)

Joanna L. Karpinski-Widzer
Systems Librarian, Web and Information Management Unit, Public Services Division, Bethesda, Maryland

Liz A. Amos
Librarian, National Information Center for Health Services Research and Health Care Technology, Bethesda, Maryland

Objectives: Our paper highlights current efforts of National Library of Medicine librarians involved in the Trans-BioMedical Informatics Coordinating Committee Working Group on Common Data Elements. This includes tools and resources to support the needs of potential NIH-funded investigators and engage biomedical science librarians in facilitating usage of common data elements.

Methods: As Big Data becomes increasingly important to scientific research, NIH created a working group to develop resources for and encourage the use of Common Data Elements (CDEs) in NIH funded research. A "Common Data Element" is a precisely defined question (variable) with a specific answer or set of answers to that question (response), common to multiple datasets or across different studies. CDEs facilitate the standardized collection of data in research studies, promoting interoperability between different systems, supporting consistency and quality of data collection. Librarians participated in the group by organizing information about the use CDEs. Librarians assisted in developing information resources to aid in the organization, collection, identification and curation of CDEs and associated initiatives. To increase awareness and facilitate standardized use of NIH CDEs across institutions, librarians describe their efforts in tracking publications and developing educational and outreach materials.

Developing Metadata for the National Institutes of Health (NIH) Data Discovery Index: One Librarian's Experience Working with the NIH BioCADDIE Initiative

Kevin B. Read
Knowledge Management Librarian, NYU Health Sciences Library, New York, New York

bioCADDIE Metadata Working Group
bioCADDIE Metadata Working Group, bioCADDIE, La Jolla, California

Objectives: In a national effort to make datasets more discoverable to promote transparency and reproducibility in research, a librarian working collaboratively as part of a working group for the NIH bioCADDIE initiative developed a comprehensive metadata schema for the NIH Data Discovery Index (NIH DDI) prototype. The NIH DDI will describe and index research datasets, just as PubMed describes biomedical literature.

Methods: Use cases were developed to serve as a guide to define what metadata to include in the NIH DDI, as well as the level of granularity at which datasets should be described. A list of data discovery and metadata initiatives were compiled and reviewed to identify the initial set of metadata elements. A list of metamodels -- the structure for how metadata elements relate to one another -- were also analyzed to evaluate their suitability for the NIH DDI. General and life science-specific metadata schemas, including one developed by the librarian author of this paper, were mapped to XML and linked open data schemas (RDF/OWL) to identify relationships between related elements. Selected metadata elements and their mappings were then compiled into a comprehensive list that warranted inclusion into the final NIH DDI prototype metadata schema.

Results:At the time of writing this abstract, a first iteration of the NIH DDI metadata schema has been developed relevant to the use cases described in the Methods section. The metadata elements are comprehensive in nature and provide descriptive information at the dataset level, the study level, as well as at the activity level (e.g. experiment that was performed to acquire the dataset). Furthermore this metadata schema will serve to link datasets to their associated publications in PubMed, the study and grant they were derived from, and the repository or location where they are stored.

Conclusions: This project demonstrates the importance of having a librarian work collaboratively with high profile stakeholders on a large scale, national initiative to develop metadata to describe NIH-funded research datasets. Bringing a librarian's knowledge of metadata schemas and information

discovery to this project was beneficial for helping biomedical researchers and leadership gain a better understanding of how datasets can, and should be described. The NIH DDI will play a significant role in the work of health sciences librarians, as it will serve as a comprehensive database of NIH-funded research datasets, just as PubMed serves as the premier database for biomedical literature.

Monday, May 16, 2016, 1:00 PM – 2:25 PM
Room: 205B

Session: Our Role and Impact on Increasing Patient Safety and Reducing Readmissions in the Hospital Setting

Moderator: Mary Katherine Haver, AHIP

Our Role and Impact on Increasing Patient Safety and Reducing Readmissions in the Hospital Setting

Karen L. Keller
Director Library Services and Health Literacy, Edwin G. Schwarz Health Sciences Library, Fort Worth, Texas

Tara Douglas-Williams
Division Head for Information Services, Library Manager, M. Delmar Edwards, M.D., Library, Atlanta, Georgia

Judy C. Stribling, AHIP
Assistant Librarian, Manager Myra Mahon Patient Resource Center, Weill Cornell Medical College, New York, New York

Sarah Sutton
Clinical Librarian, University Hospitals of Leicester, Market Harborough, N/A, United Kingdom

A panel discussion followed by an open discussion on health sciences librarian's roles within the health care setting to increase patient safety and decrease readmissions. The changing times of decreased reimbursements (or no reimbursement) based on avoidable readmissions, meaningful use requirements, and well-informed consumers who can select the hospital of their choice based on information found on the Internet, the health sciences librarian's professional role of supporting their institutions is an opportunity waiting to be taken. A panel of representatives from the Consumer and Patient Health Information Section (CAPHIS), Hospital Library Section, International Clinical Librarians Conference (ICLC), Leadership and Management Section will

start the dialog on how we can stop talking about our potential return on investment for our institutions and can become valuable team members.

Monday, May 16, 2016, 1:00 PM – 2:25 PM
Room: 203D

Session: Precision Medicine: Individual Insight into the Bigger Picture

Moderator: Jennifer Walker

Precision Medicine: Individual Insight into the Bigger Picture

Francis Ouellette
Senior Scientist, and Associate Director, Informatics & Biocomputing, Associate Professor, Cell and System Builogy, University of Toronto, Toronto, Ontario, Canada

United States' President Obama announced a Precision Medicine Initiative in January 2015. Described as a bold new research effort, it will revolutionize the treatment of disease and provide clinicians with the tools and knowledge to support a new model for patient care and move away from a "one-size-fits-all-approach." Precision medicine aims to provide clinicians with tools to better understand the complex mechanisms underlying a patient's health, disease, or condition, and to better predict which treatments will be most effective.

Precision medicine is often used to describe how genetic information about a person's disease is used to diagnose or treat their disease. The pursuit of understanding the genetic changes that occur in cancer cells will drive and accelerate more effective treatment regimes, tailored to the genetic profile of the cancer patient. Advances in the sequencing of the human genome; improved technologies for biomedical analysis; and new tools for using large datasets have made it an ideal time to unveil this far-reaching initiative. With the anticipated proliferation of this new research, how can librarians position themselves to facilitate information access to all interested parties (public, patients, regulatory agencies, 3rd party payers, employers, research community) on this topic as well as continue to contribute to the advancement of patient health care?

Join us for an overview of precision medicine and how it is changing cancer research and ultimately the fight against cancer. Gain insights from our invited speaker from the Ontario Institute for Cancer Research in how collaboration,

data sharing and research is collected and made available to interested parties.

This special content session is co-sponsored by the Cancer Librarians Section and the Translational Sciences Collaboration SIG.

Monday, May 16, 2016, 5:00 PM – 6:25 PM
Room: 104C

Session: Tech Trends

Moderator: Emily J. Hurst, AHIP

An Informatics Lab: A Collaborative Space for Innovation

Tiffany Grant
Research Informationist, University of Cincinnati, Health Sciences Library, Cincinnati, Ohio

LibAnalytics for Collecting Library Reference Statistics

Sharon Bailey
Librarian, CAMH Library, Toronto, Ontario, Canada

Sarah Bonato
Reference and Research Librarian, CAMH Library Services, Toronto, Ontario, Canada

Olark Chat: A New Tool for an Old Service

Janis Brown, AHIP
Associate Director, Systems & Information Technology, USC, Los Angeles, California

Designing an App for Clinical Rotations

Harold Bright IV, AHIP
Electronic Resources Librarian, A.T. Still University of Health Sciences, Mesa, Arizona

Creating a Conceptual Search Tool: Why It Was Needed and How It Was Achieved

Lynne M. Fox
Consulting and Training Librarian, Quertle, LLC, Henderson, Nevada

Jeffrey D. Saffer
President, Quertle, LLC

Vicki L. Burnett
Executive Vice President, Quertle, LLC

Tuesday, May 17, 2016, 3:00 PM – 4:25 PM
Room: 201D

Session: Systematic Review Methodology

Moderator: April J. Schweikhard

Knowing When to Stop: Final Results versus Work Involved in Systematic Review Database Searching RESEARCH

Rachael Posey
Pharmacy Librarian, Health Sciences Library, Raleigh, North Carolina

Jennifer Walker
Cancer Information Librarian, Health Sciences Library, Chapel Hill, North Carolina

Karen E. Crowell
Clinical Information Specialist, Health Sciences Library/UNC Chapel Hill, Chapel Hill, North Carolina

Objectives: To examine the relationship between the amount of work involved for the systematic review (SR) team for each additional database searched in an SR versus the number of unique articles included in the final review that were retrieved from each additional database searched.

Methods: We reviewed SRs and meta-analyses published in JAMA, Lancet, and Annals of Internal Medicine from January 2012 to December 2015, selecting those journals as high impact factor journals that have published over more than 50 SRs per year since 2012. We required that all papers in our analysis report a complete search strategy, with the total number of citations retrieved from each database and a complete list of articles included in the final analysis. We then used lists of included journals from each database to determine in which database(s) each of the included articles were indexed, and we calculated the percentage of articles included in each SR that could be located by searching each database. We then compared the number of results added by searching each additional database to the total number of papers added to the review phase to estimate the amount of work required for each additional paper identified.

Results: Ninety-seven SRs met our inclusion criteria. These SRs included an average of 48.13 journal articles and searched an average of 4.43 databases each. Of these, the journal articles included in 16 SRs could all be found in one database; the articles included in 58 SRs could all be found in two databases. For 20 SRs, all included articles could be found in three databases, and the remaining three SRs included articles that could be found in four databases. For 96 SRs, over 90% of articles could be found in two databases.

Conclusions: In total, 99% of articles included in each SR were found in two databases, with the majority being found in PubMed/MEDLINE, Embase, or Cochrane. SRs that found articles in three or more databases screened an additional 923 records in order to find one additional included article, plus an additional 2410 records from databases that did not return any additional included articles, adding an average of 756 hours of work to each SR. SRs for which articles were found in four databases screened an average of 1440 records in order to find one additional included article, plus an additional 8963 records from databases that did not return any additional included articles.

A Biomedical Taxonomy of Study Designs and Publication Types: A Resource for Information Professionals Who Support Systematic Review Teams RESEARCH

Tanja Bekhuis, AHIP
Assistant Professor, University of Pittsburgh School of Medicine, Pittsburgh, Pennsylvania

Eugene Tseytlin
Systems Developer, Department of Biomedical Informatics, Pittsburgh, Pennsylvania

Ashleigh Faith
Adjunct Faculty, School of Information Sciences, Pittsburgh, Pennsylvania

Faina Linkov
Associate Professor, Department of Obstetrics, Gynecology, and Reproductive Sciences, Pittsburgh, Pennsylvania

Objectives: A comprehensive taxonomy of study designs and publication types is an essential component of software currently under development for systematic reviewers. It is also useful for information professionals because it collects terms and variants in one resource. The objective of this study is to improve coverage of study designs in the EDDA Study Designs and Publications

taxonomy. We hypothesize that the process can be semi-automated with minimal human curation to ensure scalability and maintenance.

Methods: In this text mining study, we extracted phrases from two major controlled vocabularies (MeSH and NCI Thesaurus) and from 9000 titles in the HTA Database Canadian, an international repository of records for health technology assessments. We also added Sandieson's manually curated list of 80 synonyms for research synthesis. Eligible phrases included one of the following words or their variants: study, design, trial, assessment, evaluation, survey, or model. After preprocessing, we dropped specific studies by filtering out phrases that mentioned locations, names, acronyms, diagnoses, organs, and designs with semantic types other than research activity. Two methodologists independently screened terms; agreed-upon, eligible terms were added to the taxonomy. Lexical rules guided automated placement to enrich the current taxonomy; two investigators checked term placement for consistency. The improved taxonomy is publicly available on the Web in several file formats.

Results: We automatically extracted and processed 2500 terms from 4 resources. After de-duplicating processed terms in a stepwise fashion across datasets, we screened 1455 terms. The percentage overall agreement for screening judgments ranged from 82% to 92%; agreement for eligible terms ranged from 87% to 95%; for ineligible terms from 63% to 72%. In a final step, we extracted synonyms from the NCI Metathesaurus for agreed-upon, eligible terms. In total, we added 1597 new study design terms to the EDDA taxonomy of which 380 represent new concepts (1.02-fold and 1.75-fold increase, respectively).

Conclusions: Text mining and natural language processing can identify phrases for study designs, synonyms, and variants in controlled vocabularies and other knowledge resources. Further, the semi-automated process with minimal human curation enables enrichment of the taxonomy. The procedures we developed ensure scalability and maintenance of the EDDA Study Designs and Publications taxonomy for information professionals who support systematic reviewers.

The Added Value of Multiple Databases in Searching for Exhaustiveness: A Prospective Study RESEARCH

Wichor M. Bramer
Biomedical Information Specialist, Erasmus MC - Medical Library, Gouda, N/A, Netherlands

Background
When searching for a systematic review (SR) it is accepted that multiple databases should be searched. However searching multiple databases with varying thesaurus terms and syntax is complex and time-consuming. We tried to prioritize which databases should be searched, and see how much the relative recall of different database combinations is.
Methods: Librarian-mediated searches for hundreds of systematic reviews were performed combining several databases, among others: Medline Ovid,Embase.com, Cochrane Central, Web of Science, Scopus, PubMed the subset as supplied by publisher and Google Scholar. The results of these searches were saved in EndNote. When included references are known, these were searched in the EndNote file. For various database combinations and relevance ranking cut-offs we calculated the relative recall.
Results: Of a total 2090 included references from 61 SRs, 335 were retrieved by only one database. Forty percent of the unique references were found in Embase, 20% in Medline, 19% in Web-of-Science, and 13% in Google Scholar. PubMed added 4%, but other databases contributed only very small amounts. A database considered a very important gold standard for SR searches, Cochrane central , did not retrieve any unique included reference.
A combination of Embase, Medline and Web-of-Science retrieved overall 97% of all included references. However, an individual systematic review still had a chance of 33% that the recall for that review is unacceptable (below 95%). An additional search in Google Scholar reduced that chance to 13%. When searching PubMed as supplied by publisher additionally, there was a probability of 92% that all included references have been retrieved.
Depending on the preferred minimal relative recall certain database combinations can be good enough. Using cut off values for relevance ranking does not provide satisfactory results for SRs, but results are promising for non-systematic reviews. Of a random set of 200 reviews in PubMed it is estimated that only 41% of these had an acceptable recall (>95%) based on their database choices.
Conclusion
The recommendation of the Cochrane handbook to search in Embase, Medline and Cochrane central is not sufficient for most systematic reviews. Cochrane does not identify unique references, and the combination of Embase and Medline results in reasonably acceptable recall (>90%) for only 74% of all search strategies. More databases are needed, especially Web-of-Science, but additional value is found in Google Scholar and PubMed as supplied by publisher. Without Embase the risk of unacceptable recall is very high.

What Information Resources Are Searched to Prepare Systematic Reviews of Economic Evaluations in Health Care? RESEARCH

Julie M. Glanville
Associate Director, York Health Economics Consortium Ltd, York, N/A, United Kingdom

Hannah Wood
Information Specialist, York Health Economics Consortium Ltd, York

Mick Arber
Information Specialist, York Health Economics Consortium Ltd, York, United Kingdom

Objectives: Healthcare decision-makers require timely assessment of cost-effectiveness evidence through systematic review (SR). Efficient and effective searches are an essential component of SR production. The quality of searches used in recent reviews is unknown. We investigated which information resources were used to identify studies for recent SRs of economic evaluations and assessed whether the choice of resources reflected current recommendations.

Methods: SRs of economic evaluations published since January 2013 were identified from MEDLINE. Two reviewers extracted the following information from SRs which met inclusion criteria: general medical databases searched, specialist economic databases searched, HTA sources searched, supplementary search techniques used. Results were compared against information resources recommended by NICE when searching for economic evidence for STAs, and the summary of current best evidence-based practice provided in Sure Info (http://vortal.htai.org/?q=node/336).

Results: 65 SRs met the inclusion criteria; data were extracted from 42/65 reviews. 5 reviews (12%) met or exceeded the search resources recommended by NICE. 9 reviews (21%) searched at least 4 of the 6 types of resource recommended by Sure Info (specialist economic databases, general databases, HTA databases, webpages of HTA agencies, grey literature, collections of utility studies). None of the reviews searched all 6. Although all reviews explicitly described the resources searched, reporting frequently contained errors or lack of clarity in database and interface names.

Conclusions: Our results suggest that the information resources used to identify evidence for the majority of recently published SRs of EEs do not conform to current recommendations. With the closure of two key economic evaluation databases (NHS EED and HEED) the challenges of identifying economic evaluations for reviews will become more challenging. Reviewers should consult current recommendations before carrying out searches since conducting limited searches risks missing relevant EEs.

Tuesday, May 17, 2016, 3:00 PM – 4:25 PM
Room: 201F

Session: Serving Patients and Consumers

Moderator: Janice Hermer

Teaching Pharmacy Students How to Help Consumers Navigate and Select Best the Medicare Plans

Irena Dryankova-Bond
Library manager/Associate Professor of Library and Learning Resources, MCPHS University, Worcester, Massachusetts

Objectives: To describe a Medicare.gov Plan Finder training program used in an elective course and clinical practice rotations with pharmacy students. The goals of the program is to teach future health care providers how to 1) Navigate Medicare prescription drug coverage, 2) Select best plan option based on affordability, coverage, and limitations, 3) Communicate sound options to health consumers.

Methods: At a university pharmacy outreach program advanced pharmacy practice experience students and geriatrics elective students are introduced to the Medicare.gov Plan Finder Tool. Students complete a three hour instructional module with a hands on learning component. Through this pharmacoeconomic approach the students learn how to determine the best Medicare Part D Plan for patients with or without social security extra help, and assistance programs that can lower Medicare costs. In addition, students learn insurance terminology including premiums, co payments, deductibles, doughnut hole, plan limitations, and to better communicate this information to older adults.

Results: Students completed a 5-point Likert survey before training began and again, the last day of the program to assess their attitudes regarding a Medicare part D training program. The average score of the pre-survey was 2.48, showing a disagreement of comfort, understanding and confidence of Medicare Part D; post survey results were positive for these attitudes averaging a score of 4.73. The average change for the attitude assessment was an increase of 2.25, with a notable increase in “confidence of selecting an appropriate Medicare Part D Plan for a beneficiary” up 2.56 to a score of 4.72. In regards to the 7-point

knowledge assessment, given similarly as the Likert attitude assessment, the pre score for the 18 participants was 2.5/7 or 35.7%, with the post score increasing over 2 fold to 5.5/7 or 78.6% average. The area of greatest increased knowledge was of who qualifies for Medicare.

Conclusions: Teaching advanced pharmacy practice experience students how to navigate and select Medicare plans and how to communicate best plan options to consumers is an effective approach in increasing students' confidence, awareness, and knowledge of the Medicare drug coverage program.

Collaborative Collection Development: Building a Patient-Driven Consumer Health Library RESEARCH

Sharon Bailey
Librarian, CAMH Library, Toronto, Ontario, Canada

Alexxa Abi-Jaoude
Research Coordinator, CAMH Education, Toronto, Ontario, Canada

Andrew Johnson
Manager, Client and Family Education, CAMH Education, Toronto, Ontario, Canada

Objectives: The library at a large mental health teaching hospital has partnered with the volunteer-run patient library to develop and maintain a consumer health collection for hospital inpatients and outpatients. How can a research library and a patient library work together, with patient input from start to finish, to build a collection best suited to their information needs?

Methods: This project is patient-centered, with patients participating in various aspects of the research. To that end, we will first bring together an advisory committee including current and former patients and patient library volunteers to provide oversight at all stages:
1. Literature Review: A comprehensive literature review will be conducted to better understand evidence-based approaches to developing consumer health collections.
2. Needs Assessment: Interviews and focus groups will be conducted with patients and patient library volunteers to determine health information needs, preferred types of information, and means of delivery.
3. Collection Development: Drawing from the findings from the literature review, interviews, and focus groups, the hospital library will lead the development of the consumer health collection.
4. Facilitating Uptake: To better meet patient information needs we will develop

complementary health literacy/education initiatives including curriculum development for volunteer training.

Get Appy! A Mosaic of Personnel Offer an App Bar for Patients to Apply Apps to Their Health

Erica Lake
Associate Librarian, Spencer S. Eccles Health Sciences Library, Salt Lake City, Utah

Jean P. Shipman, AHIP, FMLA
Director, Spencer S. Eccles Health Sciences Library, Salt Lake City, Utah

Heidi Greenberg
Research Associate, Spencer S. Eccles Health Sciences Library, Salt Lake City, Utah

Objectives: Consumers and health care professionals alike are interested in mobile health technologies because of their potential to improve health outcomes, lower health care costs, and motivate patients to become more involved in their own care. An inter-professional committee, led by the library, established a hospital app bar where patients and health care professionals can learn about vetted apps and wearables.

Methods: The app bar allows visitors to test drive mobile technologies on demo tablets and wearables. Trained students and staff help visitors browse a vetted collection, assist in connecting patients to technologies prescribed by their providers, and provide instruction on using the technologies to improve health management. A team of health care professionals reviewed and identified patient-facing health and wellness apps and wearables relevant to their area of expertise, and utilizing established evaluation criteria for appraisal, recommended the specific apps and wearables endorsed initially. This paper will share the details that went into creating the app bar, including the composition of the committee, the vetted collection, locating and constructing the bar and equipment, funding, publicity, management and staffing.

Results: The app bar opened February 8, 2016, and is expected to be of huge interest to patients and media. Patient and family engagement and satisfaction surveys will be administered, and the number of items demoed and downloaded will be recorded to show impact. Other performance metrics to be collected include number of wearables purchased at hospital gift shop, number of referring prescriptions and follow-up assessment surveys with patients who received prescriptions (if implemented), and qualitative and quantitative clinician feedback. Future plans for development will be shared.

Conclusions: The number of health apps has exploded in the last year (100,000+), and is the fastest growing category of mobile technologies. The app bar creates a way to assist patients in navigating and utilizing these technologies in a welcoming environment. Providers are more comfortable recommending vetted apps as they have the peer perspective of efficiency built into the app formulary. Patients appreciate the one-on-one training they receive and the guidance on why tracking their activities make a difference in their clinical care. Appy-enabled patients leads to better patient satisfaction.

Perceived eHealth Literacy and Information Behavior of Older Adults Enrolled in a Health Information Outreach Program RESEARCH

Tara Malone
Assistant Professor and Librarian, Robert M. Bird Library, University of Oklahoma Health Sciences Center, Oklahoma City, Oklahoma

Phill Jo
Assistant Professor and Reference Librarian, Robert M. Bird Library, Reference and Instructional Services, Oklahoma City, Oklahoma

Jean Longo
Medical Librarian, Robert M. Bird Library, Reference and Instructional Services, Oklahoma City, Oklahoma

Shari Clifton
Professor, Head of Reference, and Associate Director, Robert M. Bird Library, Reference and Instructional Services, Oklahoma City, Oklahoma

Objectives: We assessed the perceived eHealth literacy and information behavior of older adults participating in a health information outreach program. The study determined how participants' perceived eHealth literacy compared to other populations, which websites and resources older adults search for eHealth information, participants' most/least trusted sources of health information, and if/what barriers exist for older adults when searching for eHealth information.

Methods: In collaboration with a public library system serving a population of 1.2 million, we offered a series of eHealth training courses for older adults, during which a mixed-methods survey was offered. The study took place via mobile computer labs deployed to public libraries and was administered by health sciences librarians. Participants completed the eHEALS eHealth literacy

assessment, as well as supplemental survey questions regarding their information behavior and needs related to seeking health information. Descriptive statistics and content analysis were performed on results.

Results: Based on surveys completed thus far, preliminary results indicate that participants believe access to health resources on the Internet is important and useful, but are unsure what health resources are available, how to find them, or how to evaluate their quality. Many participants owned one or more Internet-capable devices, and had previously searched the Internet for health information via a search engine or commercial medical information site. Use of government health information websites such as MedlinePlus or NIHSeniorHealth was low.

Conclusions: Older adults need more training regarding where reliable online health resources are located and how to find them, and strategies to evaluate the quality of online health information.

Libraries as Publishers: Creating an Open Access Journal to Connect Patients and Providers

Martin Wood, AHIP
Director, Charlotte Edwards Maguire Medical Library, Tallahassee, Florida

Roxann W. Mouratidis, AHIP
Scholarly Communications Librarian, Charlotte Edwards Maguire Medical Library, Florida State University College of Medicine, Tallahassee, Florida

Objectives: The library wanted to take an active role in encouraging dialogue between healthcare professionals and people living with diabetes. Recognizing that patients need access to high quality research, and their providers need a greater understanding of the practical day to day experiences of living with diabetes, the library published a peer-reviewed, open access journal designed to appeal to both groups. The People Living with And Inspired by Diabetes Journal (PLAID, ISSN: 2374-4669) is a bi-annual interdisciplinary journal that is freely available online at ThePlaidJournal.com.

Methods: In order to undertake the process of publishing an online journal, the library partnered with the College of Medicine's Office of Instructional Design. Library staff developed the scope, managed the editing and review process, obtained an ISSN for the journal, assigned DOIs to articles, and developed policies and procedures. Instructional design staff assisted with the customization of open source publishing software, developed branding for the journal, and assisted with the final layout and copyediting of the journal articles. The articles published in The PLAID Journal center around particular diabetes-related themes, as will future issues of the journal. Because the goal of the

journal was to create meaningful exchange between healthcare providers and individuals living with diabetes, we included a mix of research articles written by academics and personal perspective pieces written by patients, families, and members of their support networks.

Results: The journal created meaningful conversation between the research, clinical, and science sides of diabetes, and the practical day-to-day living with diabetes. Qualitative feedback confirms that the Audience Perspectives and Moving Forward features in The PLAID Journal add significant value and understanding of the research and review articles presented in each issue. As a new journal, we find challenges in attracting quality submissions and peer reviewers with sufficient expertise. We expect these challenges will improve as we join the Directory of Open Access Journals, as our content is indexed in PubMed, and we continue to develop an established publication record.

Conclusions: Conversations that have previously existed in social networks and informal patient communities are beginning to find representation in academic literature and benefiting from interdisciplinary consideration and peer review. We are finding that The PLAID Journal is experiencing increasing popularity within the diabetes online community and representative social networks, as well as entering research conversations within our College of Medicine and university as a whole. This trend is expected to continue as we gain national recognition as the premier publication for connecting people living with diabetes and those who work to make living with diabetes better.

Tuesday, May 17, 2016, 3:00 PM – 4:25 PM
Room: 205B

Session: Data Sharing

Moderator: Anne Mackereth

Data Sharing of Otolaryngology Research RESEARCH

Tiffany Grant
Research Informationist, University of Cincinnati, Health Sciences Library, Cincinnati, Ohio

Kristen Burgess
Assistant Director for Research and Informatics, University of Cincinnati, Cincinnati, Ohio

Objectives: Data sharing within the otolaryngology research community encounters a number of barriers that prevent the optimal sharing of data generated by those performing experiments and seeking validation for computational models. Our research goal is to alleviate these barriers by developing tools and infrastructure that will facilitate an optimal means of sharing airflow and acoustic data with the otolaryngology community.

Methods: Informationists at the University of Cincinnati will identify and develop a workflow to organize and manage the key data and file formats generated. Informationists will deposit data into an open-source repository and create a website for researchers and computational modelers to view their data. Otolaryngology research is published in engineering, medical, and international journals, making it difficult to be fully aware of what is done in other fields. The team will identify literature and make citations available on the website, allowing many different types of investigators access to research describing multiple approaches to understanding and treating voice disorders. Lastly, we plan to assess and focus on the efficacy of informationists partnering with the research team to organize and share data.

Results: The team of informationists has met monthly with the otolaryngology researchers who acquainted them with their current research and data in regards to file structures and sizes. The informationists will work with the otolaryngology team to develop a plan for submission to the repository for long term organization, storage and sharing of the selected acoustic and airflow data

created by the investigative team. We are hiring an animator and web designer to help better share and display select data. The informationist team has set up a RefWorks library and began assisting with literature searching focused on the biomechanics of voice as well as integrating the investigative team's research profiles. This project is still in early stages with more results expected by May 2016 and in the upcoming year.

Conclusions: While the project still has a year and a half remaining, the team has begun to successfully bring together varied backgrounds for new types of collaborations as informationists become more embedded into research. This project will be useful to serve as a model for future collaborations with the research community at the University of Cincinnati.

If You Share It, Will They Come? Barriers to Reuse of Shared Biomedical Research Data RESEARCH

Lisa M. Federer
Research Data Informationist, National Institutes of Health Library, Bethesda, Maryland

Objectives: As new mandates require researchers to share their research data, a wealth of shared data has become available, often freely and publicly accessible. This shared data represents a potentially valuable, but frequently underutilized, resource to other researchers. This study aims to identify barriers to reusing shared data and consider how library and information professionals can help mitigate these barriers.

Methods: This study employed a sequential exploratory mixed methods design, using qualitative interviews to inform the creation of a quantitative survey instrument for broader distribution. First, librarians conducted in-depth interviews with 10-20 researchers from a variety of career levels and biomedical research disciplines about their experiences with reusing shared data. Transcripts were coded to identify common themes and concepts with regard to barriers and attitudes toward data reuse. A survey instrument was developed based on these themes and distributed via email and responses were collected electronically from a sample of researchers. Results of the survey were analyzed to identify overall themes, as well as differences in subgroups within the sample (such as groupings by career level and research discipline).

Results: While some respondents demonstrated a willingness to reuse shared data, others had mixed feelings about doing so. Reasons for not reusing shared data included uncertainty about where to locate shared data, concerns about the validity or quality of shared data, and lack of awareness about how to reuse shared data.

Conclusions: While shared data can represent a valuable resource leading to potential discovery, it may be underutilized. Especially as new policies require more and more data to be shared, efforts should be made to communicate the availability and utility of such datasets to researchers, as well as provide training on the skills needed to locate and use these datasets. Librarians can play an important role in this process.

Improving Data Collection, Quality, Workflow, and Sharing for a Multicultural Dementia Screening Study

Kevin B. Read
Knowledge Management Librarian, NYU Health Sciences Library, New York, New York

Alisa Surkis
Translational Science Librarian, NYU Health Sciences Library, New York, New York

Fred W. Z LaPolla
Knowledge Management Librarian, NYU Health Sciences Library, New York, New York

Objectives: A clinical researcher identified the need to improve data management practices and to facilitate data sharing. A collaboration was established with the health sciences library, which identified weaknesses in the data collection, entry, and processing workflows. The library addressed these weaknesses through standardization of variables, introduction of an electronic data capture system, and creation of well-documented, reproducible data processing workflows.

Methods: To standardize variables, librarians mapped existing variable names to the Clinical Data Acquisition Standards Harmonization (CDASH) when possible. Variables not represented in CDASH were mapped to clear, unambiguous names to increase comprehensibility for sharing. REDCap was introduced for electronic data capture to replace the research team's practice of collecting data on paper forms. Data could then be directly imported into statistical programs rather than manually entered from the paper forms. Workflows around data collection for the various dementia screening tests administered were mapped out to ensure uniform data collection, and improve the tracking of study subjects taking multiple tests. Well-documented computer code was created to transform the raw data into an analysis dataset and make the transformation process reproducible and understandable. Discipline-specific data sharing options and general purpose repositories were explored.

Results: REDCap forms for all tests were completed, and clinical researchers on the team were trained on how to use REDCap effectively. New clearly-defined variable names mapped to standards when possible improved the data's ability to be shared. The workflow developed to account for unique subjects participating in different studies improved the comprehensibility of the data, and helped the data manager keep track of study participants.

Conclusions: This informationist project is a strong example of the importance of data management, form design, and electronic data capture systems when collecting clinical research data. The transformation of variables using clinical data standards and clear, comprehensible variable names drastically improved the quality of the data for sharing purposes. Planning and coordinating process workflows when collecting data is necessary for tracking study participants and ensuring appropriate data management practices.

The Library and the Lab: Exploring Partnerships to Manage Research Data

Maria M. Buda
Librarian, University of Toronto Libraries / Dentistry Library, Vaughan, Ontario, Canada

Carolyn Pecoskie
TALint Intern & MI Student, Library & Information Studies, Dentistry Library, University of Toronto Libraries, Toronto, Ontario, Canada

Objectives: To determine the current data management practices of research laboratories in a large academic professional clinical school and whether academic libraries can provide services to support or improve current practices. Data management includes methods to record, share, and archive data generated in the laboratories. Potential services from the libraries include tools and software to archive, store, share, and preserve data.

Methods: The academic institution's Ethics Board was consulted and permission was granted to move this project forward as a program evaluation. Furthermore, permission from the school's Dean was granted to obtain a list of laboratory managers and interview them, if willing to participate. A brief literature review was conducted and it was found that other similar projects did take place in academic libraries but not in a similar professional academic school. An adapted version of Data Curation Profiles Toolkit from Purdue University was selected to conduct in-person interviews with laboratory managers. This will provide us with a brief environmental scan of the current practices in laboratories at the professional school. Once current practices are determined, a report on possible library services to support current practices

will be written, outlining recommendations of how the library can enhance or improve data management services.

Results: Any results will be used to answer how the library can help with data management, such as suggesting data management software; providing training on software for data management, providing assistance in creating data management plans systematically; or suggesting repository options based on needs. Promotion and implementation of new library services will follow shortly after in the form of the action items outlined in the report.

Conclusions: The results of the interviews will be summarized and shared in the form of a report and posted on the library's website. The report will be submitted to the Chief Librarian and the Dean of the School. We hope these results will help to bring to light current data management practices in laboratories of a professional clinical school at a large academic institution. We also hope that the results will provide librarians with an overview of how they might go about the process of reaching out to their communities to talk about research data.

Beyond Data Management: Developing a Comprehensive Data Science Support Program in the Library

Lisa M. Federer
Research Data Informationist, National Institutes of Health Library, Bethesda, Maryland

Objectives: Librarians are well-situated within academic and research institutions to provide support for the increasingly technical and data-driven needs of biomedical researchers. This paper reports on the development and implementation of a comprehensive program for supporting researchers' data science needs at a health sciences library serving a national biomedical research institution.

Methods: In addition to training and services focused on data management, the library expanded its offerings to support researchers' needs in data science through a variety of services. The training curriculum was expanded to offer new courses in scientific computing, statistical programming, and data visualization. In addition, equipment and software to support data science were made available in the library's "Tech Hub," including a data visualization touchscreen. The library also partnered with groups within the institution to conduct pilot studies for data science support programs including electronic laboratory notebooks and DOI minting for datasets and other digital objects. In order to provide this high-level, comprehensive support, librarians engaged in a variety of professional development activities to round out their existing information management skills with additional data science and data

visualization expertise.

Results: With support from the library leadership, librarians and other staff have been able to gain a variety of expertise to support these new services. Library users responded enthusiastically to most new offerings, particularly classes on programming and data visualization. However, some services, like the data visualization screen, are potentially underutilized because of lack of awareness among the community. Other services, such as the electronic lab notebook support, may not move beyond the pilot phase because of lack of uptake.

Conclusions: Libraries have an opportunity to provide valuable services to researchers that may not be offered elsewhere within institutions. Given their role within the research process and their expertise with information management and systems, librarians can be ideal partners for researchers. As with any new service, it can be useful to pilot a small-scale version first to assess scalability and ensure effectiveness. In addition, because many researchers would not expect to find data science support services in the library, it is essential to promote these new services to raise awareness.

Tuesday, May 17, 2016, 3:00 PM – 4:25 PM
Room: 202B

Session: Sustaining Liaison and Embedded Librarian Relationships

Moderator: Erin R. B Eldermire

Evaluating an Embedded Program: Increasing Awareness, Expanding Services, and Fulfilling Patron Needs

Lindsay Blake, AHIP
Clinical Information Librarian, Augusta University, Augusta, Georgia

Darra Ballance, AHIP
Assistant Professor, Augusta University, Evans, Georgia

Victoria Burchfield
Nursing Information Librarian, Augusta University, Augusta, Georgia

Maryska Connolly-Brown
Technical Services Librarian, Walter M. Bortz III Library, Hampden-Sydney, Virginia

Kathy Davies
Associate Director of Research, Augusta University Libraries, Augusta University, Augusta, Georgia

Julie K. Gaines
Associate Professor / Head, GRU/UGA Medical Partnership Library, GRU/UGA Medical Partnership Campus, Athens, Georgia

Kim Mears, AHIP
Scholarly Communications Librarian, Augusta University, Augusta, Georgia

Peter Shipman
Dental Medicine and Cancer Librarian, Augusta University Robert B. Greenblatt, MD Library, Augusta, Georgia

Objectives: To provide a multifaceted, generalizable toolkit for evaluating the effectiveness of an embedded program. An embedded librarian program was assessed using a toolkit which includes traditional activity data and a survey aimed at measuring use of services, awareness of the embedded program, and the perceived value of embedded services.

Methods: A national survey of librarians in the health sciences and a literature review identified few evaluation tools for embedded activities. This lack of resources led librarians to envision developing an evaluation toolkit to assess the success of their embedded program. The evaluation toolkit consists of locally collected data on multiple service transactions and a survey to measure patron awareness, usage, and perceptions of embedded services. Local data was provided by tracking patron interactions, teaching opportunities, and inter-professional collaborations in LibAnalytics. The survey included customized question sets for different patron groups and was reviewed for content and internal structure elements to increase its validity. Qualtrics software was used for survey distribution to students, residents, and faculty in April 2015. Survey results were analyzed to identify patterns of patron usage, awareness gaps, and desired services.

Results: The survey response rate was 10% with 381 completed forms from 4,408 survey recipients. Over 58% of participants who completed the survey reported being aware of their embedded librarian. The majority of faculty strongly agreed that embedded librarians saved them time and were an integral part of their group. Analysis of locally collected data revealed that a high number of reference transactions occur in person which corresponds with survey results. Additional review of the data reflected an increasing trend toward librarian collaborations on grants, publications, and presentations.

Conclusions: The evaluation results are guiding future planning for the embedded librarian program by indicating areas of strength and opportunities for growth. Embedded librarians were contacted primarily through in-person exchanges, demonstrating the strength of having a physical presence in colleges and departments. Lack of contact through online courseware shows an opportunity to expand into virtual areas. Literature searching was the primary use of embedded librarian assistance, illustrating a possible need for greater marketing of other services. The evaluation toolkit has helped provide the library a broad analysis of the program.

Creating a Role for Embedded Librarians Within an Active Learning Curriculum

Dawn E. Hackman
Research & Education Librarian, Harley E. French Library of the Health Sciences, Grand Forks, North Dakota

Erika Fischer
Southeast Clinical Campus Librarian, Harley E. French Library of the Health Sciences, Fargo, North Dakota

Marcia Francis, AHIP
Southwest Clinical Campus Librarian, Harley E. French Library of the Health Sciences, Bismarck, North Dakota

Wendy Lehar, AHIP
Research & Education Librarian, Harley E. French Library of the Health Sciences, Grand Forks, North Dakota

Annie Nickum
Research & Education Librarian, Harley E. French Library of the Health Sciences, Grand Forks, North Dakota

Kelly Thormodson
Assistant Director, Harley E. French Library of the Health Sciences, Grand Forks, North Dakota

Objectives: This paper examines the efforts of one small academic health sciences library to create a role for its embedded librarians within a new pedagogical culture, which emphasizes the use of active learning in its professional health sciences programs. This paper will discuss the planning process in what will be a multi-staged project.

Methods: In summer 2016, the library will move into a new health sciences building, the design reflecting cultural and pedagogical shifts within the school. Students will share interdisciplinary study communities and classes will be taught in rooms optimized for active learning methodologies. These changes have given the health sciences librarians opportunities to enhance their roles within the medical and health sciences school. In 2014, the library reevaluated its mission, vision, and strategic plan to respond to anticipated changes. Library staff participated in planning retreats and group discussions, in which embedded services were first envisioned. Subsequent efforts have resulted in liaison and clinical librarians joining curriculum committees and other institutional initiatives, such as an Active Learning Task Force. Librarians are being included in faculty development workshops, where they will promote the librarian's ability to support curricular goals within the active learning classroom.

Results:This first stage of the project has already seen both subtle and drastic

successes. Librarians were involved with planning two active learning workshops. These workshops have led to the incorporation of active learning in bibliographic instruction and, more importantly, have led to faculty reaching out to librarians to be involved in their classroom instruction. One faculty member reported that, after attending one of the workshops, she completely reworked her syllabus mere days before the beginning of the semester to include active learning exercises and two librarians (liberal arts & medical) into her curriculum. The increased visibility of librarians in curriculum-focused discussions is the biggest success, as it has led to a greater interest on the part of school administrators on what librarians can do and possible future roles. Librarians are being incorporated into the school-wide transition to the new building and new pedagogies.

Conclusions: Identifying and approaching faculty/administrative champions is a valuable opportunity for librarians to become more involved in medical and health sciences curricula through active learning pedagogies. Areas for future exploration include how the design of the active learning classrooms will affect bibliographic instruction. Additionally can the inclusion of librarians in the transition teams lead to positive interactions with non-champion faculty?

The Sustainability of Embedded Librarians in Academic Health Sciences Libraries RESEARCH

Stephanie J. Schulte
Associate Professor/Head, Research and Education Services, Health Sciences Library, The Ohio State University, Columbus, Ohio

Pamela S. Bradigan, AHIP
Assistant Vice President and Director, Health Sciences Library, Columbus, Ohio

Objectives: The purpose of this research is to explore, from the library director perspective, the relationships among funding mechanisms, job classification, job stress, and career trajectory of embedded librarians. It will also explore how academic health sciences libraries have integrated embedded librarians (ELs) and the impacts of this role on the rest of the library.

Methods: This study utilized a combination of qualitative and quantitative approaches in a descriptive survey targeted to all library directors of libraries who are members of the Association of Academic Health Sciences Libraries. Survey questions included a mix of multiple choice, open-ended, and Likert-type questions. Participants self-identified their willingness to participate in follow-up interviews conducted in person or via phone using a structured interview. Statistical analysis may include descriptive statistics, cross tabulations and chi-square tests for pairs of questions, and possibly logistic regression examining

several independent variables. Interview responses will be thematically analyzed.

Results: This study is ongoing, and the response rate limits the generalizability of the survey results. Preliminary data indicate that less than half of libraries represented in the results have ELs who are physically and culturally integrated into units. Of those that do, the vast majority of these positions are funded in the same way (ongoing library general funds) and have the same rank/classification as other professional librarians in their organizations. Most respondents perceived their ELs to have job security and be connected to the health sciences librarian professional community. Most also did not perceive the nature of embedded librarian work to affect the ability of ELs to participate in professional development or library activities, nor did they perceive the specialization often required of ELs to negatively impact their careers. There was no consensus on whether ELs experienced more stress than non-ELs or whether rank/classification impacted stress levels for ELs. Data suggest that paraprofessional positions are now performing at higher levels compared to past and have assumed former librarian roles in response to embedded positions. The biggest challenges noted for the future of ELs included the skillset required of ELs, funding sources, and having enough staff to meet demand. Interviews are expected to begin in Spring 2016 and will enhance understanding of this limited survey data.

Conclusions: The challenge of sustaining ELs in academic health sciences libraries continues to evolve.

Embedding Librarian Expertise Across a Public Health Curriculum

Abraham Wheeler, AHIP
Health Sciences Librarian, MSU Libraries, East Lansing, Michigan

Objectives: I will present how a librarian's expertise in data information literacy and in locating and evaluating evidence based literature can be effectively embedded into a public health program and improve student outcomes/competencies/skills and conform to robust accreditation standards and professional goals.

Methods: Traditionally having limited interaction with the online program in public health at a large university, the librarian has become embedded longitudinally and holistically throughout the Master of Public Health curriculum. By aligning the librarian's expertise with the goals and objectives of the program, the librarian helped reinvent and improved the student experience in a totally online program. The librarian worked cooperatively with each faculty

member to identify gaps in student learning, and then to incorporate librarian expertise in research, evidence based practice and data literacy into appropriate points in the curriculum. Each course was built with librarian expertise as an essential component. The librarian's efforts were evaluated using faculty feedback and student performance data. Outcomes were increased integration of the librarian into the curriculum, increasingly rigorous course rubrics, and students with increased competence in evidence based practice and data literacy.

Results: Faculty of courses with the increased librarian involvement have reported better grades, increased sophistication of evidence presented, and fewer email questions about the assignments. Faculty also reports an improvement in the student's ability to incorporate evidence into assignments and papers. These positive results have lead to a doubling of courses with intensive librarian involvement.

Conclusions: The librarian has successfully expanded their role across the curriculum and helped shape assignments. Courses that have the expanded librarian presence have been able to increase the rigor of their rubrics, and seen an improvement in the quality and presentation of student research. Participating faculty have been able to significantly increase the amount of required evidence based practice and data literacy requirements in their rubrics.

Seeing the Big Picture: Sustaining Success in Liaison Services

John Cyrus
Research and Education Librarian, Tompkins-McCaw Library / Research & Education, Richmond, Virginia

Roy E. Brown
Tompkins-McCaw Library for the Health Sciences, VCU Libraries, Richmond, Virginia

Karen Gau, AHIP
Research and Education Librarian, Tompkins-McCaw Library for the Health Sciences, Richmond, Virginia

Irene Machowa Lubker
Research and Education Librarian, Tompkins- McCaw Library for the Health Sciences, Richmond, Virginia

Jennifer McDaniel
Research and Education Librarian, Tompkins-McCaw Library for the Health Sciences, Richmond, Virginia

Barbara A. Wright, AHIP
Project Librarian, Tompkins-McCaw Library / Research & Education, Richmond, Virginia

Emily J. Hurst, AHIP
Head, Research and Education, Tompkins-McCaw Library for the Health Sciences, Richmond, Virginia

Objectives: This program will discuss the ramifications of maintaining successful liaison services on the health sciences campus of a large, urban research university. Both sustainability and scalability of the liaison program will be explored.

Methods: Between 2012 and 2015, the liaison program of the health sciences library experienced substantial growth and success. Traditional indicators such as instruction sessions, in-depth consultations, and reference transactions increased significantly over this time. In addition, as relationships within the schools matured, liaisons took on increasingly visible and integrated roles within their schools. Librarians soon found themselves maintaining traditional services and roles and participating in new, potentially more impactful opportunities. This presentation will evaluate the success of the liaison program and describe measures taken to sustain the success of the program. Quantitative analysis of library statistics, qualitative interviews with liaison librarians, survey of faculty, and analysis of library trends were used to inform program and service options.

Results: Statistical analysis showed a year-to-year increase in the total number of sessions conducted by the liaisons and a positive trend in total number of users served. An inverse relationship was observed between the number of educational sessions and the number of research consultations, which increased year-to-year. The total number of users served varied considerably at the unit level, but both the School of Medicine and VCU Health showed consistent year-to-year increases. Nursing, Dentistry, Pharmacy, and Allied Health schools all displayed yearly fluctuations in total number served between -21% and 32%.

Seventy-eight percent of faculty provided responded (n=25) to a request for feedback. Responses to the program and individual liaisons were positive. When asked to offer examples of contributions made by liaisons, sixty-seven percent of respondents provided a listing of service offered while twenty-eight percent cited specific ways in which the liaison impacted either their work, that of their students, or the mission of their program.

A survey of liaisons showed agreement on how to determine impact and the best ways to measure success of the program as a whole. Principle methods cited for gauging both individual impact and programmatic success included formal and informal feedback, student or project success stories, statistics, and unique opportunities or collaborations.

Conclusions: Blanket assessment of liaison programs in health sciences libraries is difficult as they are often highly personalized to the local context. This program has seen a sustained upward trend in total number of sessions, hours engaged, and number served. Feedback from users is very positive, and use of liaison services is expected to continue its growth. As a result, the program has had to find efficiencies, trading narrow, low-impact efforts for broader engagement and participation in more high-impact projects.

Tuesday, May 17, 2016, 3:00 PM – 4:25 PM
Room: 202D

Session: Providing Resources for Medical Innovation into Libraries

Moderator: Susan A. Fowler

Providing Resources for Medical Innovation into Libraries

Susan A. Fowler
Medical Librarian, Washington University in St. Louis School of Medicine, St. Louis, Missouri

Advancing Medical Innovation: Biofabrication and Medical Device Prototyping in Libraries

Peace Ossom Williamson
FabLab CoManager / Health Sciences Librarian, University of Texas at Arlington, Arlington, Texas

Patricia F. Anderson
Emerging Technologies Informationist, Taubman Library, University of Michigan, Ann Arbor, Michigan

Mary Helms
Head, Strategic Initiatives, McGoogan Library of Medicine, University of Nebraska Medical Center, Xx, Nebraska

This panel is for medical librarians interested in learning about the use of "maker" or emerging technologies in health fields and in developing access to these services and equipment for use in their libraries. Participants will learn from librarians in their use of equipment like 3D printers and spaces like fab labs and makerspaces for their applications in medicine. This session will cover the various methods libraries use to implement these services, the equipment available in their libraries, and how libraries are addressing safety, copyright, and patent issues. The panelists are innovators at diverse institutions, and they will discuss trends in biofabrication, 3D printed prosthetics for children, and

innovation in prototyping medical devices. The impact these librarians found from incorporating maker technology services in health sciences libraries will also be shared.

Tuesday, May 17, 2016, 3:00 PM – 4:25 PM
Room: 203B

Session: Clinical Decision Making

Moderator: Renee Gorrell

Clinical ECompanion: Development of a Point-of-Care Information Tool

Charles B. Wessel
Head of Research Initiatives, Research, Instruction, and Clinical Information Services, Pittsburgh, Pennsylvania

Julia J. Dahm
Technology Services Librarian, University of Pittsburgh, Pittsburgh, Pennsylvania

John LaDue
Head of Knowledge Integration, Health Sciences Library System, Pittsburgh, Pennsylvania

Objectives: To build and evaluate a primary care information tool that bundles freely available Web resources in order to answer point-of-care clinical questions. This was a multi-year project funded by NN/LM Middle Atlantic Region. Clinical eCompanion is intended for health providers practicing in community settings that lack access to a medical library or where licensed information tools are cost prohibitive.

Methods: The project occurred in three phases, with each phase lasting one year. Phase I, the feasibility project, explored the viability of building the tool. Phase II, the evaluation and demonstration project, recruited five clinical experts to participate in structured interviews. They were asked to give a narrative of the tool's performance as they performed six hypothetical clinical usability tasks. Phase III, the validation study, involved a greater number of healthcare practitioners, who used Clinical eCompanion in their practice for one month to answer their point-of-care questions. After using the tool, a Likert scale questionnaire solicited their feedback on perceived impact of use and performance of the tool. The methodologies in Phases II and III were submitted for approval to the university's Institutional Review Board, which designated this project exempt from formal review.

Results: From the structure interviews in Phase II, results from five clinicians indicated success locating patient education materials and journal articles, along with suggestions for improvement of information content and navigation. The validation study in Phase III provided more detailed results on information quality, system quality and personal satisfaction. Results for information quality questions were favorable: 96% agree information is current, 78% agree information is relevant and 74% agree the information on the site is complete. System quality questions received favorable responses: 85% agree the content on the site is easy to read, 85% agreed the pages load in a timely manner, and 70% agreed the search function is helpful. Questions measuring perceived impact on the individual were distributed among the response categories. When asked "overall I'm satisfied with the tool," 52% agreed. With the statement "I would recommend the tool to other health care practitioners," and 51% agreed. An unexpected result was that licensed point of care resources were available to 96% of participants, indicating that their information needs may already be met by a paid subscription.

Conclusions: Study data and results indicate Clinical eCompanion is successful in answering primary care providers' questions at the point of care.

Taking the CASE to the People: Testing the Inter-Rater Reliability of the Critically Appraise Summaries of Evidence (CASE) Form Using Point-of-Care Tools with Health Sciences Students and Professionals RESEARCH

Suzanne Shurtz, AHIP
Associate Professor/Instructional Services Librarian, Medical Sciences Library, Texas A&M University, College Station, Texas

Margaret J. Foster, AHIP
Systematic Reviews and Research Coordinator/ Associate Professor, Texas A&M University, College Station, Texas

Objectives: To determine the inter-rater reliability of the CASE (Critically Appraise Summaries of Evidence) form when used by health professionals and students in appraising a treatment summary in a point of care tool.

Methods: Point of care tools are used to treat patients, but no standard exists to evaluate the quality of the information in these products. Two librarians at an academic medical library developed ten questions, the Critically Appraise Summaries of Evidence (CASE) form, to assess point of care treatment

summaries. To test the reliability of the form, the CASE questions were loaded into Qualtrics survey software. Qualtrics was set up to randomly select two treatment summaries on hypertension from two of four different point of care tools for each participant to evaluate using the CASE form. Thirty-two health science students and professionals were recruited via email. During in-person sessions, participants were asked to evaluate two treatment summaries using the questions of the CASE form loaded in Qualtrics. Mouse movements navigating through the summaries were captured using Morae recording software.

Results: There were thirty-two participants. Collected data were analyzed to see if CASE form questions were answered similarly for treatment summaries from the same resource. The average overall score on usability was 73.44 points. Scores above 68 are considered above average or useful. Inter-rater reliability as measured with Fleiss Kappa was low, however, percent agreement averaged 76% overall. Ninety-one percent agreed with the statement "I think this form is useful for learning/teaching how to critically appraise evidence based summaries." Sixty-nine percent would recommend the form to a colleague.

Conclusions: This study demonstrated that the CASE form is a reliable tool for health science students and healthcare professionals and could be used for educational purposes. Wording on several questions on the CASE form is being edited, based on participants' feedback, to improve its usability.

Magic Eye: Seeing Hidden Trends in Mobile Information Seeking in a Clinical Setting RESEARCH

Karen Yacobucci
Content Management Librarian, NYU Health Sciences Libraries, Maplewood, New Jersey

Aileen M. McCrillis, AHIP
Head, Information Services and User Experience, NYU Health Sciences Library, New York, New York

Joey Nicholson
Education and Curriculum Librarian, NYU Health Sciences Library, New York, New York

Stephen Maher
Assistant Director, Content Management and Scholarly Communication, NYU Health Sciences Library, New York, New York

Objectives: All medical students and residents at an academic medical center are provided with iPad Minis for their clinical duties. Our objective is to assess information-seeking behavior of these users. Previous research in this area has focused on use of institution-supplied resources or devices. This study will examine the broader use of mobile devices for clinical decision-making.

Methods: The authors will attend the clinical rounds of two departments over 4 weeks to assess the nature of the clinical questions that arise, characterize the information resources used, and evaluate the effectiveness of institution-provided iPads by using an observational checklist. Semi-structured focus groups will be conducted to qualitatively explore the subjects' preferences in finding information and the barriers they experience in device use. Frequencies will be used to assess information collected during observations and qualitative data will be analyzed using a grounded theory approach.

The results of this study will reveal the learning needs of students and residents with regards to answering clinical questions at the point-of-care and inform the development of a curriculum to enhance the use of mobile technology at the bedside. It will also elucidate the usage patterns of institution-provided iPads versus personally owned devices.

Results/Conclusions: Data collection is in progress and final results and conclusions will be presented at the conference.

Morning Report Mosaic: Information Resources Used to Answer Clinical Questions from Morning Report RESEARCH

Julia M. Esparza, AHIP
Head, User Education and Outreach Services/Associate Professor, Health Sciences Library, Shreveport, Louisiana

David C. Duggar, AHIP
Head, Library Liaison Program, LSU Health Shreveport, Health Sciences Library, Shreveport, Louisiana

Taylor Gatson
Research Apprentice, Department of Internal Medicine, Shreveport, Louisiana

Deepthi Gangireddy
House Officer Program Year 3, Department of Internal Medicine, Shreveport, Louisiana

Megan Hughes
House Officer Program Year 2, Department of Internal Medicine, Shreveport, Louisiana

Gunjan Kahlon
Chief, Internal Medicine Section, Department of Internal Medicine, Shreveport, Louisiana

Objectives: Klein-Fedyshin et al., from the University of Pittsburgh Health Sciences Library System (UPHSLS), recently published their research, "Evaluating the MEDLINE Core Clinical Journals (CCJ) Filter." This article prompted the following research questions: Do the information resources used by library faculty at LSU Health Shreveport (LSUHS) for Internal Medicine Morning Report Follow-up differ from those used by UPHSLS to answer their Morning Report questions? If there are differences, what are they?
Methods: Morning Report topics and the resources (textbooks, web documents, journals, etc.) used to answer related questions from October 2013 to June 2015 were analyzed. Journal sub-analysis was completed by: a) Bradford's law of scattering, b) Institute for Scientific Information Impact Factor, c) National Library of Medicine Medline CCJ list, d) citations per journal title, and e) date distribution of journal citations. In addition, two residents reviewed the Morning Report topics and placed them into broad Medical Subject Heading categories. If there was a conflict, a senior clinical faculty member made the final decision on subject assignment after consulting with the residents. Textbooks were ranked by usage to determine the most prevalent titles for answering Internal Medicine clinical information needs.
Results: Communicable disease, gastroenterology, cardiology, hematology, and nephrology were the top five subjects out of the 900 topics. Twenty textbooks were used to answer 76% of the clinical questions. Similarities between UPHSLS and LSUHS included: 55% of the top 20-22 journals used were on the CCJ list, 8% of the journals also appeared in the top Bradford tier, and 85% of the articles used were from the most recent ten years. A difference was that 60% of the Bradford titles were on the CCJ list at LSUHS, while 55% were at UPHSLS. The institutions had a 35% overlap of titles. When evaluating the impact factor of the journals, the top 20 journals in the *Journal Citation Reports* General, Internal Medicine list had an overlap of 65%.
Conclusions:By replicating the UPHSLS study, five points stand out. The percentage of resources used from the CCJ is comparable. Individual title use differed which should be considered when evaluating a journal collection against collection development lists. It takes many journals in comparison to textbooks to answer clinical questions. Journal articles within the last ten years will answer a majority of clinical questions. Key clinical information may be missed when using the CCJ filter in PubMed.

Does Computerized Evidence Use Improve Patient Skin Problem Outcomes? A Randomized Cluster Controlled Trial RESEARCH

Marianne Burke, AHIP
Director, Dana Medical Library, Burlington, Vermont

Benjamin Littenberg
Henry and Carleen Tufo Professor of Medicine and Professor of Nursing, General Internal Medicne Research, Burlington, Vermont

Objectives: Physician surveys report that computerized clinical evidence sources (CCES) bridge knowledge gaps and support clinical decisions. However, few studies have evaluated the impact of provider use of CCES on patient clinical outcomes. The objective of this research is to evaluate the impact of one CCES, VisualDx, a dermatology evidence source technology, on the outcomes of patient skin problems presented in primary care.

Methods: The study design is a cluster randomized controlled trial of the effect of use of VisualDx by primary care providers (PCPs) on patient skin conditions. The aims are to test whether use of VisualDx by PCPs impacts the time to resolution of patient skin problems and the number of follow-up visits for the same problem. Participants include PCPs and their patients with skin problems. Providers are randomized to the intervention group that refers to VisualDx, or to control that does not refer to it but may refer to another information source or none (standard treatment). Patients are interviewed 30 days after their visit to collect outcome data. We estimate the need to enroll 30 PCPs and 300 patients to achieve statistical power to detect an 8 day difference in time to problem resolution.

Results: To date, 31 PCPs, including 12 residents, have consented. Each completed a baseline survey and training, and permitted a letter over their signature to be sent to their patients informing them of the study. 96 patients have been identified as potentially eligible, 84 have been sent introductory letters, and 75 have been called at least once. Of 25 patients contacted by telephone, 17(68%) have consented to participate and have completed the 30-day interview. We estimate 1300 - 1500 patients need to be be identified to consent 300.

Conclusions: This patient-centered research could have clinically meaningful outcomes. It may contribute to knowledge of how research-based content technologies contribute to care.

Tuesday, May 17, 2016, 3:00 PM – 4:25 PM
Room: 203D

Session: Professional Communication Skills: Publishing and Presenting Your Research

Moderator: Patrick McLaughlin
Ariel Deardorff

Professional Communication Skills: Publishing and Presenting Your Research

Katherine G. Akers
Biomedical Research/Research Data Specialist, Wayne State University, Detroit, Michigan

Jacqueline Wirz
Director of Career & Professional Development and Graduate Student Affairs, Research Data Ninja, Assistant Professor, Oregon Health & Science University, Portland, Oregon

Natalie Clairoux
Dentistry Librarian, Bibliothèque de la santé, Université de Montréal, Montreal, Quebec, Canada

Joey Nicholson
Education and Curriculum Librarian, NYU Health Sciences Library, New York, New York

Carole M. Gilbert, AHIP, FMLA
Editor, Journal of Hospital Librarianship, Taylor & Francis, Publisher, Royal Oak, Michigan

Cari Merkley

This program will bring together leaders from the profession to share professional communication strategies. The session will be divided into two

communication areas: presentations and publishing. The presentation component will focus on how librarians can improve their presentation and public speaking skills. The publishing component will focus on how to write for publication, identify publication venues, and successfully navigate the publication process. The emphasis of this panel discussion will be in effective communication strategies for sharing information.

Panelists include Jackie Wirz (Research Data Specialist, Oregon Health & Sciences University), Natalie Clairoux (Biomedical Librarian, University of Montreal / Université de Montréal), Joey Nicholson (Education and Curriculum Librarian, New York University Health Sciences Library), Diane Cooper (editor, Journal of the Medical Library Association), Carole Gilbert (editor, Journal of Hospital Librarianship), and Cari Merkley (co-editor, Journal of the Canadian Health Libraries Association / Journal de l'Association des bibliothèques de la santé du Canada).

Tuesday, May 17, 2016, 3:00 PM – 4:25 PM
Room: 104C

Session: The Cross-cultural Mosaic Cafe: When It Comes to Making Intercultural Connections

The Cross-Cultural Mosaic Cafe: When It Comes to Making Intercultural Connections, the "Beginner" And the "Expert" Is All of Us: Sharing Your Successes and Failures Matters! Inspire and Learn from Each Other Through Collaborative Dialogue Around Thought-Provoking Intercultural Questions

Martha E. Knuth
Medical Librarian, Western Michigan University Homer Stryker MD School of Medicine, Kalamazoo, Michigan

Katie A. Prentice, AHIP
Associate Director for User Experience and Assessment, Schusterman Library, University of Oklahoma-Tulsa, Tulsa, Oklahoma

Shannon Jones

Francisco J. Fajardo
Clinical Engagement Librarian, Florida International University Medical Library, Miami, Florida

Gregg A. Stevens, AHIP
Public Services Librarian, Mercer University, Atlanta, Georgia

Gurpreet Rana
Global Health Coordinator, University of Michigan, Ann Arbor, Michigan

Medical librarians often make connections between the mosaic of cultures that surround our libraries and services. In addition, we often bridge the gap between a variety of cultures: cultures associated with ethnicity, profession, identity, religion, and much more. Rather than a melting pot, libraries are often

the meeting place for connections to be made that build a more cohesive mosaic.

Being in the heart of these intercultural connections, as well as establishing and fostering them, is a huge challenge. There are as many different approaches for librarians to be involved in cross-cultural connections as there are combinations of cultures to be connected. That means every librarian's experiences in intercultural connections, good or bad, need to be shared in order for our profession to learn from each other and inspire each other to build better practices in intercultural communication.

The World Café format is a well-established yet unique format that fosters exploration and innovation using an open dialogue format. Participants are rotated amongst small group discussions, each focusing on a different relevant topic. Like libraries, the café format represents a 'third place' that facilitates an intimate and creative conversation to take place. Participants will be rotated through 5 stations, switching tables, topics, and partners every 15 minutes. Like a speed open forum, this format seeks to equalize participants in order for all points of view to be heard and learned from. Contribute and learn from our mosaic! Join the conversation at the cross-cultural café.

Wednesday, May 18, 2016, 9:00 AM – 10:25 AM
Room: 201D

Session: Lightning Talks #3

Big Data for Small Hospital Libraries, How Do the Pieces Fit?

Louise McLaughlin
Information Specialist, Woman's Hospital, Baton Rouge, Louisiana

Question: What is the relevance of Big Data (BD) to librarians in small hospital libraries and how can BD be advantageous to this healthcare setting?
Methods: The published research reports that derive from massive data sets can be invaluable to hospital librarians who work closely with administrative and clinical decision-makers. A case report illustrates the richness of research findings and the administrative response.
Results: A list of sources and search strategies was developed by the hospital librarian for continuing use.
Conclusion: Continuing awareness of research generated from the BD aggregation and curation process can enrich a hospital librarian's value to critical stakeholders.

Partnering to Present Publishing: A Seminar on the Publishing Process

Heather L. Brown, AHIP
Head of Collection Services, McGoogan Library of Medicine, Omaha, Nebraska

Objective: The need to publish permeates throughout the health sciences. Its process can be a mystery to those without editorial experience or insider knowledge. Writers often look to the library for assistance in publishing. The library, with its connections to publishers and knowledge of scholarly communications issues, is a natural conduit for demystifying the publishing process.

Methods: A group of campus stakeholders were brought together by the library to discuss the publishing and writing needs of faculty, staff, and students. It

became evident that the campus community would greatly benefit from a seminar, featuring speakers from the publishing industry. Publishers were approached, with three agreeing to present. Topics were coordinated so that there was minimal redundancy. Topics included strategies for how and where to get published, book publishing, methods used to measure the impact of publications, and open access publishing.

Though coordinated through the library, there was a partnership with the Faculty Development department for publicity and registration needs. Other key stakeholders were also called upon to help publicize the event. Additionally, campus video services were employed to record and live stream the event.

Health and Literacy: The Mini-Med School and the Literacy Foundation Working Together to Reach Native Children

Monique Clar
Health Sciences Librarian, Bibliothèque de la santé, Montréal, Quebec, Canada

Sophie Chiasson
Library technician, Service des acquisition, Montréal, Quebec, Canada

Éric Drouin
First Nations Program Advisor, Département de pédiatrie, Montréal, Quebec, Canada

Bianca Seminaro
Student, École de bibliothéconomie et des sciences de l'information, Montréal, Quebec, Canada

Maryse Fagnant
Medical student, Medical School, Montréal, Quebec, Canada

The Mini-Med School (MMS) aims to motivate Native children to stay in school, introduce them to the health professions and encourage them to have healthy living practices. As reading skills play a major role in student retention, the MMS, in collaboration with the Health Library and the Literacy Foundation, is taking actions to facilitate children’s access to books.

The MMS is a University program where health sciences students visit Native communities' schools. The Health Library participates in those visits, develops book collections with the school libraries and recruits LIS students for the MMS book booth. The Literacy Foundation’s The Gift of Reading program aims to prevent reading and writing difficulties by giving books to underprivileged children. The MMS and the Library are joining forces with the Foundation to give

children a better access to books. The medical librarian will continue working with the schools to upgrade the donated collections according to data on book usage and appreciation. MMS book collections will also be provided to three additional Native schools. On the other hand, The Gift of Reading program will now reach many Native children at home in those communities, by allowing them to receive a brand new book.

Supporting a Compassionate and Reflective Practice

Charlotte M. Sievert, AHIP
Information Services Librarian, Summa Health System - Medical Library, Akron, Ohio

Objective: The Schwartz Center Rounds is a program dedicated to strengthening the human connection between patients and healthcare workers. This project aims to provide the attendees of the Rounds at our institution with opportunities for further reflection on the themes mentioned at each session. To achieve this purpose, we are providing easy access to relevant literature via a dedicated webpage.

Methods: Setting: A hospital campus, one of the nearly 500 healthcare institutions that host the Rounds.

Intervention: The medical library serving this campus has created a webpage that provides links to articles relevant to the themes which emerge during each session of the Rounds. Through a literature search, a few relevant articles from the biomedical literature are identified. The librarian creates links to these articles on the webpage. Included are an abstract and a reference in American Psychological Association citation style. The newly added content is then publicized to the campus. It was anticipated that some attendees would show an interest in the readings and, after each publicity effort, this was measured in clicks on the website. This project gives the librarian an opportunity to contribute to the interdisciplinary exchange that is the hallmark of the Schwartz Center Rounds.

Embedded Librarian Program in a Magnet Hospital to Increase Scholarly Output

Caroline Marshall, AHIP
Senior Medical Librarian, Public Services, Los Angeles, California

Lianna Ansryan
MSN, RN, RN_BC, CNS, Nursing Research and Development, Los Angeles, California

Bernice Coleman
PhD, RN, ACNP-BC,, Nursing Research and Development, Los Angeles, California

Objective: This paper describes the integration of a librarian into the Inspiring Writing in Nursing Program (I-WIN), a Nursing project in a Magnet Hospital, created to engage, promote and mentor publication submissions from frontline clinicians. It shows how the continued support and availability of a librarian provided the authors with critical skills for their publication.

Methods: Podium presenters from the 2014 Nursing Research Conference were invited to attend a Writing Workshop scheduled for January 2015. Prior to the first meeting the authors attended a one on one with a librarian to learn information seeking skills, such as what databases to use, search terms, search tools and how to manage their citations using EndNote. The librarian continued to provide support throughout the process, meeting the nurses at whatever information literacy level they were at. The interactions ranged from several in depth one on one sessions to brief question and answer sessions. The information required ranged from repeated information literacy training, and input on how to choose the right journal and author guidelines. An online survey showed that the authors viewed the authors valued time spent with the librarian and felt they had acquired new skills.

Increasing Access, Reducing Stigma: Improving HIV/AIDS Information Communication in North Florida

Margaret Ansell
Nursing and Consumer Health Liaison Librarian, Health Science Center Libraries, Gainesville, Florida

John Reazer
Medical Information Services Librarian, University of Florida, Jacksonville, Florida

Hannah F. Norton, AHIP
Reference & Liaison Librarian, Health Science Center Libraries, Gainesville, Florida

Gretchen Kuntz
Director, Borland Library, Jacksonville, Florida

Michele R. Tennant, AHIP
UNIVERSITY LIBRARIAN, Health Science Center Libraries, Gainesville, Florida

Objectives: With funding from the NLM HIV/AIDS Community Information Outreach Project, two libraries have partnered with local public and community health providers to design a project that will create access to HIV/AIDS information resources to vulnerable populations, providing a model for future information outreach projects and demonstrating the impact and importance of the health information profession to the community.

Methods: Four overall objectives guide the activities of the project: facilitating collaboration, resource development and dissemination, skills development, and general awareness raising. The team will facilitate collaboration among healthcare and service providers focused on HIV/AIDS prevention and treatment by hosting "Collaborating with Strangers" workshops featuring speed-meetings and community information needs assessment. Using needs assessment information, the team will design easy-to-read materials and videos highlighting local services and information gaps. A cornerstone of the project is to improve the HIV/AIDS information-seeking behaviors of vulnerable populations through information resource trainings targeted towards both health consumers and healthcare professionals. Additionally, the team will raise general awareness in our community of HIV/AIDS history and societal impact by hosting the NLM traveling exhibit "Surviving and Thriving: AIDS, Politics, and Culture."

Outbreak! LibGuides Creation to Support Users During Pandemics

Susan Swogger
Collections Development Librarian, Health Sciences Library, Chapel Hill, North Carolina

Mary E. White
Global Public Health Librarian, Health Sciences Library, Downey, California

Objective: This lightning talk describes the development of a series of timely LibGuides focused on current disease outbreaks, namely the 2014 Ebola Virus and the 2016 Zika Virus Outbreaks. It discusses the goals and process involved in this library-provided service, from identification of need, of potential users or development partners, and of relevant information sources as the outbreak progresses.

Methods: The series began as the Ebola Outbreak of 2015 was causing alarm among the general public and hospital users. Nursing clinical faculty approached the Library for assistance with a simulation project focusing on a

potential Ebola outbreak; the decision was made to create an information resource that could support both this group, the affiliated Hospital, and members of the public. The Library designed a flexible LibGuide template structure using the ongoing clinical discussion as an outline that could be used both for the current and any future similar outbreaks requiring support. The ongoing Zika Virus Disease outbreak provided another opportunity to test the template; it proved useful to quickly gather and promote relevant information.

PRESS: Peer Review of Electronic Search Strategies 2015 Updated Guideline Statement

Margaret Sampson, AHIP
Manager, Children's Hospital of Eastern Ontario, Ottawa, Ontario, Canada

Jessie McGowan, AHIP
Health information consultant / Adjunct professor, School of Epidemiology, Public Health and Preventive Medicine, Ottawa, Ontario, Canada

Douglas M. Salzwedel
Trials Search Coordinator / Information Specialist, University of British Columbia, Vancouver, British Columbia, Canada

Elise Cogo
Research Consultant & Information Specialist, Independent, Scarborough, Ontario, Canada

Carol Lefebvre, HonFCLIP
Independent Information Consultant, Lefebvre Associates Ltd, Oxfordshire, United Kingdom

Objective: To update an evidence-based guideline for peer review of searches for evidence syntheses, including systematic reviews and health technology assessments, and to assess the evidence of impact of peer review on search performance.

Methods: Evidence-based elements related to search quality or search errors were identified through a systematic review, web-based survey and international consensus development forum. Eligible evidence for the systematic review related either to individual search elements or checklists of elements and must have reported impact on recall or precision. Evidence was summarized and integrated with expert opinion obtained from the survey and forum. A guideline statement was published including an assessment form, guidance on its use and recommendations for librarians implementing PRESS in their practice. An

elaboration and explanation document was also prepared documenting complete methods for the update as well as systematic review findings.

eSRAP: A System for Collaborative Monitoring of Latest Trends in Patient-Oriented Research

Vera Granikov
Research-embedded health information specialist, Family Medicine, Montreal, Quebec, Canada

David Li Tang
Adjunct Professor, Family Medicine, Montreal, Quebec, Canada

France Bouthillier
Associate Professor, School of Information Studies, Montreal, Quebec, Canada

Pierre Pluye
Full Professor, FRQS Senior Research Scholar, Family Medicine, Montreal, Quebec, Canada

Objectives: Despite numerous literature surveillance tools and applications, users continue to be challenged by time constraints, information overload, and insufficient appraisal skills. eSRAP is a collaborative research trend monitoring system for Patient Oriented Research (POR). Through crowdsourcing, its purpose is to help POR communities to quickly identify relevant high quality studies, to support teaching, continuing education, preparation of protocols, and publications.
Methods: eSRAP is an open access system funded by the 'Methodological Developments' platform of the Quebec SPOR-SUPPORT Unit. Using search strategies developed by a librarian and subject experts, the system retrieves new publications as soon as they are indexed in bibliographic databases (e.g., Scopus). Users then read and rate abstracts according to community-determined appraisal criteria. The main advantages of eSRAP are the collaborative "filtering" of most relevant high quality studies and user-based customization. To identify the barriers and the facilitators associated with system use, we will use a mixed methods design, combining a prospective observational study with a qualitative multiple case study. Evaluation results will be used to improve eSRAP and will contribute to knowledge on research trend monitoring and literature surveillance tools. Finally, lessons learned may be transferable to other professional groups facing challenges associated with staying current.

Interprofessional Education in Action

Michelle L. Zafron
Coordinator of Reference Services, Health Sciences Library, Buffalo, New York

Amy G. Lyons
Interim Director, Health Sciences Library, Buffalo, New York

Nell Aronoff
Librarian and Liaison to the School of Medicine and Biomedical Sciences, Health Sciences Library, Buffalo, New York

Elizabeth Stellrecht
Clinical Librarian and Liaison to the School of Dental Medicine, Health Sciences Library, Buffalo, New York

Maryruth F. Glogowski
Associate Vice President for Information Services and Systems, Information Services and Systems, Buffalo, New York

Jeremiah Grabowski
Online Programs Coordinator, School Of Public Health And Health Professions, Buffalo, New York

Patricia J. Ohtake
Associate Professor, Department of Rehabilitation Science, Buffalo, New York

This lightning talk focuses on an experiment that tested the hypothesis that exposing health professions students to other professions will make them more ready to engage in collaborative patient care and lead to better shared decision-making in healthcare.
The investigators recruited fifty-four students from nine undergraduate and graduate health professions programs from two academic institutions to participate in the study. After completing some preparatory work, participants met in a technology-enhanced classroom designed to facilitate collaboration. They were given a hypothetical patient case, complex enough that it required the efforts of different professionals to develop a plan of care, basing their decisions upon their own clinical expertise, available published evidence, and patient values.

Using two measures (Adapted Fresno Test and Readiness for InterProfessional Learning Scale), participants' competence in evidence-based practice and readiness to engage in interprofessional collaborative care and were assessed three times: before and after completing the online-modules and after the interprofessional collaborative case discussion.

Librarians! Let’s Leverage Our Role to Raise the Quality of Biomedical Research

Heather Cunningham
Assistant Director, Research & Innovation Unit, Gerstein Science Information Centre, Toronto, Ontario, Canada

Erica Lenton
Faculty Liaison & Instruction Librarian, Gerstein Science Information Centre, Toronto, Ontario, Canada

Ana Patricia Ayala
Instruction & Faculty Liaison Librarian, Gerstein Science Information Centre, Toronto, Ontario, Canada

Shona Kirtley
EQUATOR Knowledge and Information Manager|Senior Research Information Specialist, Centre for Statistics in Medicine (CSM), University of Oxford, Oxford, United Kingdom

Objective: Reducing research waste through adherence to protocols and reporting guidelines is increasingly important in health science research. This presentation examines how librarians can leverage our participation on research teams to raise awareness of, and compliance with, reporting guidelines and protocols for biomedical research.

Brief Description: Dedication to research reproducibility is a key aspect of our library’s knowledge synthesis service. We see collaborating on protocol registration and publication as crucial. Incorporating protocol adherence in the early stages of synthesis studies will result in a more robust manuscript and will ease the journal submission process

Program Evaluation: A mixed methods approach of this long term study will measure protocol compliance, knowledge and experience of research teams regarding reporting guidelines.

Anticipated Outcomes: - Reproducibility of the knowledge synthesis model for other libraries/settings
- Librarians use their unique position in the knowledge synthesis cycle to raise awareness about reporting guidelines
- Open dialogue in the medical librarianship community on the ways they can positively impact the quality of biomedical research
- Determining incentives and barriers to reporting guideline compliance

- Demonstrating improved quality in the conduct and reporting of knowledge syntheses

"Is It a Fair Use?" Celebrating Fair Use Week to Promote Critical Thinking about Copyrights

Virginia Pannabecker, AHIP
Health, Life Science, and Scholarly Communications Librarian, University Libraries, Blacksburg, Virginia

Anita Walz
Open Education, Copyright & Scholarly Communications Librarian, University Libraries, Virginia Tech, Scholarly Communication Unit, Blacksburg, Virginia

Robert Sebek
Collections Technology Specialist, University Libraries, Virginia Tech, Blacksburg, Virginia

Scott Fralin
Exhibit Specialist and Event Operations Coordinator, University Libraries, Virginia Tech, Blacksburg, Virginia

Keith Gilbertson
Technology Development Librarian, University Libraries, Virginia Tech, Blacksburg, Virginia

Objective: Have you ever been asked, “Can I use this?” in reference to an article, book, image, or other copyrightable work intended for a course, publication, or professional training? This lightning talk presents one university library’s Fair Use Week celebration that built in training opportunities and self-directed learning tools to address such questions.

Methods: Learn about one public university library’s experience celebrating Fair Use Week 2016. The celebration focused on engaging students, staff, faculty, and the public in critical thinking activities related to U.S. copyrights and fair use. This lightning talk will provide a five-minute summary of the highlights, lessons learned, and event components: an exhibit, programming, and accompanying materials. A link to an online toolkit of event files and information will be made available for further exploration by interested participants. The toolkit will include items such as: handouts; exhibit designs and photos; program descriptions and materials; and example budget, equipment, and timeline.

Dare to Know: Sharing the Value of the Library

Susan Baer
Director of Libraries & Archives, Health Sciences Library, Regina, Saskatchewan, Canada

Objective: To raise awareness of the library's value to the organization to avoid cuts to the library's budget, a multi-faceted promotional campaign was designed featuring the library's new brand. Under the provincial library week theme, five champions shared their insights regarding the value of the library to their practice, providing excellent, impartial quotes for promotion with other clinical staff.

Methods:
Champions were selected based on frequency of library use or from large research projects. The campaign also included comments from the CEO regarding the library's alignment with the organization's goals. Interviews consisted of five questions of 15 minutes, which were taped to capture all comments. Quotes were selected to integrate into the library's rebranding project. During library week, the new pamphlet was launched, and five promotional messages were broadcast using digital signage, websites, posters, the organization's e-newsletter and email in an attempt to capture the attention of clinical staff. Statistics from previous research studies connecting the library's value to patient care reinforced the messaging. Snapshots of library statistics before library week were compared with statistics for the following two months. Along with the increase in usage, the advocacy component was an immeasurable benefit of the campaign.

Finding the Middle Ground Between a Web Specialist and the Wild West: A New Web Governance Model for an Academic Health Sciences Library

Susanna Galbraith
Virtual Services Librarian, McMaster University, Hamilton, Ontario, Canada

Stephanie Sanger
Clinical Services Librarian, Health Sciences Library, Hamilton, Ontario, Canada

Purpose: This project investigated current web governance practices within and outside libraries to develop an internal web governance model for an academic health sciences library.

Setting/Participants/Resources: Librarians in collaboration with the management committee at an academic health sciences library.

Brief Description: This project was initiated by the director of the library to solve existing issues with content management and web management at the library. This talk describes the process, final outcome successes and challenges encountered in developing a new web governance model. Web governance for this project has been defined as a framework for establishing accountability, roles and decision-making authority for an organization's web presence. Challenges we foresee with implementing the proposed governance model include working with existing organizational structures, change management and buy-in from library staff.

Results/Outcome: The new web governance model has been presented to the management committee at the library. The recommendations are being finalized for implementation.

Evaluation Method: The recommendations will be piloted for one year. Library staff will be surveyed before, during and again at the end of the year to assess the project's success.

Introducing Hospital Librarians to Clinical Data Management

Kendra Godwin
Public Service Assistant, UCLA Louise M. Darling Biomedical Library, Los Angeles, California

Objectives: A LibGuide was developed to provide information and resources about clinical data management for hospital librarians. This resource was designed to drive advocacy and conversation about translating information management skills beyond established library services into clinical data management, specifically data from electronic health records.

Methods: This project was developed in response to a regional data management needs assessment survey. Half of respondents reported that they hadn't addressed the data management needs at their institutions. Books, articles, presentations, and LibGuides on data and data management were examined to create this resource, with consideration given to relevant subject matter, introductory skill level, and ease of access. This talk examines how the topic of data is introduced to a beginner, how the hospital setting influences data management, and the skills and support needed for hospital librarians to develop clinical data management services.

Wednesday, May 18, 2016, 9:00 AM – 10:25 AM
Room: 202B

Session: Building and Maintaining Collaborations

Moderator: Mindy Thuna

An Evidence-Based Approach to Assessing Writing of Undergraduate Nursing Students' Leadership Capstone Papers RESEARCH

Xan Goodman, AHIP
Health & Life Sciences Librarian, Lied Library, Henderson, Nevada

Pamela M. Juniel
Doctoral Candidate, Lied Library, Las Vegas, Nevada

Cheryl Perna
Lecturer and Clinical Instructor, MSN, RN, School of Nursing, Las Vegas, Nevada

Rachelle Weigel
Library Technician II, UNLV Libraries, Educational Initiatives, Las Vegas, Nevada

Objectives: This research paper will describe undergraduate nursing student performance with selecting and citing reputable sources in capstone papers after receiving an intervention of library instruction. This paper will also describe how a health science librarian and nursing faculty member refined their instruction and support to improve the writing of baccalaureate nursing students.

Methods: An hour and a half information literacy intervention was delivered to improve students' information literacy skills through a library instruction session that included instruction on APA and active learning activities. This session was completed by all students at the start of the semester. Capstone papers (N = 95) were collected over six semesters from Fall 2012 to Summer 2014. Of those papers, 54 were pre-intervention and 41 were post-intervention. To evaluate the intervention, faculty examined the students' leadership

capstone papers using a rubric developed by the authors looking at adherence to APA (title page, in text citation and reference page) and reference quality (relevant journal/websites and external sources). The four team members scored the papers individually using the newly developed rubric. The team then compared scores for each paper, discussed any variations, and came to a consensus.

Results: To test inter-rater reliability the Intraclass Correlation with a two-way mixed model was used. The Intraclass Correlation Coefficient is .672 for all raters which demonstrated a moderate agreement. An increase in mean scores was found between pre-intervention (1.48) and post -intervention (2.39) APA title page. There was also an increase in mean scores found for pre-intervention (1.48) and post-intervention (2.23) APA in-text citations. There were no significant changes in mean scores for reference page, journal sources and external sources.

Conclusions: APA usage on the title page and in-text citations improved in post-intervention capstone papers. The researchers identified the reference page as a complex element of the capstone paper. Common errors on the reference page were identified and it is recommended to provide an additional instruction intervention to encourage students to approach the formatting of the reference page by: identifying one student to write the reference page, and at least two students to review the reference page.
Instruction was not provided for quality of journal sources and external sources. However, overall students selected quality references.Based on these findings, teaching practices will be altered. Revisions to the scoring rubric were also identified. Rubric revisions will enhance information about the use of APA. Our intervention helped to improve the scaffolding of information literacy skills throughout the curriculum. Future research should include an examination of scaffolding considerations over time.

Potential Opportunities and Collaborations for Library Engagement in Shared Decision Making: Results from a Global Survey RESEARCH

Lindsey Sikora
Acting Head, GSG and Social Sciences Library, University of Ottawa Library, Ottawa, Ontario, Canada

Melissa Helwig
Information Services Librarian, W.K. Kellogg Health Sciences Library, Halifax, Nova Scotia, Canada

Objectives: As physicians and patients move towards a collaborative process that allows them to make healthcare decisions together through shared decision making (SDM), where do libraries fit? The purpose of this study was to explore the roles of libraries within SDM. The results of our survey will be used to formulate a more comprehensive view of these roles within SDM.

Methods: A bilingual survey was distributed via social media and health library listservs, which included both structured and open-ended questions. These questions were based on models of SDM and the potential roles of libraries, and potential gaps identified from our scoping review. The survey questions were tested for clarity and content validity with the help of librarians, technicians and library students, previous to launch. Analysis will be focused on obtaining descriptive statistics to identify current trends between SDM and libraries, while elucidating the gaps between these two fields. The results will be used to formulate a more comprehensive view of the roles of information professionals in SDM.

Results:

The 113 responses from the survey were collected from a variety of settings, including academia, hospital and special libraries. Approximately 45% of the respondents felt they were doing activities that supported SDM in their library, while 31% felt they were not, and 24% were unsure. When asked about the potential roles for libraries in SDM, some suggestions were recurring, while others were newly established. Respondents who felt they were supporting SDM were able to clearly identify new roles and challenges to engaging in SDM.

Conclusions:

The results of this study indicate that librarians are already engaging in activities that support SDM, and many can identify roles for information professionals in supporting SDM for health care professionals and clinicians. The information collected in the survey will allow us to formulate a more comprehensive view of the roles that information professionals can undertake in SDM, along with the challenges they may encounter going forward.

Completing the Mosaic: Expanding a Health Information Specialists Program for Public Librarians

Phill Jo
Assistant Professor and Reference Librarian, Robert M. Bird Library, Reference and Instructional Services, Oklahoma City, Oklahoma

Shari Clifton
Professor, Head of Reference, and Associate Director, Robert M. Bird Library, Reference and Instructional Services, Oklahoma City, Oklahoma

Jean Longo
Medical Librarian, Robert M. Bird Library, Reference and Instructional Services, Oklahoma City, Oklahoma

Tara Malone
Assistant Professor and Librarian, Robert M. Bird Library, University of Oklahoma Health Sciences Center, Oklahoma City, Oklahoma

Objectives: The purpose of the Health Information Specialists program expansion is to introduce public library staff across the state to high quality consumer health information resources and to enhance strategic partnerships between public libraries and an academic health sciences library.

Methods:The Health Information Specialists program began as a pilot project in Fall 2013 for staff members in a large metropolitan public library system. Successful implementation in a single system over a two year period led to extending the program to public libraries throughout the state, with participants ranging from small municipal library settings to additional public library systems. Recruitment utilized a variety of methods including a presentation to the state's Public Library Director's Council and coordination with consultants at the state's Department of Libraries. Through the program, five classes approved for the Medical Library Association's (MLA) Consumer Health Information Specialization (CHIS) program were taught at five locations throughout the state from September 2015 through March 2016. This paper discusses the implementation process, program outcomes, unforeseen challenges, and recommendations for further applications.

Results: The majority of the program participants completed the requirements to achieve their Level I CHIS. The course evaluation analysis disclosed that participants received numerous benefits such as exposure to various authentic health information resources, enhanced knowledge, networking, information sharing, and continuous collaborative opportunities between libraries. The cumulative evaluation of the program is in process and the results will be presented at the meeting.

Conclusions: Even though this program requires a significant and ongoing investment of time for planning, coordination, and implementation, it is rewarding for both health sciences librarians and public library staff. Through the Health Information Specialists program, our health sciences library has strengthened relationships and laid the groundwork for future collaborations with public libraries throughout our state.

Library and Institutional Animal Care and Use Committee Partnerships: Successful Collaborations to Further Animal Welfare RESEARCH

Emily Mazure, AHIP
Biomedical Research Liaison Librarian, Duke University, Durham, North Carolina

Objectives: To examine and document the experience and knowledge of libraries and librarians who have long-standing relationships with Institutional Animal Care and Use Committees (IACUC) in order to document similar features of and best practices in library partnerships with their IACUC.

Methods: This is an IRB-approved, two-part, study involving a brief questionnaire, followed by an in-depth interview of academic librarians known to have standing relationships with their IACUC. The initial questionnaire will ascertain basic details about animal alternative literature searching practices and the relationship with IACUC. Responses will be analyzed and will guide the focus of subsequent in-person interviews. Part two of the study consists of conducting in-person interviews with selected IACUC-involved librarians from the initial survey. When possible, interviews will coincide with relevant, observable work or events, such as instructional sessions or literature searches. Data from the questionnaire, notes from interviews, and materials shared by hosting libraries will be analyzed for themes using NVIVO software. Commonalities and variations in the participant's relationships and approaches to searching will be explored.

Making Health Information Count: Impacting Health Care Through the Provision of Online Resources Statewide RESEARCH

Gail Kouame
Assistant Director for HEALWA, Health Sciences Library, Seattle, Washington

Tania Bardyn, AHIP
Associate Dean of Libraries & Director, Health Sciences Library, Health Sciences Library, Seattle, Washington

Objectives: The goal of this study was to analyze the impact of the provision of online access to evidence based health information on patient care, and to determine if there is value for health care providers statewide in having access

to online health information. Our research was modeled after Sollenberger and Holloway's study published in JAMA in September 2013.

Methods: Researchers administered an online survey to over 100,000 health care practitioners throughout the state of Washington regarding their use of and opinions about a statewide health information portal: HEALWA. The Value of Library and Information Services in Patient Care Study Facilitator Handbook served as a foundation for developing the survey questions and methodology. The survey was administered by an outside consulting firm, and launched in February 2015 and closed in April. Two reminders were sent to individuals who had not yet completed the survey before the closing date. Additionally, the Washington State Medical Association mentioned the survey in their email digest. Participants qualified to enter a random drawing to win one of five $100 Amazon gift cards. By the close date, over 7400 health care professionals had completed the survey. IRB approval was secured through the UW Human Subjects Division.

Results: Of the total respondents to the survey, 22% reported having used the HEALWA portal site, with the remaining 78% reporting having never used the online resources. Those who had utilized it, however, provided some useful information. Practitioners stated that the most frequent daily information searches they perform are for drug information , information for patients, diagnosis information, and adverse effects information. E--journals were reported to be the most frequently used resource across topics. The majority of HEALWA users (about 80%) report that access to the resources contributes to higher quality of care, provides clinical value, and results in better-informed decisions.

Conclusions: This study demonstrates that access to health information resources is valuable in a clinical setting when providers know about the resources and what information they can access. The primary challenge identified by study participants is the need to educate eligible users of the HEALWA portal site about the existence of the resources. Getting login credentials and navigating the site have proved challenging for some. The HEALWA team plans to follow up with focus groups comprised of survey respondents and to re-work the portal web site using feedback from this survey and focus groups. They also plan to increase promotion activities.

Wednesday, May 18, 2016, 9:00 AM – 10:25 AM
Room: 205B

Session: Library Operations: Adapting to Change

Moderator: Michael Garner

Revitalizing Library Services: Redefining and Maximizing Operational Efficiency and Excellence

Stevo Roksandic, AHIP
Regional Director, Mount Carmel Health System Library Services, Columbus, Ohio

Objectives: This paper describes how a regional multi-site operating health system and academic center, led by newly appointed library leadership, redefined operational efficiency by developing a new business model focused on synergizing staff and centralizing resources.

Methods: Facing 35% in staffing reduction, budgetary decrease and low staff engagements, library leadership has identified a critical need to restructure Library Services. Environmental scanning and analysis of collected data were major drivers for immediate change, implementation of innovative services and creating a vision for further development. That has created an opportunity to realize the vision to broaden Library Services by providing Consumer Health Services to patients and community. A three-year reconstruction period encompassed a transformation including organizational and reporting structure, collection management, technology, client-librarian communication conversions, optimizing physical space and marketing and outreach initiatives. Repositioning and aligning Library Services to organizational strategic initiatives was essential and has created additional opportunities to establish an innovative model of library support to the corporate health system across several states.

Results: Transforming Health Sciences Library services in the Health System Centralized three Library Businesses (i.e. Health Sciences Library, Consumer Health Library and Corporate Library Support) has moved Library services away from obsolesce towards vitality as a knowledge information center supporting medical professional staff at six operating locations, multi-operating sites of two

large academic entities, local communities and two corporate sites.

Conclusions: A resulting 72% staffing increase, almost double budgetary increase and innovation in providing services define today's Health System Library Services as exemplary in continuous improvement, operational efficiency and standards of service excellence.

Building Community: Engaging the 21st Century Library Staff Member

Jamie M. Gray, AHIP
Director for Research & Instruction, Lane Medical Library & Knowledge Management Center, Stanford, California

Terry Ann Jankowski, AHIP, FMLA
Assistant Director for User Experience, University of Washington, Seattle, Washington

Patrick V. Osby
Libraries Human Resources Director, Human Resources, Seattle, Washington

Objectives: According to Gallup, US employee non-engagement stands at approximately 68%. Engagement factors are multifaceted, but the areas of employee recognition and wellness are two foundational bedrocks linked to motivation. As part of the University of Washington (UW) - Health Sciences Library's strategic plan, our objective was to build a unit specific wellness and recognition program for all staff.

Methods: Led by select members of the Health Sciences Library (HSL) management team, the library undertook efforts to uncover the general needs and interests of staff at all levels. Data gathering was conducted in two phases. The first phase consisted of hour long focus group sessions with library departments. Phase two asked all staff to fill out a survey regarding their interests and preferences around wellness and recognition. The collected input was then used to develop a multi-layered program targeting improvements in employees' feelings regarding these areas.

Results: Based on staff feedback, the areas of recognition and engagement became the focus for the first round of programming. Targeted activities to enhance community development were implemented at both the individual and group level. Activities included cross departmental thank you notes from members of the management team, creation of a staff game collection, and random Friday "pop"contests. Future activities include crafty lunches and the establishment of an expanded staff Community Engagement Team.

Conclusions: Although in its infancy, initial response to the program has been positive. The UW Libraries larger peer-to-peer recognition program is still widely used across the Health Sciences Library. However, there has been sustained interest and participation across the various activities HSL has developed. Creating and maintaining a departmental level program requires commitment and identification of champions at both the management and staff levels. Long-term planning is needed to ensure program sustainability and responsiveness. Program evaluation should happen periodically to help realign activities as the environment changes.

Training a New Librarian in the What, How, Where, and Why of Health Sciences Collection Management

Susan K. Kendall, AHIP
Health Sciences Coordinator, Michigan State University Libraries, East Lansing, Michigan

Mari Monosoff-Richards
Health Sciences Librarian, Michigan State University Libraries, East Lansing, Michigan

Objectives: : Health sciences collection management is an increasingly complex job. Newly graduated librarians report little exposure to this role, and few current materials exist to help supervisors train new collections librarians. A detailed training program was developed to teach a newly graduated librarian to manage an extensive clinical medicine collection serving medical schools. Details of the program will be discussed.

Methods: : Book chapters and articles on health sciences collections management were consulted for context but did not provide enough information for a new librarian to begin making selections. Building on general materials about training librarians, supplemented with experience particular to the health sciences, the supervisory librarian developed a step-wise training program which focuses on learning by doing and moves from specifics to general principles, rather than the other way around. Decision-making for selection of materials was approached from multiple angles: institutional analysis, subject analysis, and publisher and vendor knowledge. The new librarian has been working through the training program, focusing primarily on monograph selection and making selection decisions.

Results: As the new librarian worked through the training program, her feedback helped evaluate the program and allowed the supervisor to revise the steps and better understand the learning process. She reported that learning

about institutional strengths, to use tools like Doody's, and to organize decisions by publisher and platform rather than medical subject area were keys to her progress. She has been able to confidently make appropriate purchase decisions. Some steps of the training program, particularly the assignment to read and learn the collection development policy, were found to be less useful until more knowledge was gained.

Conclusions: Training a new librarian in health sciences collections development needs to be approached as a long term project and is challenging. An organized approach and feedback from the trainee are important to ensure that key concepts are not missed as the librarian works towards more independence. While the program was developed with the idea that the new librarian would focus primarily on collections, it was found that, without a liaison component, learning about the needs of the medical school did not happen as naturally as hoped. The training program as developed could be adapted by supervisors at other institutions, however, more thought is also needed to determine how best to integrate liaison activities with collections activities to maximize the development of new librarians.

Envisioning Health Information Science: Critical Reflections on an Interdisciplinary Identity RESEARCH

Nicole K. Dalmer
PhD Candidate, Library and Information Science, Faculty of Information and Media Studies, The University of Western Ontario, London, Ontario, Canada

Eugenia Canas
PhD Candidate, Faculty of Information and Media Studies; Faculty of Health Sciences, London, Ontario, Canada

Lyndsay Foisey
PhD Candidate, Faculty of Information and Media Studies; Faculty of Health Sciences, Mississauga, Ontario, Canada

Brad Hiebert
PhD Student, Faculty of Information and Media Studies; Faculty of Health Sciences, London, Ontario, Canada

Anthony Naimi
PhD Candidate, Faculty of Information and Media Studies; Faculty of Health Sciences, Cambridge, Ontario, Canada

Sadiq Raji
PhD Candidate, Faculty of Information and Media Studies; Faculty of Health Sciences, London, Ontario, Canada

Shamiram Zendo
PhD Student, Faculty of Information & Media Studies; Faculty of Health Sciences, London, Ontario, Canada

Jill Veenendaal
PhD Candidate, Faculty of Information and Media Studies; Faculty of Health Sciences, London, Ontario, Canada

Objectives: As an evolving field related to and impacting the health, information and library sciences, Health Information Science (HIS) presents emergent and varied considerations for researchers, policymakers, health services providers and information professionals. This paper describes the iterative and reflexive process in identifying and questioning conceptual underpinnings, thematic tensions and methodological approaches currently delineating the field of Health Information Science, with considerations given to future HIS trajectories and configurations. Methods: To explore the identity of the HIS field, a group of doctoral students affiliated with an HIS program at a Canadian academic institution embarked on a process of delineation and description. This iterative and critically reflexive process of honing a definition through discussion necessitated multiple voices, given the relative newness of the field and the few programs dedicated to its study. Over a series of discussion periods, they collaboratively questioned existing conceptualizations of the field, consulting with and integrating available literature, and drawing from their own situatedness and experiences as researchers and practitioners.

Results:The heterogeneity of approaches inherent in HIS resulted in an examination of institutions, technologies, people and information in relationship to health information in health care systems, practices and consumption. Each of the members in the group contributed their own experiences and perspectives to an iterative and ongoing dialogue that revealed lingering questions regarding the fundamental identity of health information science as a cohesive discipline. Instead of coming to a definitive answer, the authors acknowledge key questions that must be asked and grappled with as this field continues its development. The multiple voices that created this paper advance the importance of reflexivity among practitioners and scholars, and encourage those that join this much-needed conversation to be comfortable with conflict and tensions as this disciplinary culture is slowly unearthed.Conclusions: This paper details the output of a conversational community's micro-experiences and findings in delineating an emergent field. Committing to continued and reflexive dialogue, this paper advances a unique lens for inquiry and knowledge generation. The questions posed throughout this paper underscore the overall potency of pluralism, affirming that a diversity of coexisting epistemologies,

methodologies and methods within the HIS field make for rich ground for its growth.

Evaluating a Health Sciences Librarianship Assistantship for Library School Students Interested in Serving Latino and Native American Communities RESEARCH

Sanjana Quasem
MPH Candidate, Department of Behavioral & Community Health, School of Public Health, College Park, Maryland

Gale Dutcher, AHIP
Deputy Associate Director, National Library of Medicine, Bethesda, Maryland

Janice E. Kelly
Chief, Outreach and Special Populations Branch, Specialized Information Services, Bethesda, Maryland

Alla Keselman
Senior Social Science Analyst, Specialized Information Services, Bethesda, Maryland

Objectives: A major federal medical library provides a health sciences library of a large state university with funding towards graduate assistantship for the university's School of Library Science students wishing to focus on serving Latino and Native American populations. This study evaluates the impact of the assistantship on the recipients' career trajectories and relevant health librarianship expertise.

Methods: This qualitative evaluation involved structured interviews with 13 of 22 program graduates over 11 years. Questions focused on participants' pre- and post-assistantship interest in health librarianship and understanding of the issues unique to Latino and Native American populations, as well as on their subsequent career trajectories. The interviews investigated how various components of the assistantship program, including mentorship, professional development and networking opportunities, affected the outcomes. Interviews were transcribed and entered into NVivo software for analysis. The ongoing analysis focuses on data-driven discovery of repeating themes. Expected results are based on preliminary analysis, which suggests that most participants enter the program with little knowledge of health librarianship prior to the internship and come to developed strong appreciation for it. While not all become health librarians, nearly all provide health information. Strong

mentorship and MLA attendance appear to enhance the experience.

Wednesday, May 18, 2016, 9:00 AM – 10:25 AM
Room: 201F

Session: Analyzing the Literature

Moderator: Suzanne L. Fricke

Enhancing Understanding of a Research Discipline Through the Use of Text Mining Analyses RESEARCH

Jane Yatcilla
Health & Life Sciences Information Specialist, Veterinary Medical Library, West Lafayette, Indiana

Jennifer McDaniel
Research and Education Librarian, Tompkins-McCaw Library for the Health Sciences, Richmond, Virginia

Objectives: "Mapping the literature" studies provide librarians with valuable insights into the information needs of researchers, especially the types of source materials these researchers use for their work. The objective of this study was to determine how text mining techniques can add to the understanding librarians have developed of disciplinary researchers based on other bibliometric analyses such as mapping the literature.

Methods: To understand how the results of a text mining analysis could complement the knowledge gained from a mapping the literature study, we compiled a data set similar to a previous mapping study, limiting the included articles to the same three source journals and date range. We used VantagePoint text mining software to examine this data set for informative patterns by analyzing: the trends of keyword/phrase associations found across titles, abstracts, and indexing; patterns of collaboration (author-to-author, institution-to-institution, country-to-country); and the degree to which the research conducted was interdisciplinary. Further text mining was conducted by adding to the data set relevant articles that were published outside the three journals targeted in the mapping study during the same time period to demonstrate the scalability of text mining software while augmenting the results derived from our first text mining iteration.

Results: VantagePoint software made it easy to determine additional information about healthcare chaplaincy research that was not apparent in the original

mapping study. This includes top authors, patterns and frequency of research collaborations, insight into the institutions and countries most often represented, and information about funding agencies. Further, while a few topics did emerge as most frequently studied, such as pastoral care and spirituality, the research of health care chaplaincy is varied, and in the three years studied the articles covered a myriad of subjects as well as clients from every phase of life, from infancy to old age.

Conclusions: Discussion will address the benefits, and limitations, of using both text mining software as well as the output from multiple bibliographic databases. For example, VantagePoint made short work of extracting specific data from fields that would have been difficult to do in an Excel spreadsheet, and VantagePoint can create useful visualizations with a couple mouse clicks. However, files of bibliographic citations and abstracts can require considerable amounts of time for data clean-up before analyses can be run, and different databases export data in different formats and do not always export all of the same fields, so merging data from multiple databases is not a trivial task.

A Descriptive Review of Randomized Controlled Trials and Systematic Reviews Published in Veterinary Medicine Journals RESEARCH

Lorraine Toews
Librarian, Veterinary Medicine, Bachelor of Health Sciences, University of Calgary Health Sciences Library, Calgary, Alberta, Canada

Objectives: Randomized controlled trials (RCTs) and systematic reviews are regarded as the gold standard of evidence for clinical interventions in veterinary medicine, yet few published studies quantify or describe the nature of RCTs and systematic reviews in the veterinary medical literature. The purpose of this descriptive review was to fill this gap and identify trends in the published literature.

Methods: Using a subset of veterinary journal titles from the Basic List of Veterinary Medical Serials, third edition (2010) by Ugaz AG, Boyd CT, Croft VF, Carrigan EE, Anderson KM, articles published at 4-year intervals over a 16 year period were located by searching OVID MEDLINE using a combination of MEDLINE publication types and title-abstract words to filter. Retrieved articles were classified according to number published per year in each journal, species studied and purpose of the study (ie. treatment, prevention, diagnosis). Publication patterns and trends were then identified in each of these areas.

Results
The number of RCTs and systematic reviews (SRs) in the study sample

increased significantly from 2000 to 2015, but there was considerable variability in the number published from year to year with RCTs ranging from 174 published in 2001, to 818 published in 2012, and SRs ranging from 6 published in 2001, to 78 published in 2013. Both RCTs & SRs were a very small proportion of the total literature in the study sample: RCTs were 3.68% and SRs were 0.24% of the total respectively. The majority of RCTs and SRs in the study sample were concentrated in three journals within each of three subject categories: RCTs in the top 3 subject categories of Food Animal, Internal Medicine and Microbiology & Immunology accounted for 52% of the total number of RCTS in the study. Systematic reviews in the same 3 subject categories accounted for 73% of the total number of systematic reviews in the study.

Conclusions: Randomized controlled trials and systematic reviews in the sample of veterinary literature studied have increased in number significantly in the past fifteen years, but they are a very small proportion of the veterinary literature and are concentrated in relatively few journals and subject areas.

Bibliographic Identities and Linked Data: An Investigation of Title Ambiguity in the Scientific Literature RESEARCH

Dick R. Miller
Director for Resource Management, Lane Medical Library, Stanford, Stanford, California

Thea S. Allen
Metadata Librarian, Lane Medical Library, Stanford, Stanford, California

Joanne Banko
Metadata Transformation Librarian, Lane Medical Library, Stanford, Stanford, California

Ariel Vanderpool
Resource Acquisitions Specialist, Lane Medical Library, Stanford, Stanford, California

Ryan M. Steinberg
Software Developer, Lane Medical Library, Stanford, Stanford, California

Objectives: To assess titles' uniqueness and role in a linked data environment, we analyzed the degree and types of ambiguity occurring in large sets of scientific bibliographic data. Comparison of the effect of various title addenda in reducing or eliminating identified ambiguity may have implications regarding choice of title, citation practice, and metadata policies for cataloging and indexing.

Methods: This bibliometric study measured degree of title ambiguity in: 1. PubMed, ~850 thousand titles published in 2012 (omitting titles with only translated titles) 2. NLM Catalog, ~1.5 million titles, using "title proper" (title subfields plus subtitle) and uniform titles, and appending date(s) 3. Lane Medical Library Stanford Catalog, ~300,000 titles, using title proper plus normalized enumeration, chronology and format. We used an algorithm to convert edition statements to normalized enumeration.

Sequence (using NCBI's eUtilities, UNIX, Excel and programming tools like Java): 1. Extraction of data from the selected sources 2. Consideration of differences in punctuation, capitalization, spacing, initial articles, etc. 3. Running UNIX utilities to determine quantity of multiple title occurrences 4. Comparison of results of the degree of ambiguity found considering dates, numbering (in case of Lane Catalog), etc. 5. Assessment of ambiguous titles to determine any patterns of occurrence.

Results: PRELIMINARY 1. PubMed: 99% of titles were unambiguous, with 25% of the ambiguous titles being generic types of publication, e.g. Editorial. Of the remainder, many appeared to be columns or series. 2. NLM Catalog: 87% of titles were unambiguous, with 69% of the ambiguity occurring in book titles. 14% of titles included edition statements. 3. Lane Catalog: 81% of titles were unambiguous, with 73% of the ambiguity occurring in books. 12% contained edition statements (98% enumerative rather than named).

Appending chronology to titles in Lane data reduced ambiguity to 2%. Enumeration provided a negligible improvement over chronology alone. (The effect of enumeration alone for Lane and NLM catalogs and chronology for the NLM Catalog results using the Lane algorithm is pending.)

Conclusions: PRELIMINARY. Cataloging data contains more title ambiguity than in indexing data. Chronology reduces ambiguity impressively. Lane's algorithm was effective in detecting enumeration from textual strings. Recording titles, enumeration and chronology data more formally has potential for improving bibliographic data display/manipulation and indexing. Ambiguity can be automatically reduced sufficiently to allow manual resolution of remaining cases.

Wednesday, May 18, 2016, 9:00 AM – 10:25 AM
Room: 202D

Session: Clinician & Clinical Support

Canadian Academic Health Sciences Libraries and Their Relationships with Health Care Practitioners RESEARCH

Orvie Dingwall, AHIP
Head Outreach Services, University of Manitoba Health Sciences Libraries, Winnipeg, Manitoba, Canada

Trina Fyfe
Northern Health Sciences Librarian, Northern Medical Program- Geoffrey R Weller Library, Prince George, British Columbia, Canada

Objectives: The purpose of this research is to explore the relationships between academic and healthcare libraries in Canada and their provision of resources and services to healthcare providers. The objectives are to discover the relationships between academic health sciences libraries and healthcare practitioners, how consortia impact these relationships, factors that impact relationship development and sustainability, and methods of evaluation.

Methods: This study will use a qualitative grounded theory approach utilizing semi-structured interviews and focus groups with key stakeholders from Canada's 17 academic health sciences libraries, and 10 Canadian health science information consortia. Interviews will be conducted until themes emerge. Focus groups will be conducted with key stakeholders to provide them with the results of the interviews and the opportunity to be involved in the analysis. Interview transcripts and focus group notes will be coded using open and axil coding.

Results: Based on our preliminary investigations we hypothesize that there are commonalities in these relationships that have not yet been defined. We believe that these relationships are being evaluated, both formally and informally, and that identifying best practices in relationship evaluation will be beneficial to the Canadian health library community.

Conclusions: Our findings will identify relationship development strategies and future directions for relationships amongst health science libraries and healthcare practitioners. Identifying best practices will assist libraries in

information exchange, supporting vibrant programs of collaboration and engagement, and best methods for evaluation.

Examining the Pieces: Usability Testing to Improve Outreach to Unaffiliated Health Professionals RESEARCH

Emily J. Hurst, AHIP
Head, Research and Education, Tompkins-McCaw Library for the Health Sciences, Richmond, Virginia

Irene Machowa Lubker
Research and Education Librarian, Tompkins- McCaw Library for the Health Sciences, Richmond, Virginia

Barbara A. Wright, AHIP
Project Librarian, Tompkins-McCaw Library / Research & Education, Richmond, Virginia

Objectives: In order to better address the information needs of unaffiliated public health and primary care professionals, librarians undertook a project to strengthen usability and refine the content of a freely available website maintained by the library. Collaboration with an advisory committee of local and state health care professionals allowed librarians to schedule focus groups and usability testing with diverse groups.

Methods:

While a freely available, library maintained website had a long history, usability testing had not been done after a platform upgrade. Outside funding provided library staff with resources to engage with community groups focused on supporting public health and primary care professionals. An advisory committee of stakeholders was formed to support the project. Both qualitative and quantitative approaches, including focus groups and usability testing, were used to collect and analyze data. Focus groups provided feedback on usability as well as suggestions for content. Feedback was transcribed to provide effective information for website updates and regularly shared with the advisory committee for review and input. Project librarians further researched content suggestions. Website content and navigation will be updated based on feedback and reviewed by the advisory committee. Other funding opportunities will be sought to further publicize an updated website.

Results:

Feedback from multiple focus groups provided project librarians with

information about how practitioners used the website. Feedback exposed issues related to the website that could easily be corrected and others that would need more in depth work. In addition, feedback on content demonstrated the need for resources on mental health and other topics. Usability testing was limited but structured task completion questions provided an overview of navigation and general design flaws that needed to be addressed. Preliminary consultations with library web designers generated an action plan for improving site design and navigation. Most focus group sessions concluded with a brief training period in which project librarians answered questions from participants about the site and demonstrated how to use site resources.

Conclusions:

Project librarians gathered valuable information using the methods described. This was the first time the website had undergone any type of user testing and the project librarians concluded that further iterations of the site should be tested by practitioners. Unexpected benefits of the project include relationships built with practitioners through focus groups and the advisory committee. Future funding will be sought to finalize website improvements and begin efforts to train and promote the use of the site.

The Academic Health Sciences Library and the Accountable Care Organization (ACO): New Opportunities for Traditional Clinical Service

Andrea L. Ball
Care Management and Population Health Librarian, Health Sciences Library, Seattle, Washington

Frances Chu
Health Sciences Collections Manager and Clinical Liaison, Health Sciences Library, Seattle, Washington

Tania Bardyn, AHIP
Associate Dean of Libraries & Director, Health Sciences Library, Health Sciences Library, Seattle, Washington

Ann Whitney Gleason
Associate Director, Resources and Systems, University of Washington, Seattle, Washington

Objectives: In response to changes in healthcare, this paper will detail strategies being implemented at the University of Washington's Health Sciences Library to align and modernize the library's clinical librarianship program with the very new care delivery model in the healthcare system.

Methods: This is a case study of a new position and program in a large academic health sciences library. The goals of the position and program are to 1) develop non-traditional relationships; 2) redesign the clinical librarian program; 3) support the organization as it transitions into an Accountable Care Organization; and, 4) share insights and knowledge via a conference with health sciences library professionals. This study will examine the utility and level of integration of the clinical library program into and in support of the organization's transition into an Accountable Care Organization and the Triple Aim approach to healthcare reform. Additionally, the results of the post-conference assessment will be presented.

Results: As the University of Washington transforms into an Accountable Care Organization several opportunities for the Health Sciences Library's Clinical Services program have presented themselves, including several enhancements to our library services. We endeavored to answer the question "How can we (the clinical librarians) collaborate with you (the clinical professionals) to support the current and future transformations in healthcare?" Through the development and delivery of the Forum and the conversations held with the focus groups, the Library was able to recalibrate its clinical services program to better meet the needs of the research, teaching and clinical operation of the organization.

Conclusions: The University of Washington Health Sciences Library's clinical librarians believe that with this assessment and forum, any investment in collaboration by the libraries and its parent organization will be a worthwhile endeavor. Using this qualitative assessment and cross-regional forum, the clinical librarians will identify opportunities for librarians to teach, support research and the clinical services of the organization in addition to re-defining the clinical librarianship program at University of Washington, and further develop the Care Management and Population Health Librarian position to support the organization's move towards becoming an Accountable Care Organization and achieve the Triple Aim.

The Writing Club: Promoting Writing for Publication for Junior Doctors

Pip Divall
Clinical Librarian Service Manager, Education Centre Library, Leicester, England, United Kingdom

Objectives: To promote writing and publishing case reports, audits, reviews and systematic reviews in a university teaching hospital. The writing club aligns with the Trust's objective to become a centre of excellence for research. It is run as a joint enterprise by the Clinical Librarian Service and two consultant physicians.

Methods: The Writing Club was originally begun in 2009 and ran until 2010. It was revived with the assistance of a consultant physician in Stroke Medicine in 2015, to promote the writing of articles for publication. Regular bi-monthly meetings are held on a variety of topics, hosted by experts in the field of case reports, audit, referencing, systematic reviews and statistics, followed by an open discussion on topics the attendees bring. These are complemented by open review sessions in which attendees can bring their own writing for critique and support by library staff and clinical colleagues. Sessions so far have been well attended, and have led to the promotion of services the library provides to would-be authors.

Results: The Writing Club has been well attended, depending upon the topics up for discussion at each session. There is enthusiasm from senior medical staff about supporting the group, and doctors have responded well to invitations to speak at the Writing Club.

A blog has been set up for the group, to promote the sessions, provide a resource for sharing information and hopefully promoting discussion of writing for publication.

A separate library training workshop has been established on "Writing for Publication" to provide an introduction for less confident would-be authors, and this is used to promote membership of the Writing Club.

Conclusions: We have not yet seen if the Writing Club has led to higher levels of publication of research in the organisation. Further research is necessary to establish if the Writing Club has contributed to the organisation's research profile.

Integrating Information Literacy Through a Biomedical Informatics Course at the National Autonomous University of Mexico Faculty of Medicine in Mexico *CANCELLED*

Alejandra Martinez del Prado
Medical Reference Librarian, Facultad de Medicina, Universidad Nacional Autónoma de México, Distrito Federal, Mexico

Luis Fernando Kieffer Escobar
Pediatric Surgeon, UNAM Biomedical Informatics Department, Distrito Federal, Mexico

Sandra G. Moncada Hernandez
Head librarian, Instituto de Fisiología Celular, Universidad Nacional Autónoma de México, Ciudad de México, Mexico

Objectives: Information-based decision making is a necessary skill for health professionals. With the emergence and continuous development of Information Technologies the challenge has turned from finding information to finding quality information. This paper examines the integration of information literacy through a biomedical informatics (BMI) course at the National Autonomous University of México (UNAM) Faculty of Medicine in Mexico and outlines the advantages that students have with this course during their professional career.

Methods: The UNAM Faculty of Medicine, one of the largest medical schools in Latin America, developed and implemented a biomedical informatics mandatory course for two years (BMI-1 and BMI-2). Information literacy components were included in BMI-1 according to the Association of American Medical Colleges and the International Medical Informatics Association recommendations on biomedical informatics education. BMI-1 has the unit 'Databases and medical digital libraries' which goal is to know the main biomedical information resources, apply and use the strategies and tools needed for their effective use. Librarians joined the health care team and have teaching roles with physicians since the BMI course started and implemented strategies and didactic tools to achieve this goal. Information searching skills of students have been observed and evaluated while they are in BMI-2 and when they use the Faculty of Medicine library in person and remotely.

Results: BMI-1 students were evaluated during the semester with two departmental examinations and a final grade for the whole generation was obtained (for all BMI-1 groups). The 2015 generation evaluated in the two sessions addressing the library and information literacy themes obtained the minimum passing score. While students are coursing BMI-2, they are familiar with the information searching topic, however it is notable that not all of them retain the basic concepts studied in BMI-1 and it is still difficult for them to implement search strategies revised BMI-1. At the library, staff has started to notice that undergraduate students who have taken the BMI courses have basic idea regarding information searching skills, and at least are aware of the electronic resources available for them, in contrast to students who did not take the BMI courses.

Conclusions: The creation of the Biomedical Informatics department and the implementation of BMI courses, since six years ago, has had a positive impact for the benefit of integral formation of the school students. Information literacy integration during the courses provides them the opportunity to acquire knowledge, skills and attitudes needed for the searching, identification, analysis and effective application of biomedical information during their medical practice.

Nevertheless, in order to have more satisfactory scores, there is still a lot of work to do during classes. Librarians have a lot more to do, working within multidisciplinary teams. Continuous training is needed for teachers involved in the BMI courses.

Wednesday, May 18, 2016, 9:00 AM – 10:25 AM
Room: 203B

Session: The Future of Global Public Access: Will Public Access to the Medical Literature Grow?

The Future of Global Public Access: Will Public Access to Medical Literature Grow?

Kacy Allgood, AHIP
Research and Community Engagement Librarian, Ruth LIlly Medical Library, Indianapolis, Indiana

Kathryn Funk
Program Specialist, National Library of Medicine

Jayne Marks
VP, Global Publishing, Wolters Kluwer

Alicia Wise
Directory of Policy & Access, Elsevier

Lee-Anne Ufholz
Director, Health Sciences Library, Ottawa, Ontario, Canada

90 minute panel presentation. (English Language) A panel of 4 experts will present and answer questions on the similarities, differences and international impact of The Tri-Agency Open Access Policy and NIH Public Access Policy in the United States, Canada, and developing nations. Q & A session follows presentations.

Invited speakers will address the following questions:

a. What are the barriers to realizing global public access to medical literature?
b. What if public access ends? What is at stake for libraries, publishers, communities and research organizations?

c. How would the growth of global public access impact publishers, societies, readers, and libraries in the near and distant future?

Wednesday, May 18, 2016, 9:00 AM – 10:25 AM
Room: 104C

Session: The Data Discovery Bootcamp Challenge!

Moderator: Lisa M. Federer

The Data Discovery Bootcamp Challenge!

Lisa M. Federer
Research Data Informationist, National Institutes of Health Library, Bethesda, Maryland

In this unique, hands-on session, librarians will learn about resources to help them locate freely available data from National Center for Biotechnology Information (NCBI) resources, including BioProject, GenBank, and more. In the first part of the session, invited speaker Ben Busby of NCBI will introduce users to resources for locating datasets and discuss how datasets can be useful to a wide variety of library users, including researchers, clinicians, faculty, and students. In the second half of the session, attendees will have the chance to compete in "data discovery challenges." In small groups, attendees will work to come up with a solution to real-world problems using the resources they've just learned about, as well as other resources they may be familiar with. Groups will present their solutions and judges will determine a winner for each round based on effectiveness and originality of their solutions. Attendees are encouraged to bring mobile devices or laptops to access the resources in order to complete the challenge, thus giving them hands-on practice with using resources in a non-library setting, as they would likely do with their users.

Wednesday, May 18, 2016, 9:00 AM – 10:25 AM
Room: 203D

Session: Expert Searching

Moderator: Erla Heyns

Developing Search Hedges for MEDLINE/PsycINFO Searches on Aboriginal/Native American Peoples RESEARCH

Sarah Bonato
Reference and Research Librarian, CAMH Library Services, Toronto, Ontario, Canada

David Lightfoot
Librarian, Library, Toronto, Ontario, Canada

Objectives: Both MeSH and PsychInfo thesaurus terms delineate Native Americans/First Nations peoples from the antiquated, yet persistent misnomer Indians. Still these terms do not obviate the needs for a sensitive and thorough search filter for any aboriginal population. A systematic review, and even a through search, needs to take into competing nomenclatures, whereby different nations use the different terms for the same people. Our objective is to develop a highly sensitive search filter for systematic review searching in Medline and PsycINFO to identify articles relevant to Native American/First Nations/Aboriginal populations.

Methods: Individual search filters were developed for both Medline and PsycINFO. Separate search filters were designed to be highly sensitive for use in systematic reviews or to be specific to quickly capture the most relevant citations. The highly sensitive search filter was designed for maximum retrieval, since systematic reviews on Native American/First Nations/Aboriginal populations should include research from a large evidence base. The search filters were revised and tested in each database individually. The strategies were also tested in a number of limited searches to compared with a list of previously known publications to ensure retrieval validity. The search filters were also submitted to other health science librarians and researchers for peer reviewed.

The search filters were made freely available in Medline and PsycInfo. A follow up will be done to analyze use of the search filters over time.

Sensitivity of a Search Filter Designed to Identify Studies Reporting Health State Utility Values RESEARCH

Julie M. Glanville
Associate Director, York Health Economics Consortium Ltd, York, N/A, United Kingdom

Mick Arber
Information Specialist, York Health Economics Consortium Ltd, York, United Kingdom

Sonia Garcia
Information Specialist, York Health Economics Consortium Ltd, York, United Kingdom

Thomas Veale
Information Specialist, York Health Economics Consortium Ltd, York, United Kingdom

Objectives: The importance of quality-adjusted life years (QALYs) in healthcare decision-making means there is a need for methods which facilitate the effective and timely identification of studies reporting Health State Utility Values (HSUVs). The objective of this study was to use the relative recall (RR) method to test the sensitivity of the three search filters.

Methods: We identified a sample of systematic reviews (SRs) of studies reporting HSUVs by searching MEDLINE via Ovid (2004 to date) for SRs and by identifying 10 Manufacturers' Submissions (MSs) for NICE Single Technology Assessments which had conducted reviews of studies reporting HSUVs. The title and abstracts of records were screened by two researchers. The search methodology of each SR was quality assessed using a pragmatic checklist and SRs were rejected if they did not fulfil all the criteria. From the selected SRs and MSs, eligible studies formed the quasi-gold standard (QGS) set of studies reporting HSUVs. The perfomance of three search filters was assessed in terms of finding those QGS records. Perfomance was measured in terms of the sensitivity of the filters and their precision.

Results: 1,485 potential SRs were identified in MEDLINE. Full text documents were sought for 132 SRs. 88 SRs were obtained and 10 had search strategies which fulfilled the quality checklist criteria. From these 10 SRs and the 10 MSs, 346 citations for studies reporting HSUVs were extracted. After de-duplication 327 records formed the QGS set. 293 of these records could be identified in Ovid MEDLINE. Each of the search filters retrieved 267 of the 293 QGS records in MEDLINE, giving a RR of 0.91 for each. The highest precision achieved was

0.003.

Conclusions: High sensitivity search filters for HSUVs are available. The low precision of the filters is likely to improve when the filters are used in searches for specific conditions. Further analysis of the 26 records missed by the search filters will be undertaken to see if it is possible to improve sensitivity without significant loss in precision. We also plan to validate the revised filter on a further QGS set.

The Development of Highly Sensitive Search Filters for Complementary and Alternative Medicine-Specific, Natural Therapies for Use in Integrative Oncology RESEARCH

Becky Skidmore
Information Specialist, Skidmore Research & Information Consulting, Gloucester, Ontario, Canada

Renee Lang
Naturopathic Doctor, Naturopathic Medicine, Portland, Maine

Doaa Abdelfattah
MPharm Senior Research Assisstant, oncANP, Ottawa, Ontario, Canada

Heather L. Wright
Naturopathic Doctor, Naturopathic Medicine Department, Bala Cynwyd, Pennsylvania

Dugald Seely
Founder & Executive Director, Ottawa, Ontario, Canada

Anne Thiel
Naturopathic Doctor/Private Practice, Goshen, Indiana

Linlu Zhao
Research Fellow, Ottawa Integrative Cancer Centre, Ottawa, Ontario, Canada

Jen Green
Research Director, Bloomfield Twp, Michigan

Objectives: To develop highly sensitive filters in MEDLINE and Embase for CAM-specific, natural therapies (dietary, supplemental, intravenous, physical and mental/emotional) relevant to oncologic medicine. Systematic searches using these filters will be run sequentially for cancer topics by tumor type and

symptom (e.g., breast cancer, peripheral neuropathy, thoracic cancers), from which we will write structured research summaries on all identified therapies.

Methods: We used PubMed's dietary supplement subset as our base. We consulted with naturopathic doctors to identify and add vocabulary for further concepts. Building on previous work wherein a line-by-line version of PubMed's CAM subset was created in OVID MEDLINE, we developed a MEDLINE version of the expanded dietary supplement subset. We translated and adjusted this strategy for use in OVID Embase. Beginning with breast cancer, we ran multi-file searches and removed duplicates in OVID to reduce the deduplication burden. We compiled and circulated screening packages in Reference Manager. After tagging potentially relevant records, we exported them to Mendeley for additional tagging and full-text retrieval. We created a structured template for each relevant trial, including citation, study design, population, intervention, comparator, outcomes, side effects and interactions, to be used to present peer-reviewed data about human trial natural therapies in oncologic medicine.

Results: We retrieved 54,061 records on CAM-specific, natural therapies in breast cancer. After deduping in OVID followed by further deduplication in Reference Manager, we reduced the number to 44,383. From this we identified and tagged 827 potentially relevant records and another 370 as "maybe" potentially relevant. We have finalized the tagging template from which we will derive our peer-reviewed evidence summaries. Work is ongoing and we will further report developments as they occur.

Conclusions: Our customized CAM-specific, natural therapies filter has allowed us to efficiently and systematically identify evidence-based CAM material in MEDLINE and Embase for breast cancer. Relevant records have been tagged and will be developed into electronic evidence-based summaries. Our experience with breast cancer will allow us to fine-tune processes for further high-priority topics in integrative oncology. Translating these filters to additional databases (e.g., PsycINFO, CINAHL, AMED) will also be explored.

The Yale Medical Subject Headings (MeSH) Analyzer: A New Tool for Search Refinement

Holly K. Grossetta Nardini
Research and Education Librarian, Cushing/Whitney Medical Library, New Haven, Connecticut

Lei Wang
Instructional Design Librarian, Cushing/Whitney Medical Library, New Haven, Connecticut

Objectives: When conducting a systematic search, it is critical to design a search strategy that retrieves all potentially relevant articles. Even experienced searchers are frustrated when they cannot pinpoint why articles known to be relevant are missing from an initial retrieval set. We will describe our MeSH analysis methodology, a technique that helps craft more comprehensive searches, and the use of a new tool that saves time by doing this analysis automatically.

Methods: Librarians have long analyzed MeSH terms to improve searching, but the analysis process can be time-consuming. The Yale MeSH Analyzer, a web-based tool, saves time by automatically retrieving the MEDLINE metadata for up to 20 articles, and creating an easy-to-scan tabular grid. The grid helps validate search strategies by allowing quick identification of problems with the search. Librarians can easily scan the grid and identify appropriate MesH terms, missing terms, term variants, indexing consistency, and the reasons why some articles are retrieved and others are not. This inevitably leads to fresh iterations of the search strategy. In addition to MeSH terms, author-assigned keywords, article titles, and abstracts can be included in the analysis. A browser button for the toolbar is also available to do an analysis in native database interfaces such as PubMed.

Results: Since the MeSH Analyzer's release in mid-October 2015, there have been over 6,000 user sessions in 90 countries, with the heaviest use in the USA, Canada, the UK, Australia, Russia and the Netherlands. Systematic reviews which have incorporated results from the Yale MeSH Analyzer have already been published.

Conclusions: The Yale MeSH Analyzer helps refine searches, particularly those where indexing is challenging, by creating a quick, scannable grid of MeSH terms for easy review. The grid helps trouble-shoot searches and explain the search process to researchers. In the six months since its release, and with minimal promotion, it has become widely adopted both by librarians and end users to simplify search design for database searching.

Improving Access to Reports of Randomized Controlled Trials in Embase: Innovative Methods Enhance the Cochrane Central Register of Controlled Trials (CENTRAL) RESEARCH

Julie M. Glanville
Associate Director, York Health Economics Consortium Ltd, York, N/A, United Kingdom

Gordon Dooley
Director, Metaxis Ltd, Curbridge, United Kingdom

Anna Noel-Storr
Trials Search Coordinator, Cochrane Dementia and Cognitive Improvement Group, Oxford, United Kingdom

Ruth Foxlee
Information Specialist, Cochrane Editorial Unit, London, United Kingdom

Objectives: Systematic reviews rely on the efficient identification of research evidence, specificallyfrom randomised controlled trials (RCTs). The largest single source of reports of RCTs is the Cochrane Central Register of Controlled Trials (CENTRAL) in The Cochrane Library. Our objective was to develop a search filter to identify reports of RCTs from Embase, for inclusion in CENTRAL .

Methods: We developed, validated and refined a sensitive search filter to identify reports of RCTs in Embase for inclusion in CENTRAL. The filter was developed using textual analysis of ten randomly selected gold standard sets of RCT records from Embase (totalling 10,000 records published over ten years). The filter performance was tested on a second set of randomly selected 10,000 RCT reports. Following revisions to the filter, it was then validated on a third set of randomly selected 10,000 RCT reports. The performance of the filter was also tested against the previous Cochrane Embase RCT filter. The search filter was then used for one year to identify candidate RCT records from Embase. After one year the filter was refined based on an analysis of the records rejected over the previous year.

Results: The development of the search filter and the analysis of output from Embase has resulted in a tiered assessment process, where the most obvious RCT reports are fast-tracked for publication in CENTRAL, leaving more capacity to assess the relevance of less obvious candidate records. Over twelve months of operating the new filter 146879 records have been processed by the crowd and 60814 reports of RCTs identified. The revised filter was implemented in early 2015 and the results using the revised filter will be presented after February 1 2016.

Conclusions: The records identified by the filter and processed by Cochrane Collaboration volunteers has made many thousands of reports of RCTs unique to Embase, available in CENTRAL. These RCTs might be otherwise inaccessible to rsearchers conducting systematic reviews since many reviewers may not have access to Embase.
